# Isolation of Anionic *N*‐Heterocyclic Olefins and Their Versatile Reactivity

**DOI:** 10.1002/anie.202516374

**Published:** 2025-10-06

**Authors:** Prakash Duari, Alexander Linke, Margarita Shishkova, Quentin Le Dé, Arpan Das, Viktoria H. Gessner

**Affiliations:** ^1^ Faculty of Chemistry and Biochemistry Ruhr‐University Bochum Universitätsstrasse 150 44801 Bochum Germany

**Keywords:** Carbenes, Ligand design, Structure elucidation, Synthetic methodologies, Ylides

## Abstract

*N*‐Heterocyclic olefins (NHOs) have emerged as powerful bases and nucleophiles with broad applications in synthesis and catalysis. Here, we report a new synthetic approach to accessing their anionic derivatives through a formal ligand exchange reaction of metalated ylides or diazomethanes with free carbenes. The reaction was found to depend on the nature of the carbene, with more electrophilic carbenes reacting more readily via N_2_ and PPh_3_ extrusion, respectively. Spectroscopic and crystallographic analyses, combined with computational studies revealed that the anionic NHOs exhibit bent structures. In these structures, the anionic charge at the central carbon atom is partly delocalized into the carbene and the second substituent (Z). These stabilizing effects confer versatile reactivity at the central carbon atom, the nitrogen atom of the *N*‐heterocyclic carbene (NHC) or at the Z substituent. While the predominant reactivity occurs at the central carbon atom—enabling the convenient synthesis of functionalized NHOs—anionic NHOs also undergo a thermally induced rearrangement to *N*‐heterocyclic imines via a skeletal rearrangement involving ring‐opening of the *N*‐heterocyclic carbene.

## Graphical abstract

A new synthetic approach to anionic *N*‐heterocyclic olefins (NHOs) via ligand exchange of metalated ylides/diazomethanes with free carbenes is reported. The anionic NHOs adopt bent structures with delocalized anionic charge, which enables versatile reactivity, including the formation of highly functionalized NHOs, thermal rearrangement to *N*‐heterocyclic imines and generation of carbo(phosphino)carbene ligands.

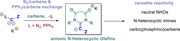

## Introduction

**Figure 1:**
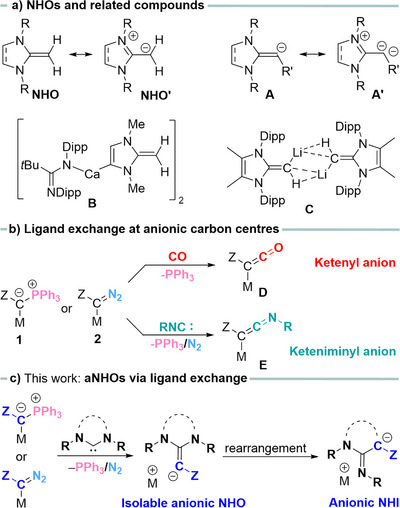
a) General structures of *N*‐heterocyclic olefins and their anionic derivatives and examples of anionic NHOs. b) Examples of ligand exchange reactions in metalated species. c) This work: Synthesis of anionic *N*‐heterocyclic olefin through ligand exchange reaction at metalated ylide or diazo by carbenes and their rearranged product.

*N*‐Heterocyclic olefins (NHOs) represent a unique class of alkenes featuring a strongly polarized carbon–carbon double bond directly bonded to an electron‐rich *N*‐heterocycle.^[^ ^1^, ^2^ ^]^ NHOs were first reported by Kuhn and coworkers in 1993,^[^ ^3^ ^]^ who already recognized the pronounced polarization of the exocyclic C═C bond, which results in an exceptional donor strength and high nucleophilicity of the exocyclic carbon atom as illustrated by the zwitterionic form NHO’ (Figure 1). Measurements of Tolman electronic parameters (TEPs) demonstrated that NHOs are even more potent electron donors compared to *N*‐heterocyclic carbenes (NHCs).^[^ ^4^ ^]^ This distinctive electronic structure of NHOs was highlighted by Rivard and coworkers in 2011, showcasing their potential for stabilizing reactive main group compounds.^[^ ^5^ ^]^ Since then, NHOs have demonstrated broad utility across diverse areas of chemistry, thanks to their strong donor properties.^[^ ^6^, ^7^, ^8^ ^]^ Their application spans transition metal and organocatalysis,^[^ ^9^ ^]^ main group,^[^ ^10^, ^11^ ^]^ coordination,^[^ ^12^, ^13^, ^14^, ^15^, ^16^ ^]^ and polymer chemistry^[^ ^17^, ^18^ ^]^ as well as ligand design.^[^ ^19^, ^20^, ^21^, ^22^, ^23^, ^24^ ^]^

Anionic NHOs with a deprotonated terminal C═C^−^R moiety (**A**, Figure 1) are promising reagents for the derivatization and facile introduction of NHOs via simple salt metathesis. However, while the chemistry of NHOs has been extensively studied, reports on their anionic derivatives remain scarce.^[^ ^25^, ^26^ ^]^ Initial reports by the Harder group demonstrated that backbone ring deprotonation of the *N*‐heterocyclic olefin is preferred over metalation of the CH_2_ moiety using lithium or calcium reagents, leading to complexes such as **B**.^[^ ^27^, ^28^ ^]^ To date, the only example of an isolated s‐block metal complex of an anionic NHO with a deprotonated CH_2_ moiety is lithium complex **C**, reported by Rivard and coworkers.^[^ ^29^ ^]^ This complex was synthesized from a backbone methylated NHO via sequential treatment with I_2_/KHMDS and *n*‐BuLi and proved to be a valuable precursor to main group^[^ ^10^, ^29^, ^30^, ^31^, ^32^ ^]^ and transition metal complexes.^[^ ^12^, ^33^, ^34^, ^35^, ^36^ ^]^

Given the ongoing interest in NHOs, an alternative approach to anionic NHOs that does not rely on pre‐prepared NHO precursors would provide a valuable entry into this chemistry. We hypothesized that the concept of ligand exchange reactions at carbon—an increasingly versatile strategy for generating novel reagents—could offer such an alternative.^[^ ^37^, ^38^ ^]^ This hypothesis is supported by previous reports from our group and others, demonstrating the facile exchange of PPh_3_ or N_2_ ligands at carbon by neutral ligands such as CO or isocyanides. For example, we disclosed an efficient synthetic approach to ketenyl anions through mild PPh_3_/CO exchange at the ylidic carbon center in α‐metalated ylides (Figure 1b).^[^ ^39^ ^]^ This synthetic route enabled the preparation of a series of ketenyl anions,^[^ ^40^, ^41^, ^42^, ^43^, ^44^ ^]^ which served as precursors to ketenes and various CO‐containing compounds. Liu and coworkers reported an analogous strategy to access ketenyl anions through an N_2_/CO exchange reaction in diazomethanides.^[^ ^45^ ^]^ Subsequently, this concept was transferred to PPh_3_/isonitrile^[^ ^46^ ^]^ and N_2_/isonitrile^[^ ^46^, ^47^ ^]^ exchange reactions to yield isolable keteniminyl anions, underscoring the synthetic potential of this methodology.

Building on these and other ligand exchange reactions involving low‐valent main group elements,^[^ ^48^, ^49^, ^50^, ^51^, ^52^, ^53^ ^]^ we aimed to generate anionic NHOs via a direct phosphine/carbene and N_2_/carbene exchange, respectively. While this reaction is elusive for anionic systems, its principal feasibility was demonstrated by Hansmann and coworkers for neutral diazoalkenes, resulting in the formation of vinylidene‐type compounds.^[^ ^54^, ^55^ ^]^ In addition, Severin and coworkers reported the formation of carbodicarbene complexes through denitrogenative carbene transfer from Fischer‐type carbene complexes to neutral diazoalkanes.^[^ ^56^, ^57^ ^]^ In this work, we establish a direct, synthetic pathway to anionic NHOs. We demonstrate that ligand exchange at the anionic carbon center is feasible but highly dependent on the nature of the carbene.^[^ ^58^, ^59^, ^60^ ^]^ Besides enabling the synthesis of anionic NHOs, this approach also provides access to carbo(phosphino)carbenes^[^ ^61^, ^62^, ^63^ ^]^ as well as *N*‐heterocyclic imines^[^ ^64^, ^65^ ^]^ through skeletal rearrangement.

## Results and Discussion

### Isolation and Characterization of Anionic NHOs and Their Rearrangement to N‐Heterocyclic Imines

We began our studies with the reaction of metalated ylide **1^PO^** with *N*‐heterocyclic carbenes and cyclic(alkyl)(amino)carbenes (CAAC), both in the absence and presence of 18‐crown‐6 (18‐c‐6). However, monitoring the reaction by ^31^P{^1^H} NMR spectroscopy in toluene showed no detectable conversion, even after heating to 80 °C, providing no evidence of ligand exchange under these conditions. We hypothesized that metalated diazo compounds **2** might be more susceptible to ligand exchange, given the superior leaving group ability of dinitrogen compared to phosphines.^[^ ^66^, ^67^ ^]^ However, also the reaction of **2^PO^** with NHCs or CAACs yielded no detectable conversion.

**Scheme 1:**
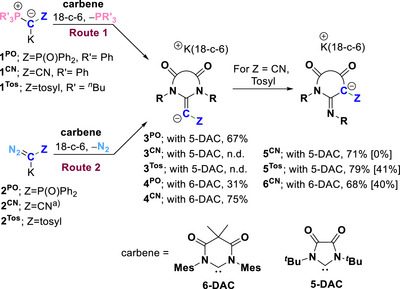
Synthetic routes to anionic *N*‐heterocyclic olefins **3** and **4** and their transformation to the rearranged products **5**. Yields in brackets refer to route 1, other yields to route 2. ^a)^ **2^CN^** was used as an isolated 18‐c‐6 complex. No additional 18‐C‐6 was added.

We postulated that the electrophilicity of carbenes is key to promoting ligand exchange by facilitating the nucleophilic attack of the metalated ylide or diazo compounds. Therefore, we switched to *N*,*N*′‐diamidocarbene **5‐DAC** (Scheme 1). Indeed, treatment of the metalated ylide **1^PO^** with **5‐DAC** in toluene in the presence of 18‐crown‐6 led to the formation of triphenylphosphine as detected by ^31^P{^1^H} NMR spectroscopy. However, multiple products were formed under these conditions, preventing the isolation of the targeted anionic NHO **3^PO^**. Using diazomethanide **2^PO^** instead of the yldiide, resulted in a noticeable gas evolution and formation of a deep orange solution, showing a single signal at *δ*_P_ = 3.2 ppm. The product exhibited reduced solubility and precipitated from the reaction mixture, allowing the isolation of the anionic NHO **3^PO^** as an orange solid in 67% yield. The structure of **3^PO^** was unambiguously confirmed by spectroscopic and crystallographic methods (see Figure 3 and the Supporting Information).

To probe the generality of the N_2_/carbene exchange, **2^PO^** was reacted with **6‐DAC** in THF in the presence of 18‐c‐6, resulting in an analogous gas evolution and a color change to deep orange. The product precipitated directly from the reaction mixture and could be isolated in a 31% crystalline yield. Although its characterization in solution was hindered by its poor solubility in THF and benzene and its instability in dichloromethane, acetonitrile, and DMSO, the identity of the anionic NHO **4^PO^** was unambiguously confirmed by X‐ray diffraction (XRD) analysis (Figure 3). It is noteworthy that the use of 18‐crown‐6 proved crucial for the successful isolation of the anionic NHOs. In its absence, rapid protonation to the H‐substituted NHO occurs (see below). Nevertheless, we were able to structurally characterize **4^PO^** in absence of 18‐crown‐6 (see Figure S58).

Next, we turned to the cyano‐substituted ylide **1^CN^**. Upon reaction with **6‐DAC** for 3 h at room temperature, clean formation of PPh_3_ was observed and subsequent workup afforded an off‐white solid in a 40% yield. Unexpectedly, the product was found to be the anionic *N*‐heterocyclic imine **6^CN^** instead of the olefin as evidenced by NMR spectroscopy and single‐crystal XRD analysis. The same product was formed when using diazomethanide **2^CN^**. Likewise, reaction of **2^CN^** with **5‐DAC** afforded the rearranged product **5^CN^** as a colorless solid in 71% yield and also the tosyl‐substituted reagents **1^Tos^** and **2^Tos^** underwent smooth transformation to the *N*‐heterocyclic imine **5^Tos^** when treated with **5‐DAC** in the presence of 18‐crown‐6.

**Figure 2:**
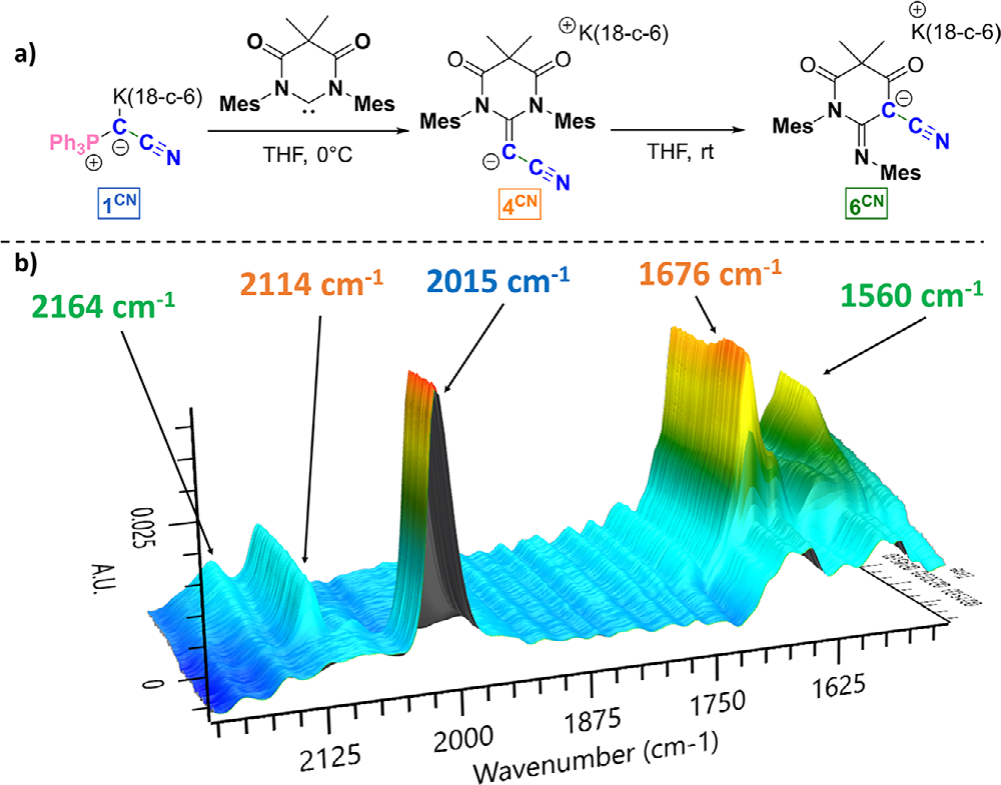
a) Reaction followed by in situ IR spectroscopy. b) Surface plot for the formation of **6^CN^** from **1^CN^** and **6‐DAC** via the intermediate **4^CN^**.

We hypothesized that the rearrangement to imines **5** and **6** proceeds via the anionic NHO **3** and **4**, respectively. To test this hypothesis, the reaction between **1^CN^** and **6‐DAC** in THF was monitored using real‐time in situ IR spectroscopy (Figure 2). After adding a THF solution of **6‐DAC** to a THF solution of **1^CN^** at 0 °C, the formation of a new species was observed featuring bands at 1676 and 2114 cm^−1^ corresponding to the carbonyl and the cyano group, respectively. This species—which we assumed to be the anionic NHO **4^CN^**—was unstable at 0 °C, and converted into product **6^CN^** characterized by new bands at 1560 and 2164 cm^−1^.

To unambiguously confirm the identity of the intermediate, we attempted its isolation by reacting **2^CN^** with **6‐DAC** at −30 °C. After stirring for 15 min, the reaction mixture was stored at low temperature overnight, affording yellow crystals of **4^CN^** in 75% yield. XRD analysis confirmed **4^CN^** as the anionic NHO. Notably, isolated **4^CN^** is stable at −30 °C, but undergoes slow conversion to **6^CN^** when warmed to room temperature, as confirmed by ^1^H NMR spectroscopy (see Figure S27). Having confirmed the anionic NHO as intermediate of the imine formation, we also tested the thermal stability of the phosphoryl‐substituted system. **3^PO^** is thermally stable up to 50 °C, but undergoes a rearrangement upon heating to 80 °C, affording a furan heterocycle, which presumably forms via the anionic *N*‐heterocyclic imine intermediate (see Figure S1). To investigate the effect of the cation on the stability of the anionic NHO, we examined the thermal stability of **3^PO^** with crown ether replaced by 2,2,2‐cryptand and lithium instead of the potassium salt. Both variations resulted in a decreased stability and rearrangement already at 50 °C (Figures S3 and S4). These observations suggest that the stability of anionic NHOs can be fine‐tuned by varying the cation. Both the choice of co‐ligands and the nature of the metal cation can be used to influence stability, presumably by modulating cation–anion interactions. Overall, these findings demonstrate that anionic NHOs are generally formed through N_2_/carbene and PPh_3_/carbene exchange, but that they are thermally unstable toward rearrangement of the carbene moiety.

To understand the different reactivities of the carbenes in the ligand exchange reactions, we calculated the reaction energies for the exchange of N_2_ in **2^CN^** with five different carbenes (r^2^SCAN‐3c/CPCM(THF), Table 1).^[^ ^68^ ^]^ The obtained energies show that the formation of the anionic NHOs is thermodynamically favored for all carbenes. This is especially true for carbenes with a low‐lying LUMO and small singlet–triplet energy gaps, such as the diamido **5‐DAC** or aryl amido carbene ^Mes^ArAmC. This suggests that the more electrophilic carbenes are active in the exchange reaction, a trend previously observed for CO activation by carbenes.^[^ ^69^ ^]^

**Table 1:** LUMO energies and adiabatic singlet–triplet energy gaps of five different singlet carbenes and the free reaction enthalpy Δ_R_ *G* ^°^ for the formation of the corresponding anionic NHOs (r^2^SCAN‐3c/CPCM(THF)).

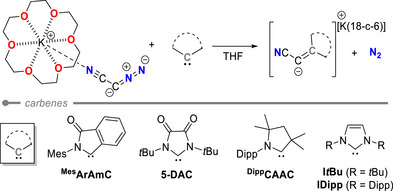			
Carbene	*E*_LUMO_ (eV)	*E*_S‐T_ (eV)	Δ_R_ *G*° (kcal mol^−1^)
^Mes^ArAmC	−3.76	1.22	−88.5
5‐DAC	−3.74	1.76	−62.0
^Dipp^CAAC	−1.12	4.09	−62.8
I*t*Bu	0.27	3.80	−33.4
IDipp	−1.08	4.09	−47.3

Interestingly, almost identical reaction energies of 62.8 and 62.0 kcal mol^−1^ were calculated for DAC and CAAC, indicating the thermodynamic viability of the formation of anionic NHOs with CAACs. However, no (NMR) spectroscopic evidence for their formation was observed with ^Dipp^CAAC, suggesting a kinetic inhibition of the reaction. This indicates that the transformation may be feasible when using less sterically hindered CAACs or more nucleophilic diazomethanides. Unfortunately, investigating the reaction mechanism and identifying the corresponding transition states for the exchange reaction proved challenging and was only successful for the carbenes 5‐DAC and I*t*Bu. These results indicate a stepwise process in which initially a nucleophilic attack of the diazomethanide (**2^CN^**) at the carbene carbon atom takes place, resulting in the formation of an intermediate adduct, which upon back‐donation from the carbene releases dinitrogen and generates the anionic NHO. The calculated activation barrier was found to require only 2.2 kcal mol^−1^ for 5‐DAC (Figure S75), which is well in line with the rapid formation of the NHO at room temperature. In contrast, I*t*Bu showed a considerably higher barrier of 44.3 kcal mol^−1^, confirming the kinetic preference of the exchange reaction for the more electrophilic carbene.

### Structures of the Anionic NHOs

With the isolated anionic NHOs **3** and **4** at hand, we analyzed their molecular properties and electronic structures. Previous reports explained the structural features of the related CO and N_2_ complexes, **D^PO^** and **2**, by the π‐accepting properties of the ligands according to the Dewar–Chatt–Duncanson model.^[^ ^42^, ^66^ ^]^ Unfortunately, NMR spectroscopic analysis of the anionic NHOs was complicated by their instability and low solubility in most common organic solvents and could only be achieved for the phosphinoyl‐substituted system **3^PO^**. This anionic NHO is characterized by a doublet at *δ*_C_ = 163.2 ppm for the C1 carbon atom in the ^13^C{^1^H} NMR spectrum (Table 2), which is downfield‐shifted compared to the diazomethanide **2^PO^** likely due to the stronger accepting properties of the diamidocarbene. The ^1^ *J*_PC_ coupling constant of 150.2 Hz is larger than that found in **2^PO^** arguing for a larger angle around the central carbon atom, but a slightly smaller one than that in ketenyl anion **D^PO^**. Interestingly, the [2.2.2]‐cryptand complex of **3^PO^** is characterized by an upfield‐shifted signal at 157.6 ppm (^1^ *J*_CP_ = 125.4 Hz), indicating an increased localization of negative charge at C1.

**Table 2:** Comparison of the NMR spectroscopic and crystallographic data of the anionic NHOs **3** and **4** and the related ketenyl anion **D^PO^** and diazomethanide **2^PO^**.

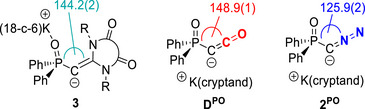				
	*δ* (P) (ppm)	*δ* (C1) (ppm)	^1^ *J*_PC_ (Hz)	Angle at C1 (^ο^)
**3^PO^**	3.1	163.2	150.2	144.2(2)
**4^PO^**	–	n.d.	n.d.	148.0(2)
**4^CN^**	–	n.d.	–	127.8(2)
**D^PO^** ^a)^	13.2	3.1	209.6	148.9(1)
**2^PO^** ^b)^	17.2	19.8	57.6	125.9(2)

^a)^ Structure and NMR parameters taken from Ref. [42].

^b)^ Structure and NMR parameters taken from Ref. [66].

**Figure 3:**
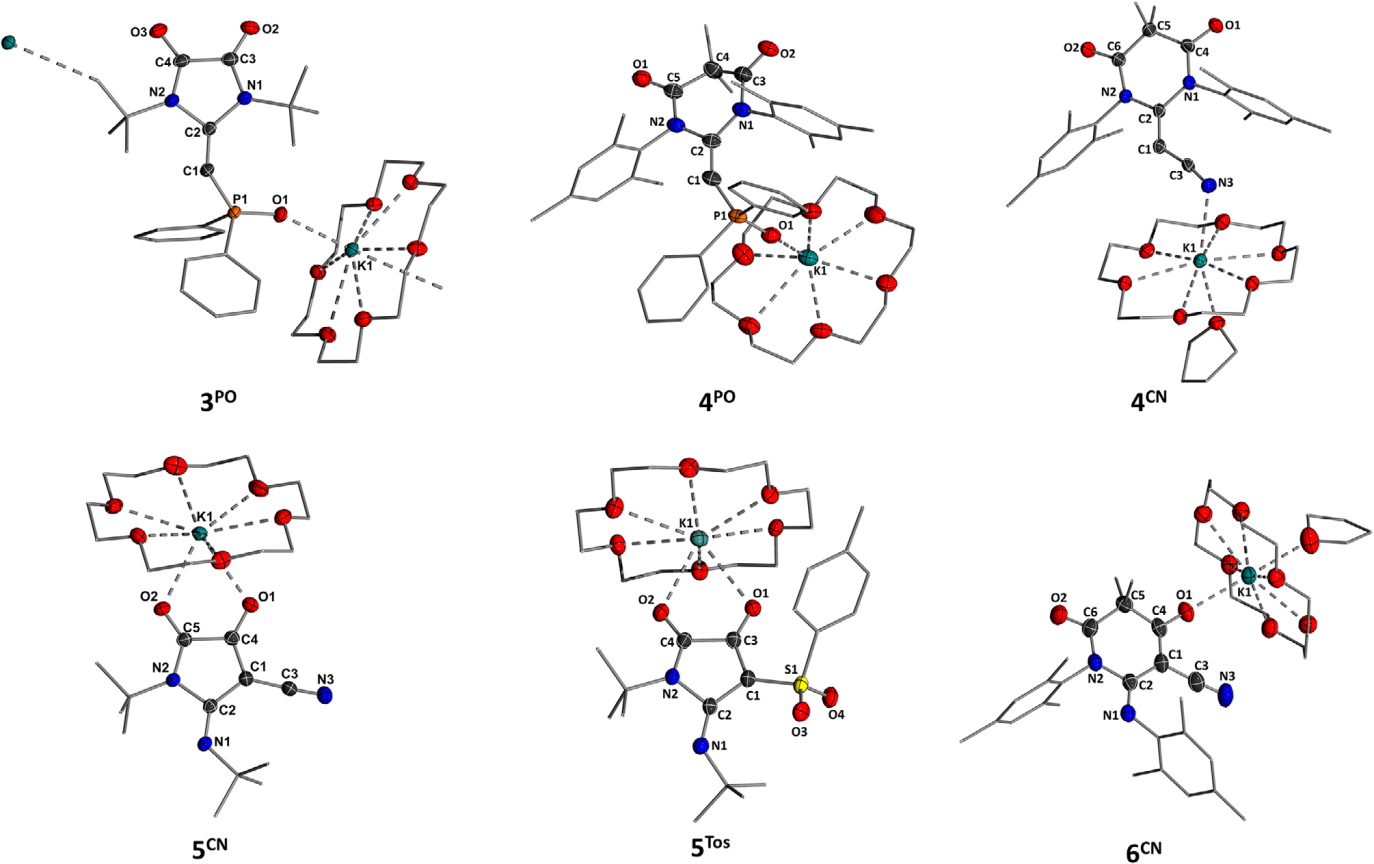
Molecular structure of anionic *N*‐heterocyclic olefins **3^PO^**, **4^PO^**, and **4^CN^** and imines **5^CN^**, **5** **^Tos^**, and **6^CN^** as their potassium crown ether complexes. Hydrogen atoms are omitted for clarity. Thermal ellipsoids are shown at the 50% probability level. Crystallographic details are given in Supporting Information.^[^ ^70^ ^]^

Single crystals of the anionic NHOs were obtained by vapor diffusion of pentane into a concentrated THF solution at −30 °C (**3^PO^**) or directly from the reaction mixtures (**4^PO^** and **4^CN^**, Figure 3).

XRD analysis revealed that the complexes feature no contact between the potassium cation and the exocyclic carbon atom C1. Instead, the metal is bound by the oxygen of the phosphinoyl group and cyano nitrogen, respectively. The C1─C2 bond lengths of 1.306(4)−1.329(3) Å are consistent with typical carbon─carbon double bonds, in line with the deshielded signal in the ^13^C NMR spectrum of **3^PO^**. The bond angles around the C1 carbon atom are measured to be 144.2(2)° and 148.0(2)° for **3^PO^** and **4^PO^**, respectively, while a notably smaller angle of 127.8(2)° is observed for **4^CN^**. In general, the angles of **3** and **4** are larger than the one reported for the diazo compound **2^PO^** and are in the range of the related ketenyl anion **D**. This trend is in line with the weaker acceptor properties of the N_2_ ligand and suggests similar ligand properties of the diamidocarbenes compared to CO. This corroborates well with trends indicated by NMR spectroscopy and recorded Tolman electronic parameters for DACs.^[^ ^4^ ^]^

**Figure 4:**
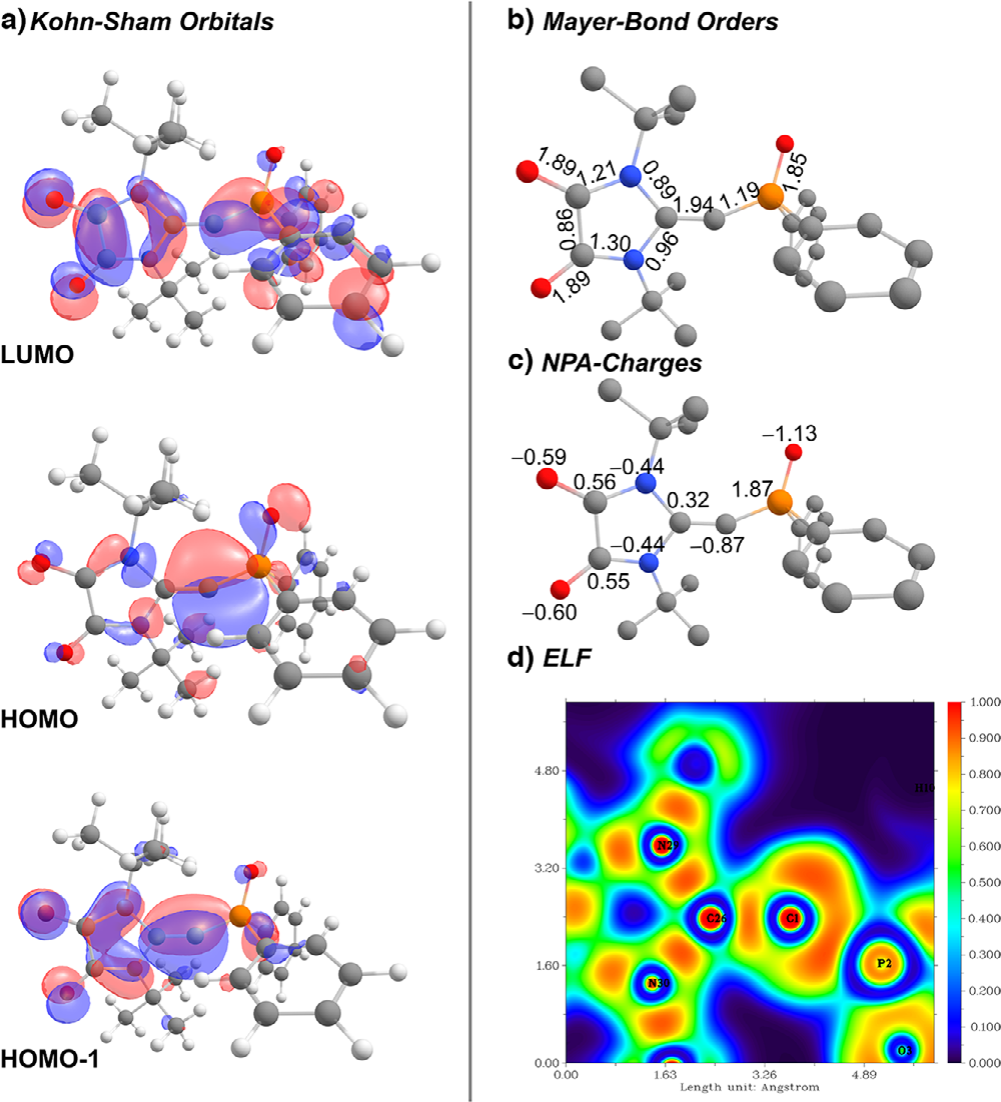
a) Kohn–Sham orbitals, b) Mayer bond orders, c) NPA charges, and d) ELF plot of the C–C–P linkage of **3^PO^** calculated at PBE0‐D4/ma‐def2‐TZVPP level of theory.

To gain further insights into the electronic structure of the anionic NHOs, we performed computational studies on **3^PO^** as model system (PBE0‐D4/ma‐def2‐TZVPP).^[^ ^71^, ^72^, ^73^, ^74^, ^75^ ^]^ Analysis of the Kohn–Sham frontier orbitals (Figure 4) of the free anion shows that the highest occupied molecular orbital (HOMO) is primarily localized at the C1 carbon atom. The HOMO1 corresponds to the double bond between C1 and the former carbene carbon atom, although it is polarized toward C1. This is consistent with the predominant description of **3** as an anionic olefin featuring a lone pair at C1, with a minor contribution from a carbone‐like structure possessing two lone pairs at C1 (**A’**, Figure 1). The lowest unoccupied molecular orbital (LUMO) is delocalized almost equally over the Z and the DAC group.

Natural bond orbital (NBO) analysis^[^ ^76^ ^]^ further supported the description of **3^PO^** as an anionic olefin, yielding a lone pair at the exocyclic carbon atom C1 and a σ‐bond to each substituent plus one π‐bond to the DAC ligand. This agrees well with the calculated bond indices (Mayer: 1.94; Wiberg: 1.97; NBO: 2.03) of the C1─C2 bond and the large negative natural charge at the C1 carbon atom (*q*_C_(NBO) = −0.87). The lone pair at C1 is—according to second‐order perturbation theory analysis—stabilized by charge delocalization into the antibonding P─O σ*‐orbital (*E* ^(2)^ = 25.5 kcal mol^−1^) and the antibonding C─N σ*‐orbital (Σ*E* ^(2)^ = 57.5 kcal mol^−1^) of the carbene fragment (c.f. Table S5). However, the electron localization function (ELF) clearly suggests the preference of the Lewis structure with a lone pair at the C1 carbon atom.^[^ ^77^ ^]^

### Reactivity of Anionic NHOs

Since the computational analyses indicated that the negative charge in the anionic NHOs is highly delocalized over the carbene and Z substituents, we next explored the reactivity of **3** and **4** with respect to the regioselectivity of their reaction with different electrophiles. Overall, the anionic NHOs turned out to be highly reactive and readily abstract a proton from the solvent, in particular from ethereal solvents. For example, preparation of **4^PO^** in a toluene/THF mixture selectively resulted in its protonation at the C1 carbon atom and the precipitation of NHO **7**, which could be isolated as a colorless solid in a high yield of 74%. The ^31^P{^1^H} NMR spectrum of **7** displays a resonance at 10.3 ppm, while the signal of the proton at the exocyclic carbon atom is observed as a doublet at *δ*_H_ = 4.66 ppm (^2^ *J*_HP_ = 4.1 Hz) in the ^1^H NMR spectrum. XRD analysis of **7** revealed an elongation of the C1─C2 bond from 1.310(4) in **4^PO^** to 1.353(6) Å and for the C1─P1 bond from 1.688(3) to 1.790(4) Å. In contrast, a slight contraction of the P1─O1 bond length (1.488(3) versus 1.503(2) Å) is observed. These bond length changes are well in line with the missing negative hyperconjugation effects in **7** compared to the anionic NHO.

C‐centered reactivity was also observed in the reaction of the anionic NHO **4^CN^** toward methyl iodide at −30 °C, confirming the utility of anionic NHOs as precursors to neutral NHOs. However, reaction of **3^PO^** with MeI in THF resulted in the methylation at the nitrogen atom, accompanied by C─N bond cleavage and ring‐opening to alkyne **9**. The reaction is markedly slower, requiring stirring overnight to reach completion. **9** is characterized by a ^31^P{^1^H} NMR resonance at *δ*_P_ = 3.76 ppm and was isolated as a colorless solid in a high yield of 83%. This unexpected transformation to **9** provides compelling evidence for considerable charge delocalization into the DAC framework. Due to the weaker anion‐stabilizing ability of the phosphinoyl moiety compared to the CN group (see Table S6), negative hyperconjugation effects lead to a stronger charge delocalization into the DAC moiety in **3^PO^** and thus to the methylation at nitrogen rather than at the C1 carbon.

Interestingly, a further reactivity pattern was observed in the reaction of **3^PO^** with trimethylsilyl chloride. The reaction proceeded smoothly to a new species, characterized by a high‐field shifted ^31^P{^1^H} NMR resonance at *δ*_P_ = −6.13 ppm. Subsequent XRD analysis revealed silylation at the oxygen atom of the phosphinoyl moiety, consistent with the high oxophilicity of silicon and the high negative charge at this position. Compound **10** formally represents a carbophosphinocarbene (CPC), which has been described as strongly donating carbon base. Similar to carbodiphosphoranes, CPCs feature a carbone character and can function as σ and π donor ligands owing to the two lone pairs located at the central carbon atom. However, the synthetic access to these compounds has been severely limited, with only a few examples reported to date.^[^ ^61^, ^62^, ^63^ ^]^ Therefore, the pathway to **10** represents a simple alternative to previously reported routes. The ^13^C{^1^H} NMR signal for the C1 carbon atom in CPC **10** resonates at 135.0 ppm (^1^ *J*_PC_ = 259.6 Hz), which is upfield shifted compared to literature systems due to the electron‐withdrawing DAC ligand. This implies that ligands with different electronic properties become accessible.

Despite the observed reactivity at the nitrogen and Z substituent to form **9** and **10**, the anionic NHOs predominantly reacted at the C1 carbon atom. This is further substantiated by the reaction of **3^PO^** with carbon dioxide,^[^ ^78^ ^]^ affording the anionic NHO−CO_2_ adduct **11**, which was isolated as a colorless solid in good yield of 75%. Similarly, the anionic carbon center of the anionic NHO **4^CN^** undergoes nucleophilic attack at the terminal nitrogen atom of mesityl azide, affording the anionic triazene derivative **12**. This compound was isolated as a bright yellow solid in an excellent yield of 80%. XRD analysis confirms the coordination of the potassium cation to the anionic triazene linkage. However, the ^1^H NMR spectrum recorded in THF reveals the presence of two isomeric species, likely arising from different modes of potassium ion coordination in solution (see Supporting Information).

**Figure 5:**
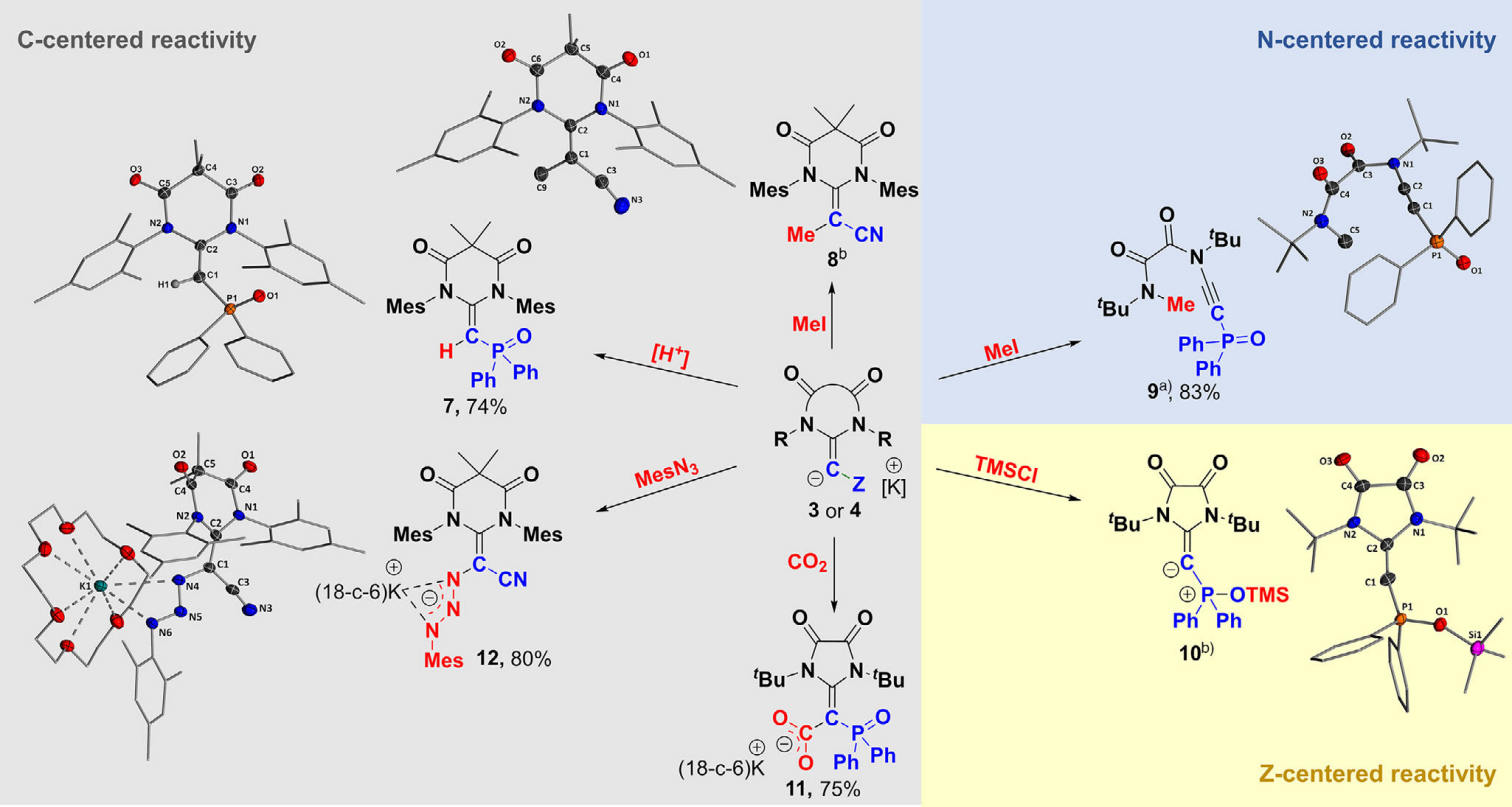
Reactivities of anionic NHOs toward different electrophiles. For details on reaction conditions, see the Supporting Information. Thermal ellipsoids are shown at the 50% probability level. Crystallographic details are given in the Supporting Information. ^a)^Crystal data of low quality.^b)^Yield cannot be obtained due to inseparable 18‐c‐6 complex of KI or KCl from the product.

In summary, Figure 5 illustrates the broad reactivity of anionic NHOs, showcasing their synthetic versatility. These reactivities enable the preparation of neutral NHOs, highly functionalized derivatives such as **12**, as well as *N*‐heterocyclic imines via a skeletal rearrangement. Moreover, they facilitate access to carbone‐like ligands as demonstrated by the synthesis of CPC **10**.

## Conclusion

In conclusion, we have reported the synthesis of anionic NHOs via ligand exchange, replacing the N_2_ or PPh_3_ ligand in metalated ylides and diazomethanes, respectively, with singlet carbenes. The reaction proceeds preferentially with electrophilic carbenes, which promote the nucleophilic attack by the metalated reagent. The anionic NHOs adopt bent structures with larger angles than those observed in diazomethanides, attributable to the enhanced acceptor ability of carbenes compared to dinitrogen. While their molecular structure is best described as an anionic olefin with a lone pair localized at the exocyclic carbon atom, the negative charge is delocalized into the carbene and the second substituent. This delocalization imparts versatile reactivity to anionic NHOs, primarily driven by C‐centered nucleophilicity but also possible at both substituents. Anionic NHOs are thermally unstable and undergo a unique carbene skeleton rearrangement to form anionic *N*‐heterocyclic imines. Alkylation occurs either at the excocyclic carbon or the carbene nitrogen atom, enabling access to highly functionalized molecules. Additionally, silylation at the second substituent has opened a new synthetic route to carbophosphinocarbene, a unique class of σ and π donor ligands. This rich chemical reactivity highlights the broad synthetic applicability of anionic NHO, which we will further explore in the future.

## Supporting Information

The authors have cited additional references within the Supporting Information.^[^ ^79^, ^80^, ^81^, ^82^, ^83^, ^84^, ^85^, ^86^, ^87^, ^88^, ^89^, ^90^, ^91^, ^92^, ^93^, ^94^, ^95^, ^96^, ^97^, ^98^, ^99^, ^100^, ^101^, ^102^, ^103^, ^104^, ^105^ ^]^

## Author Contributions

Prakash Duari planned the study and performed all synthetic experiments and spectroscopic analyses, except for compounds **8** and **5^Tos^**, which were synthesized by Margarita Shishkova and Arpan Das, respectively. Alexander Linke carried out all quantum chemical calculations. Quentin Le Dé helped Prakash Duari with the in situ IR spectroscopy and prepared the ReactIR figure. Viktoria H. Gessner supervised the entire study and assisted with data analysis. The manuscript was written by Prakash Duari, Alexander Linke, and Viktoria H. Gessner.

## Conflict of Interests

The authors declare no conflict of interest.

## Supporting information



Supporting Information

Supporting Information

## Data Availability

The data that support the findings of this study are available in the Supporting Information of this article.
