# Supplementary material for: Isolation of Anionic N‐Heterocyclic Olefins and Their Versatile Reactivity
Source: Angew Chem Int Ed Engl. 2025 Oct 6;64(48):e202516374. doi: 10.1002/anie.202516374 (PMC12643338; doi:10.1002/anie.202516374)
Supplement: Supplementary file 1 — Supporting Information [file ANIE-64-e202516374-s002.pdf]

Supporting Information

Isolation of Anionic N-heterocyclic Olefins and their Versatile Reactivity

Prakash Duari,[a] Alexander Linke,[a] Margarita Shishkova,[a] Quentin Le Dé,[a] Arpan Das[a] and Viktoria H. Gessner *[a]

[a]Faculty of Chemistry of Biochemistry, Ruhr-University Bochum, 44801 Bochum, Germany, Email: viktoria.gessner@rub.de

Table of Contents

1. Experimental Procedures 3
- 1.1. General Information 3
- 1.2. Synthesis of compound 3^{PO} 4
- 1.3. Synthesis of compound 4^{PO} 7
- 1.4. Synthesis of compound 4^{CN} 8
- 1.5. Synthesis of compound 5^{CN} 8
- 1.6. Synthesis of compound 5^{Tos} 8
- 1.7. Synthesis of compound 6^{CN} 9
- 1.8. Synthesis of compound 7 10
- 1.9. Synthesis of compound 8 10
- 1.10. Synthesis of compound 9 11
- 1.11. Synthesis of compound 10 11
- 1.12. Synthesis of compound 11 12
- 1.13. Synthesis of compound 12 13

2. NMR and IR spectra 14

3. Real-Time IR Spectroscopy 39
- 3.1. General Procedure 39
- 3.2. Monitoring of the formation of 6^{CN} 39

4. Crystal structure determination 41
- 4.1. General Information 41
- 4.2. Molecular Structure of 3^{PO} 46
- 4.3. Molecular Structure of 4^{PO} 46
- 4.4. Molecular Structure of 4^{PO} with THF 47
- 4.5. Molecular Structure of 4^{CN} 47
- 4.6. Molecular Structure of $5^{PO'}$ 48
- 4.7. Molecular Structure of 5^{CN} 48
- 4.8. Molecular Structure of 5^{Tos} 49
- 4.9. Molecular Structure of 6^{CN} 49
- 4.10. Molecular Structure of 7 50
- 4.11. Molecular Structure of 8 50
- 4.12. Molecular Structure of 9 51
- 4.13. Molecular Structure of 10 51
- 4.14. Molecular Structure of 12 52

5. Computational studies **53**
5.1. General remarks 53
5.2. Electronic structure of 3^{PO} 53
5.3. Thermodynamics of carbene exchange 61
5.4. Calculation of HOMO-LUMO gaps and Singlet-Triplet gaps 65
5.5. Thermodynamic calculation of trimethylsilyl addition to free anion 3^{CN} 67
5.6. Mechanism 68
5.7. Calculation of activation barriers for different singlet carbenes 71
5.8. Optimized structures (.xyz-files) 72
6. References **105**

1. Experimental Procedures

1.1. General Information

Chemical and Conditions

If not stated otherwise, all experiments were carried out using standard Schlenk techniques under an argon atmosphere, which was dry and free of oxygen. Argon (99.999%) was a product of *Air Liquide* and was used without any further drying. An MBraun SPS 800 was used to dry solvents before their usage (THF, toluene, DCM, ACN, *n*-pentane, *n*-hexane). All solvents were stored over molecular sieves under an argon atmosphere. Reagents were purchased from Sigma-Aldrich, ABCR, Acros Organics or TCI Chemicals and used without further purification if not stated otherwise.**5-DAC**[1] and **6-DAC**[2] were prepared following literature procedures. **1^{PO}**, **1^{CN}**, **1^{Tos}**, **2^{PO}**, **2^{CN}**, **2^{Tos}** were also synthesized following literature procedures.[3–5]

Caution! Strong bases such as organopotassium bases, especially as neat compounds, are severely air-/moisture-sensitive and pyrophoric organometallic compounds. These compounds need to be handled under an inert gas atmosphere to exclude reactions with oxygen and water. Guidelines for their handling can be found in literature: T. L. Rathman, J. A. Schwindeman, *Org. Process Res. Dev.* **2014**, *18*, 1192.

Analytical methods

NMR Spectroscopy. ^{1}H, ^{7}Li, ^{13}C{^{1}H}, ^{19}Si{^{1}H} ^{31}P{^{1}H} NMR spectra were recorded on Avance-III-400 spectrometers at 22 °C if not stated otherwise. All values of the chemical shift are in ppm regarding the δ-scale. All spin-spin coupling constants (*J*) are printed in Hertz (Hz). To display multiplicities and signal forms correctly the following abbreviations were used: s = singlet, d = doublet, t = triplet m = multiplet, dd = doublet of doublet, br = broad signal. Signal assignment was supported by, HSQC (^{1}H / ^{13}C), HMBC (^{1}H / ^{13}C, ^{1}H / ^{31}P) correlation experiments. The measurement conditions for each spectrum are provided in a text box in the corresponding figures included in Chapter 2.

IR spectra were recorded on a Shimadzu IRSpirit with QATR-S module in an argon filled glovebox. Measurement and processing details for all spectra: Temperature: 22 °C; Apodization function – Happ-Genzel, No. of Scans – 40, Resolution – 2 cm^{-1}.

Elemental analyses were performed on an Elementar vario MICRO cube elemental analyzer in our in-house facility.

HRMS-LIFDI mass spectra were measured in our in-house facility on a JEOL AccuTof GCv (JMS-T100GCV) (JEOL, Tokyo, Japan) instrument equipped with a LIFDI source from Linden (CMS, Weyhe, Germany). The emitter heating current was set to 20 mA min-1 at a constant rate.

XRD analyses. For details about the single-crystal Xray diffraction analyses, see chapter 4.

1.2. Synthesis of compound 3^{PO}

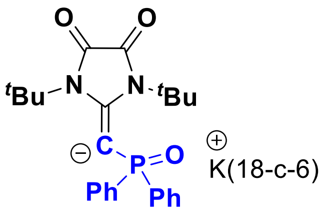

150 mg (0.535 mmol) $\mathbf{2^{PO}}$ and 141 mg (0.535 mmol) 18-c-6 were dissolved in 5 mL of THF and stirred for 5 min. To the resulting solution, 112 mg (0.535 mmol) **5-DAC** was added, upon which substantial N_2 evolution was observed with a color change to red. After stirring for 15 min, a red solid precipitated. The solution was decanted and subsequently, the red solid was washed with pentane. The solid was dried in *vacuo* to afford $\mathbf{3^{PO}}$ as a red solid. (259 mg, 0.356 mmol, 67%). Single crystals suitable for X-ray diffraction analysis were grown by slow vapor diffusion of pentane into a saturated solution of $\mathbf{3^{PO}}$ in THF at –30 °C.

^{1}**H NMR** (400 MHz, CD_2Cl_2): δ = 7.81 – 7.74 (m, 4H, $CH_{Ph,ortho}$), 7.30 – 7.21 (m, 6H, $CH_{Ph,meta,para}$), 3.59 (s, 24H, $CH_{2,crown}$), 1.71 (s, 18H, N-C(CH_3)$_3$). ^{13}**C{^{1}H} NMR** (101 MHz, CD_2Cl_2): δ = 163.2 (d, $^{1}J_{CP}$ = 150.2 Hz, C=$C^{(-)}$-P), 161.0 (s, *C*=O), 142.9 (d, $^{1}J_{CP}$ = 127.8 Hz, $C_{Ph,ipso}$), 132.3 (d, $^{2}J_{CP}$ = 8.7 Hz, $C_{Ph,ortho}$), 128.4 (d, $^{4}J_{CP}$ = 2.6 Hz, $C_{Ph,para}$), 127.5 (d, $^{3}J_{CP}$ = 11.7 Hz, $C_{Ph,meta}$), 112.1 (d, $^{2}J_{CP}$ = 23.1 Hz, *C*=C-P), 70.4 (s, $CH_{2,crown}$), 57.7 (s, N-*C*(CH_3)$_3$), 30.1 (s, N-C(CH_3)$_3$). ^{31}**P{^{1}H} NMR** (400 MHz, CD_2Cl_2): δ = 3.07 (s, *P*Ph_2O) ppm. **FT-IR** (ATR, cm^{-1}): 2959.9 (bw), 2898.2 (bw), 2871.6 (bw), 1711.9 (s), 1647.3 (s), 1472.1(w), 1435.4 (w), 1348.6 (m), 1311.2 (w), 1245.2 (s), 1130.9 (m), 1105.1 (s), 965.8 (m), 965.8 (w), 867.4 (w), 711.6 (s), 634.1(m), 567.3 (m), 547.9 (s) 424.4(m). **Anal. Calcd** for $C_{36}H_{52}KN_2O_9P$: C, 59.49; H, 7.21; N, 3.85. Found: C, 59.50; H, 6.94; N, 3.63.

Stability of 3^{PO}:

Compound $\mathbf{3^{PO}}$ is stable at room temperature inside a glove box for months. $\mathbf{3^{PO}}$ in C_6D_6 is stable up to 50 °C temperature. However, when $\mathbf{3^{PO}}$ in C_6D_6 is heated to 80 °C, it converts to a new product, as observed by ^{31}P{^{1}H} NMR spectroscopy (Figure S2). Diffusion of hexane into the reaction mixture yields colorless crystals, which are suitable for XRD analysis, confirming a rearranged compound $\mathbf{5^{PO\prime}}$ containing a furan moiety. The crystal quality was not good for discussing bonding parameters (Figure S60). The formation of $\mathbf{5^{PO\prime}}$ is assumed to go through the intermediate $\mathbf{5^{PO}}$.

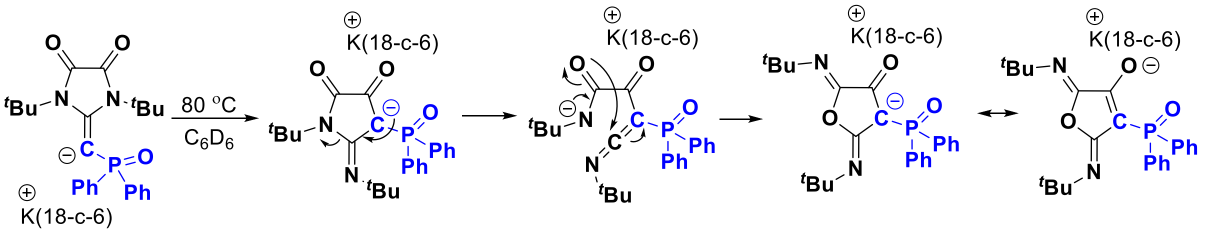

$\mathbf{3^{PO}}$ $\mathbf{5^{PO}}$ $\mathbf{5^{PO\prime}}$

Figure S1 Plausible mechanism for the formation of $\mathbf{5^{PO\prime}}$ after heating $\mathbf{3^{PO}}$ at 80 °C in C_6D_6.

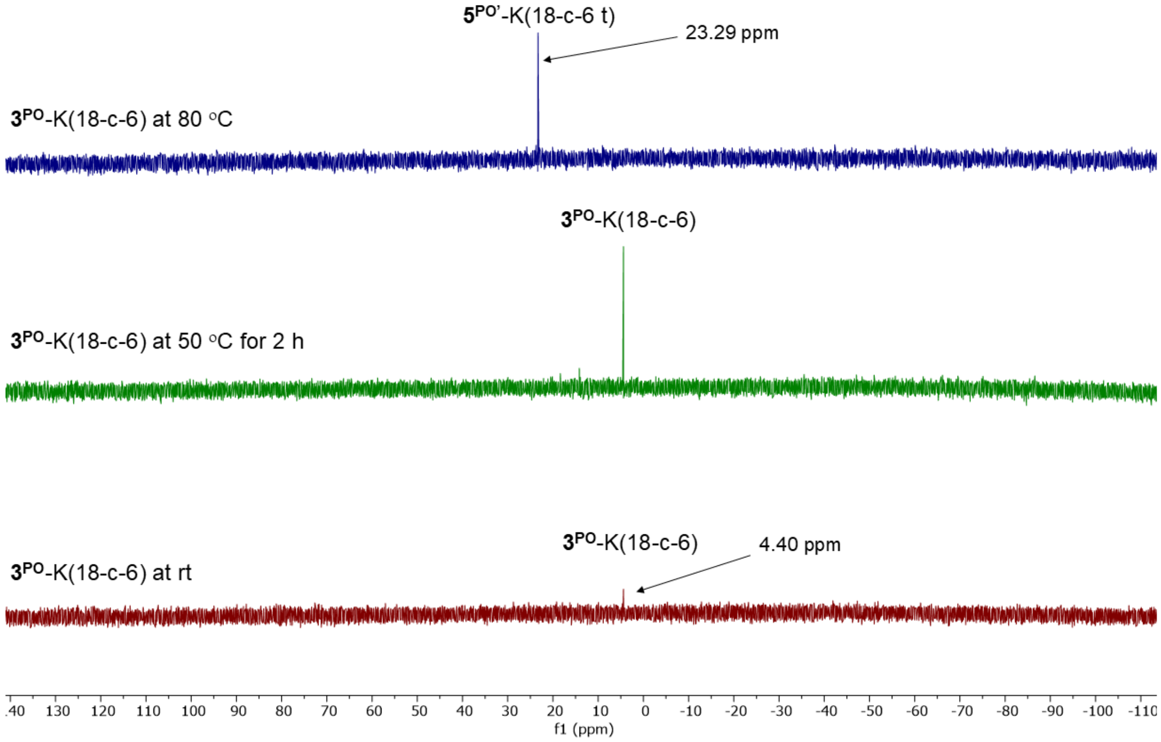

Figure S2 Monitoring of the conversion of $\mathbf{3^{PO}}$ in presence of 18-c-6 into $\mathbf{5^{PO'}}$ by $^{31}P\{^1H\}$ NMR spectroscopy in C_6D_6. Note: The solubility of $\mathbf{3^{PO}}$-K(18-c-6) is low at room temperature but increases when heated at 50 °C.

$\mathbf{3^{PO}}$ without 18-c-6:

15 mg (0.0535 mmol) $\mathbf{2^{PO}}$ was dissolved in 0.6 mL THF-d8 inside a J-Young tube. 11.2 mg (0.0535 mmol) **5-DAC** was added to the solution, upon which N_2 evolution was observed. The solution turned red. ^{31}P NMR spectroscopy showed full conversion of the reactant to product. Isolation of the compound as a solid was not possible from the solution due to the high reactivity of the compound. NMR spectroscopy studies of the reaction mixture confirmed the product formation.

^{1}H NMR (400 MHz, THF-d_8): δ = 7.79 – 7.72 (m, 4H, C$H_{Ph,ortho}$), 7.22 – 7.13 (m, 6H, C$H_{Ph,meta,para}$), 1.69 (s, 18H, N-C(CH_3)$_3$). **^{13}C{^{1}H} NMR** (101 MHz, THF-d_8): δ = 163.8 (d, $^{1}J_{CP}$ = 152.7 Hz, C=$C^{(-)}$-P), 161.8 (s, *C*=O), 143.4 (d, $^{1}J_{CP}$ = 129.6 Hz, $C_{Ph,ipso}$), 133.0 (d, $^{2}J_{CP}$ = 8.7 Hz, $C_{Ph,ortho}$), 128.8 (d, $^{4}J_{CP}$ = 2.6 Hz, $C_{Ph,para}$), 127.9 (d, $^{3}J_{CP}$ = 11.9 Hz, $C_{Ph,meta}$), 112.4 (d, $^{2}J_{CP}$ = 23.5 Hz, *C*=C-P), 58.3 (s, N-*C*(CH_3)$_3$), 30.5 (s, N-C(*C*H_3)$_3$). **^{31}P{^{1}H} NMR** (400 MHz, THF-d_8): δ = 3.23 (s, *P*Ph_2O) ppm.

$\mathbf{3^{PO}}$ with [2.2.2]-Cryptand:

In a J-Young tube, 20 mg (0.0714 mmol) $\mathbf{2^{PO}}$ were dissolved in 0.6 mL THF-d8 and 26.9 mg (0.0714 mmol) of [2.2.2]-cryptand were added. 15 mg (0.0714 mmol) **5-DAC** were added to the solution, upon which N_2 evolution was observed together with a color change to red. NMR spectroscopic studies of the reaction mixture confirmed the formation of the product with a

signal at 4.04 ppm. This compound is stable at room temperature; however, it starts converting to the rearranged product **$5^{PO'}$** after heating for 2 h at 50 °C, which is evidenced by the appearance of a new peak at 23.47 ppm in the $^{31}P\{^{1}H\}$ NMR spectrum. Complete transformation is observed upon heating to 80 °C (Figure S3).

^{1}H NMR (400 MHz, THF-d_8): δ = 7.94 – 7.84 (m, 4H, C$H_{Ph,ortho}$), 7.17 – 7.07 (m, 6H, C$H_{Ph,meta,para}$), 3.55 (s, C$H_{2,cryptand}$), 3.51 (t, $^{3}J_{HH}$ = 4.8 Hz, C$H_{2,cryptand}$), 2.53 (t, $^{3}J_{HH}$ = 4.8 Hz, C$H_{2,cryptand}$), 1.73 (s, 18H, N-C(CH_3)$_3$). **$^{13}C\{^{1}H\}$ NMR** (101 MHz, THF-d_8): δ = 160.6 (s, *C*=O), 157.6 (d, $^{1}J_{CP}$ = 125.4 Hz, C=$C^{(-)}$-P), 146.0 (d, $^{1}J_{CP}$ = 123.5 Hz, $C_{Ph,ipso}$), 133.5 (d, $^{2}J_{CP}$ = 8.0 Hz, $C_{Ph,ortho}$), 128.1 (d, $^{4}J_{CP}$ = 2.5 Hz, $C_{Ph,para}$), 127.4 (d, $^{3}J_{CP}$ = 11.1 Hz, $C_{Ph,meta}$), 114.6 ((d, $^{2}J_{CP}$ = 18.4 Hz, *C*=C-P), 71.5 (s, *C*$H_{2,cryptand}$), 69.0(s, *C*$H_{2,cryptand}$), 57.3 (s, N-*C*(CH_3)$_3$),55.5 (s, *C*$H_{2,cryptand}$), 30.6 (s, N-C(*C*H_3)$_3$). **$^{31}P\{^{1}H\}$ NMR** (400 MHz, THF-d_8): δ = 4.04 (s, *P*Ph_2O) ppm.

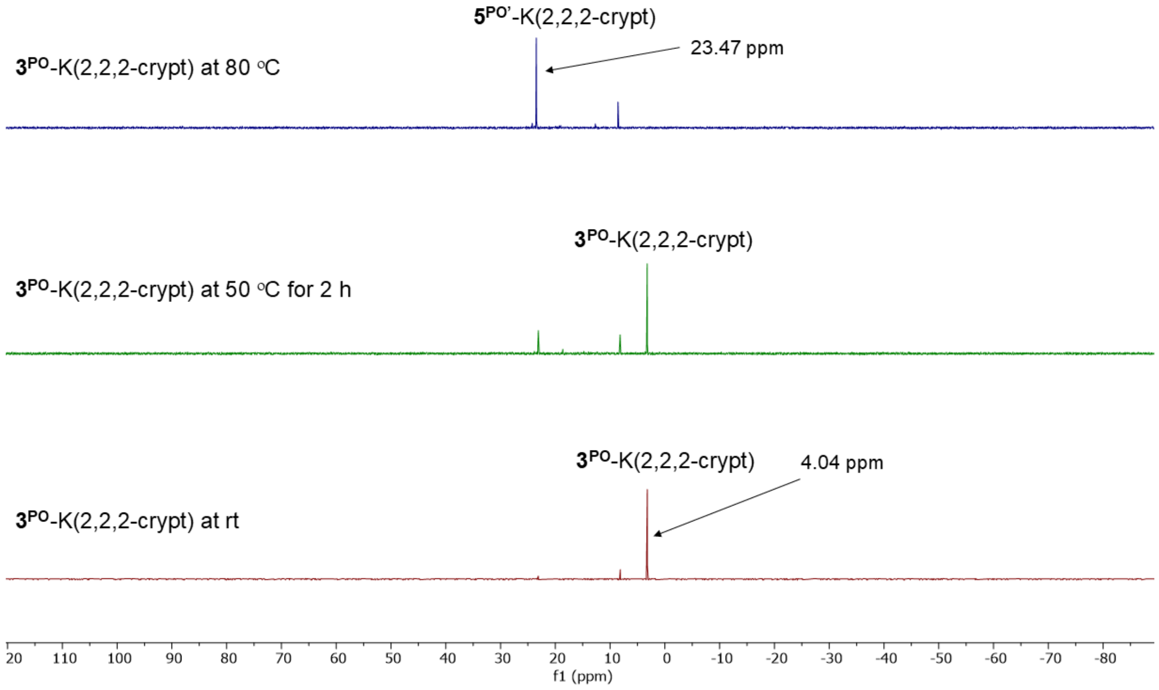

Figure S3 Monitoring of the conversion of **3^{PO}** in presence of [2.2.2]-cryptand into **$5^{PO'}$** by $^{31}P\{^{1}H\}$ NMR spectroscopy in THF-d8.

The lithium salt of 3^{PO}:

40 mg (0.0839 mmol) **$_{H}1^{PO}$** were dissolved in 0.7 mL THf-d_8. 9.36 mg (0.0847 mmol) LDA were added and the mixture stirred for 20 min to in situ form **1^{PO}-Li**. After filtration into a J-Young NMR tube, N_2O was purged into the solution. The formation of **2^{PO}-Li** was observed by a peak at 23.1 ppm in the ^{31}P NMR spectrum along with the formation of $POPh_3$ at 25.4 ppm. To the obtained solution, 17.6 mg (0.0839 mmol) **5-DAC** were added, which resulted in visible N_2 evolution accompanied by a color change to red. The formation of **3^{PO}-Li** was confirmed by a signal at 3.45 ppm in the $^{31}P\{^{1}H\}$ NMR spectroscopy. **3^{PO}-Li** is stable at room temperature. However, it starts converting to the rearranged product **$5^{PO'}$** after heating for 2 h at 50 °C.

$^{31}P\{^1H\}$ NMR spectroscopy clearly showed the consumption of **3^{PO}-Li**, which quickly completed after heating to 80 °C (Figure S4).

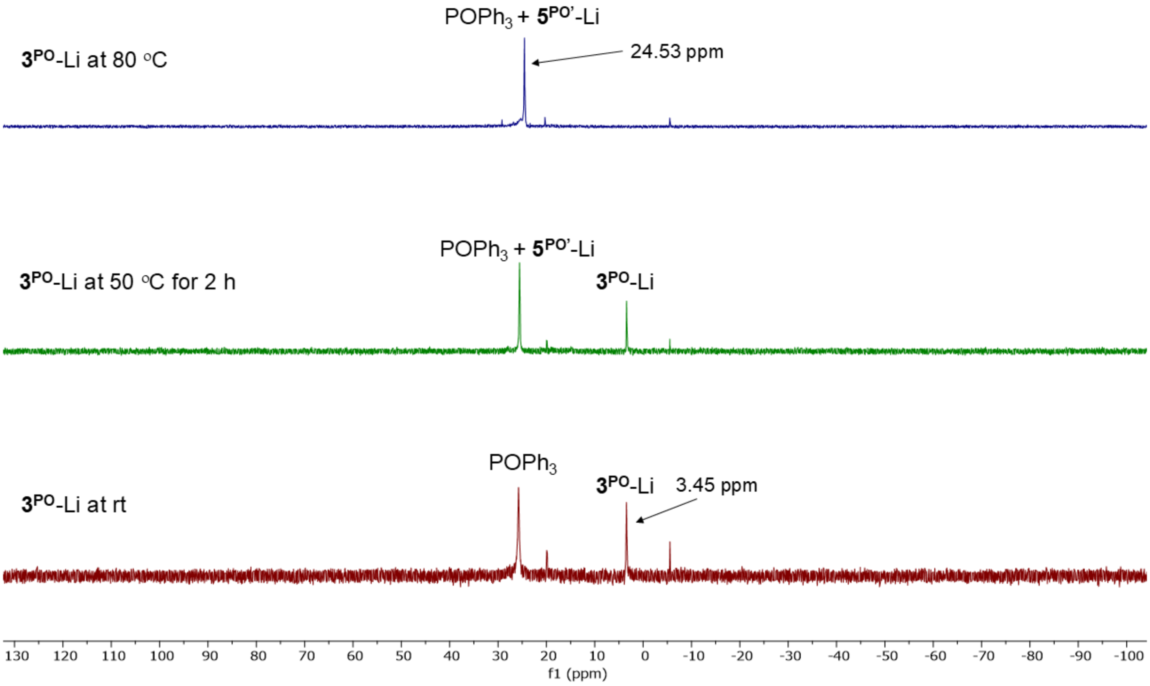

Figure S4 Monitoring of the conversion of lithium salt of **3^{PO}** into **5$^{PO'}$** by $^{31}P\{^1H\}$ NMR spectroscopy in THF-d8.

1.3. Synthesis of compound 4^{PO}

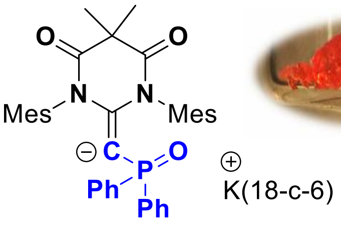

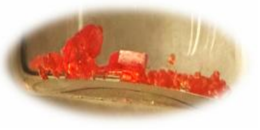

30 mg (0.107 mmol) **2^{PO}** and 40.3 mg (0.107 mmol) 18-c-6 were dissolved in 1 mL of THF inside a J-Young tube and stirred for 5 min. To the resulting solution, 28.3 mg (0.107 mmol) **6-DAC** was added, upon which substantial N_2 evolution was observed. After 1 h, orange crystals formed inside the tube, which were washed with 1 mL THF (x1) and 1 mL pentane (x2). The orange crystals were dried in *vacuo* to afford **4^{PO}**. (29.5 mg, 0.033 mmol, 31%). Single-crystal X-ray diffraction analysis was performed on those crystals.

Solution-phase spectroscopic data are missing because of the insolubility of **4^{PO}** in THF, toluene, and benzene, as well as its high reactivity towards other solvents. Even if **4^{PO}** is kept in THF for a longer time, the protonated compound **7** is formed. **FT-IR** (ATR, cm^{-1}): 2909.7 (bw), 2866.6 (bw), 1633.6 (m), 1560.4 (w), 1472.1 (w), 1350.71 (m), 1249.5 (w), 1105.1 (s), 1027.6 (s), 960.1 (m), 910.5 (w), 838.0 (m), 698.7 (s), 589.5 (w), 528.5 (s), 520.6 (s), 455.3 (m). **Anal. Calcd** for $C_{49}H_{62}KN_2O_9P$: C, 65.90; H, 7.00; N, 3.14. Found: C, 65.39; H, 7.10; N, 2.84.

1.4. Synthesis of compound 4^{CN}

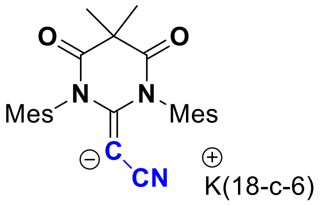

The toluene (2 mL) solution of 58 mg (0.154 mmol) was added to the THF solution (0.5 mL) of 100 mg (0.154 mmol) $\mathbf{2^{CN}}$(18-c-6)(O=PPh_3) in a vial. Gas evolution was noticed in the vial. After 15 minutes of stirring, the vial was kept in the freezer overnight, yielding yellow-colored crystals. The crystals were washed with cold toluene and cold pentane and dried in vacuo to afford $\mathbf{4^{CN}}$ as yellow crystals. (112 mg, 0.115 mmol, 75%). Single-crystal X-ray diffraction analysis was done with that crystal. Note: $\mathbf{4^{CN}}$ is only stable at –30 °C and can be stored at –30 °C for several months.

^{1}H NMR (400 MHz, THF-d_8): δ = 6.71 (s, 4H, CH_{Mes}), 2.21 (s, 6H, $CH_{3,para,\text{N-Mes}}$), 2.14 (s, 12H, $CH_{3,ortho,\text{N-Mes}}$), 1.51 (s, 6H, $(CH_3)_{2,DAC}$) ppm. **^{13}C{^{1}H} NMR** is missing because of its rearrangement to imine at room temperature. **FT-IR** (ATR, cm^{-1}): 2910.4 (bw), 2861.6 (bw), 2071.7 (m, CN stretching), 1967.5 (w), 1678.5 (m), 1643.7 (m), 1602.0 (m), 1469.9 (w), 1406.7 (m), 1348.6 (s), 1235.1 (w), 1103.7 (s), 1012.5 (w), 960.1 (m), 783.4 (w), 720.2 (w), 568.0 (w), 529.2 (w), 501.2 (w). **Anal. Calcd** for $C_{42}H_{60}KN_3O_9$: C, 63.85; H, 7.65; N, 5.32. Found: C, 63.98; H, 6.13; N, 5.42.

1.5. Synthesis of compound 5^{CN}

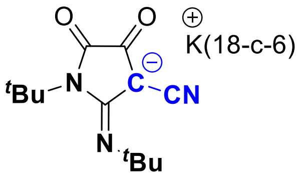

100 mg (0.154 mmol) $\mathbf{2^{CN}}$(18-c-6)(O=PPh_3) was dissolved in 2 mL THF, and 32.4 mg (0.154 mmol) **5-DAC** was dissolved in 2 mL THF. They were mixed together, resulting in the evolution of gas. The resulting reaction mixture was stirred at room temperature for 30 min. Vapor diffusion of hexane to the reaction mixture, resulting in the colorless crystals of $\mathbf{5^{CN}}$, which were filtered off and dried in vacuo. (60 mg, 0.109 mmol, 71%). Single-crystal X-ray diffraction analysis was performed on those crystals.

^{1}H NMR (400 MHz, THF-d_8): δ = 3.63 (s, 24H, $CH_{2,crown}$), 1.66 (s, 9H, N-C(CH_3)$_3$), 1.41 (s, 9H, C=N-C(CH_3)$_3$). **^{13}C{^{1}H} NMR** (101 MHz, THF-d_8): δ = 178.1 (s, N-*C*=O), 167.9 (s, C-*C*=O), 152.2 (s, ^{t}Bu-N-*C*=N-^{t}Bu), 121.4 (s, *C*N), 71.1 (s, $CH_{2,crown}$), 69.1 (s, CO-$C^{(-)}$-CN), 58.0 (s, N-*C*(CH_3)$_3$), 53.9 (s, C=N-*C*(CH_3)$_3$), 32.4 (s, C=N-C(CH_3)$_3$), 31.4 (s, N-C(CH_3)$_3$). **FT-IR** (ATR, cm^{-1}): 2902.5 (w), 2891.7 (w), 2180.1 (m, CN stretching), 1700.4 (m), 1617.1 (m), 1607.06 (m), 1472.8 (w), 1401.7 (w), 1352.1 (s), 1285.4 (w), 1252.3 (w), 1102.3 (s), 1027.6 (w), 961.5 (s), 838.0 (m), 781.3 (w), 699.4 (m), 566.6 (m), 471.1 (w). **Anal. Calcd** for $C_{25}H_{42}KN_3O_8$: C, 54.42; H, 7.67; N, 7.62. Found: C, 53.97; H, 7.60; N, 5.56.

1.6. Synthesis of compound 5^{Tos}

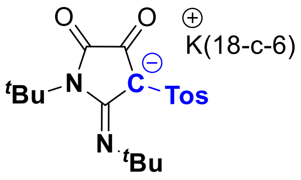

Path A: Inside an argon-filled glovebox, 40 mg (0.108 mmol) of $\mathbf{1^{Tos}}$ was taken in a vial in 2 mL Et_2O and cooled down to -30 °C. Benzyl potassium (1.5) equivalent was added to it and kept it stirring for 30 minutes at room temperature inside the glovebox. The solution becomes clear and 13.6 mg (0.0648 mmol) of carbene was added to it. The reaction mixture was stirred overnight. The reaction mixture was transferred to a 25 mL Schlenk tube, dried under vacuum and dissolved in THF. The reaction mixture was kept at room temperature inside glovebox. THF evaporated and needle shape crystals were obtained

along with a red oily compound. The red oil was washed with (3 × 0.4 mL) cold toluene (-30 °C) and dried under vacuum to get **5^{Tos}** as an off-white powder. (25 mg, 41 %).

Path B: Inside an argon filled glovebox, the **2^{Tos}** (15.4 mg, 0.0658 mmol) was added with 18-c-6 (17.4 mg, 0.0658 mmol) in 0.6 mL C_6D_6 in a vial. The carbene (12.6 mg, 0.0598 mmol) was then added to it. N_2 gas evolution was observed and the reaction mixture became dark red. The reaction mixture was left stirring for 12 hours at RT in NMR tube. The ^{1}H NMR shows clean formation of a single compound with a little impurity in aliphatic region. The reaction mixture in C_6D_6 was taken inside glovebox and pentane was added to it to precipitate the expected compound and the supernatant liquid was discarded. The process was repeated three times. The precipitate was then dried under vacuum to obtain 5Tos as an off-white powder (32 mg, 79%).

^{1}H NMR (400 MHz, CD_2Cl_2): δ = 7.84 (d, 2H, $^3J_{HH}$ = 8.2 Hz, $CH_{Tol,o}$), 7.10 (d, 2H, $^3J_{HH}$ = 8.2 Hz, $CH_{Tol,m}$), 3.59 (s, 24H, $CH_{2,crown}$ + $CH_{2,THF}$), 2.31 (s, 3H, $CH_{3,Tol}$), 1.64 (s, 9H, N-C(CH_3)$_3$), 1.43 (s, 9H, C=N-C(CH_3)$_3$). **^{13}C{^{1}H} NMR** (101 MHz, THF-d$_8$): δ = 175.8 (s, N-*C*=O), 166.9 (s, C-*C*=O), 146.8 (s, $C_{Tol,p}$), 145.5 (s, ^{t}Bu-N-*C*=N-^{t}Bu), 140.8 (s, S-$C_{Tol,ipso}$), 128.8 (s, $C_{Tol,m}$) , 127.7(s, $C_{Tol,o}$), 96.6 (s, CO-$C^{(-)}$-Tos), 71.1 (s, $CH_{2,crown}$), 57.5 (s, N-*C*(CH_3)$_3$), 55.7 (s, C=N-*C*(CH_3)$_3$), 31.9 (s, C=N-C(CH_3)$_3$), 31.1 (s, N-C(CH_3)$_3$), 21.5(s, $C_{Tol,Me}$). **FT-IR** (ATR, cm^{-1}): 2958.5 (w), 2896.7 (w), 1708.3 (w), 1667.4 (w), 1623.6 (m), 1476.4 (w), 1351.4 (m), 1264.5 (m), 1217.9 (w), 1102.9 (s), 1013.2 (w), 985.2 (w), 962.9 (s), 838.0 (m), 812.9 (w), 764.8 (w), 688.6 (m), 662.1 (w), 607.5 (m), 435.9 (w), 424.4 (w). **Anal. Calcd** for $C_{31}H_{49}KN_2O_{10}S$: C, 54.68; H, 7.25; N, 4.11; S, 4.71. Found: C, 54.91; H, 7.40; N, 4.05; S, 4.31.

1.7. Synthesis of compound 6^{CN}

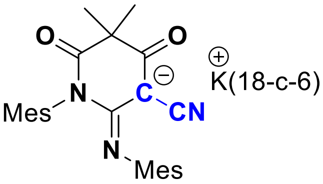

Path A: 150 mg (0.442 mmol) **1^{CN}** and 117 mg (0.442 mmol) 18-c-6 were dissolved in 10 mL THF and stirred for 10 min. To the resulting red solution, 166 mg (0.442 mmol) of **6-DAC** was added, and the mixture was stirred for 3 h. ^{31}P NMR spectroscopy shows the release of PPh_3. The solution was concentrated to 3 mL and the off-white precipitation formed. The solid was filtered and washed with pentane (2 times). After drying in vacuo, **6^{CN}** was obtained as an off-white solid. (141 mg, 0.175 mmol, 40 %).

Path B: 30 mg (0.038 mmol) **4^{CN}** was dissolved in 0.6 mL THF-d$_8$ and stirred overnight. The new product formation was confirmed by ^{1}H NMR spectroscopy. Vapor diffusion of pentane into the THF solution resulted in the formation of colorless crystals of **6^{CN}**, which were filtered off and dried in vacuo. (27 mg, 0.034 mmol, 90 %). Overall yield from the diazomethanide **2^{CN}** is 68%.

^{1}H NMR (400 MHz, CD_2Cl_2): δ = 6.92 (s, 2H, CH_{Mes}), 6.65 (s, 2H, CH_{Mes}), 3.70 – 3.66 (m, THF-OCH_2), 3.58 (s, 24H, $CH_{2,crown}$), 2.30 (s, 3H, $CH_{3,para,N\text{-}Mes}$), 2.19 (s, 3H, $CH_{3,para,C=N\text{-}Mes}$), 2.15 (s, 6H, $CH_{3,ortho,N\text{-}Mes}$), 1.97 (s, 6H, $CH_{3,ortho,C=N\text{-}Mes}$), 1.84 – 1.80 (m, 2H, THF-OCH$_2$CH_2), 1.46 (s, 6H, (CH_3)$_{2,DAC}$. **^{13}C{^{1}H} NMR** (101 MHz, CD_2Cl_2): 191.0 (s, N-*C*=O), 176.0 (s, C-*C*=O), 149.2 (s, Mes-N-*C*=N-Mes), 145.2 (s, $C_{ipso,N\text{-}Mes}$) 136.9 (s, *C*-$CH_{3,para,N\text{-}Mes}$), 136.1 (s, *C*-$CH_{3,ortho,N\text{-}Mes}$), 135.9 (s, $C_{ipso,C=N\text{-}Mes}$) 130.5 (s, *C*-$CH_{3,para,C=N\text{-}Mes}$), 128.9 (s, $CH_{N\text{-}Mes}$), 128.4 (s, *C*-$CH_{3,ortho,C=N\text{-}Mes}$), 127.8 (s, $CH_{C=N\text{-}Mes}$), 120.7 (s, *C*N), 70.4 ($CH_{2,crown}$), 69.0 (s, CO-$C^{(-)}$-CN), 68.1 (s,THF-OCH_2), 49.4 (s, *C*(CH_3)$_{2,DAC}$), 26.0 (s, THF-OCH$_2$CH_2), 25.8 (s, C(CH_3)$_{2,DAC}$), 21.2 (s, $CH_{3,para,N\text{-}Mes}$), 20.9 (s, $CH_{3,para,C=N\text{-}Mes}$), 18.9 (s, $CH_{3,ortho,C=N\text{-}Mes}$), 18.0 (s, $CH_{3,ortho,N\text{-}Mes}$). **FT-**

IR (ATR, cm^{-1}): 2908.9 (w), 2888.1 (w), 2862.3 (w), 2188.7 (w, CN stretching), 2138.4 (w, CN stretching), 1680.3 (m), 1617.1 (m), 1605.6 (m), 1560.4 (s), 1472.1 (w), 1434.7 (s), 1388.1 (m), 1349.3 (m), 1283.9 (w), 1248.0 (w), 1104.4 (s), 1011.1 (w), 962.9 (m), 886.8 (w), 850.9 (m), 707.3 (w), 569.4 (w), 504.8 (w), 468.9 (w). **Anal. Calcd** for $C_{43}H_{63}KN_3O_9$: C, 64.15; H, 7.89; N, 5.22. Found: C, 64.10; H, 7.33; N, 5.26.

1.8. Synthesis of compound 7

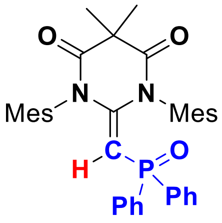

The toluene solution of 40.3 mg (0.107 mmol) **6-DAC** was added to the THF solution of 30 mg (0.107 mmol) **2^{PO}**, upon which substantial N_2 evolution was observed. The reaction mixture was stirred for 10 min, and whitish-yellow precipitation formed. The solvent was decanted and washed with 2 mL pentane (X2). The solid was dried in vacuo to get **7** as a off-whitish solid (47 mg, 0.0796 mmol, 74%) . Single crystals suitable for X-ray diffraction analysis were grown by slow vapor diffusion of hexane into a saturated solution of **7** in THF.

^{1}H NMR (400 MHz, THF-d$_8$): δ = 7.30 – 7.21 (m, 2H, C*H*$_{Ph,para}$), 7.17 – 7.11 (m, 8H, C*H*$_{Ph,ortho,meta}$), 6.99 (s, 2H, C*H*$_{Mes}$), 6.31 (s, 2H, C*H*$_{Mes}$), 4.66 (d, $^2J_{HP}$ = 4.1 Hz, 1H, C=C(*H*)-P, 2.30 (s, 3H, C*H*$_{3,para,N\text{-}Mes}$), 2.20 (s, 6H, C*H*$_{3,ortho,N\text{-}Mes}$), 2.08 (s, 3H, C*H*$_{3,para,N\text{-}Mes}$), 2.04 (s, 6H, C*H*$_{3,ortho,N\text{-}Mes}$), 1.62 (s, 6H, (C*H*$_3$)$_{2,DAC}$). **^{13}C{^{1}H} NMR** (101 MHz, THF-d$_8$): δ = 170.4 (s, *C*=O), 170.2 (s, *C*=O), 147.36 (d, $^2J_{CP}$ = 2.8 Hz, *C*=C(H)-P), 139.8 (s, *C*$_{ipso,N\text{-}Mes}$), 139.2 (s, *C*$_{ipso,N\text{-}Mes}$), 138.7 (s, *C*-CH$_{3,ortho,N\text{-}Mes}$), 138.7 ($^1J_{CP}$ = 109.6 Hz, *C*$_{Ph,ipso}$), 136.9 (s, *C*-CH$_{3,ortho,N\text{-}Mes}$), 134.6 (s, *C*-CH$_{3,para,N\text{-}Mes}$), 132.8 (s, *C*-CH$_{3,para,N\text{-}Mes}$), 131.1 (d, $^4J_{CP}$ = 2.8 Hz, *C*$_{Ph,para}$), 130.8 (d, $^2J_{CP}$ = 8.4 Hz, *C*$_{Ph,ortho}$), 130.7 (s, *C*H$_{N\text{-}Mes}$), 129.8 (s, *C*H$_{N\text{-}Mes}$), 128.5 (d, $^3J_{CP}$ = 11.7 Hz, *C*$_{Ph,meta}$), 85.0 (d, $^1J_{CP}$ = 114.4 Hz, C=*C*(H)-P), 48.7 (s, *C*(CH$_3$)$_{2,DAC}$), 24.6 (s, C(*C*H$_3$)$_{2,DAC}$), 21.3 (s, *C*H$_{3,para,N\text{-}Mes}$), 21.2 (s, *C*H$_{3,para,N\text{-}Mes}$), 19.4 (s, *C*H$_{3,ortho,N\text{-}Mes}$), 18.3 (s, *C*H$_{3,ortho,N\text{-}Mes}$). **^{31}P{^{1}H} NMR** (400 MHz, THF-d$_8$): δ = 10.32 (s, *P*Ph$_2$O) ppm. **FT-IR** (ATR, cm^{-1}): 2982.2 (w), 2918.3 (w), 2866.6 (w), 1724.1 (w), 1685.3 (m), 1560.4 (s), 1480.7 (w), 1436.9 (m), 1408.2 (w), 1388.8 (m), 1347.8 (m), 1288.9 (w), 1228.6 (w), 1186.3 (m), 1036.9 (w), 975.2 (w), 901.9 (w), 853.8 (m), 816.5 (w), 697.9 (s), 625.5 (w), 557.9 (m), 534.9 (s), 481.8 (w). **HRMS-LIFDI** (m/z): [M] $^{\bullet+}$ calcd for $C_{37}H_{39}N_2PO_3$, 590.2698; found, 590.2696.

1.9. Synthesis of compound 8

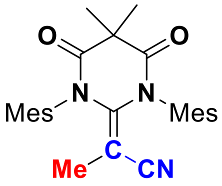

13.6 mg (0.019 mmol,) **4^{CN}** were dissolved in THF. 2.38 µL (0.038 mmol) of iodomethane were added. The solution was stirred for 2 min. The reaction mixture was filtered and the solvent and excess iodomethane were evaporated under reduced pressure. The resulting solid was dissolved in 1 mL of THF. Vapor diffusion of hexane to the THF solution resulted in the formation of colourless crystals of **8**, which were filtered off and dried in vacuo. Due to the co-crystallization of K(18-c-6)I with the product yield is not reported. Single-crystal X-ray diffraction analysis was performed on those crystals.

^{1}H NMR (400 MHz, THF-d$_8$): δ=6.95 (s, 2H, C*H*$_{Mes}$), 6.88 (s, 2H, C*H*$_{Mes}$), 2.29 (s, 3H, C*H*$_{3,para,N\text{-}Mes}$), 2.28 (s, 3H, C*H*$_{3,para,N\text{-}Mes}$), 2.21 (s, 6H, C*H*$_{3,ortho,N\text{-}Mes}$), 2.19 (s, 6H, C*H*$_{3,ortho,N\text{-}Mes}$), 1.62 (s, 6H, (C*H*$_3$)$_{2,DAC}$), 1.17 (s, 3H, C=C(C*H*$_3$)-CN) ppm. **^{13}C{^{1}H} NMR** (101 MHz, THF-d$_8$): δ =170.8 (s, *C*=O), 169.6 (s, *C*=O), 143.4 (s, *C*N), 140.9 (s, *C*$_{Ph,para}$), 139.7 (s, *C*$_{Ph,para}$), 138.6 (s, *C*$_{Ph,ortho}$), 137.5 (s, *C*$_{Ph,ortho}$), 135.7 (s, *C*$_{ipso,N\text{-}Mes}$), 134.2 (s, *C*$_{ipso,N\text{-}Mes}$), 130.3 (s, *C*$_{Ph,meta}$), 130.3 (s,

$C_{Ph,meta}$), 118.1 (s, C=C(CH$_3$)-CN), 75.2 (s, C=C(CH$_3$)-CN), 48.9 (s, C(CH$_3$)$_{2,DAC}$), 24.1 (s, C(CH$_3$)$_{2,DAC}$), 21.4 (s, $CH_{3,para,N\text{-}Mes}$), 21.1 (s, $CH_{3,para,N\text{-}Mes}$), 20.1 (s, C=C(CH$_3$)-CN), 19.0 (s, $CH_{3,ortho,N\text{-}Mes}$), 18.9 (s, $CH_{3,ortho,N\text{-}Mes}$) ppm. **FT-IR** (ATR, cm^{-1}): 2982.1 (w), 2868.6 (w), 2182.2 (m, CN stretching), 1716.2 (s), 1685.3 (s), 1583.3 (s), 1482.8 (w), 1461.2 (m), 1444.7 (m), 1389.4 (m), 1342.8 (s),1218.5 (m), 1193.4 (m), 1158.2 (w), 1114.4 (w), 1102.2 (m), 1011.7 (w), 973.7 (w), 853.8 (s), 807.8 (m), 747.5 (w), 664.2 (w), 577.3 (m), 522.7 (m), 469.6 (m). **HRMS-LIFDI** (m/z): [M] $^{\bullet+}$ calcd for $C_{27}H_{31}N_3O_2$, 429.2416; found, 429.2443

1.10. Synthesis of compound 9

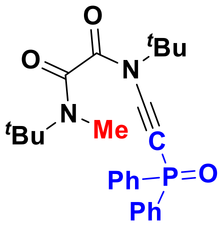

The THF solution of 22.5 mg (0.107 mmol) 5-DAC was added to the THF solution of 30 mg (0.107 mmol) **2^{PO}**, upon which substantial N_2 evolution was observed. After stirring for 10 min, 10 µL (0.161 mmol) iodomethane was added. The resulting solution was stirred overnight. The next day, the solution was filtered and concentrated. Pentane was added over the concentrated solution, and the whole mixture was kept overnight. Precipitation was obtained, which was washed with pentane twice and dried in *vacuo* to get **9** as an off-white solid. (39 mg, 0.089 mmol, 83 %). Single crystals suitable for X-ray diffraction analysis were grown by slow vapor diffusion of pentane into a saturated solution of **9** in THF.

^{1}H NMR (400 MHz, THF-d$_8$): δ = 7.87 – 7.77 (m, 4H, $CH_{Ph,ortho}$), 7.52 – 7.42 (m, 6H, $CH_{Ph,meta,para}$), 2.86 (s, 3H, N(CH$_3$)-C(CH$_3$)$_3$), 1.50 (s, 9H, N-C(CH_3)$_3$), 1.25 (s, 9H, N(CH$_3$)-C(CH_3)$_3$). **^{13}C{^{1}H} NMR** (101 MHz, THF-d$_8$): δ = 167.7 (s, O=C-NC(CH$_3$)$_3$), 164.3 (s, O=C-N(CH$_3$)-C(CH$_3$)$_3$), 136.0 (d, $^1J_{CP}$ = 121.1 Hz, $C_{Ph,ipso}$), 132.7 (d, $^4J_{CP}$ = 2.9 Hz, $C_{Ph,para}$), 131.9 (d, $^2J_{CP}$ = 10.7 Hz, $C_{Ph,ortho}$), 129.4 (d, $^3J_{CP}$ = 13.2 Hz, $C_{Ph,meta}$), 97.1 (d, $^2J_{CP}$ = 34.3 Hz, P-CCN), 70.6 (d, $^1J_{CP}$ = 71.1 Hz, P-CCN), 62.3 (s, N-C(CH$_3$)$_3$), 57.9 (s, N(CH$_3$)-C(CH$_3$)$_3$), 32.4 (s, N(CH$_3$)-C(CH$_3$)$_3$), 28.1, (s, N-C(CH$_3$)$_3$), 27.7 (s, N(CH$_3$)-C(CH$_3$)$_3$). **^{31}P{^{1}H} NMR** (400 MHz, THF-d$_8$): δ = 3.76 (s, PPh$_2$O) ppm. **FT-IR** (ATR, cm^{-1}): 2975.7 (w), 2181.5 (m, C-C triple bond), 1691.1 (s), 1653.7 (s), 1589.8 (w), 1472.1 (w), 1438.3 (m), 1394.5 (m), 1367.2 (m), 1314.8 (m), 1268.8 (w), 1197.0 (s), 1122.4 (m), 1097.2 (s), 1025.4 (w), 997.4 (w), 848.8 (w), 811.4 (m), 725.9 (m), 559.4 (m), 534.3 (s), 451.6 (m). **HRMS-LIFDI** (m/z): [M] $^{\bullet+}$ calcd for $C_{37}H_{39}N_2PO_3$, 438.2072; found, 438.3203.

1.11. Synthesis of compound 10

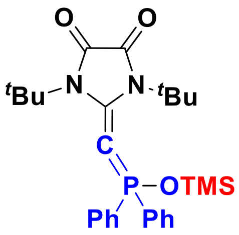

30 mg (0.0413 mmol) **3^{PO}** was dissolved in 1 mL THF in a J-Young tube. 8 µL (0.062 mmol) TMSCl was added to the THF solution. ^{31}P NMR spectroscopy showed a new peak at –6.6 ppm. The solvent was evaporated under reduced pressure, and the resulting solid was extracted with pentane. Yellow crystals were formed from the pentane solution, which were filtered off and dried in *vacuo*. Single-crystal XRD analysis was performed on the crystals. Most of the time, due to the co-crystallization of K(18-c-6)Cl with the product, the yield is not reported.

^{1}H NMR (400 MHz, THF-d$_8$): δ = 7.73 – 7.62 (m, 4H, $CH_{Ph,ortho}$), 7.54 – 7.47 (m, 6H, $CH_{Ph,meta,para}$), 1.58 (s, 18H, NC(CH_3)$_3$), 0.01 (s, 9H, Si(CH_3)$_3$) ppm. **^{13}C{^{1}H} NMR** (101 MHz,

THF-d_8): δ = 158.7 (s, *C*=O), 135.0 (d, $^1J_{CP}$ = 259.66 Hz, C=*C*=P), 132.9 (d, $^1J_{CP}$ =153.02 Hz, $C_{Ph,ipso}$), 132.4 (d, $^2J_{CP}$ = 11.1 Hz, $C_{Ph,ortho}$), 132.1 (d, $^4J_{CP}$ = 3.2 Hz, $C_{Ph,para}$), 129.6 (d, $^3J_{CP}$ = 14.6 Hz, $C_{Ph,meta}$), 126.2 (d, *J* = 39.6 Hz, *C*=C=P), 58.4 (s, N-*C*(CH_3)$_3$), 30.1 (s, N-C(CH_3)$_3$), 1.4 (s, Si-O-(CH_3)$_3$) ppm. **^{29}Si{^{1}H} NMR** shifts were extracted from the indirect dimension of a ^{1}H, ^{29}Si HMBC experiment: 15.15 ppm. **^{31}P{^{1}H} NMR** (400 MHz, THF-d_8): δ = –6.65 (s, *P*Ph_2O) ppm. **FT-IR** (ATR, cm^{-1}): 3076.1 (w), 2979.9 (w), 2927.5 (w), 1747.7 (s), 1728.4 (s), 1696.8 (w), 1599.8 (m), 1572.5 (w), 1482.1 (w), 1434.7 (m), 1396.6 (w), 1362.9 (s), 1308.3 (w), 1247.9 (w), 1171.2 (m), 1117.3 (m), 1021.8 (w), 995.2 (w), 936.3 (w), 837.3 (m), 778.4 (m), 693.6 (s), 624.7 (w), 533.5 (s), 465.3 (w). **Anal. Calcd** for $C_{27}H_{37}N_2O_3P_1Si_1$: C, 65.29; H, 7.51; N, 5.64. Found: C, 65.69; H, 7.22; N, 5.70.

1.12. Synthesis of compound 11

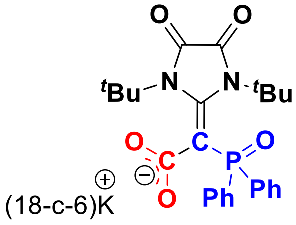

30 mg (0.0413 mmol) **3^{PO}** was dissolved in 1 mL benzene/THF (5:1) in a J-Young NMR tube. The atmosphere of the tube was changed to CO_2, upon which the insoluble solid dissolved, resulting in a color change from orange to colorless. The solvent was concentrated, and pentane was added, resulting in a white solid. The solid was filtered and washed with pentane. After drying in *vacuo*, **11** was obtained as a white solid (24 mg, 0.0311 mmol, 75%). ^{1}H NMR spectrum of **11** in THF-d_8 shows two isomers, which arise due to different modes of potassium cation coordination. Variable-temperature (VT) NMR spectroscopy studies confirm a reversible interconversion between these two isomers. After adding 1 eq. of [2.2.2]-cryptand to the THF-d_8 solution of **11**, only a single isomer is formed due to the encapsulation of the potassium cation.

^{1}H NMR (400 MHz, THF-d_8): δ = 7.96 – 7.89, 7.82 – 7.75 (m, 0.8H + 3.2H, $CH_{Ph,ortho}$), 7.36 – 7.16 (m, 6H, $CH_{Ph,meta,para}$), 3.51 (s, 24H, $CH_{2,crown}$), 1.70, 1.32 (s, 7H + 2H, NC(CH_3)$_3$), 1.42, 1.14 (s, 7H+2H, NC(CH_3)$_3$) ppm. **^{13}C{^{1}H} NMR** (101 MHz, THF-d_8): δ = 179.2 (s, *C*=O), 179.1 (s, *C*=O), 168.8 (d, $^2J_{CP}$=14.1 Hz, *C*O_2), 150.8 (d, $^2J_{CP}$ = 13.6 Hz, *C*=C-P), 141.4 (d, $^1J_{CP}$ =106.8 Hz, $C_{Ph,ipso}$), 132.9 (d, $^2J_{CP}$ = 10.2 Hz, $C_{Ph,ortho}$), 129.8 (d, $^4J_{CP}$ = 2.8 Hz, $C_{Ph,para}$), 127.7 (d, $^3J_{CP}$ = 12.0 Hz, $C_{Ph,meta}$), 83.9 (d, $^1J_{CP}$ = 128.9 Hz, C=*C*-P), 71.1 (s, $CH_{2,crown}$), 57.4 (s, N-*C*(CH_3)$_3$), 55.3 (s, N-*C*(CH_3)$_3$), 32.0 (s, N-C(CH_3)$_3$), 31.4 (s, N-C(CH_3)$_3$) ppm. **^{31}P{^{1}H} NMR** (400 MHz, THF-d_8): δ = 23.27, 22.59 (s, *P*Ph_2O) ppm. **FT-IR** (ATR, cm^{-1}): 2960.5 (w), 2888.0 (w), 1667.3 (w), 1619.2 (s), 1597.7 (m), 1438.3 (w), 1352.8 (m), 1342.8 (m), 1238.6 (w), 1206.3 (w), 1109.4 (s), 1098.6 (s), 1029.7 (w), 962.2 (m), 954.3 (m), 889.7 (w), 835.1 (w), 758.9 (w), 722.4 (m), 702.9 (m), 534.9 (s). **Anal. Calcd** for $C_{37}H_{52}KN_2O_{11}P$: C, 57.65; H, 6.80; N, 3.63. Found: C, 58.72; H, 6.66; N, 4.04.

Addition of 1 eq. [2.2.2]-cryptand:

^{1}H NMR (400 MHz, THF-d_8): δ = 7.85 – 7.75 (m, 4H, $CH_{Ph,ortho}$), 7.20 – 7.14 (m, 6H, $CH_{Ph,meta,para}$), 3.57 (s, $CH_{2,crown}$ and $CH_{2,cryptand}$), 3.52 (t, $^3J_{HH}$ = 4.8 Hz, $CH_{2,cryptand}$), 2.56 (t, $^3J_{HH}$ = 4.8 Hz, $CH_{2,cryptand}$), 1.68 (s, 9H, NC(CH_3)$_3$), 1.36 (s, 9H, NC(CH_3)$_3$). **^{13}C{^{1}H} NMR** (101 MHz, THF-d_8): δ = 179.1 (s, *C*=O), 179.0 (s, *C*=O), 167.5 (d, $^2J_{CP}$ =14.6 Hz, *C*O_2), 151.8 (d, $^2J_{CP}$ = 14.1 Hz, *C*=C-P), 142.0 (d, $^1J_{CP}$ =105.9 Hz, $C_{Ph,ipso}$), 133.1 (d, $^2J_{CP}$ = 9.9 Hz, $C_{Ph,ortho}$), 129.4 (d, $^4J_{CP}$ = 2.8 Hz, $C_{Ph,para}$), 127.4 (d, $^3J_{CP}$ = 11.9 Hz, $C_{Ph,meta}$), 82.5 (d, $^1J_{CP}$ = 132.2 Hz, C=*C*-P), 71.7 (s, $CH_{2,crown}$), 71.5 (s, $CH_{2,cryptand}$), 68.9 (s, $CH_{2,cryptand}$), 55.8 (s, N-*C*(CH_3)$_3$), 55.5 (s,

$CH_{2,cryptand}$), 54.9 (s, N-C(CH_3)$_3$), 32.2 (s, N-C(CH_3)$_3$), 31.4 (s, N-C(CH_3)$_3$) ppm. **^{31}P{^{1}H} NMR** (400 MHz, THF-d_8): δ = 22.63 (s, PPh$_2$O) ppm.

1.13. Synthesis of compound 12

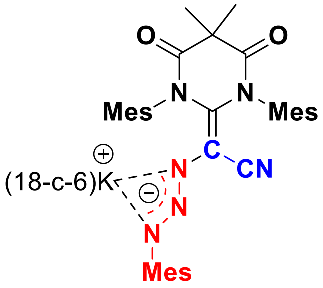

30 mg (0.0418 mmol) **4^{CN}** was dissolved in 1 mL of cold benzene in a vial. 10.1 mg (0.0627 mmol) mesityl azide was added and stirred for 30 min, resulting in yellow precipitation. The solid was filtered and washed with 1 mL of benzene (twice) and 3 mL of pentane (three times). After drying in *vacuo*, **12** was obtained as a yellow solid (29 mg, 0.033 mmol, 80%).

^{1}H NMR (400 MHz, THF-d_8): δ = 6.83 (s, 0.71H, CH_{Mes}), 6.81 (s, 1.24H, CH_{Mes}), 6.64 (s, 1.24H, CH_{Mes}), 6.59 (s, 0.69H, CH_{Mes}), 6.49 (s, 1.22H, CH_{Mes}), 6.30 (s, 0.66H, CH_{Mes}), 2.28 + 2.27 + 2.26 + 2.25 (s, 9.76H, CH_3), 2.21 (s, 3.89H, CH_3), 2.17 (s, 1.1H, CH_3), 2.16 (s, 1.8H, CH_3), 2.09 (s, 1.92H, CH_3), 2.06 (s, 3.73H, CH_3), 1.75 (s, 2.07H, CH_3), 1.69 (s, 1.98H, CH_3), 1.55 (s, 3.79H, CH_3), 1.46 (s, 2H, CH_3). The **^{13}C{^{1}H} NMR** spectrum showed many peaks due to the presence of two isomers, which were very difficult to assign. **FT-IR** (ATR, cm^{-1}): 2981.4 (m), 2905.9 (w), 2870.1 (w), 2188.6 (w, CN stretching), 1691.7 (m), 1657.3 (m), 1609.2, (w), 1554.6 (w), 1472.7 (w), 1388.0 (m), 1336.3 (s), 1240.8 (s), 1182.6 (s), 1144.6 (m), 1107.9 (s), 950.7 (m), 808.5 (w), 712.3 (w), 606.0 (w), 558.6 (w), 519.1 (m), 500.5 (w), 448.1 (w). Attempts to obtain satisfactory elemental analysis data were repeatedly unsuccessful.

2. NMR and IR spectra

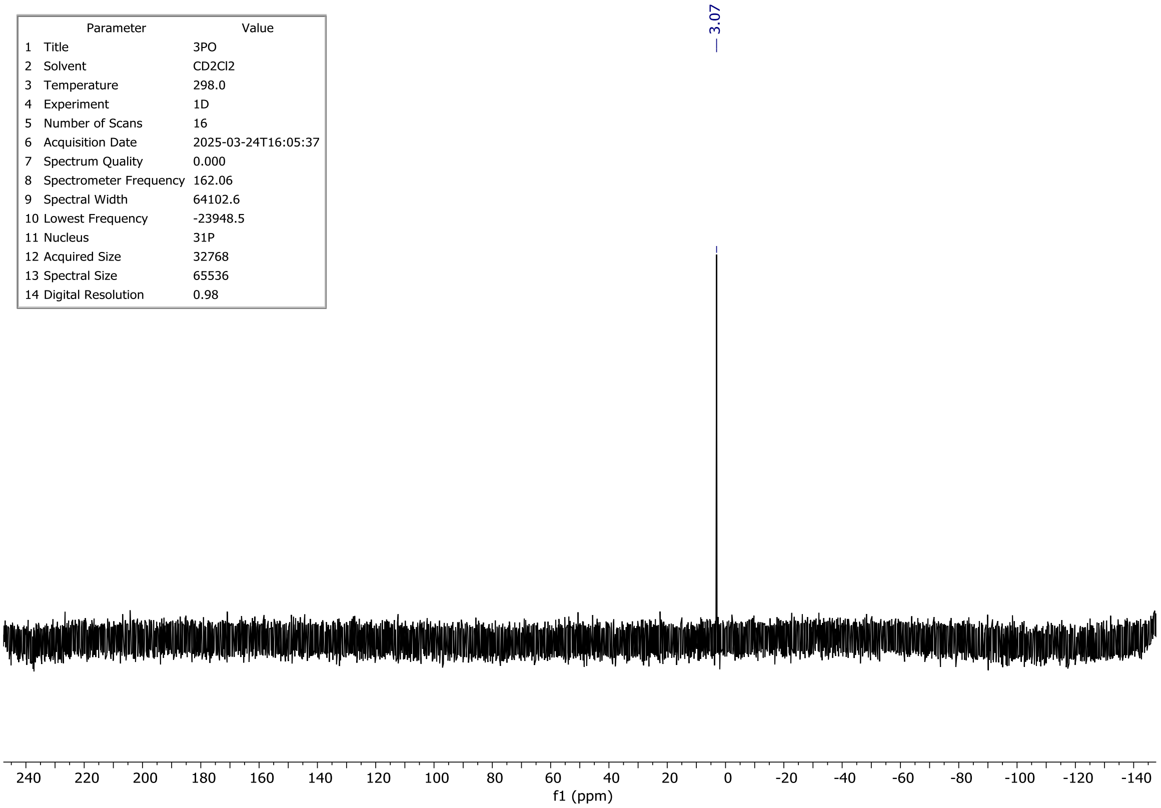

Figure S5 $^{31}P\{^{1}H\}$ NMR spectrum of compound $\mathbf{3^{PO}}$ in CD_2Cl_2.

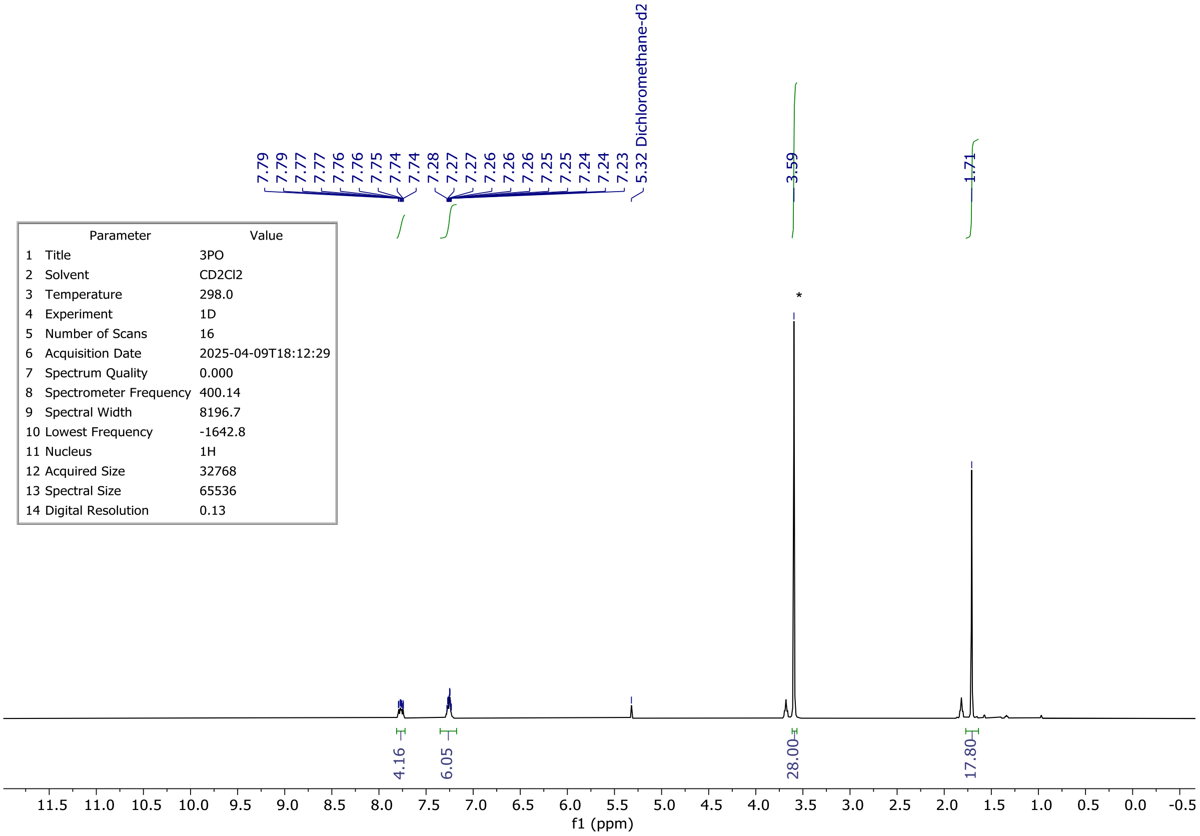

Figure S6 ^{1}H NMR spectrum of compound $\mathbf{3^{PO}}$ in CD_2Cl_2. (* = residual THF + 18-c-6).

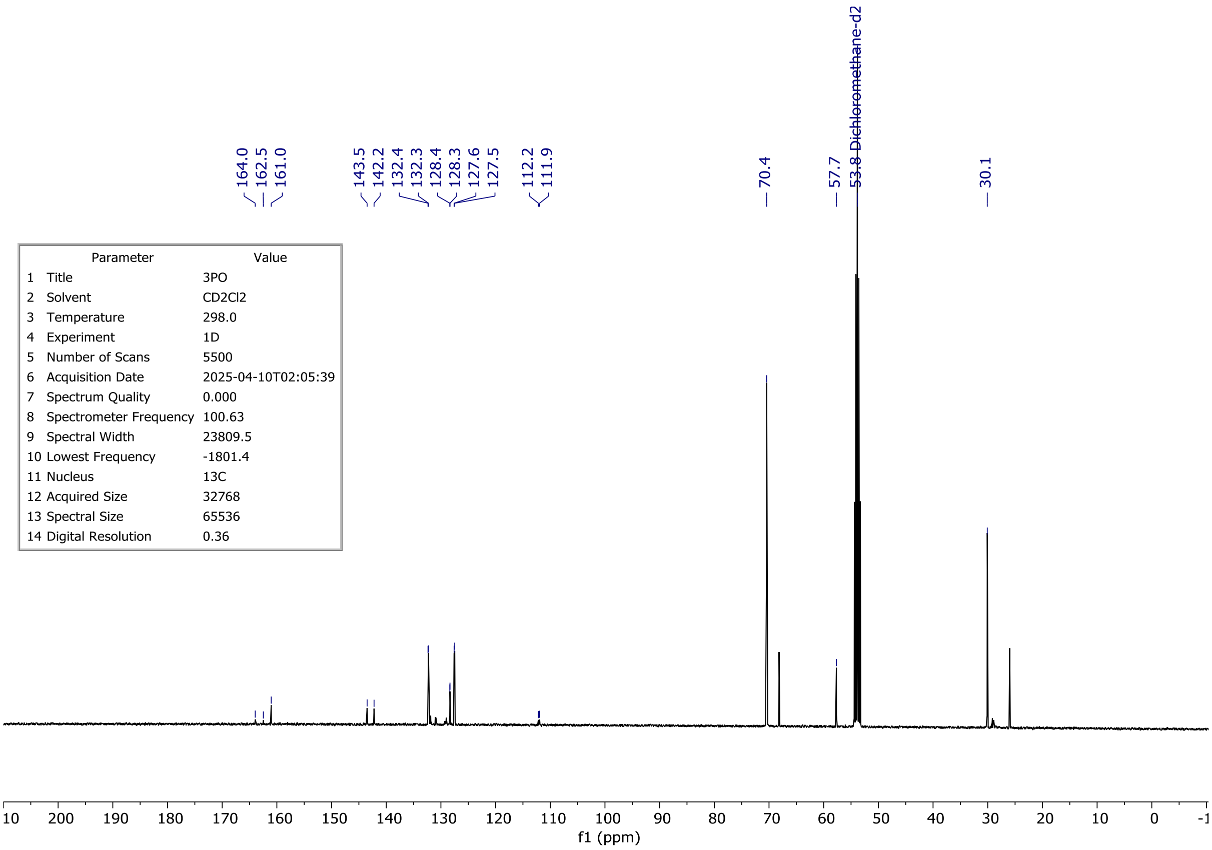

Figure S7 $^{13}C\{^{1}H\}$ NMR spectrum of compound **3^{PO}** in CD_2Cl_2.

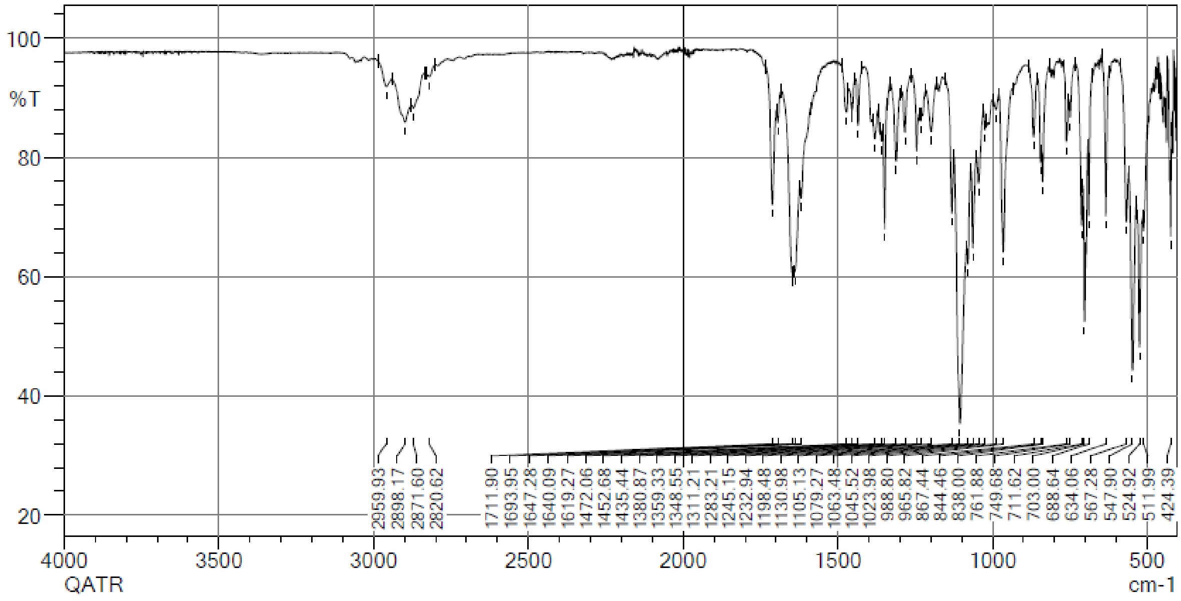

Figure S8 IR spectrum of compound **3^{PO}** (solid state).

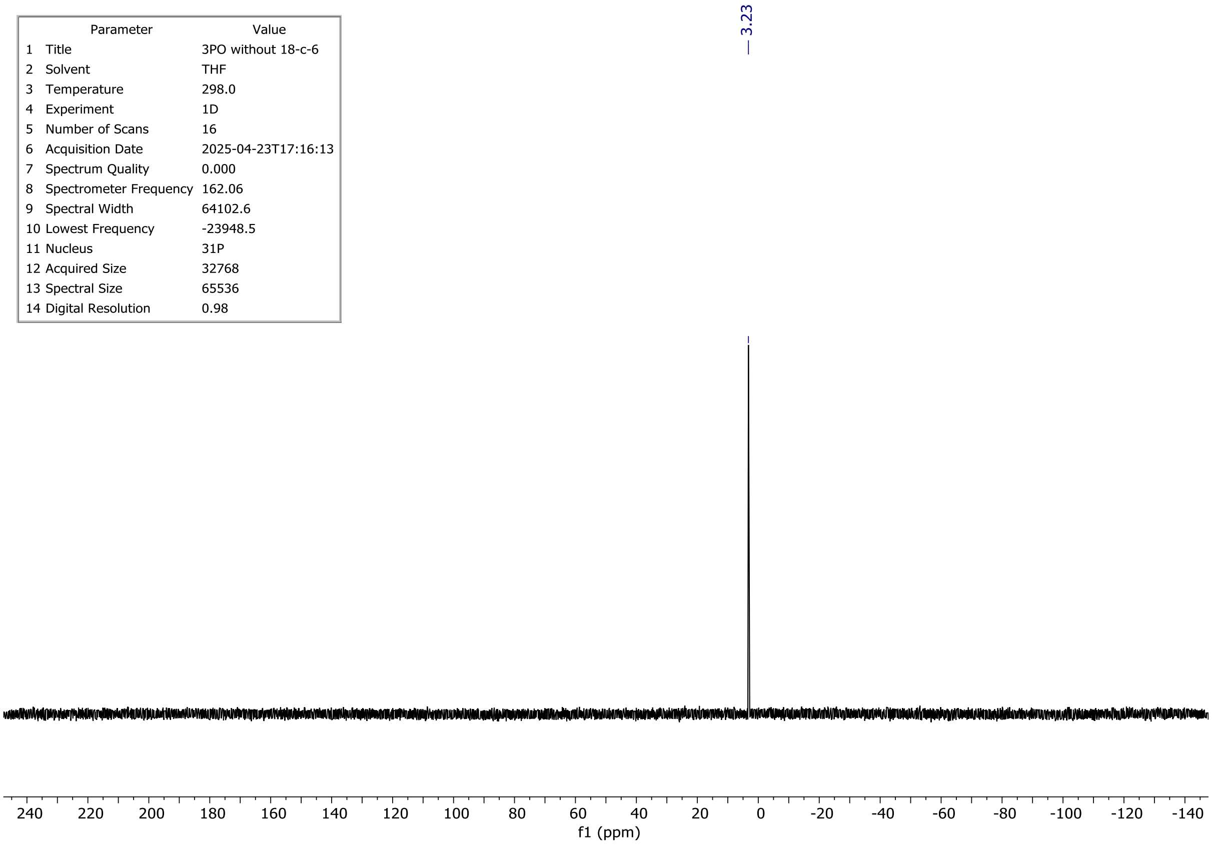

Figure S9 $^{31}P\{^{1}H\}$ NMR spectrum from the reaction mixture of $\mathbf{2^{PO}}$ and **5-DAC** ($\mathbf{3^{PO}}$ without 18-c-6) in THF-d_8.

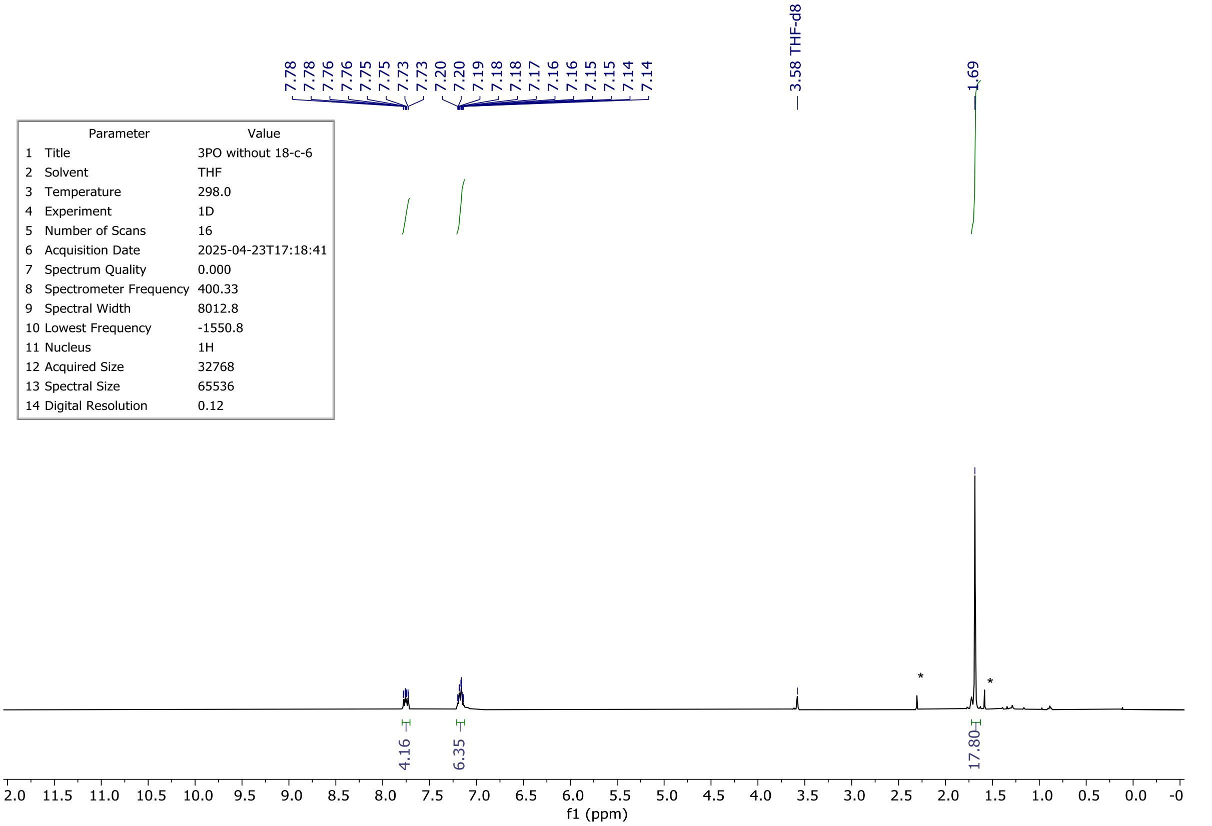

Figure S10 ^{1}H NMR spectrum from the reaction mixture of $\mathbf{2^{PO}}$ and **5-DAC** ($\mathbf{3^{PO}}$ without 18-c-6) in THF-d_8. * corresponds to the unknown impurities.

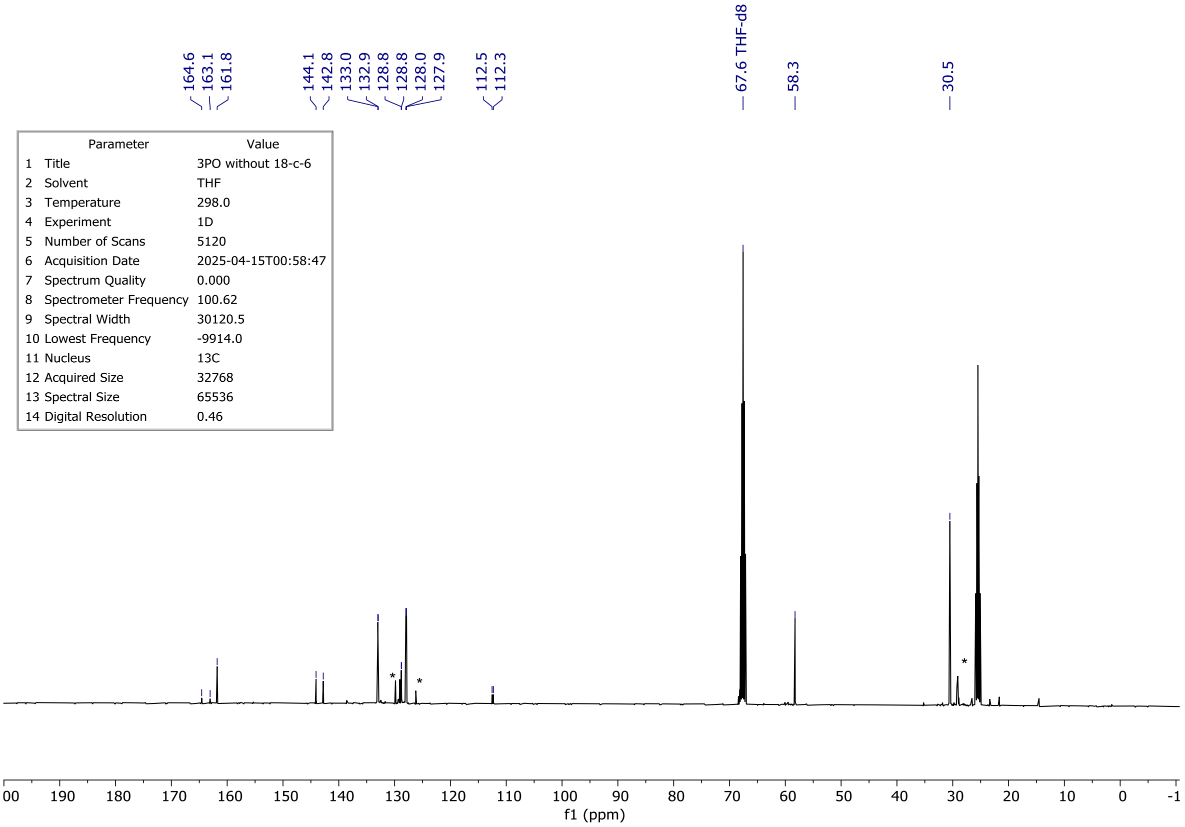

	Parameter	Value
1	Title	3PO without 18-c-6
2	Solvent	THF
3	Temperature	298.0
4	Experiment	1D
5	Number of Scans	5120
6	Acquisition Date	2025-04-15T00:58:47
7	Spectrum Quality	0.000
8	Spectrometer Frequency	100.62
9	Spectral Width	30120.5
10	Lowest Frequency	-9914.0
11	Nucleus	13C
12	Acquired Size	32768
13	Spectral Size	65536
14	Digital Resolution	0.46

Figure S11 $^{13}C\{^{1}H\}$ NMR spectrum from the reaction mixture of **2^{PO}** and **5-DAC** (**3^{PO}** without 18-c-6) in THF-d_8. * corresponds to the unknown impurities.

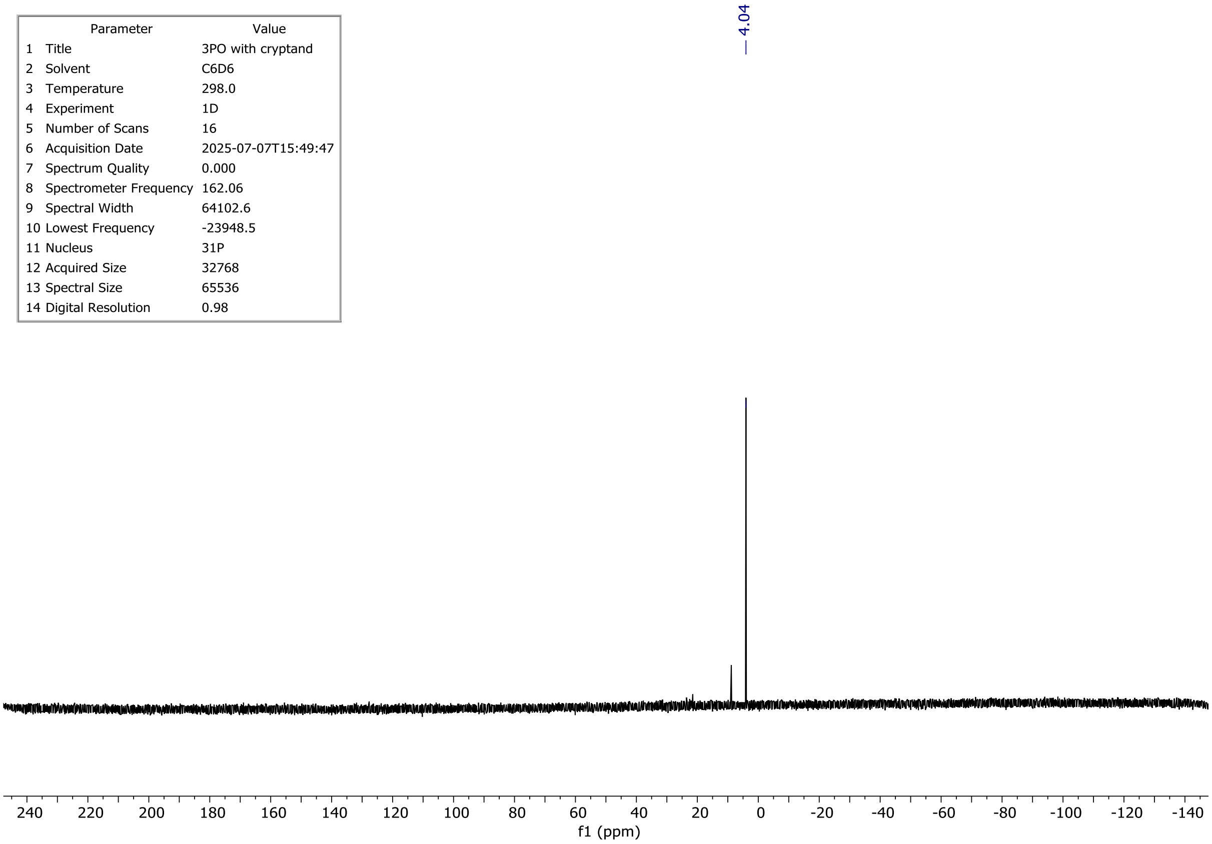

	Parameter	Value
1	Title	3PO with cryptand
2	Solvent	C6D6
3	Temperature	298.0
4	Experiment	1D
5	Number of Scans	16
6	Acquisition Date	2025-07-07T15:49:47
7	Spectrum Quality	0.000
8	Spectrometer Frequency	162.06
9	Spectral Width	64102.6
10	Lowest Frequency	-23948.5
11	Nucleus	31P
12	Acquired Size	32768
13	Spectral Size	65536
14	Digital Resolution	0.98

Figure S12 $^{31}P\{^{1}H\}$ NMR spectrum from the reaction mixture of **2^{PO}** and **5-DAC** in presence of [2.2.2]-cryptand (**3^{PO}** with [2.2.2]-cryptand) in THF-d_8.

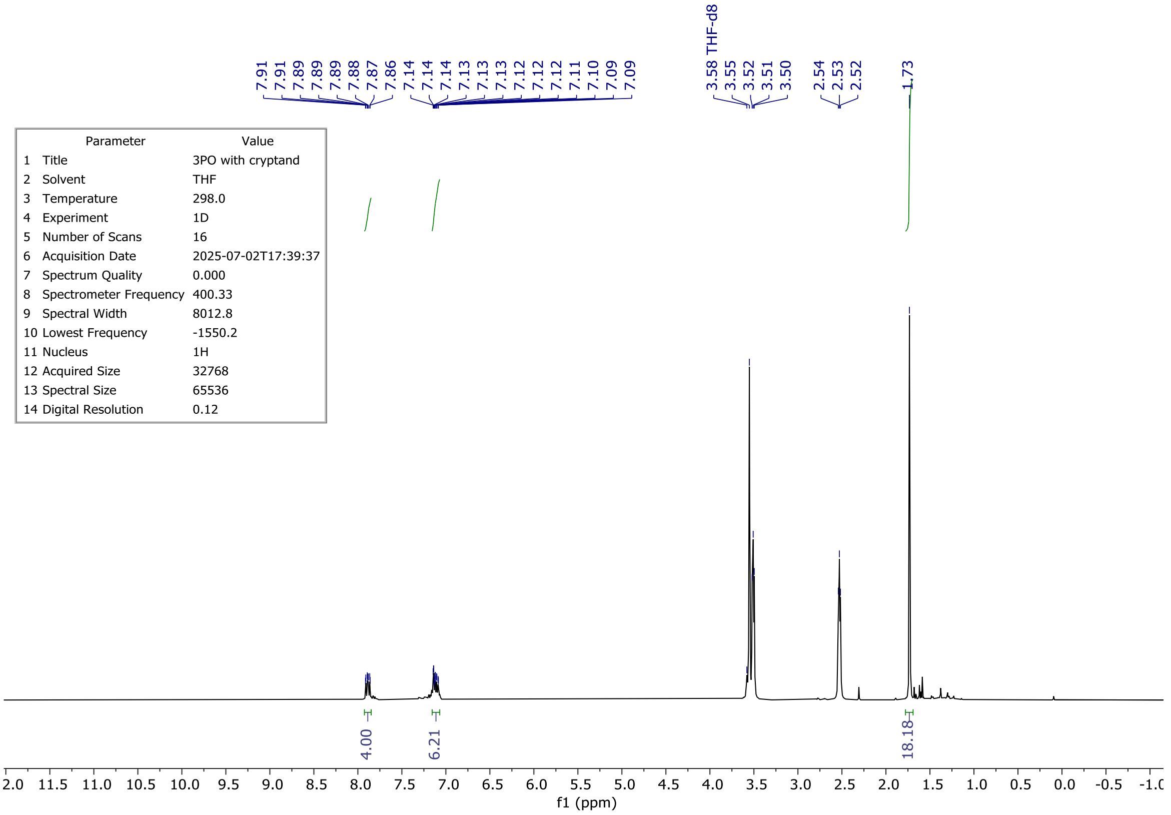

Figure S13 ^{1}H NMR spectrum from the reaction mixture of **2^{PO}** and **5-DAC** in presence of [2.2.2]-cryptand (**3^{PO}** with [2.2.2]-cryptand) in THF-d_8.

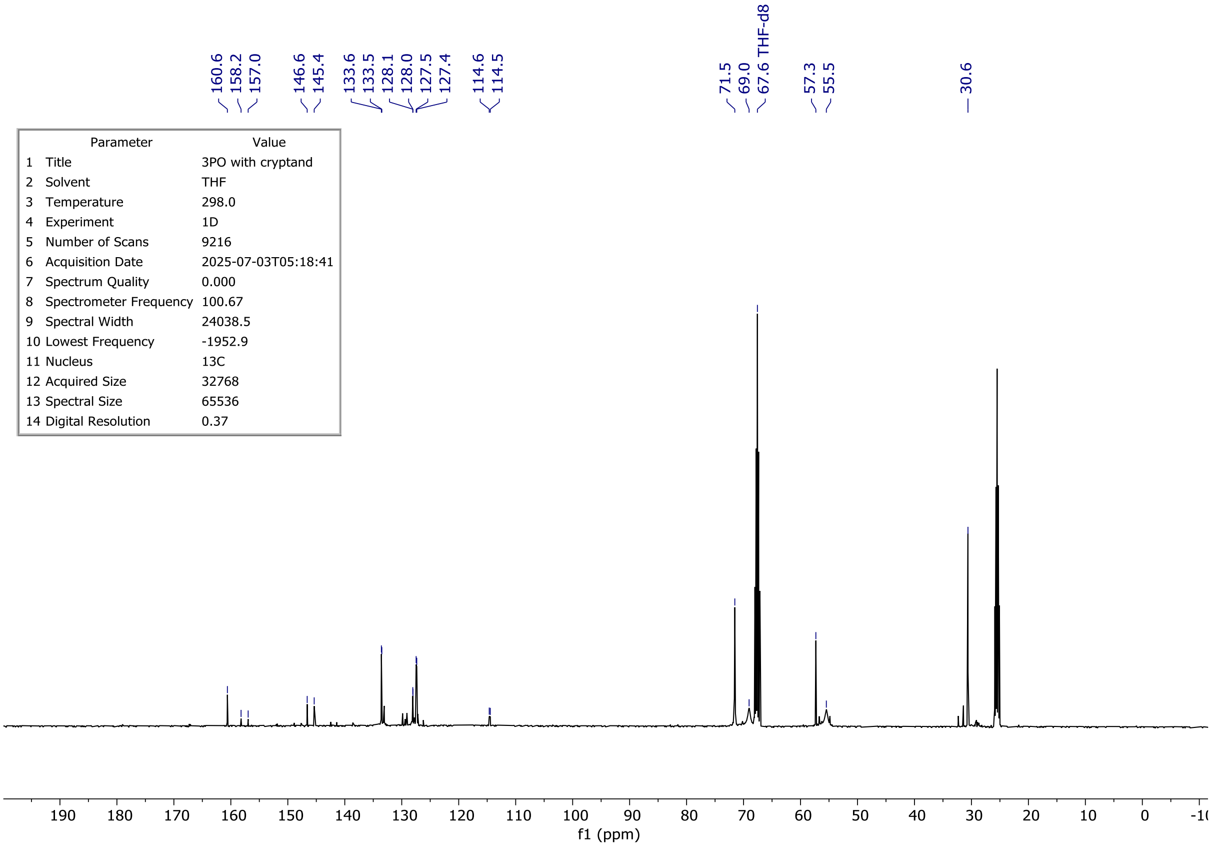

Figure S14 ^{13}C{^{1}H} NMR spectrum from the reaction mixture of **2^{PO}** and **5-DAC** in presence of [2.2.2]-cryptand (**3^{PO}** with [2.2.2]-cryptand) in THF-d_8.

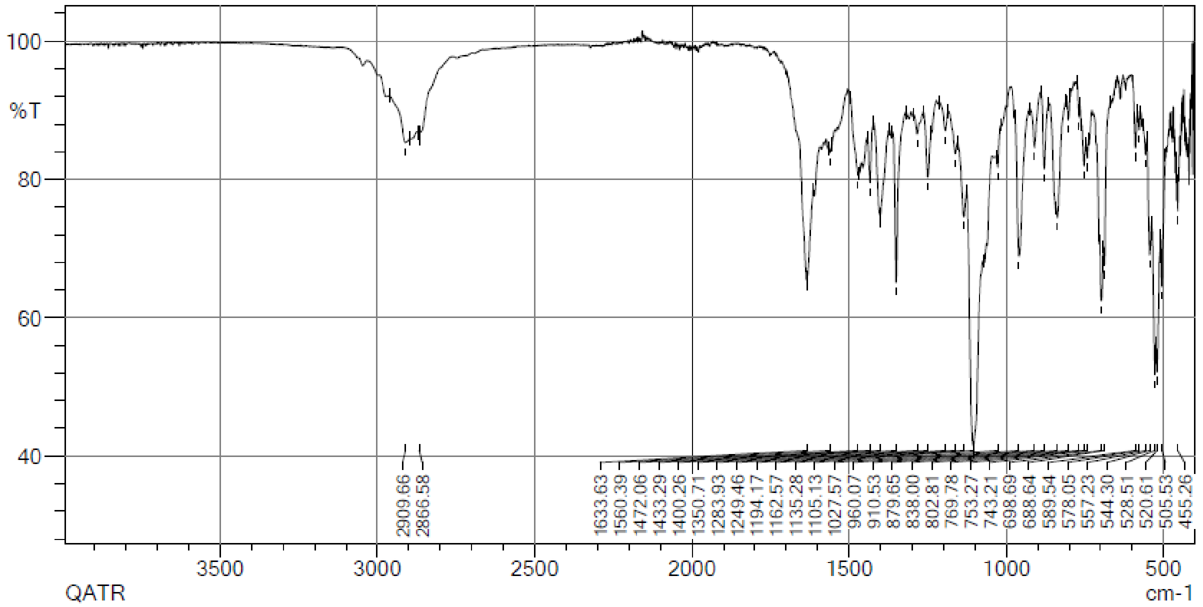

Figure S15 IR spectrum of compound $\mathbf{4^{PO}}$ (solid state).

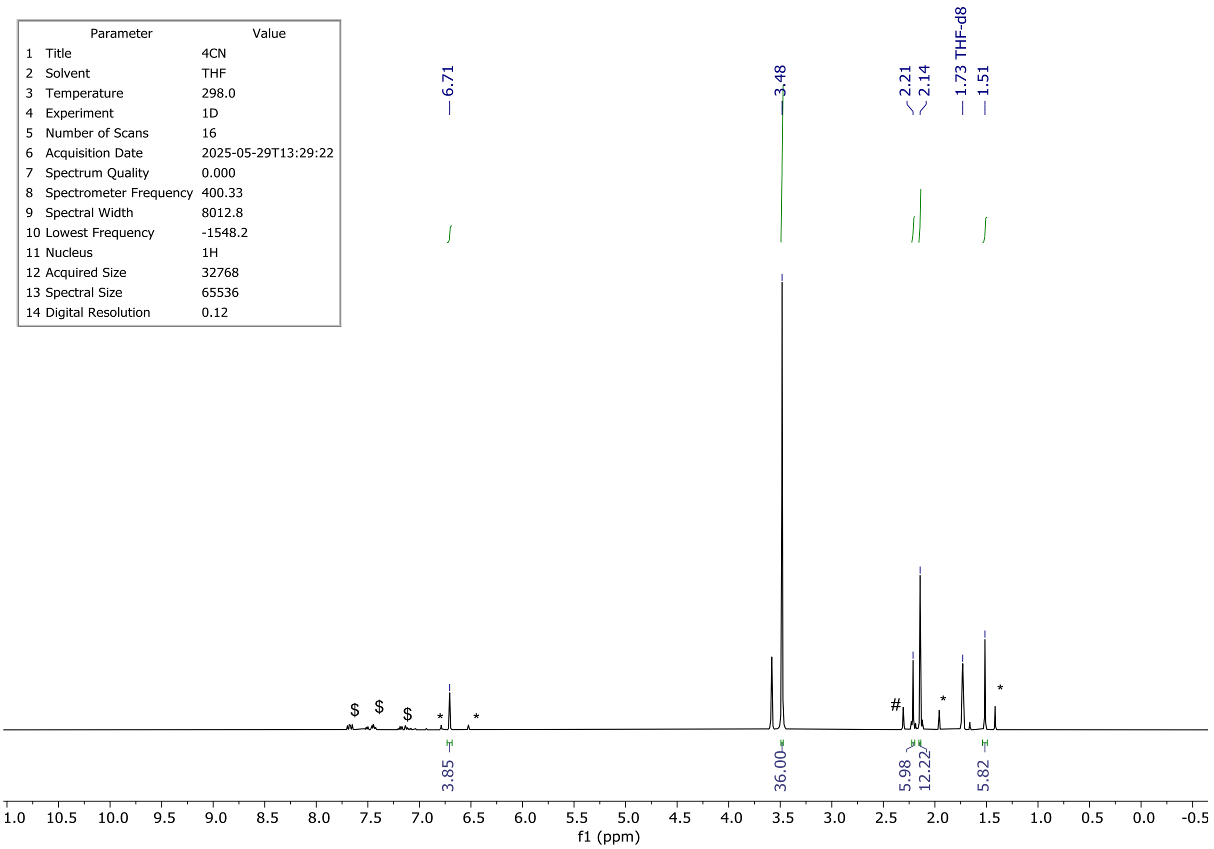

Figure S16 ^{1}H NMR spectrum of compound $\mathbf{4^{CN}}$ in THF-d_8. * corresponds to $\mathbf{6^{CN}}$, $ corresponds to P(O)Ph$_3$. # corresponds toluene.

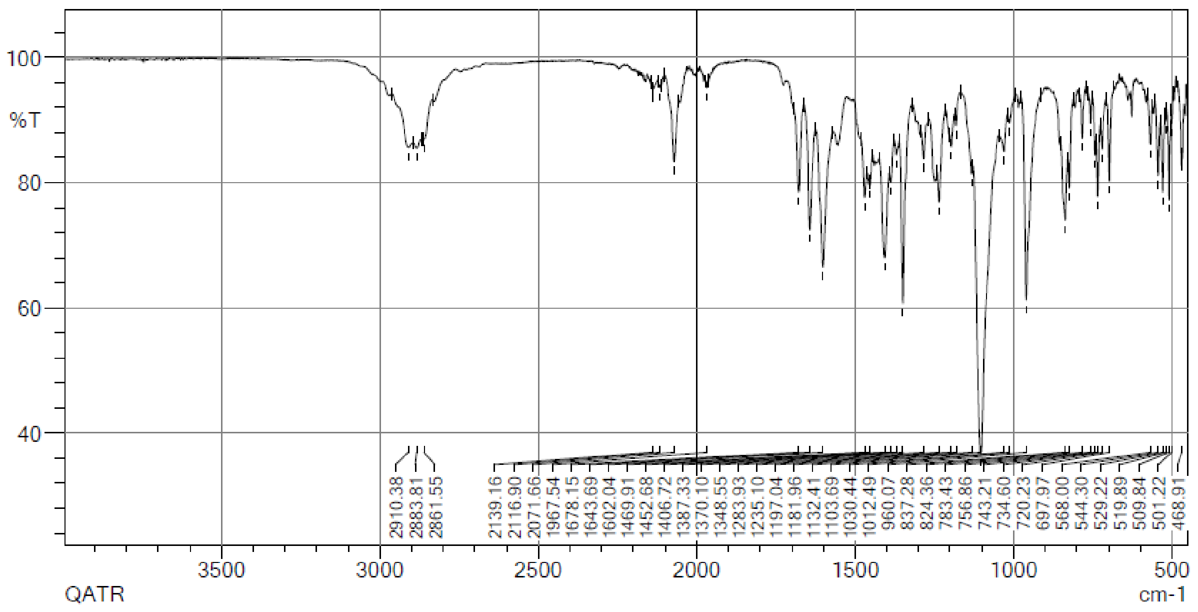

Figure S17 IR spectrum of compound $\mathbf{4^{CN}}$ (solid state).

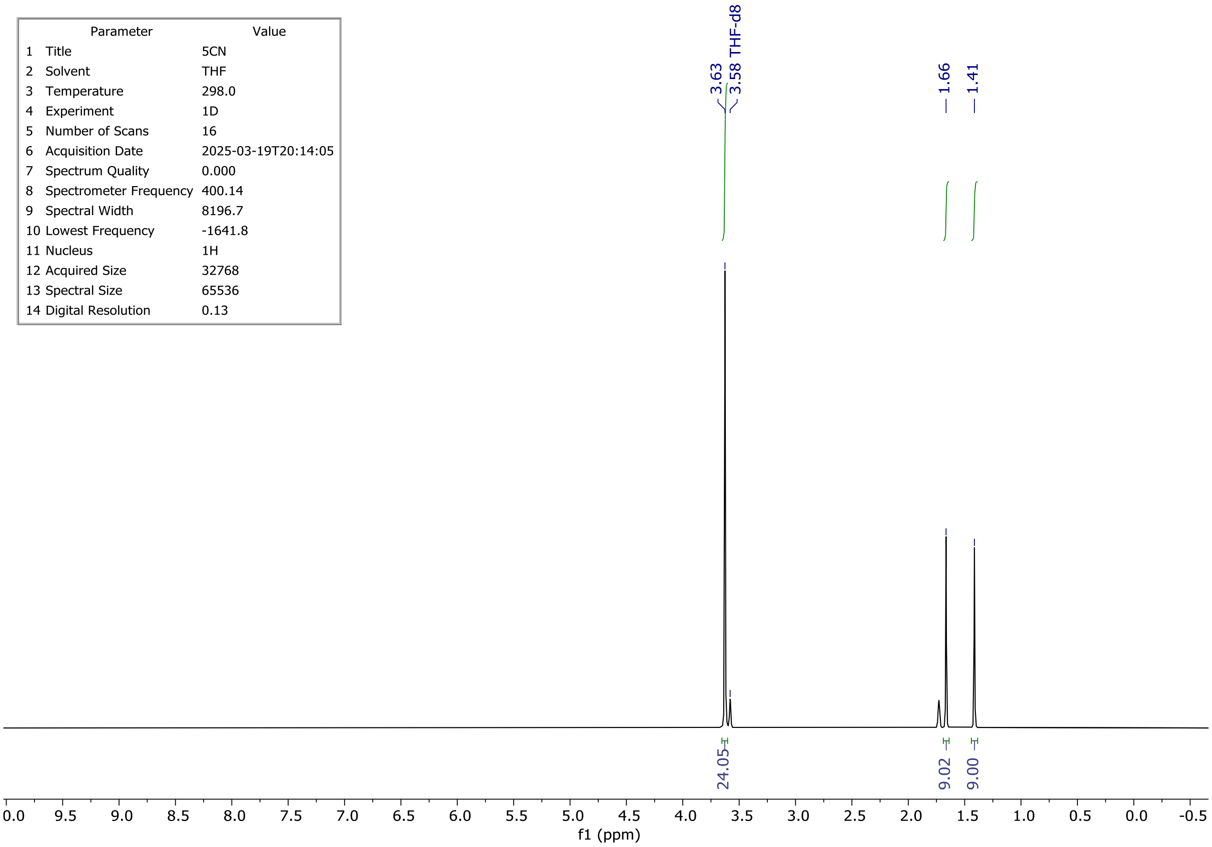

Figure S18 ^{1}H NMR spectrum of compound $\mathbf{5^{CN}}$ in THF-d$_8$.

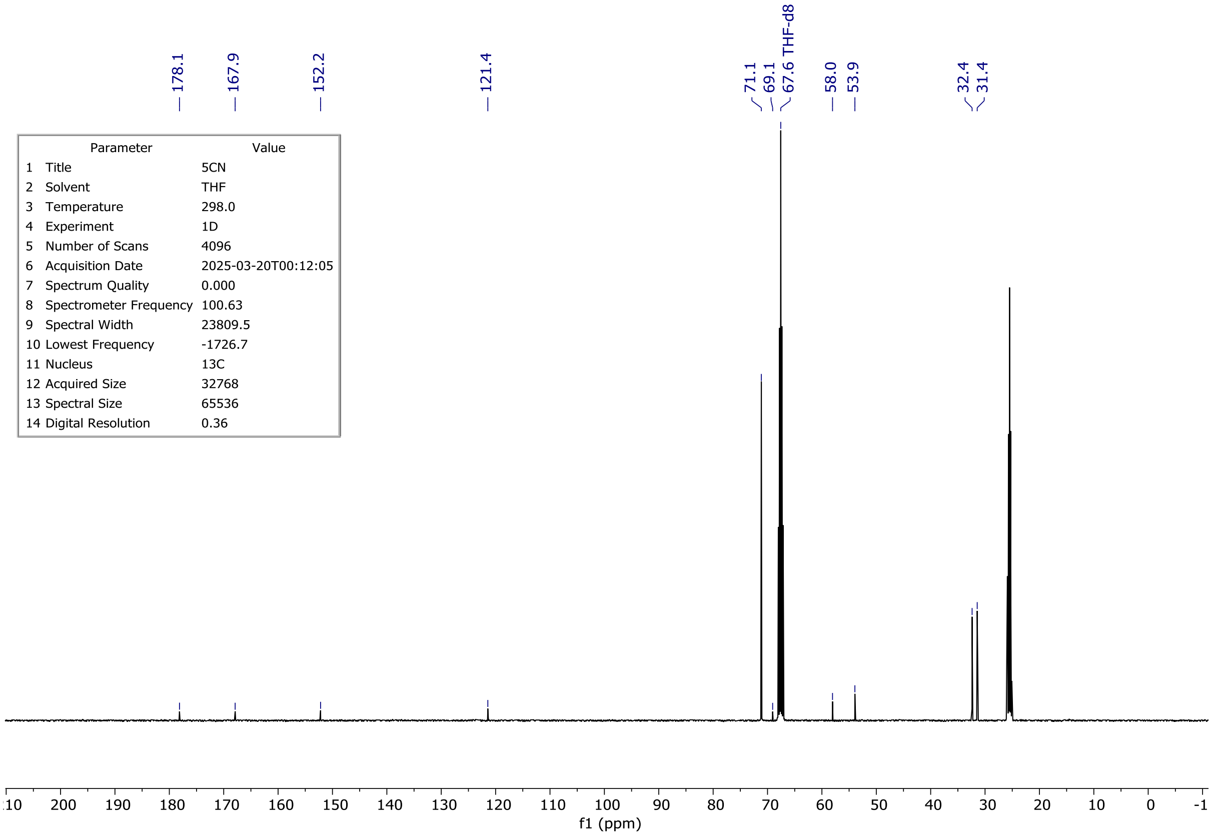

Figure S19 $^{13}C\{^{1}H\}$ NMR spectrum of compound **5^CN^** in THF-d_8.

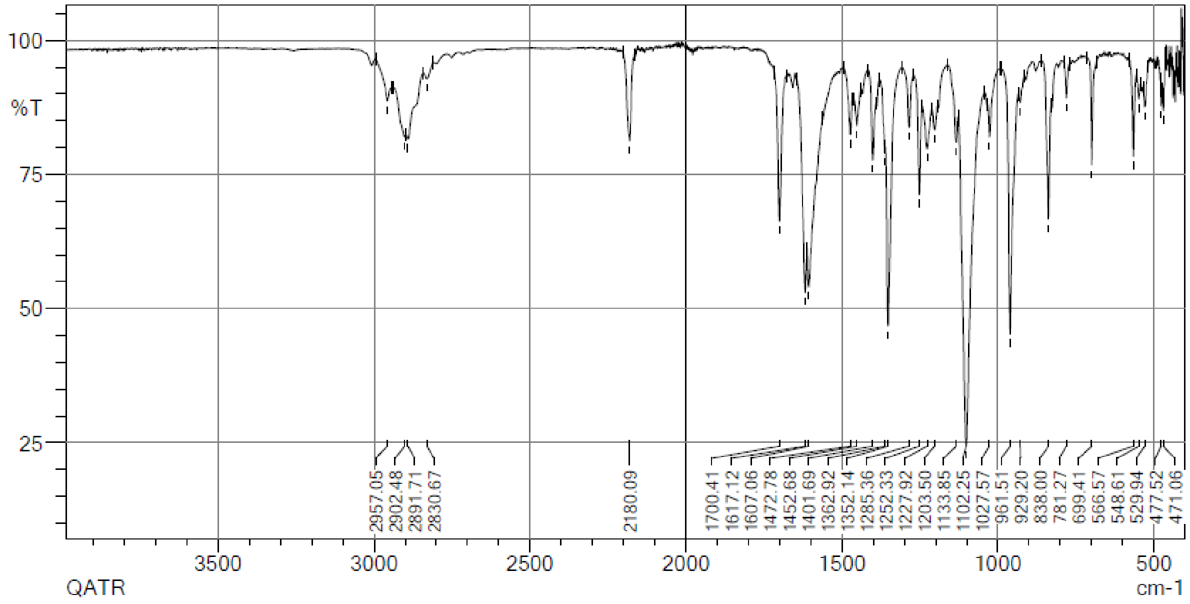

Figure S20 IR spectrum of compound **5^CN^** (solid state).

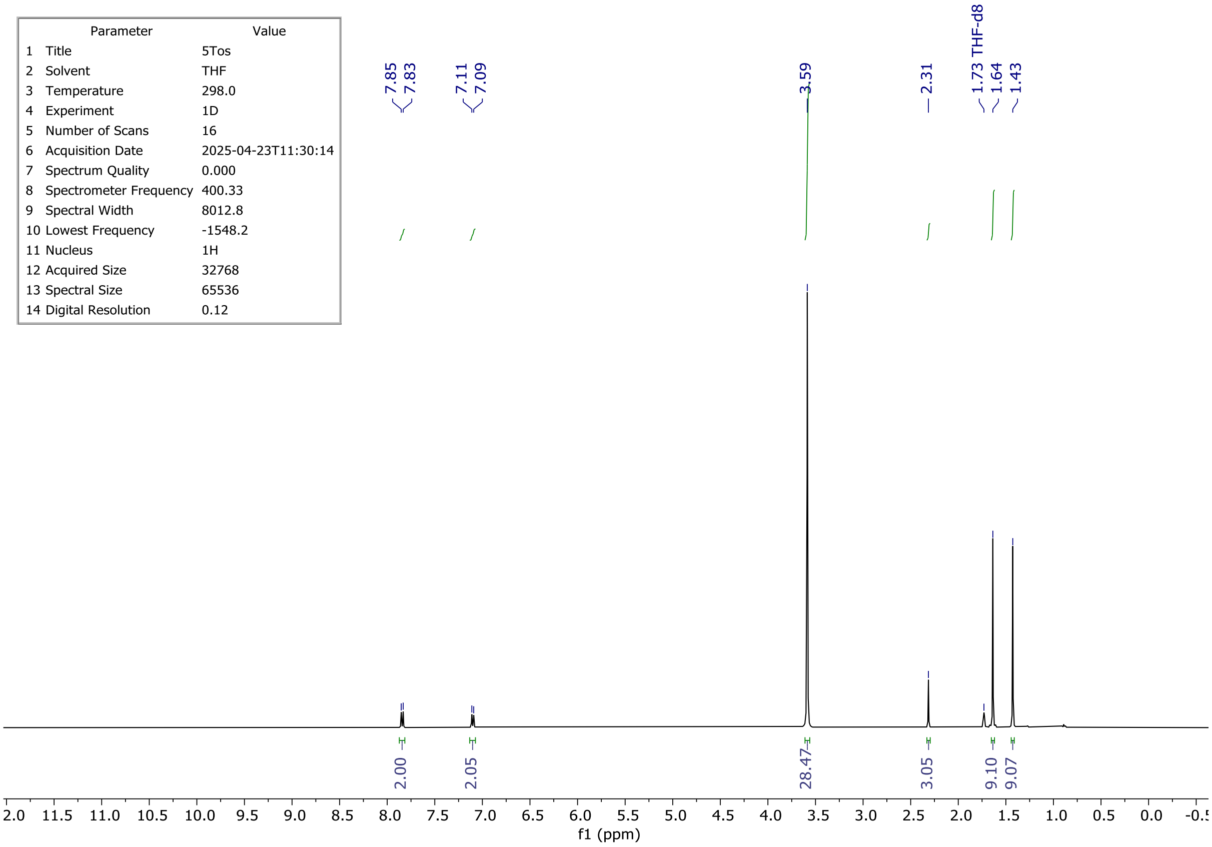

Figure S21 ^{1}H NMR spectrum of compound **5**$^{\mathbf{Tos}}$ in THF-d_8.

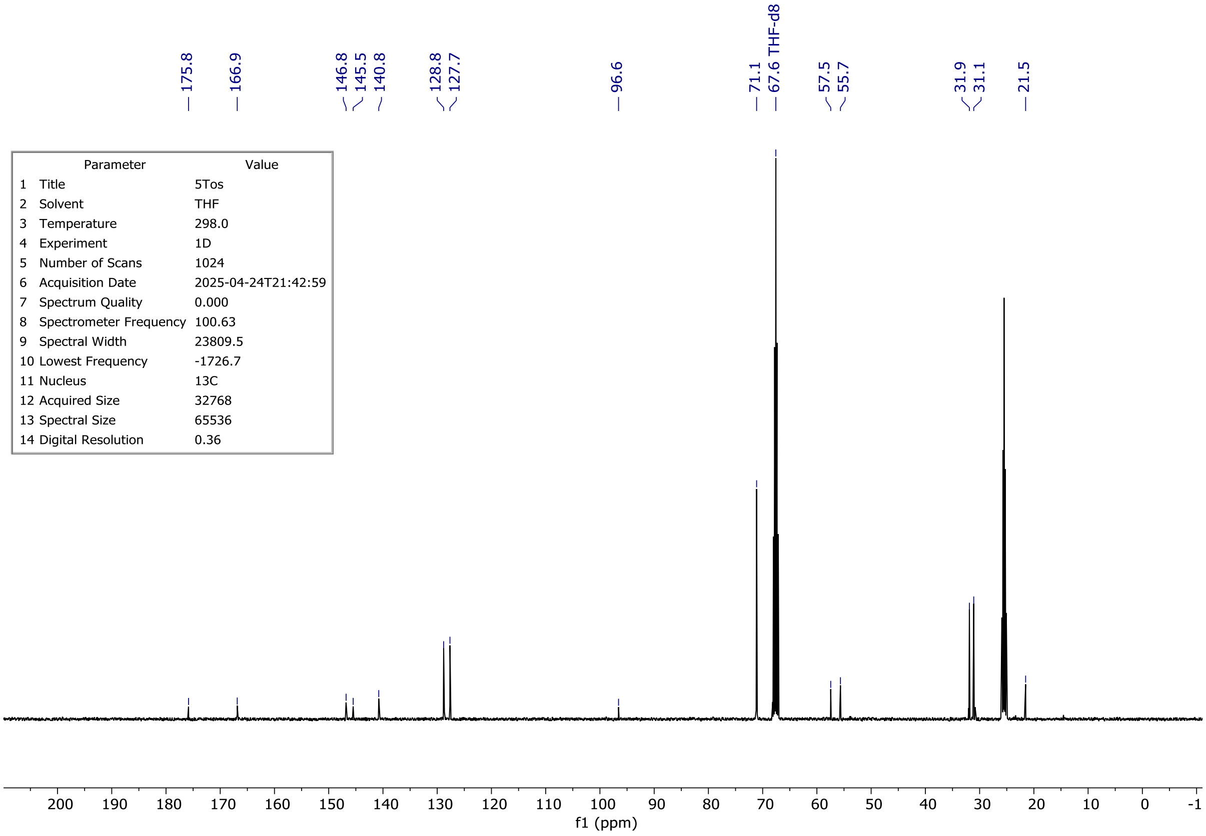

Figure S22 $^{13}C\{^1H\}$ NMR spectrum of compound **5**$^{\mathbf{Tos}}$ in THF-d_8.

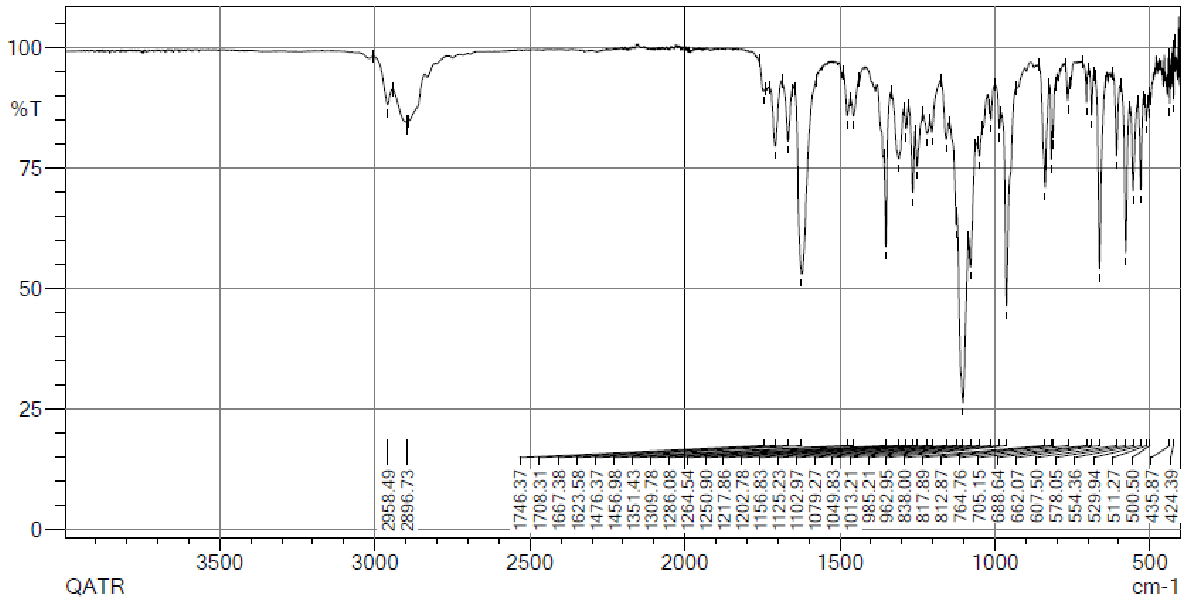

Figure S23 IR spectrum of compound **5^{Tos}** (solid state).

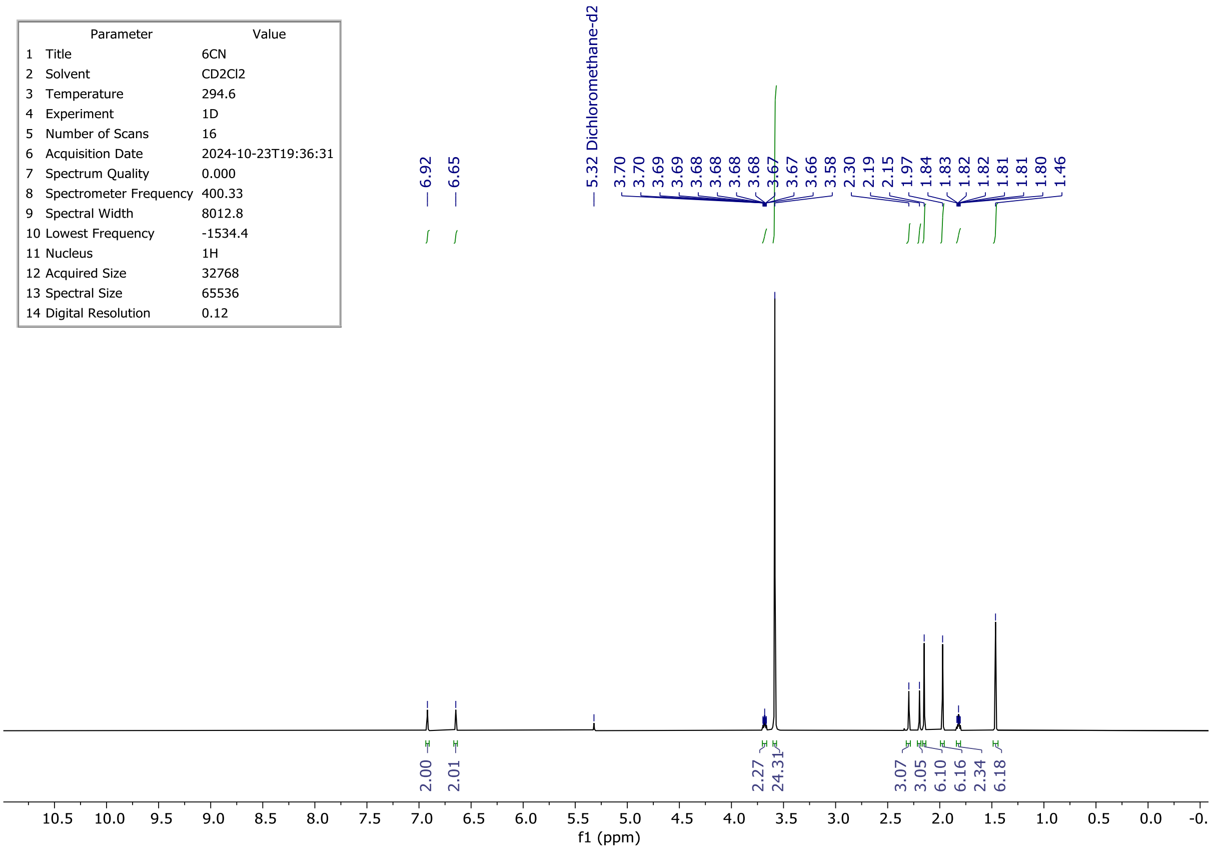

Figure S24 ^{1}H NMR spectrum of compound **6^{CN}** in CD_2Cl_2.

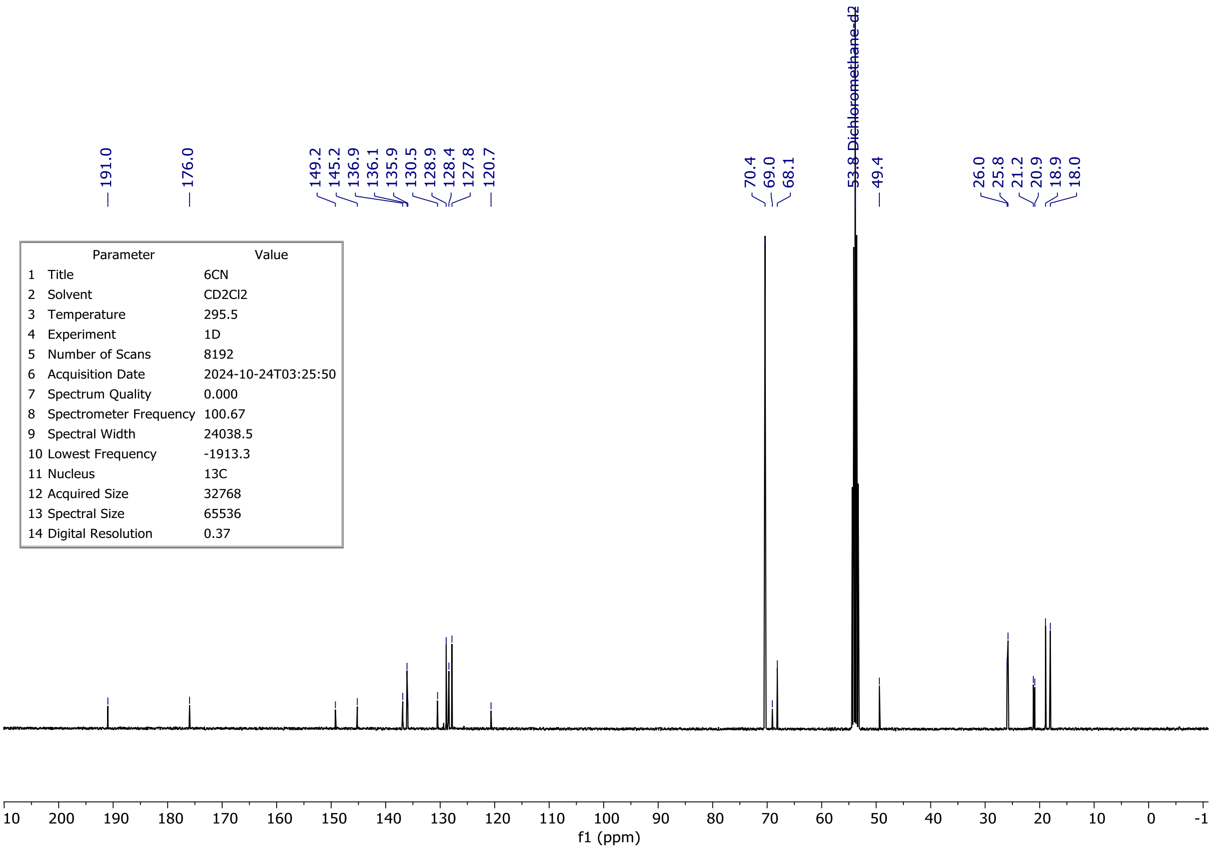

Figure S25 $^{13}C\{^{1}H\}$ NMR spectrum of compound $\mathbf{6^{CN}}$ in CD_2Cl_2.

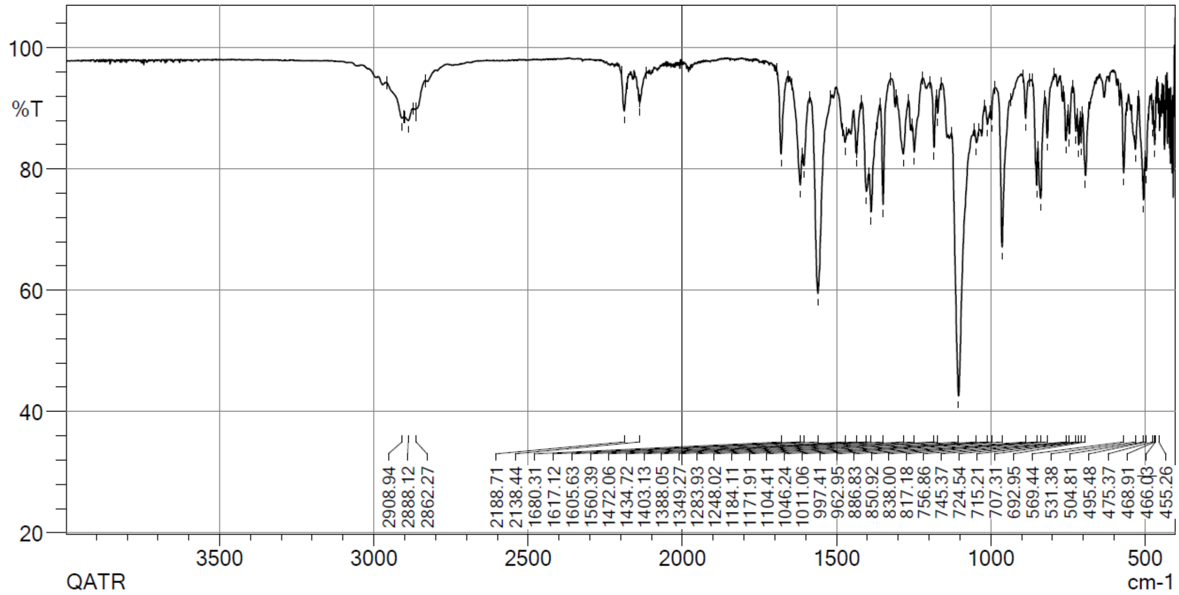

Figure S26 IR spectrum of compound $\mathbf{6^{CN}}$ (solid state).

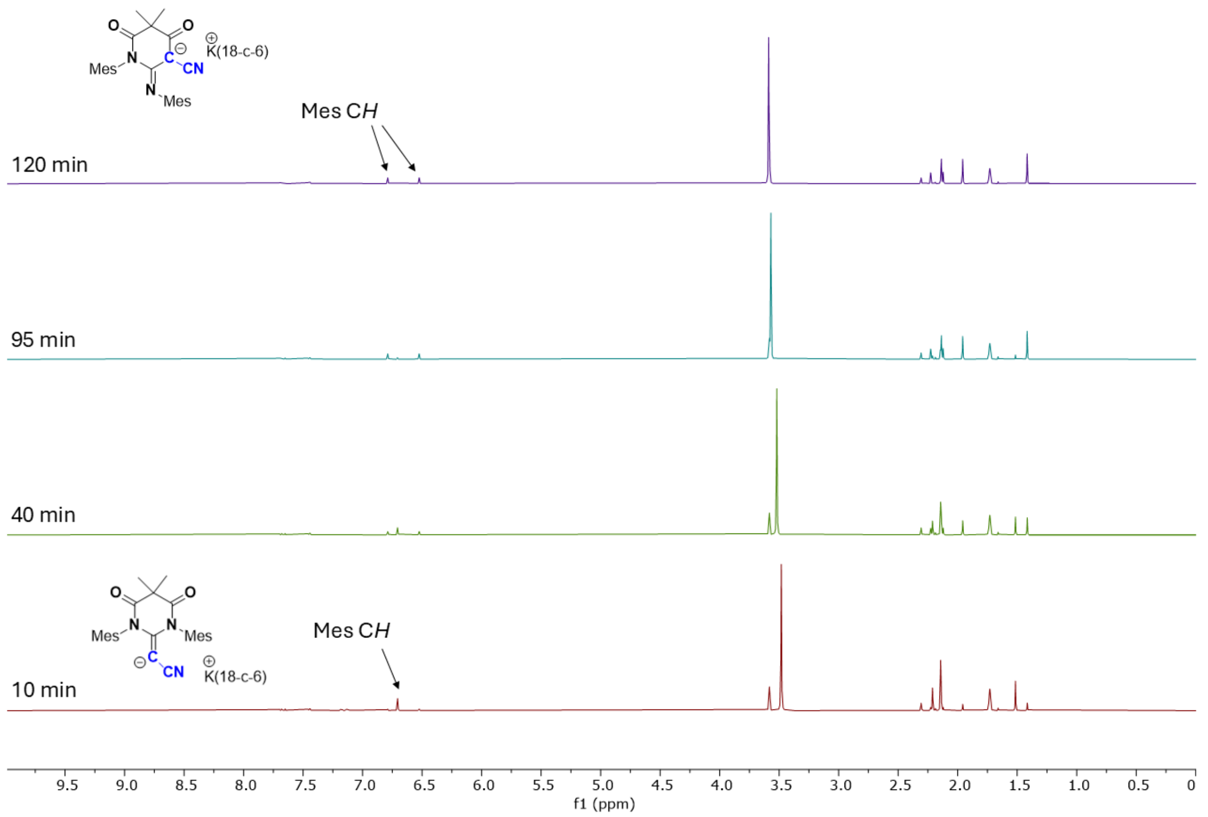

Figure S27 Monitoring of the conversion of $\mathbf{4^{CN}}$ into $\mathbf{6^{CN}}$ by ^{1}H NMR spectroscopy in THF-d8 at room temperature.

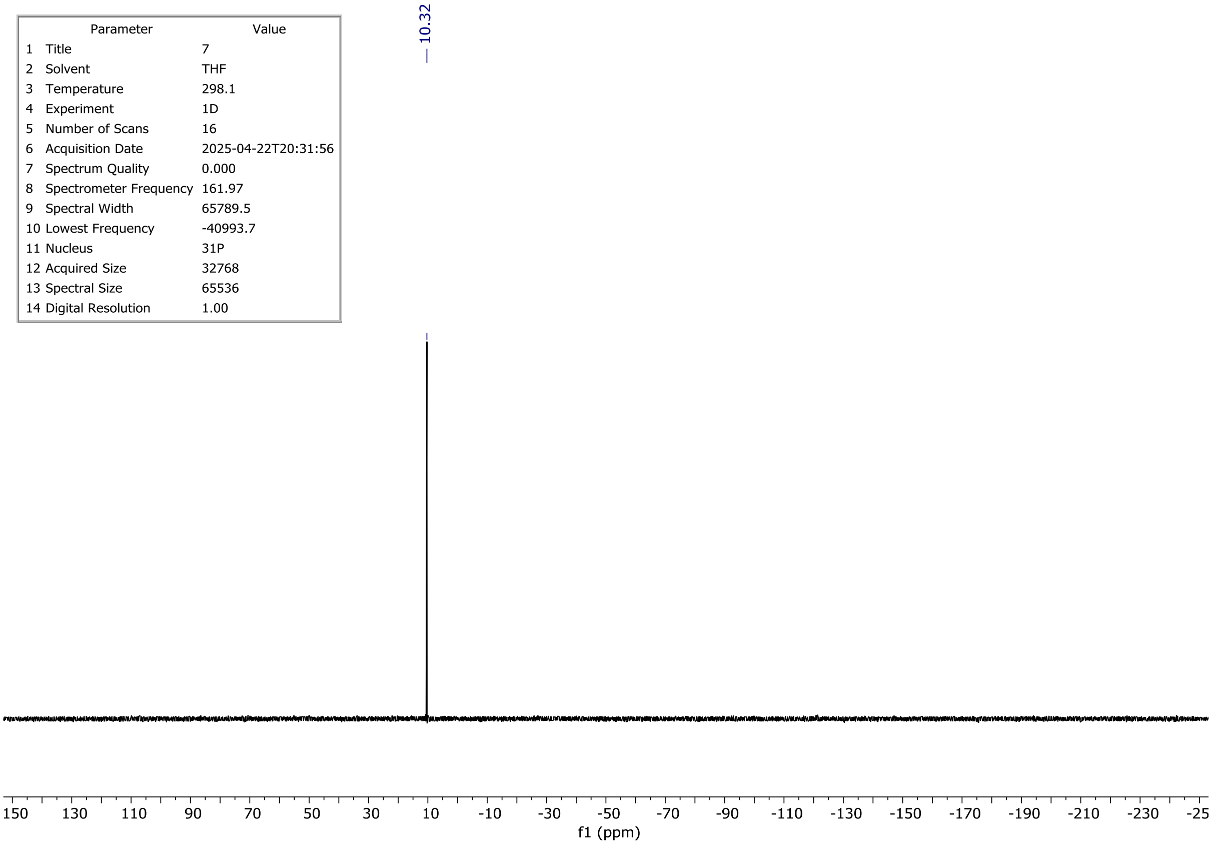

	Parameter	Value
1	Title	7
2	Solvent	THF
3	Temperature	298.1
4	Experiment	1D
5	Number of Scans	16
6	Acquisition Date	2025-04-22T20:31:56
7	Spectrum Quality	0.000
8	Spectrometer Frequency	161.97
9	Spectral Width	65789.5
10	Lowest Frequency	-40993.7
11	Nucleus	31P
12	Acquired Size	32768
13	Spectral Size	65536
14	Digital Resolution	1.00

Figure S28 ^{31}P{^{1}H} NMR spectrum of compound **7** in THF-d_8.

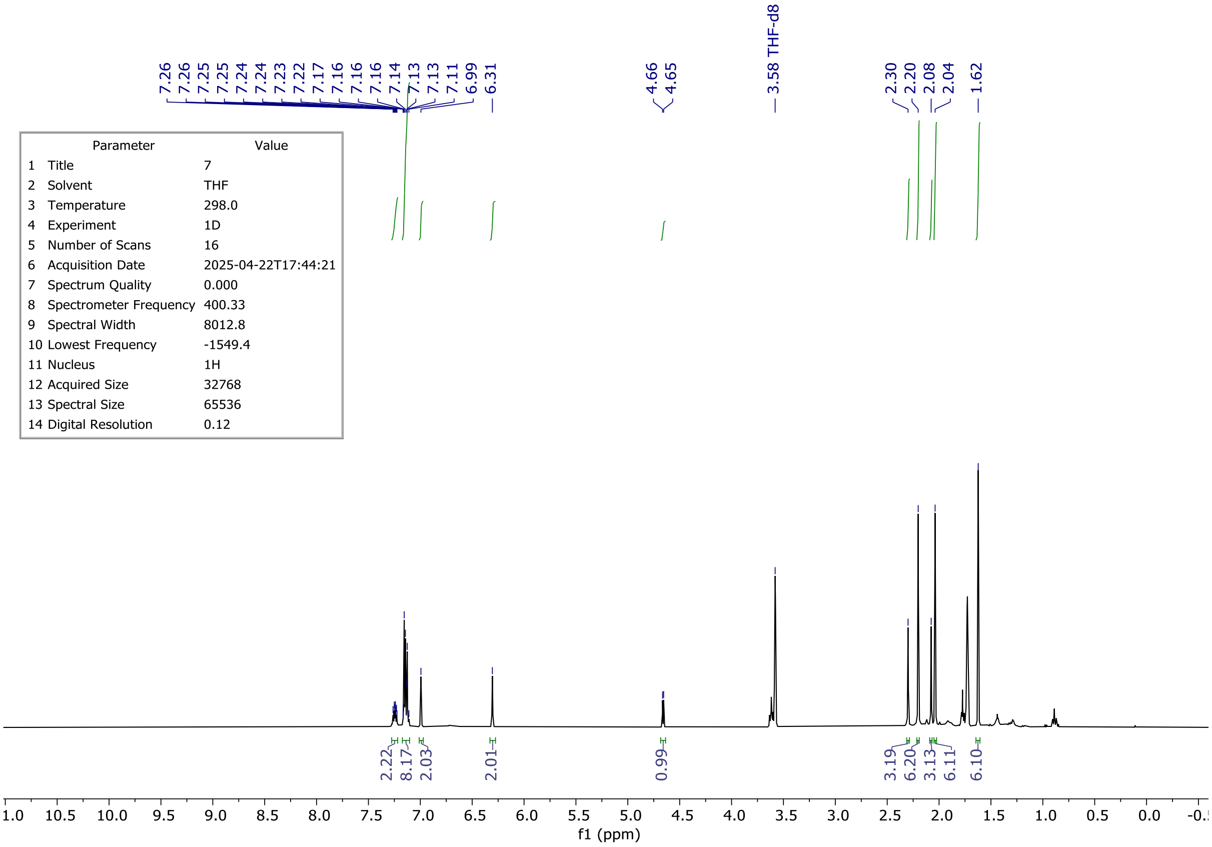

Figure S29 ^{1}H NMR spectrum of compound **7** in THF-d_8.

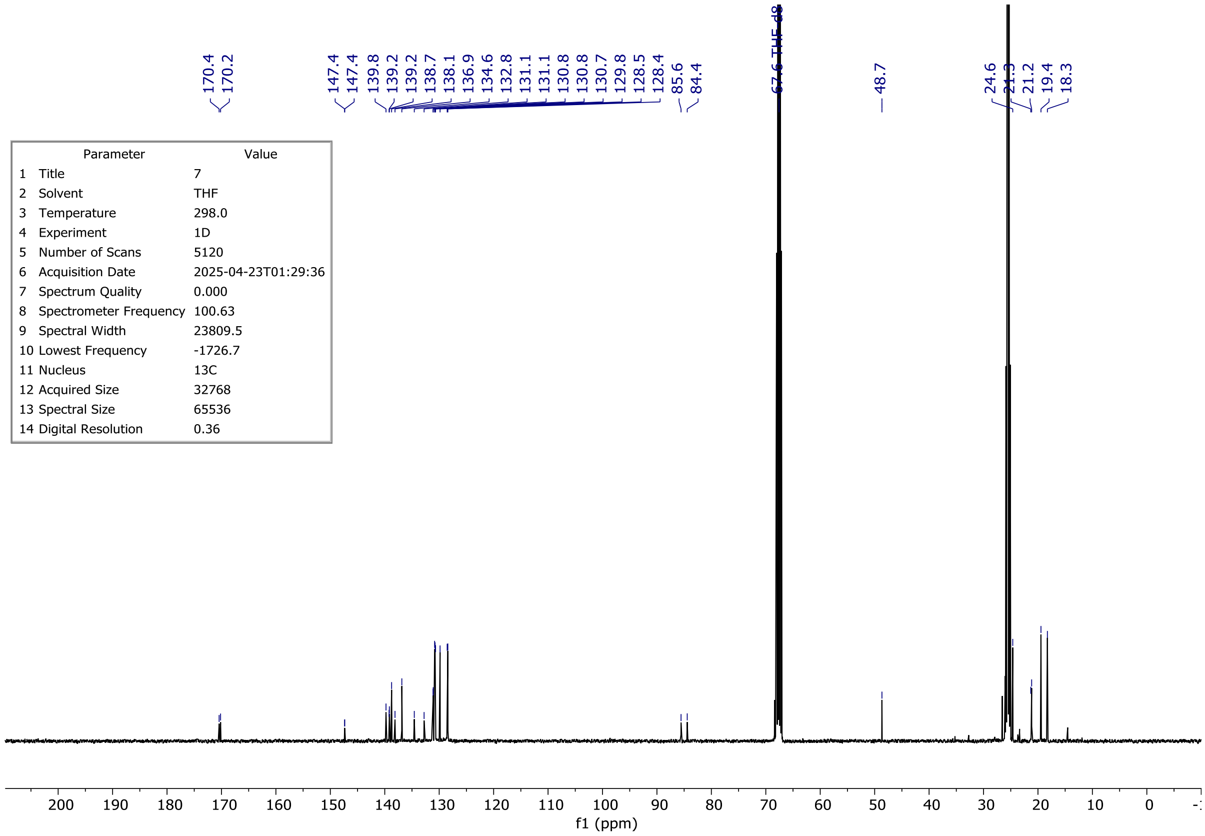

Figure S30 ^{13}C{^{1}H} NMR spectrum of compound **7** in THF-d_8.

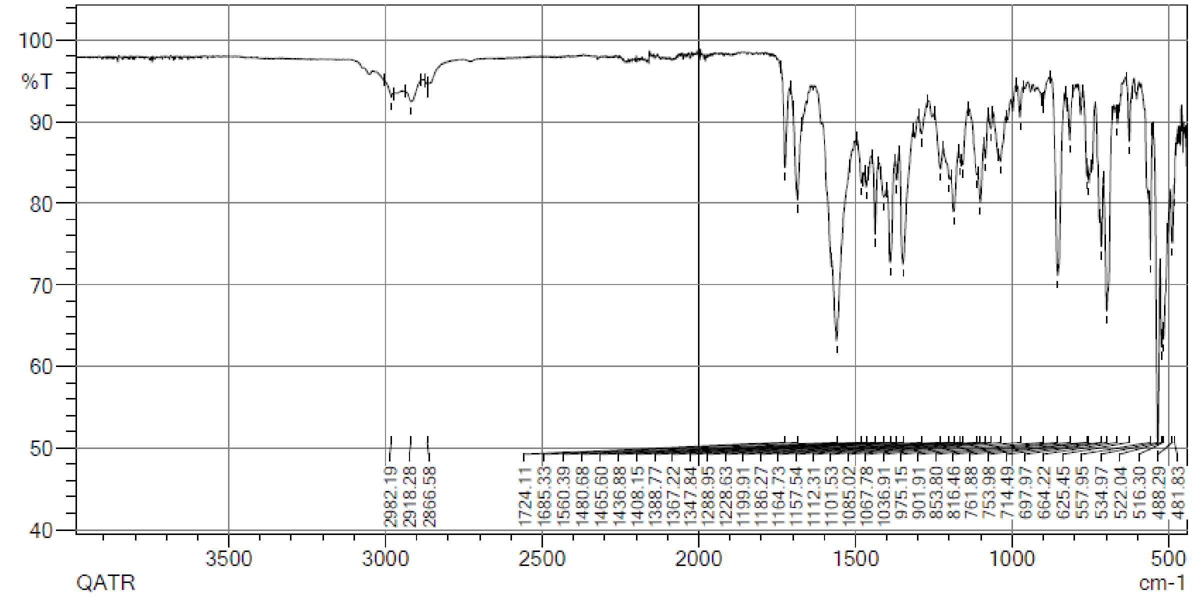

Figure S31 IR spectrum of compound **7** (solid state).

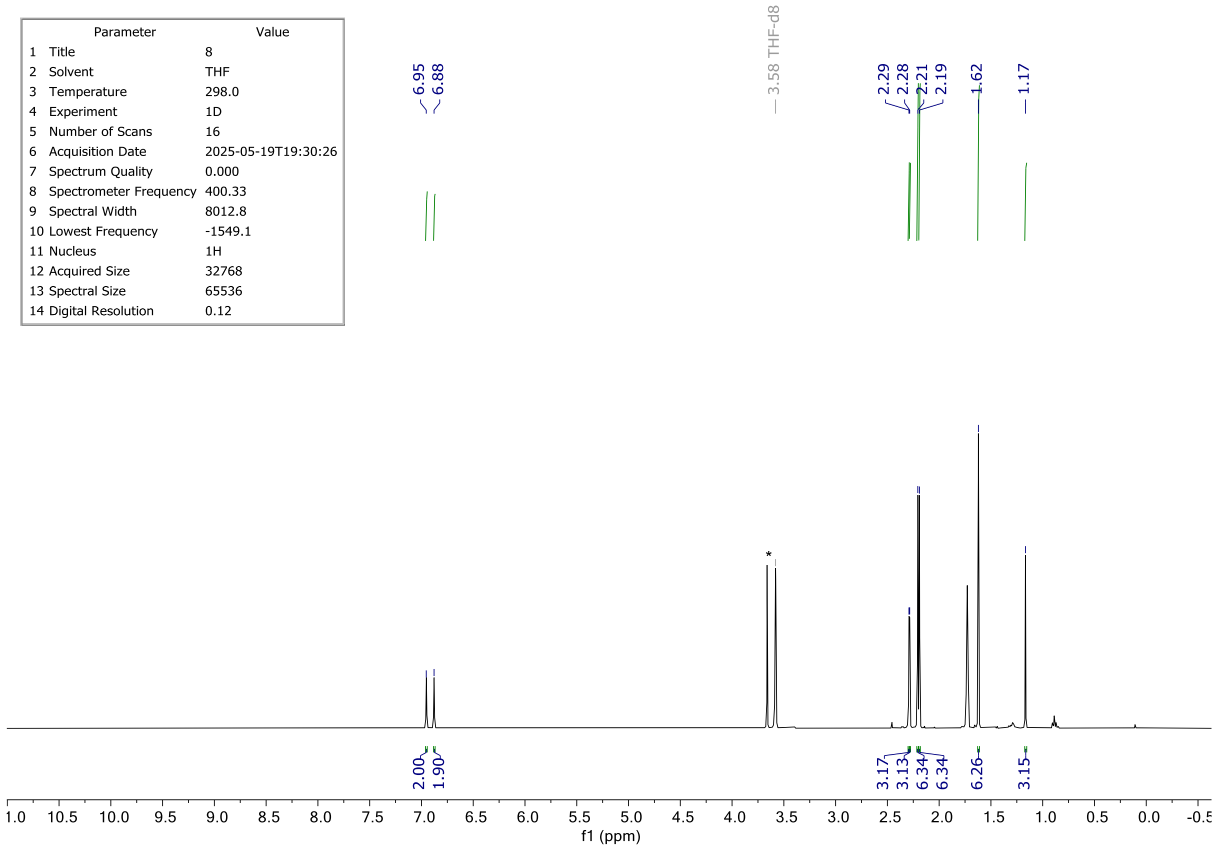

Figure S32 ^{1}H NMR spectrum of compound **8** in THF-d_8. * corresponds to the 18-c-6, which is not separable.

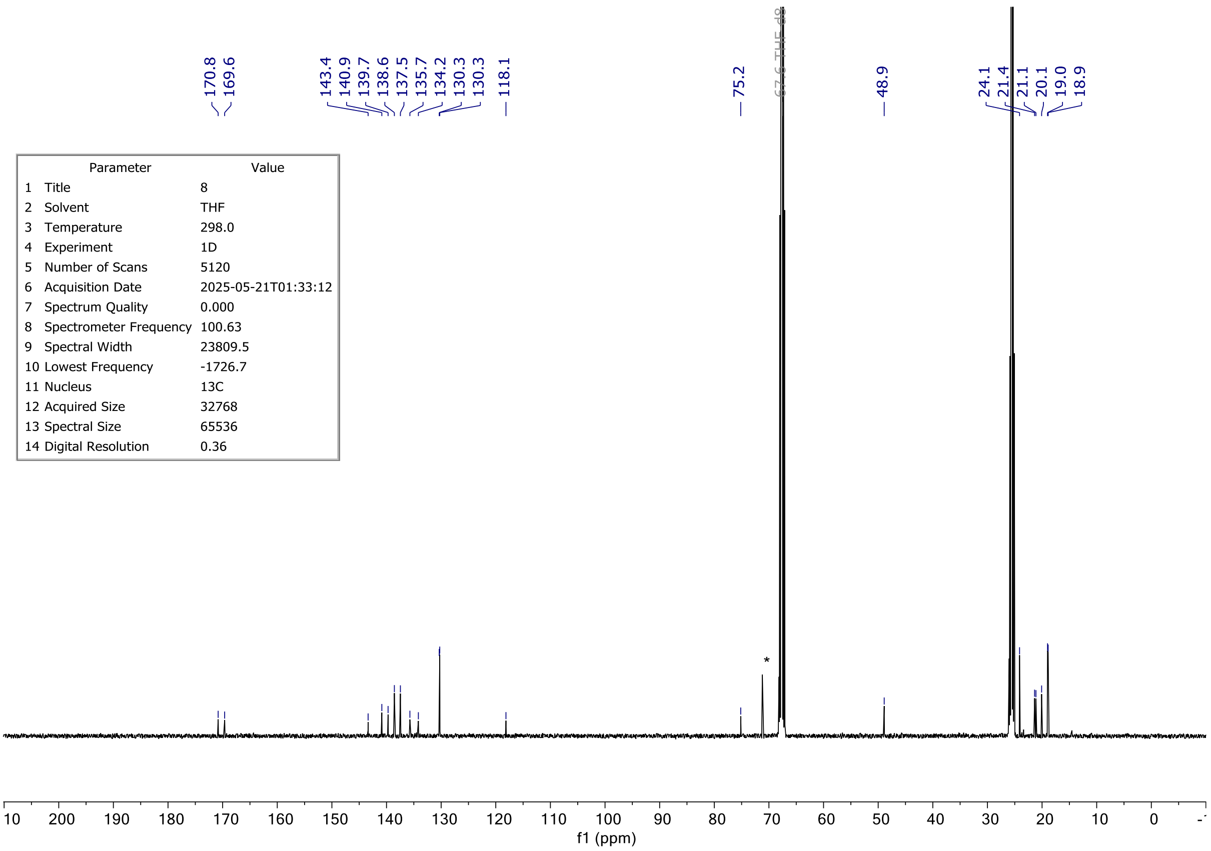

Figure S33 $^{13}C\{^{1}H\}$ NMR spectrum of compound **8** in THF-d_8. * corresponds to the 18-c-6, which is not separable.

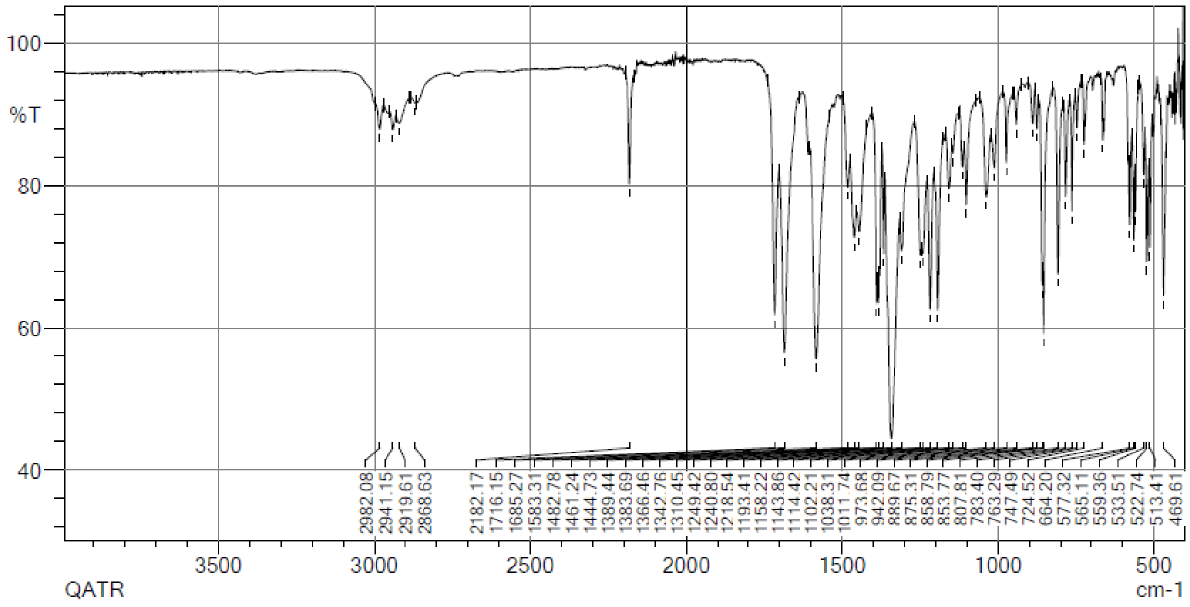

Figure S34 IR spectrum of compound **8** (solid state).

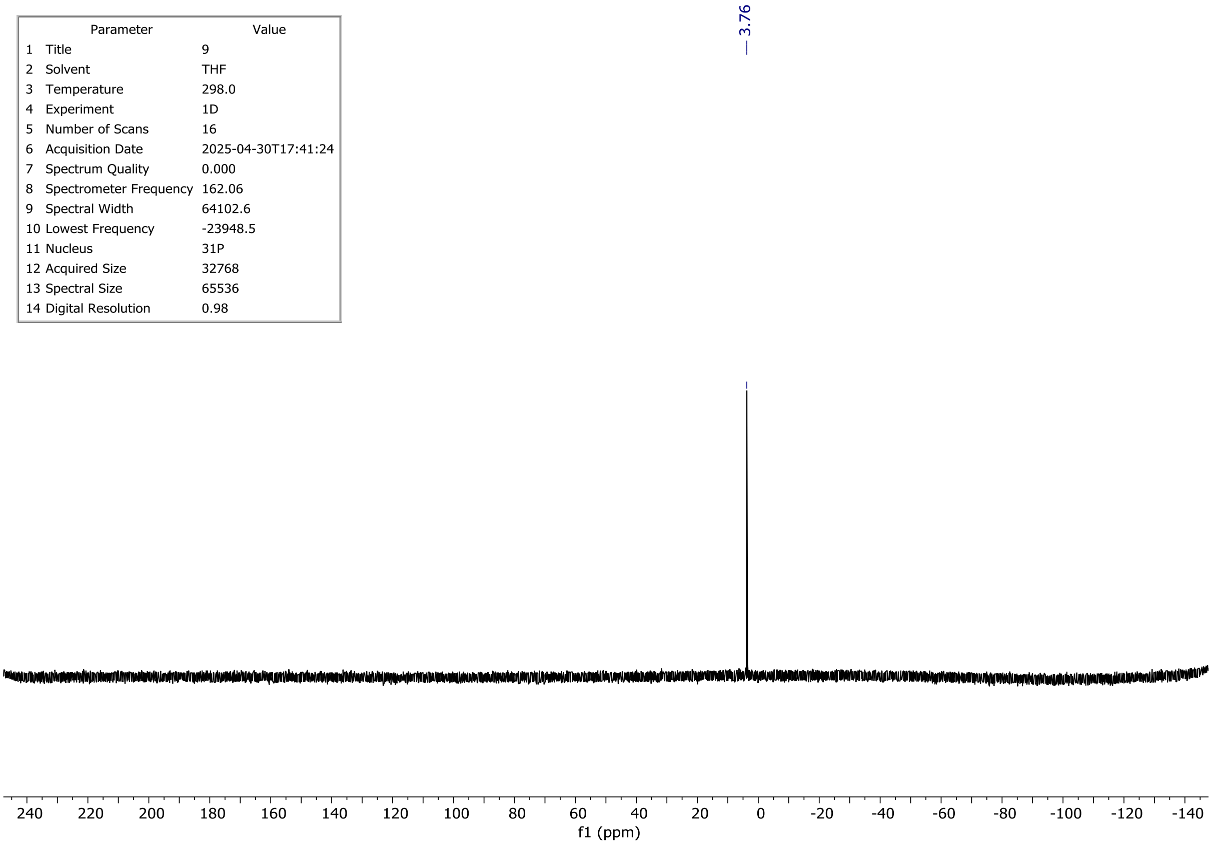

Figure S35 $^{31}P\{^{1}H\}$ NMR spectrum of compound **9** in THF-d_8.

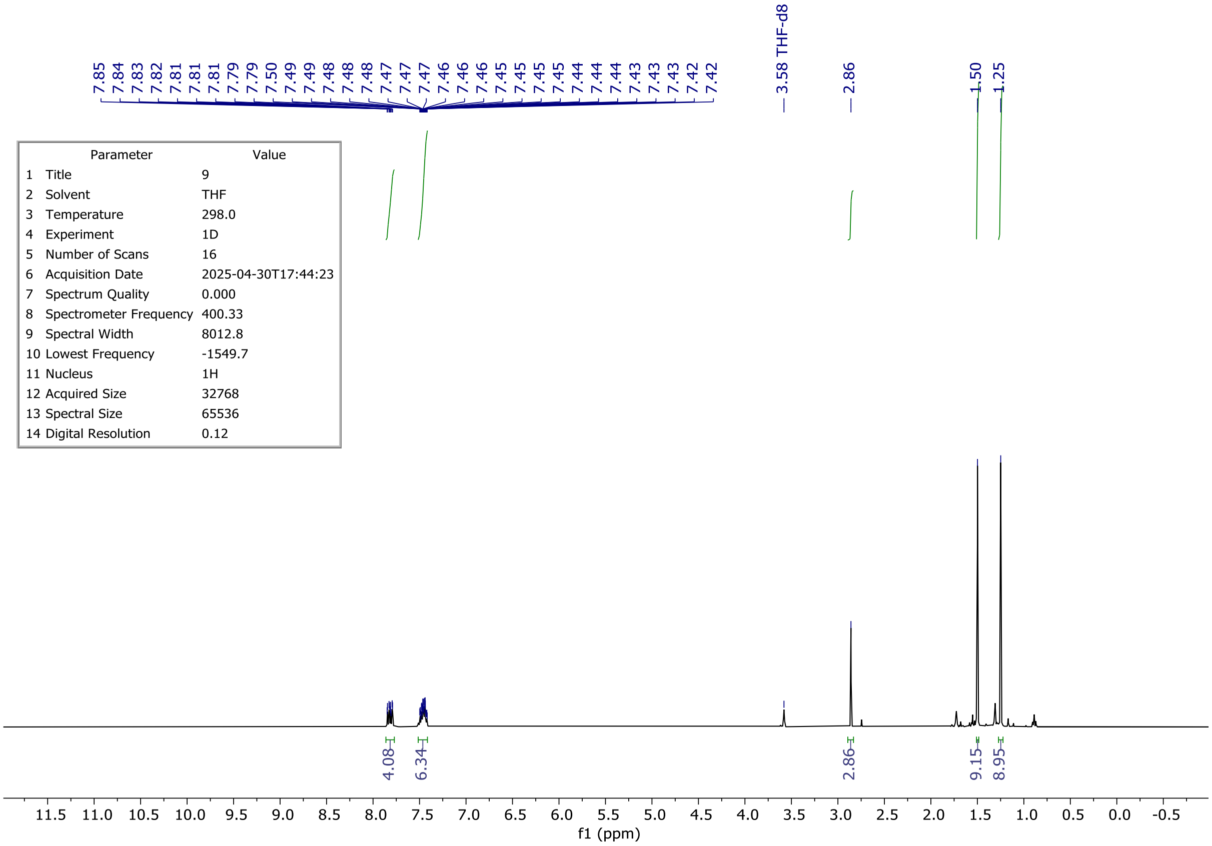

Figure S36 ^{1}H NMR spectrum of compound **9** in THF-d_8.

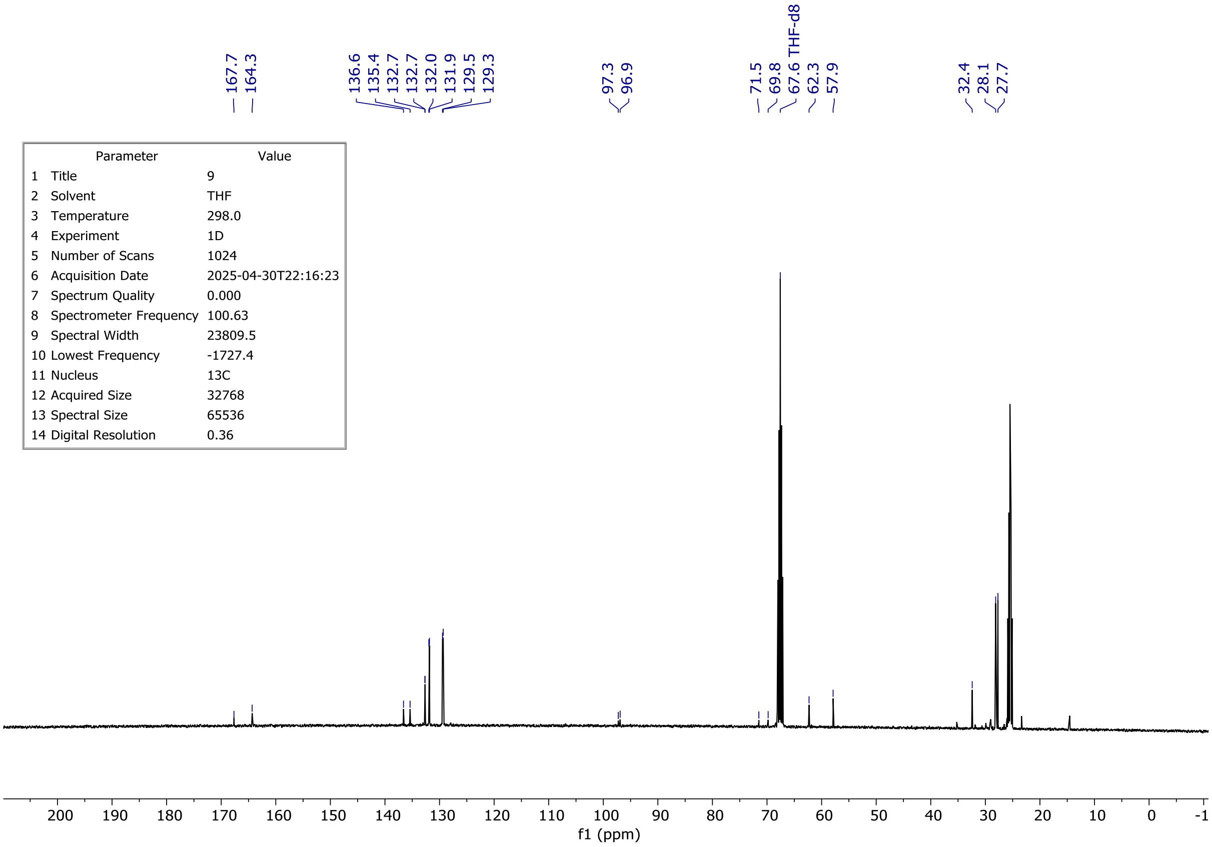

	Parameter	Value
1	Title	9
2	Solvent	THF
3	Temperature	298.0
4	Experiment	1D
5	Number of Scans	1024
6	Acquisition Date	2025-04-30T22:16:23
7	Spectrum Quality	0.000
8	Spectrometer Frequency	100.63
9	Spectral Width	23809.5
10	Lowest Frequency	-1727.4
11	Nucleus	13C
12	Acquired Size	32768
13	Spectral Size	65536
14	Digital Resolution	0.36

Figure S37 $^{13}C\{^{1}H\}$ NMR spectrum of compound **9** in THF-d_8

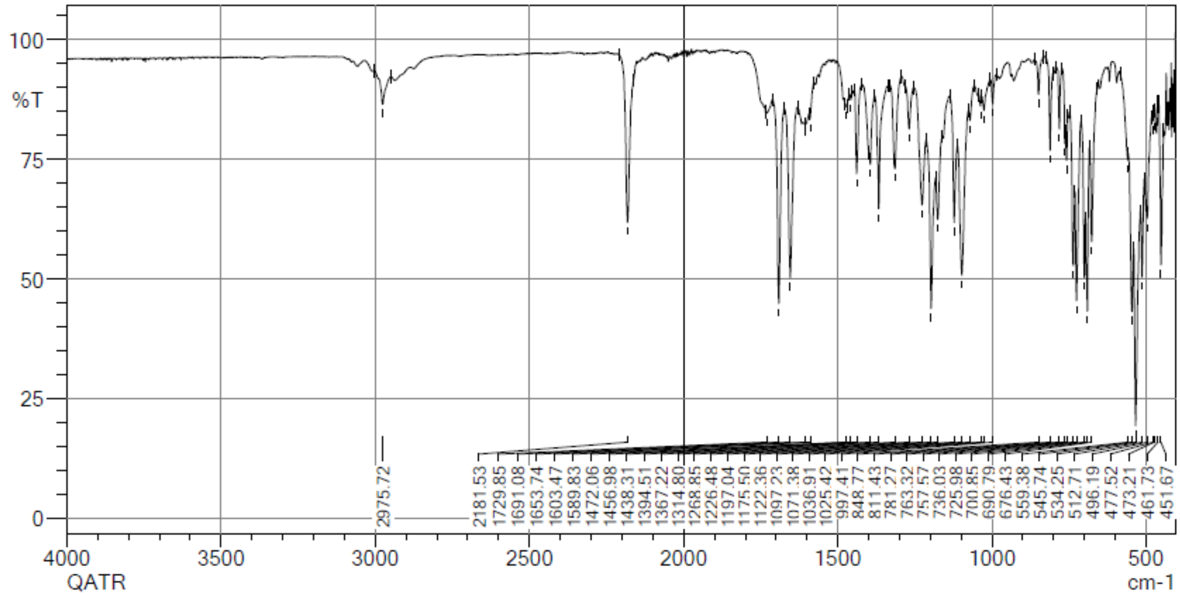

Figure S38 IR spectrum of compound **9** (solid state).

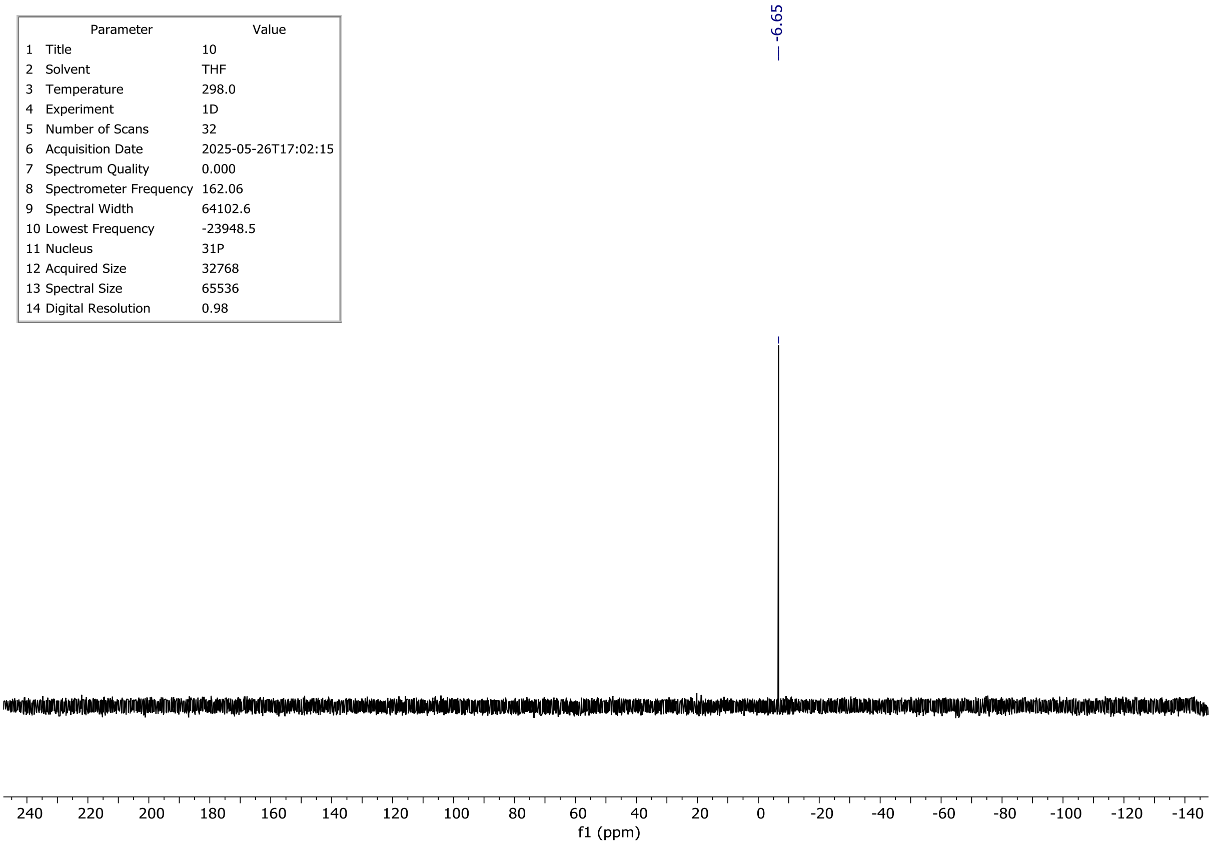

Figure S39 $^{31}P\{^{1}H\}$ NMR spectrum of compound **10** in THF-d_8.

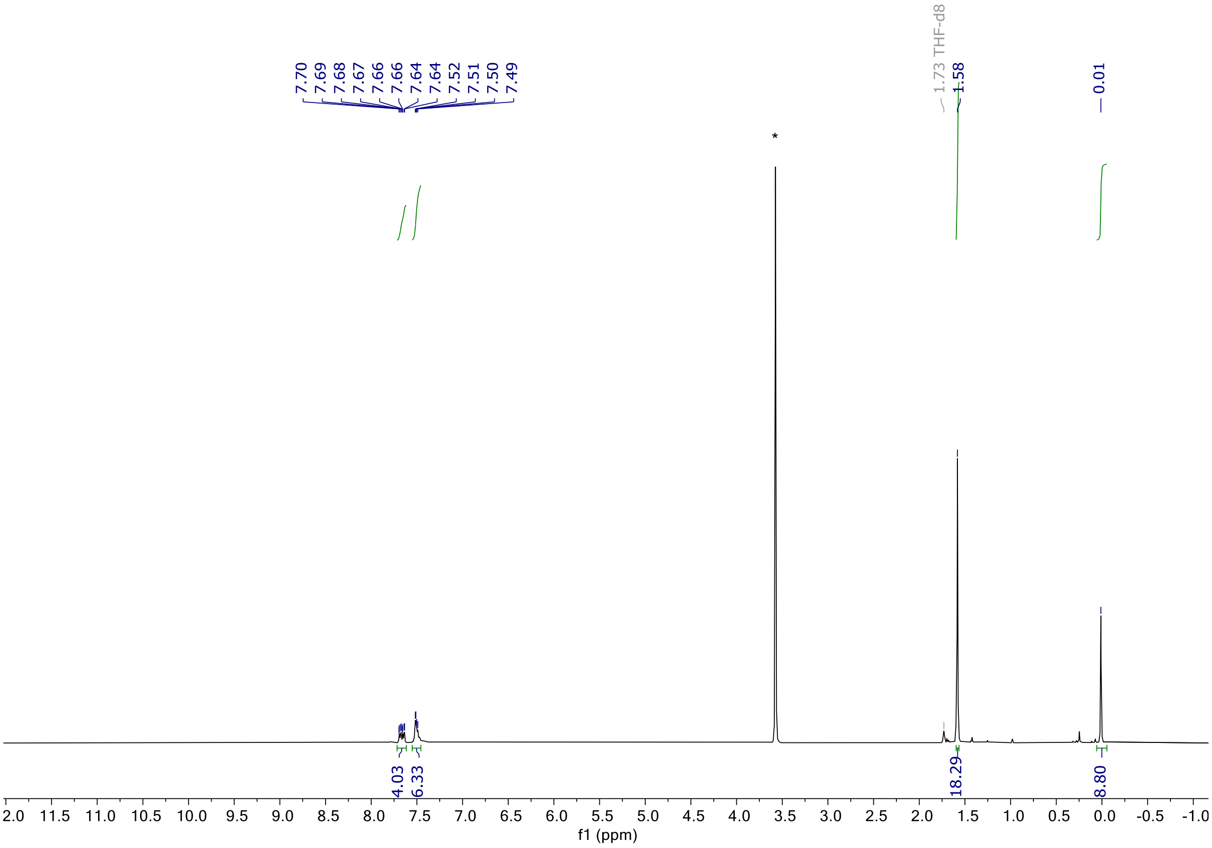

Figure S40 ^{1}H NMR spectrum of compound **10** in THF-d_8. * corresponds to the 18-c-6 which is not separable.

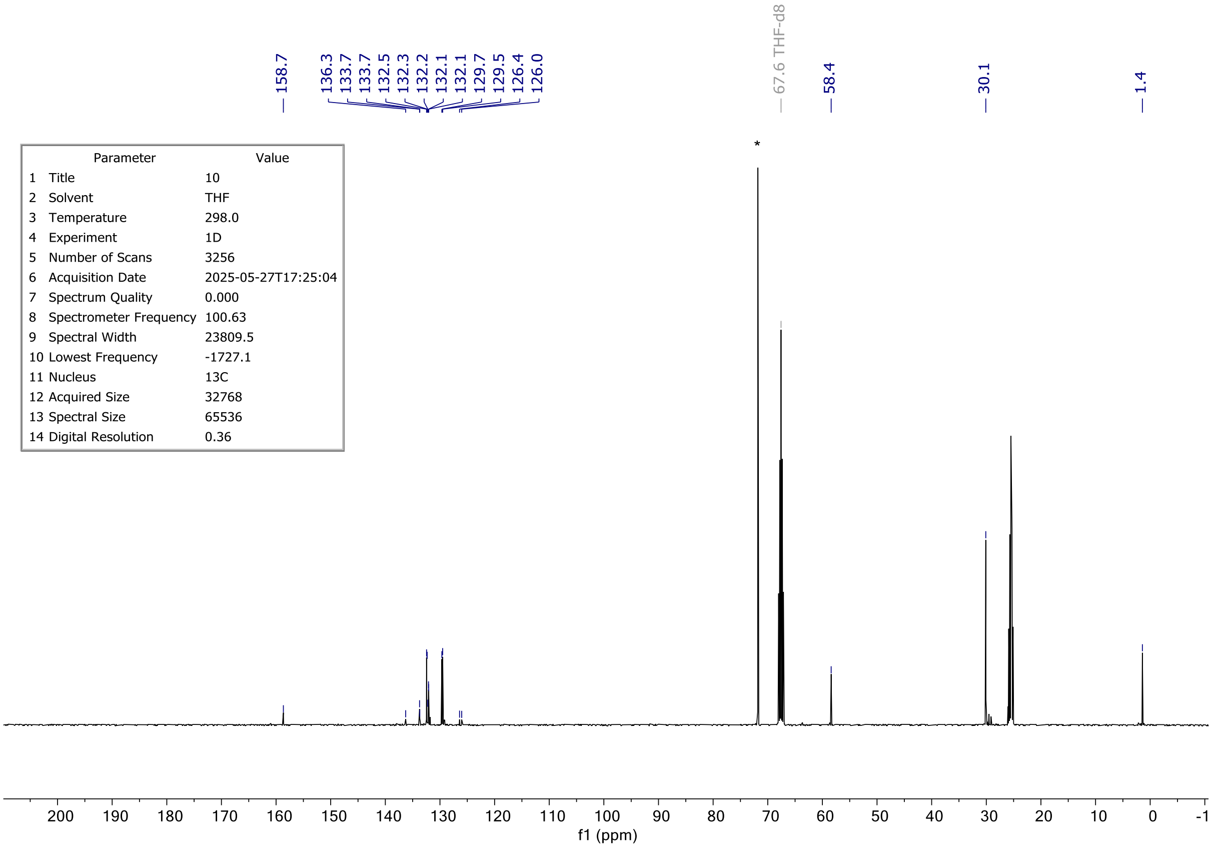

Parameter		Value
1	Title	10
2	Solvent	THF
3	Temperature	298.0
4	Experiment	1D
5	Number of Scans	3256
6	Acquisition Date	2025-05-27T17:25:04
7	Spectrum Quality	0.000
8	Spectrometer Frequency	100.63
9	Spectral Width	23809.5
10	Lowest Frequency	-1727.1
11	Nucleus	13C
12	Acquired Size	32768
13	Spectral Size	65536
14	Digital Resolution	0.36

Figure S41 $^{13}C\{^{1}H\}$ NMR spectrum of compound **10** in THF-d_8. * corresponds to the 18-c-6 which is not separable.

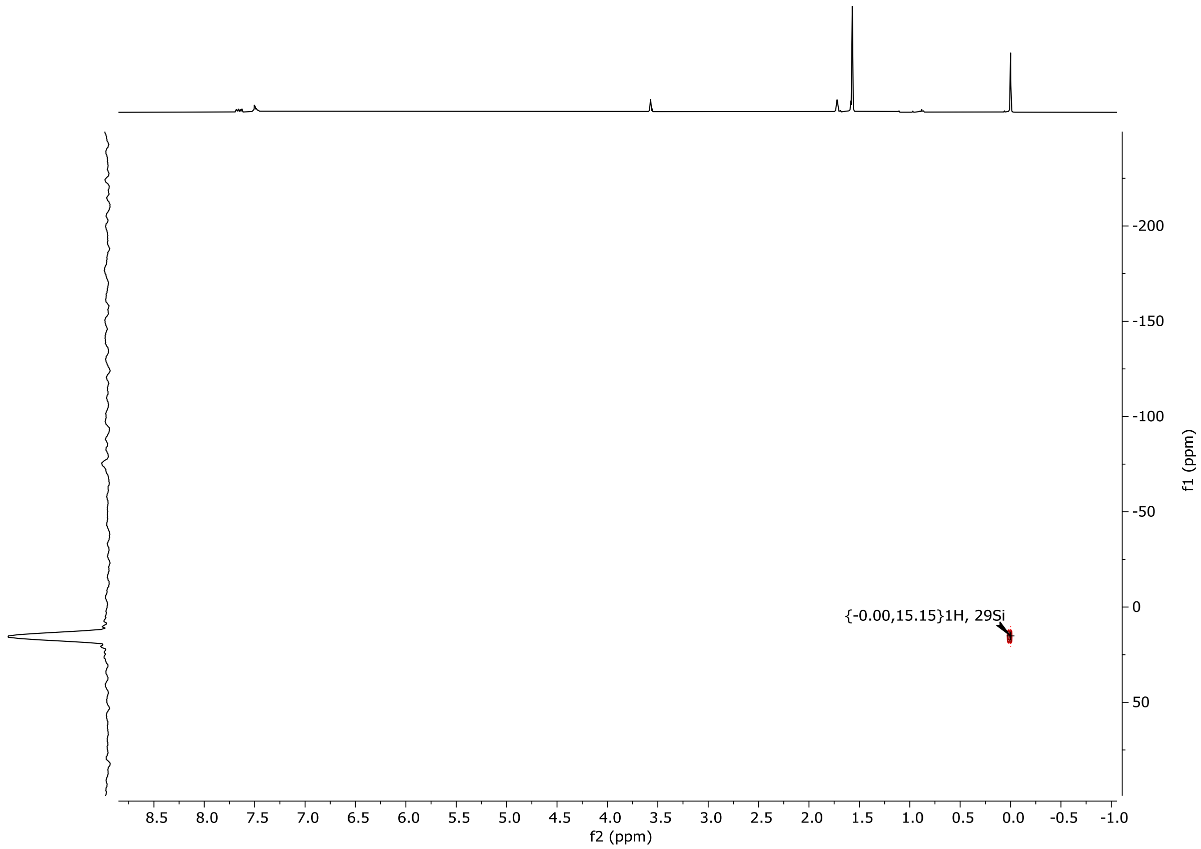

Figure S42 ^{1}H-^{29}Si HMBC spectrum of compound **10** in THF-d_8.

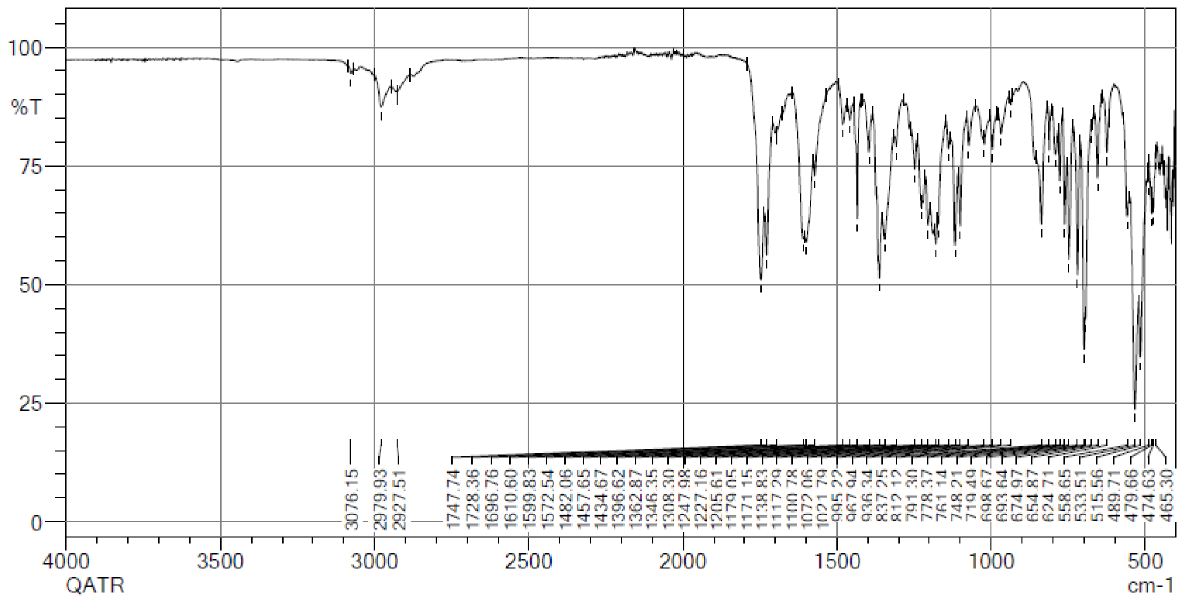

Figure S43 IR spectrum of compound **10** (solid state).

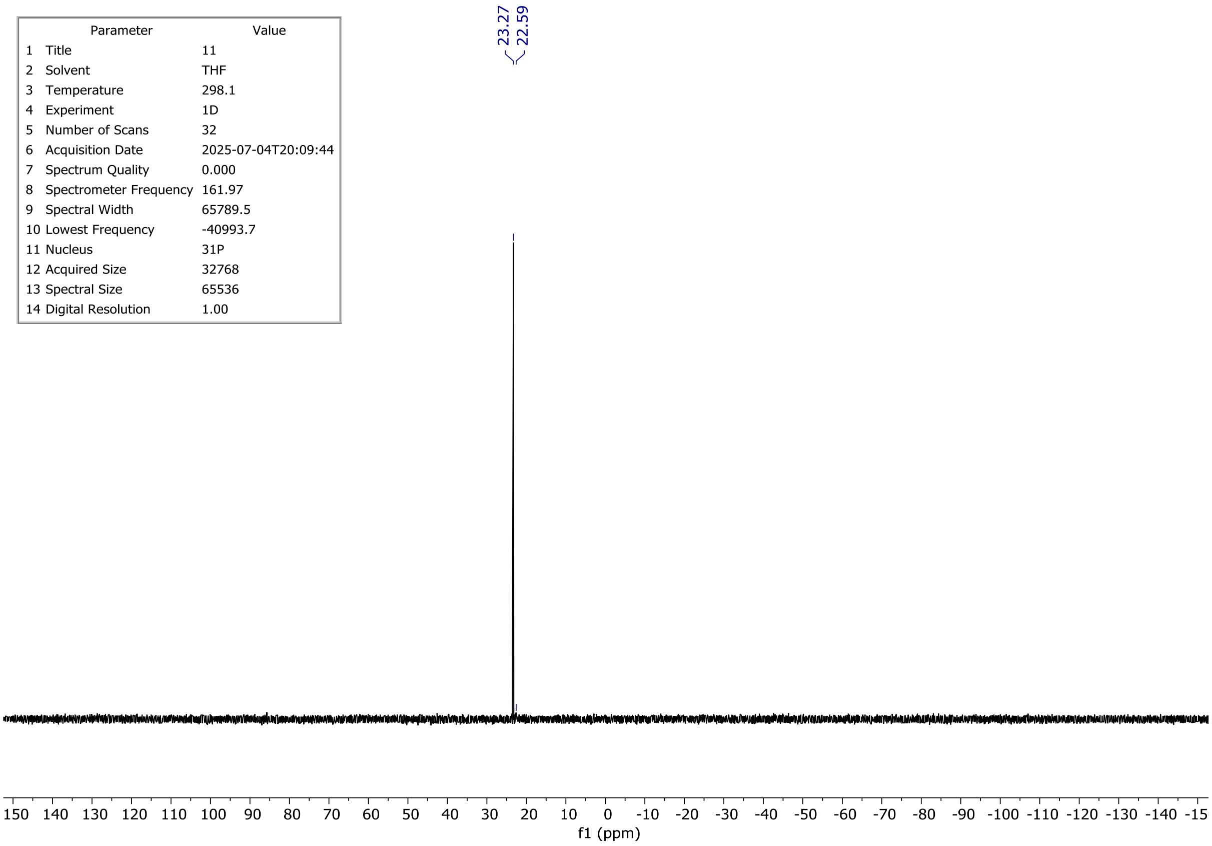

Figure S44 $^{31}P\{^{1}H\}$ NMR spectrum of compound **11** in THF-d_8.

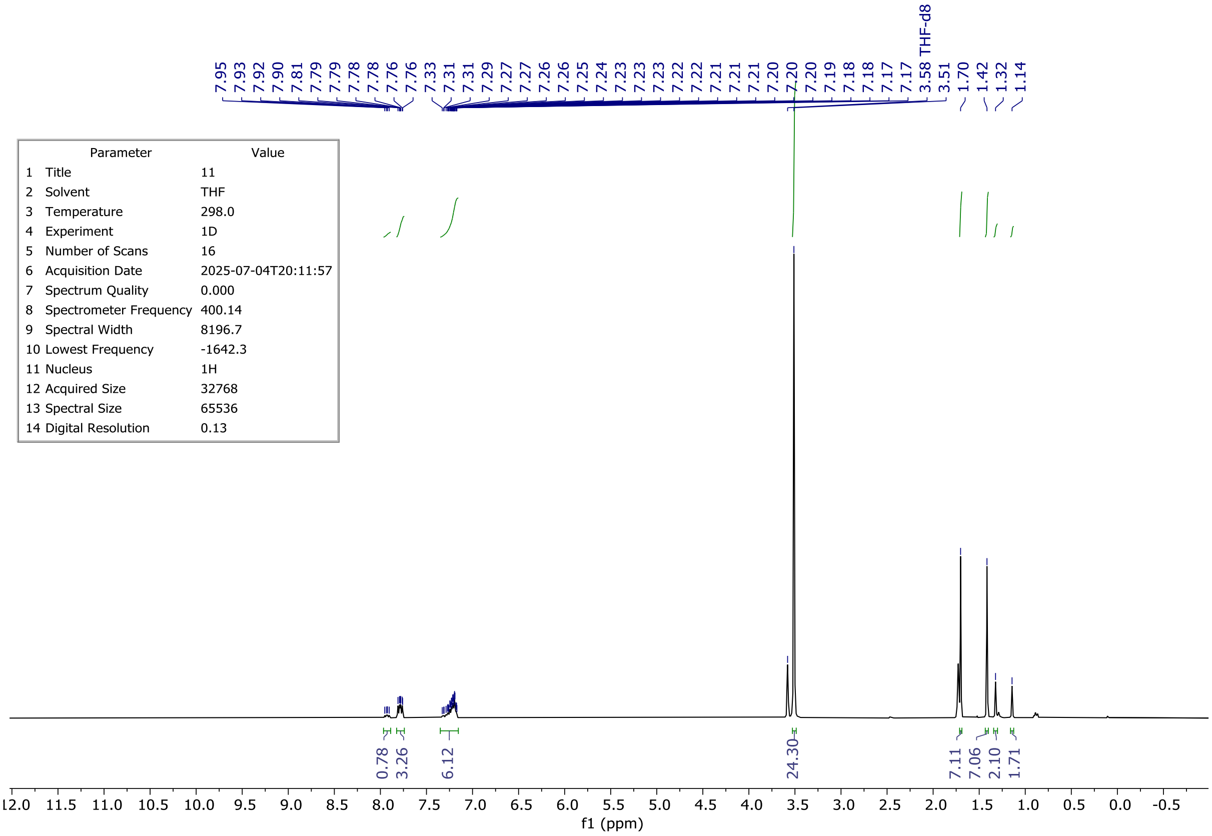

Figure S45 ^{1}H NMR spectrum of compound **11** in THF-d$_8$.

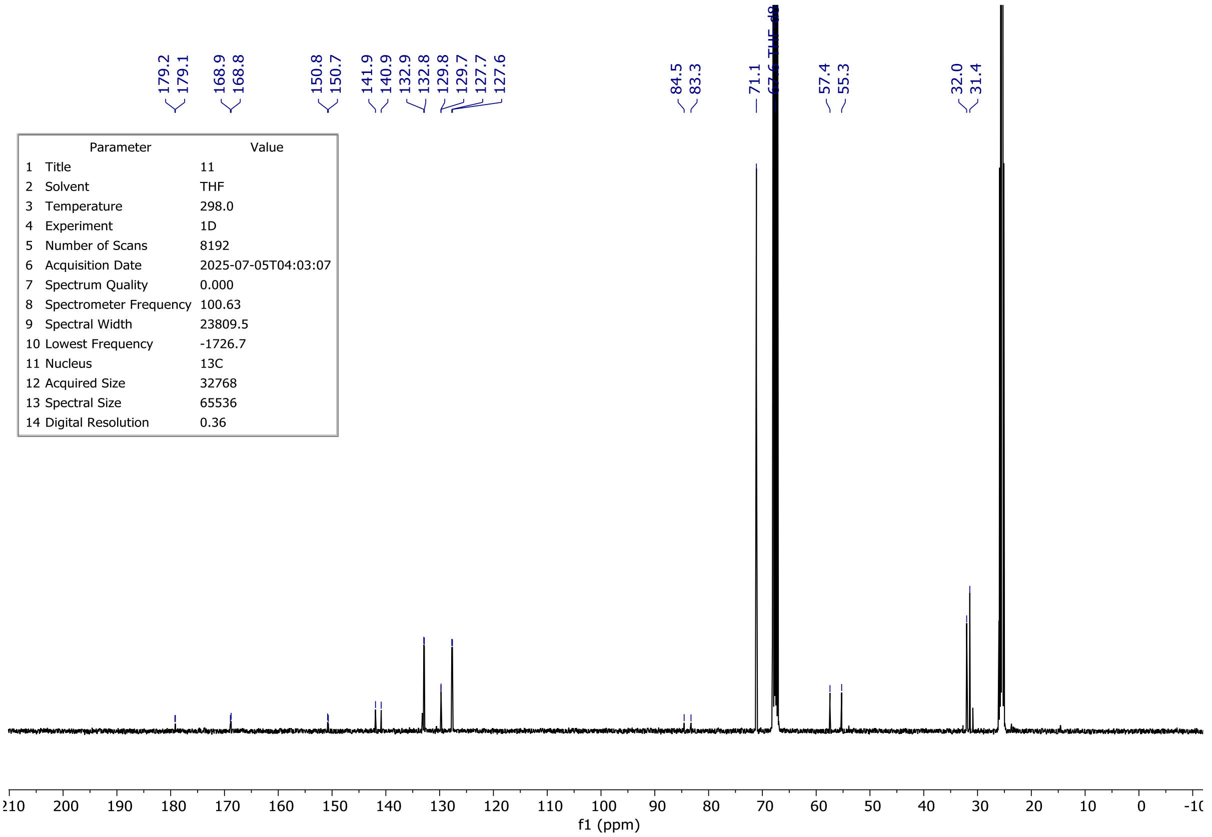

Figure S46 ^{13}C{^{1}H} NMR spectrum of compound **11** in THF-d$_8$. Note: These peaks arise for the major isomer. Some peaks for the minor isomer are also noticed.

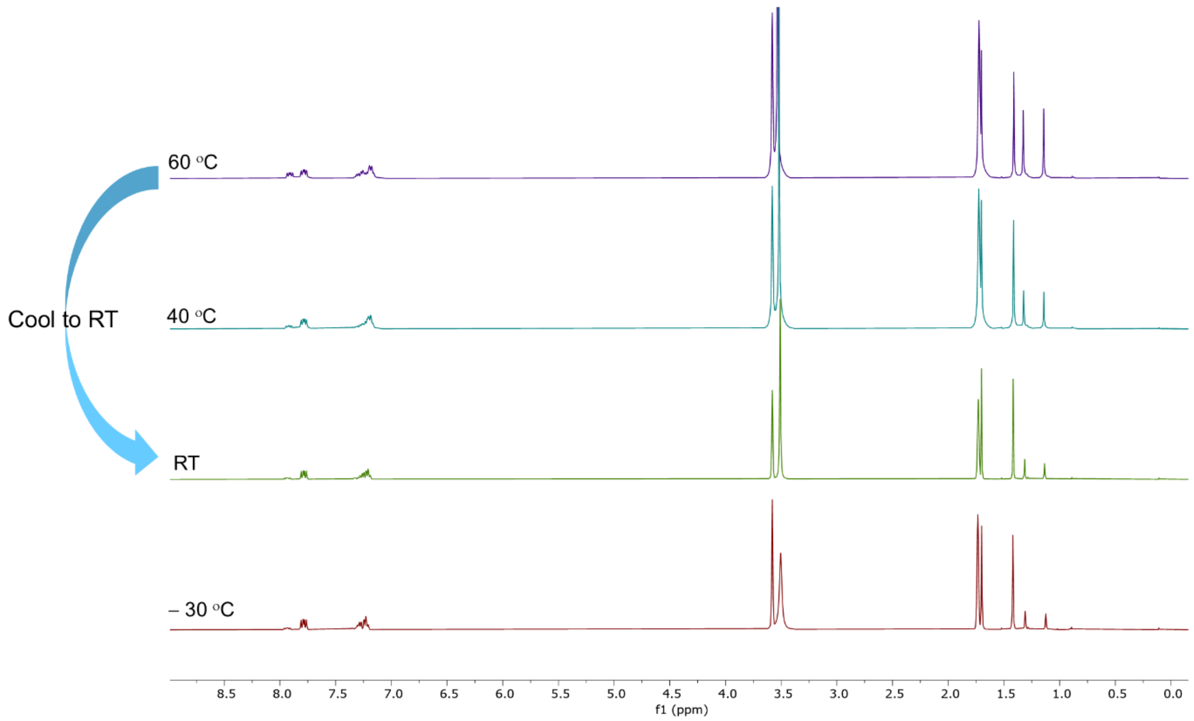

Figure S47 ^{1}H NMR spectrum of compound **11** in THF-d_8 at variable temperature.

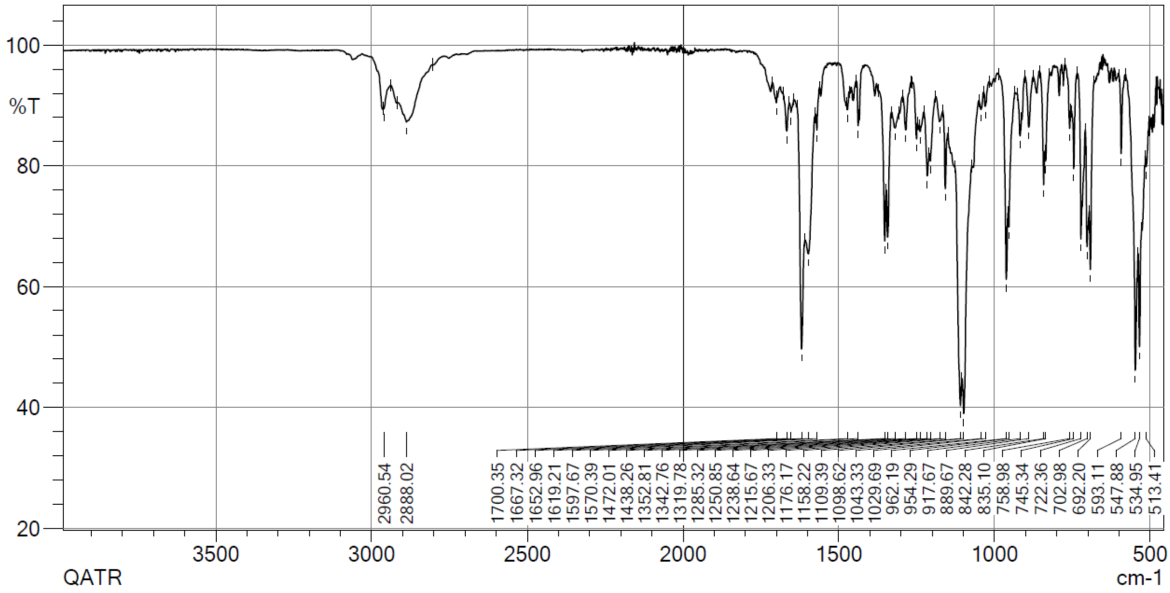

Figure S48 IR spectrum of compound **11** (solid state).

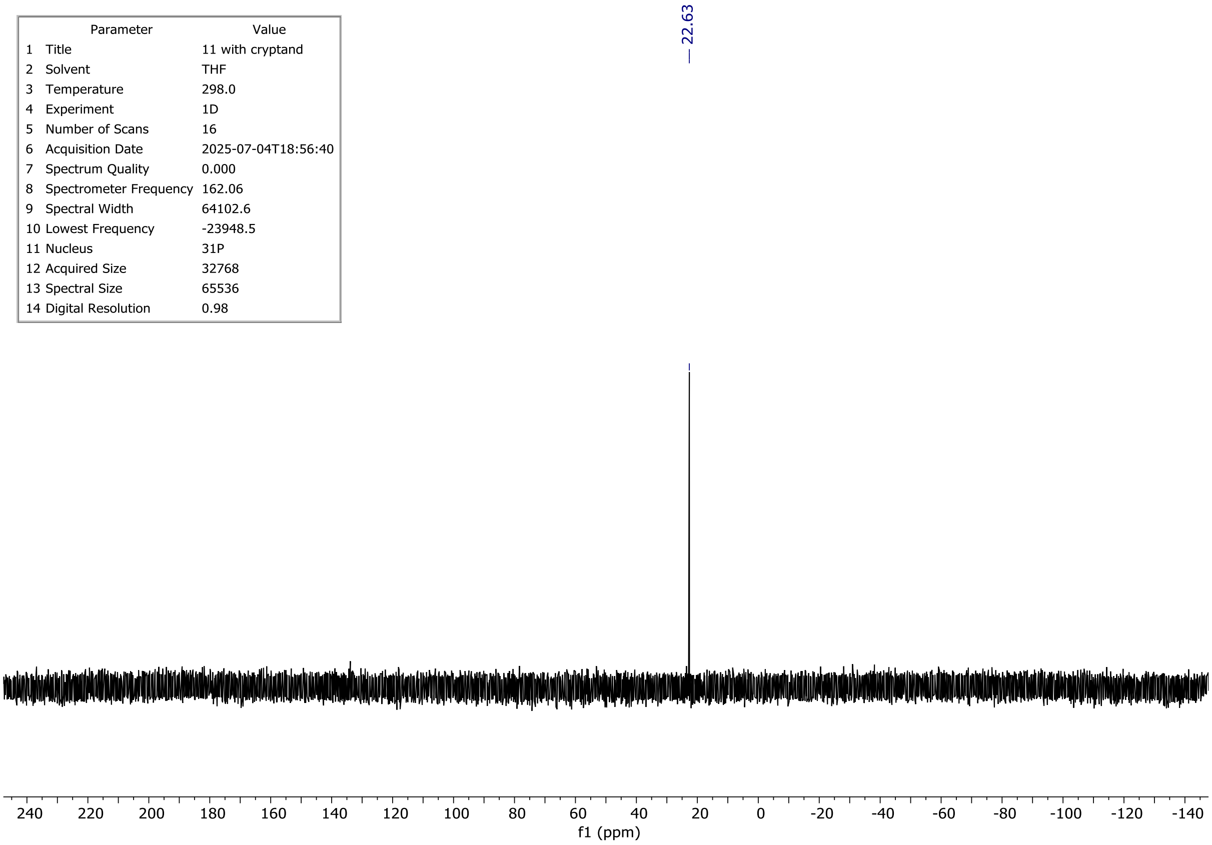

Figure S49 $^{31}P\{^{1}H\}$ NMR spectrum of compound **11** in THF-d_8 after adding 2.2.2-cryptand.

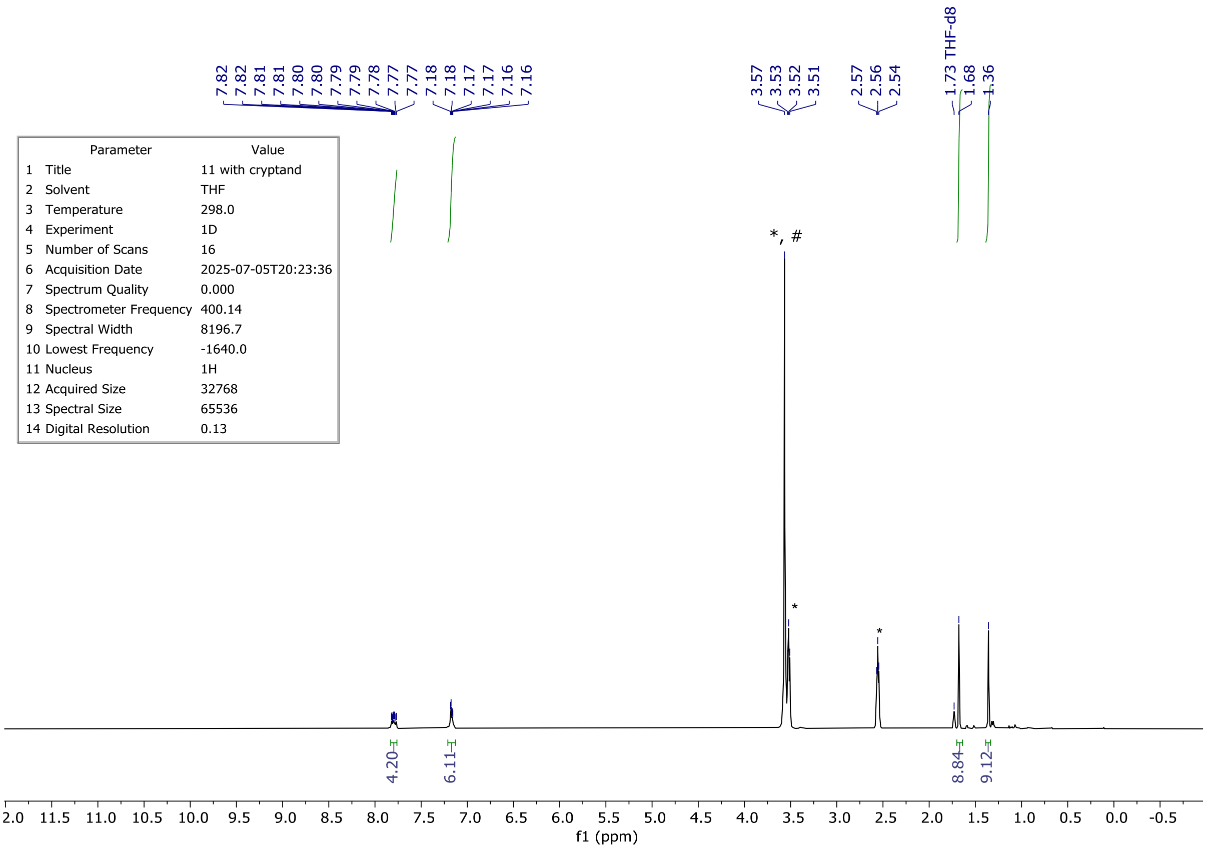

Figure S50 ^{1}H NMR spectrum of compound **11** in THF-d_8 after adding [2.2.2]-cryptand. * corresponds to the [2.2.2]-cryptand, # corresponds to the 18-c-6.

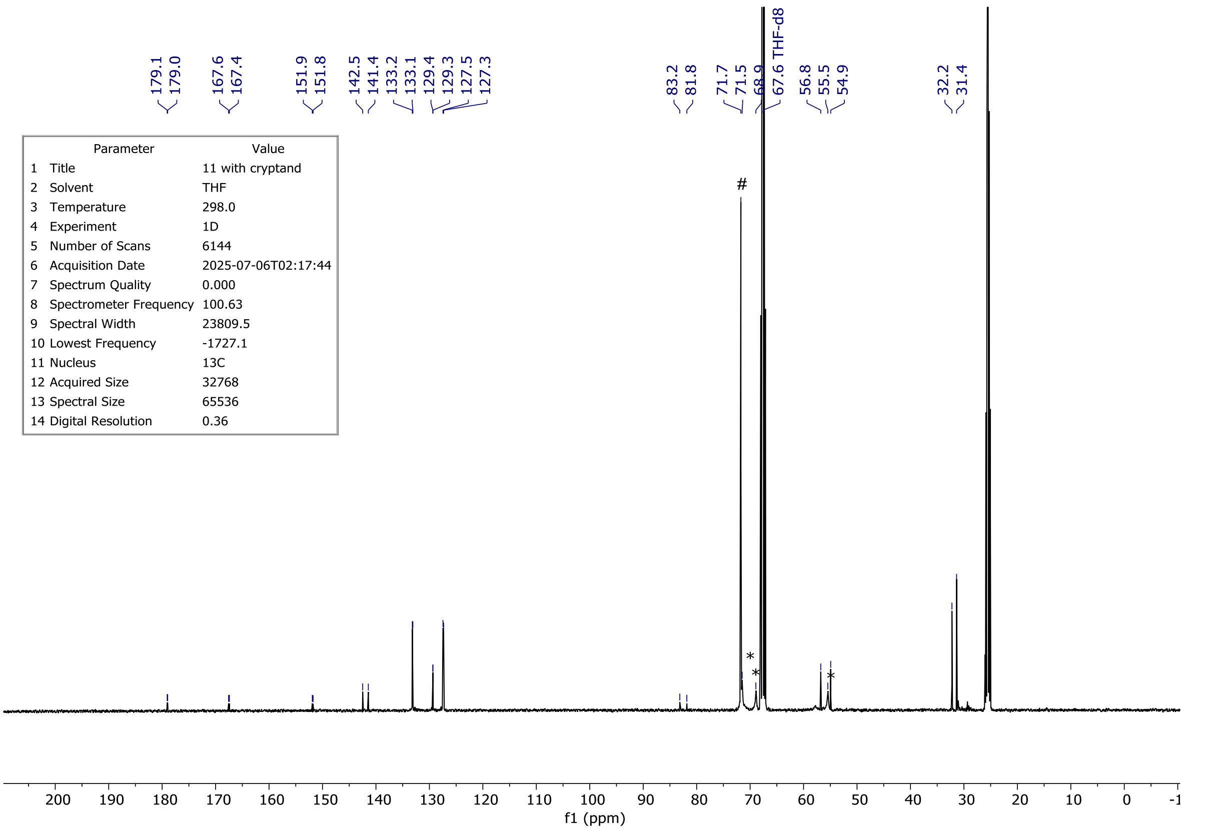

Figure S51 $^{13}C\{^{1}H\}$ NMR spectrum of compound **11** in THF-d_8 after adding [2.2.2]-cryptand. * corresponds to the [2.2.2]-cryptand, # corresponds to the 18-c-6.

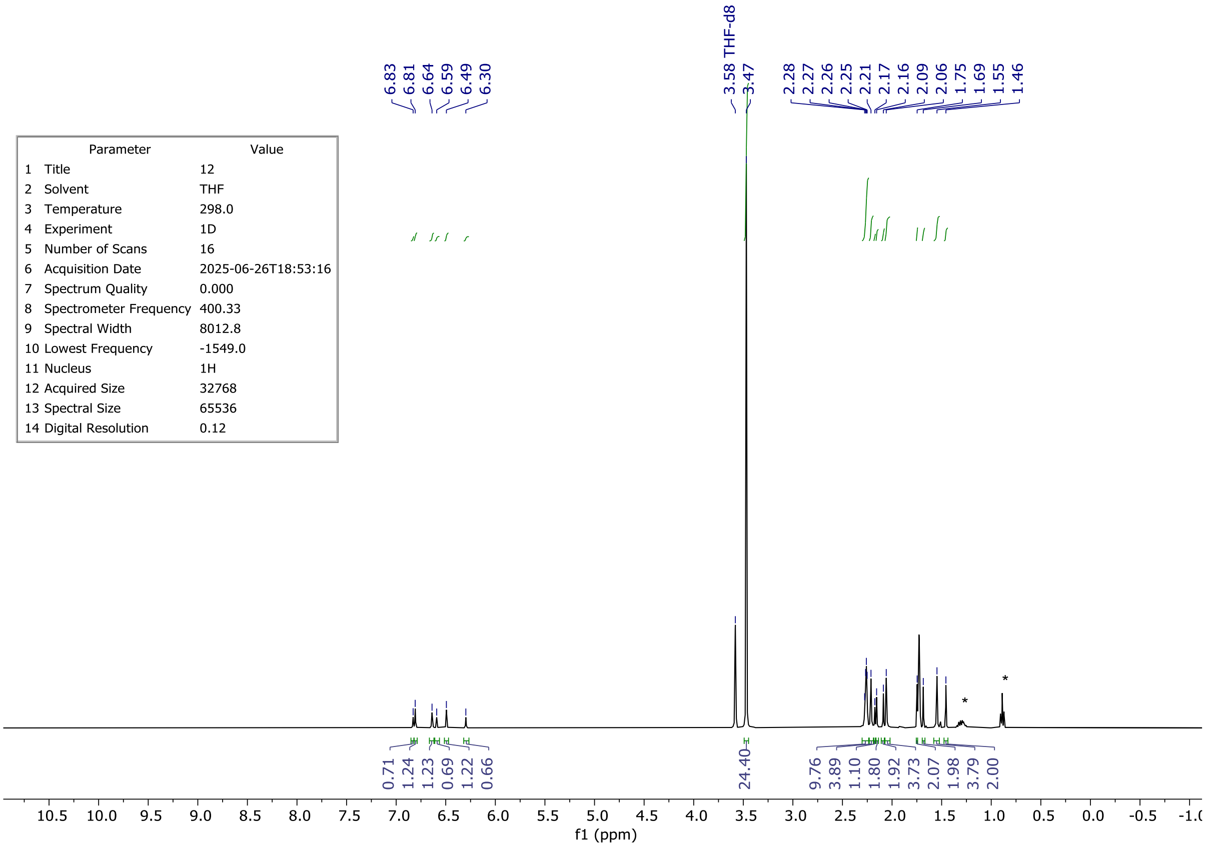

Figure S52. ^{1}H NMR spectrum of compound **12** in THF-d_8. * corresponds to pentane.

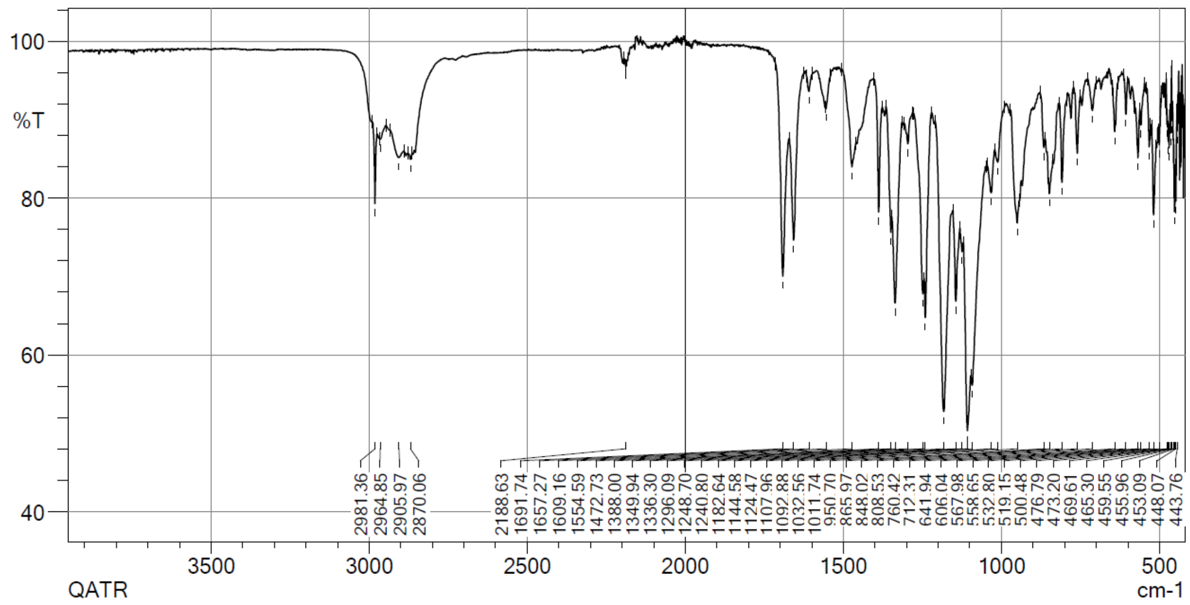

Figure S53 IR spectrum of compound **12** (solid state).

3. Real-Time IR Spectroscopy

3.1. General Procedure

A two neck Schlenk tube is fitted with an IR SiComp probe. The depth is adjusted using a Teflon QuickFit adapter. The background is recorded and a 12 mL solution of the starting material in the appropriate solvent is introduced via the side arm. Acquisition is initiated in the software controlling the ReactIR spectrometer and spectra were recorded every 15 s. After a few spectra were recorded, an appropriate reactant was added and the reaction was monitored.

3.2. Monitoring of the formation of 6^{CN}

The reaction between $\mathbf{1^{CN}}$ and **6-DAC** in THF was monitored using real-time *in situ* IR spectroscopy (Figure S51 and S52). To this end, compound $\mathbf{1^{CN}}$ (100 mg, 0.295 mmol) and 18-c-6 (78 mg, 0.295 mmol) were dissolved in THF, the mixture was cooled to 0°C and the acquisition started. $\mathbf{1^{CN}}$ is characterized by an intense band at 2015 cm^{-1}. After adding a THF solution of **6-DAC** (1 equiv.) to the THF solution of $\mathbf{1^{CN}}$ at 0 °C, the formation of a new species was observed featuring bands at 1676 and 2114 cm^{-1} (orange line) corresponding to the carbonyl and the cyano group, respectively. This species – which we assumed to be the anionic NHO $\mathbf{4^{CN}}$ – was unstable at 0 °C and converted into product $\mathbf{6^{CN}}$ upon heating the mixture to room temperature characterized by new bands at 1560 and 2164 cm^{-1} (green line). Notably, the band at 1676 cm^{-1} in $\mathbf{4^{CN}}$ did not decrease during the formation of $\mathbf{6^{CN}}$ due to the overlap of this signal with a band also present in $\mathbf{6^{CN}}$. Similarly, the cyano bands (2114 cm^{-1} for $\mathbf{4^{CN}}$ and 2114 and 2164 cm^{-1} for $\mathbf{6^{CN}}$) exhibited the same behavior. The observed cyano bands for $\mathbf{6^{CN}}$ were consistent with the IR spectrum recorded from crystals of $\mathbf{6^{CN}}$ (see Figure S26). The formation of multiple cyano bands for $\mathbf{6^{CN}}$ may be attributed to different coordination modes of the K^+ cation, involving an equilibrium between coordination to the nitrogen of the cyano group and the oxygen of a carbonyl group.

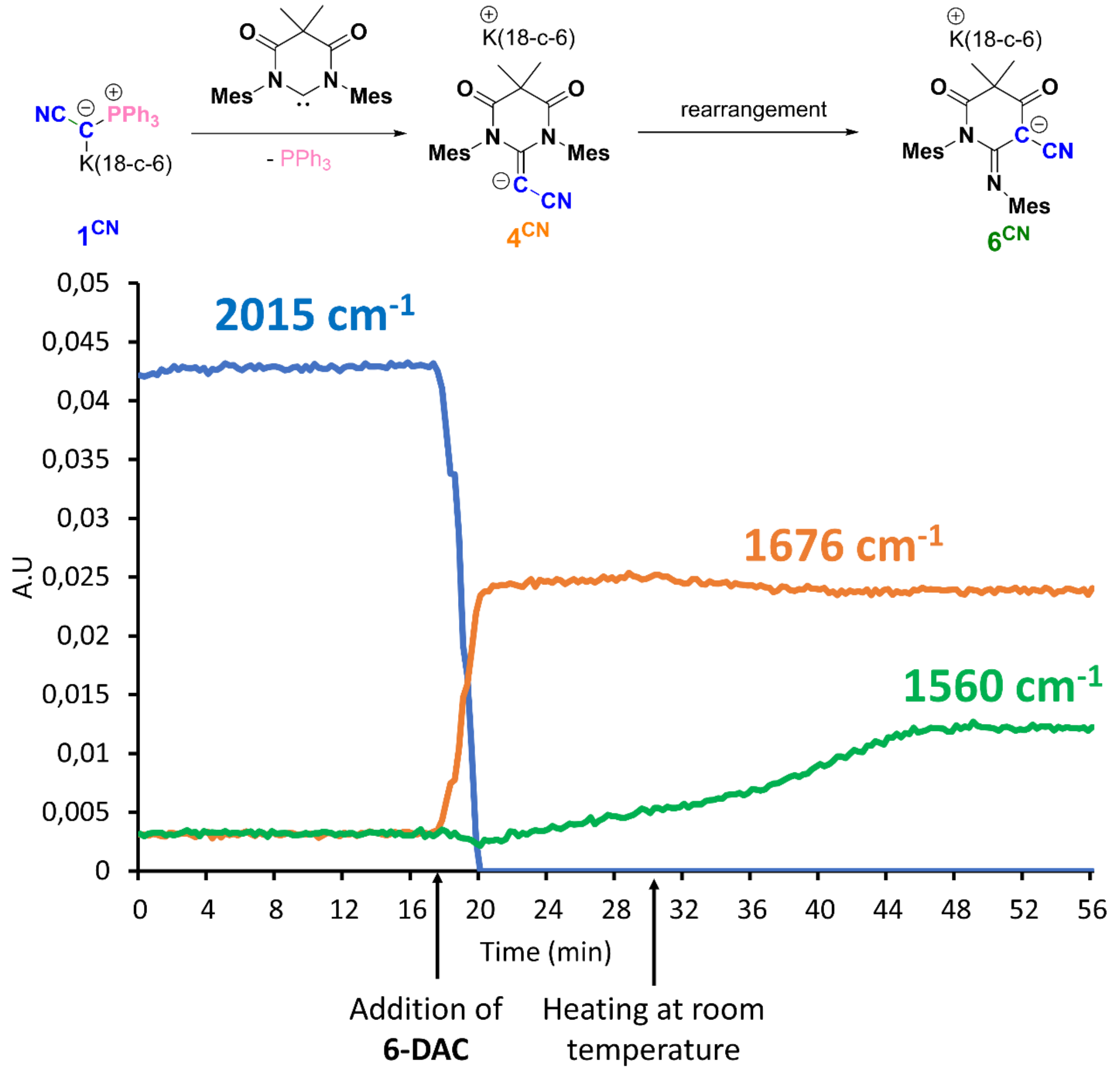

Figure S54: Kinetic plots of the formation of $\mathbf{6^{CN}}$.

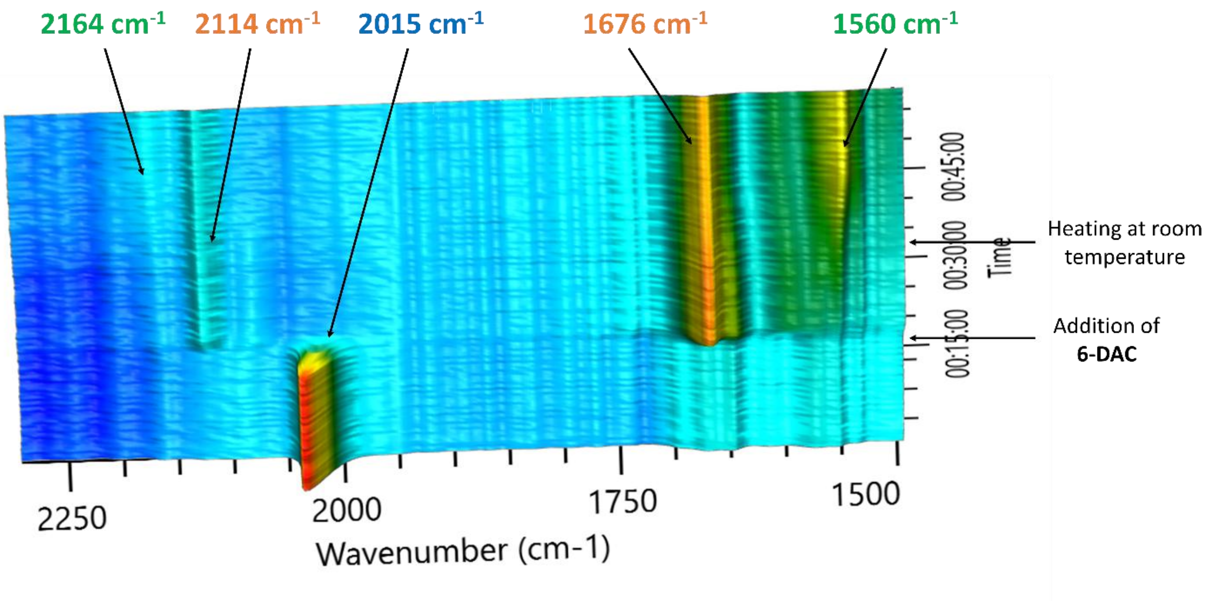

Figure S55: Top view of the surface plot of the formation of $\mathbf{6^{CN}}$.

4. Crystal structure determination

4.1. General Information

High-quality single crystals of appropriate dimensions were placed in an inert oil such as perfluoropolyalkylether, hand-picked under polarized optical microscopy and then mounted on the diffractometer. The data collection was done at 100 K. X-ray intensity data measurements of all compounds were carried out on an Oxford SuperNova diffractometer with graphite-monochromatized (CuK_{α}= 1.54184 Å) radiation. The X-ray generator was operated at 50 kV and 30 mA.

All structures were solved by intrinsic phasing and refined by the full-matrix least-squares on F^2 using software package and expanded using Fourier techniques.[6–10] Non-hydrogen atoms were refined anisotropically, while all hydrogen atoms were placed on the ideal positions using riding models and refined isotropically with displacement parameters constrained to those of the parent atoms (1.5 times Ueq for methyl groups and 1.2 times Ueq for all other carbon bound H).

Data collection and structure refinement details for all compounds are given in the following tables. Further details on the structure refinement are provided in the following sections for each individual structure.

Crystallographic data including structure factors have been deposited with the Cambridge Crystallographic Data Centre as supplementary publication no. CCDC - 2473675 to CCDC - 2473685 Copies of the data can be gained free of charge on application to Cambridge Crystallographic Data Centre, 12 Union Road, Cambridge CB2 1EZ, UK; [fax: (+44) 1223-336-033; email: deposit@ccdc.cam.ac.uk].

Table S1 Data collection and structure refinement details for compounds **3^{PO}, 4^{PO}** and **4^{PO}** with THF.

**Compound**	**3^{PO}**	**4^{PO}**	**4^{PO}** with THF
Formula	$C_{40}H_{60}KN_2O_{10}P$	$C_{53}H_{70}KN_2O_{10}P$	$C_{41}H_{46}KN_2O_4P$
CCDC	2473675	2473678	2473677
Formula weight	798.97	965.18	700.87
Temperature [K]	100(2)	100(2)	100(2)
Wave lenght [Å]	1.54184	1.54184	1.54184
Crystal system	Monoclinic	Triclinic	Monoclinic
Space group	*I*2/a	*P-1*	*P21/n*
a [Å]	19.0740(2)	13.9168(10)	10.13640(10)
b [Å]	9.85380(10)	14.1864(10)	23.0936(2)
c [Å]	23.4572(3)	15.5667(6)	15.8344(2)
α [°]	90	94.737(4)	90
β [°]	110.0010(10)	100.983(5)	90.4170(10)
γ [°]	90	119.160(7)	90
Volumen [$Å^3$]	4142.90(8)	2578.9(3)	3706.51(7)
Z	4	2	4
Calc. density [$Mg \cdot m^{-3}$]	1.281	1.243	1.256
μ ($Mo_{K\alpha}$) [mm^{-1}]	1.960	1.666	2.003
F(000)	1712	1032	1488
Crystal dimensions [mm]	0.140 x 0.060 x 0.050	0.320 x 0.220 x 0.190	0.150 x 0.090 x 0.060
Theta range θ [°]	4.011 to 76.753	2.954 to 68.000	3.384 to 67.964
Index ranges	-23<=h<=23	-14<=h<=16	-12<=h<=12
	-11<=k<=12,	-17<=k<=16	-17 <= k< = 27
	-29<=l<=29	-18<=l<=18	-19<=l<=19
Reflections collected	14058	29170	44530
Independent reflections	6384 [R(int) = 0.0267]	9244 [R(int) = 0.0677]	6743 [Rint = 0.0419]
Data/Restraints/Parameter	6384 / 2 / 493	9244 / 22 / 613	6743 / 0 / 450
Goodness-of-fit on F^2	1.035	1.169	1.081
Final R indices [I>2sigma(I)]	R1 = 0.0321, wR2 = 0.0847	R1 = 0.0636, wR2 = 0.1867	R1 = 0.0444, wR2 = 0.1122
Largest diff. peak and hole [e·Å-3]	0.240 and -0.228	0.610 and -0.917	0.459 and -0.246

Table S2 Data collection and structure refinement details for compounds **4**^{CN}, **5**^{CN} and **5**^{Tos}.

**Compound**	**4**^{CN}	**5**^{CN}	**5**^{Tos}
Formula	$C_{42}H_{60}KN_3O_9$	$C_{25}H_{42}KN3O_8$	$C_{31}H_{49}KN_2O_{10}S$
CCDC	2473676	2473679	2473680
Formula weight	790.03	551.71	680.88
Temperature [K]	100(2)	100(2)	100(2)
Wave lenght [Å]	1.54184	1.54184	1.54184
Crystal system	Triclinic	Monoclinic	Orthorhombic
Space group	*P-1*	$P2_1/c$	$P2_12_12_1$
a [Å]	13.0217(5)	21.8699(3)	8.33690(10)
b [Å]	13.1895(4)	8.91370(10)	19.1128(2)
c [Å]	13.6076(6)	31.1649(3)	22.5775(2)
α [°]	84.434(3)	90	90
β [°]	68.581(4)	106.9570(10)	90
γ [°]	77.556(3)	90	90
Volume [$Å^3$]	2124.15(15)	5811.21(12)	3597.53(7)
Z	2	8	4
Calc. density [$Mg \cdot m^{-3}$]	1.235	1.261	1.257
μ ($Mo_{K\alpha}$) [mm^{-1}]	1.551	2.013	2.288
F(000)	848	2368	1456
Crystal dimensions [mm]	0.460 x 0.160 x 0.120	0.264 x 0.102 x 0.063	0.490 x 0.120 x 0.080
Theta range θ [°]	3.432 to 67.996	2.965 to 76.827	3.029 to 76.821
Index ranges	-15<=h<=14	-27<=h<=24	-10<=h<=7
	-15<=k<=15,	-11<=k<=10	-24<=k<=23
	-16<=l<=16	-38<=l<=37	-28<=l<=28
Reflections collected	21726	75311	48404
Independent reflections	7647 [R(int) = 0.0593]	11939 [R(int) = 0.0423]	7408 [R(int) = 0.0495]
Data/Restraints/Parameter	7647 / 0 / 504	11939 / 55 / 746	7408 / 213 / 577
Goodness-of-fit on F^2	1.082	1.063	1.056
Final R indices [I>2sigma(I)]	R1 = 0.0640, wR2 = 0.1756	R1 = 0.0440, wR2 = 0.1148	R1 = 0.0344, wR2 = 0.0837
Largest diff. peak and hole [e·Å-3]	0.777 and -0.502	0.481 and -0.266	0.278 and -0.354

Table S3 Data collection and structure refinement details for compounds **6^{CN}**, **7** and **8**.

**Compound**	**6^{CN}**	**7**	**8**
Formula	$C_{84}H_{120}K_2N_6O_{18}$	$C_{41}H_{47}N_2O_4P$	$C_{27}H_{31}N_3O_2$
CCDC	2473681	2473682	2473683
Formula weight	1580.05	662.77	429.55
Temperature [K]	100(2)	100(2)	100(2)
Wave lenght [Å]	1.54184	1.54184	1.54184
Crystal system	Monoclinic	Triclinic	Monoclinic
Space group	$P2_1$	*P-1*	$P\ 2_1/n$
a [Å]	13.73114(12)	8.5404(4)	16.2342(6)
b [Å]	18.95137(19)	12.7880(5)	8.3809(3)
c [Å]	16.69090(18)	17.6593(7)	17.3585(8)
α [°]	90	109.896(4)	90
β [°]	92.6464(8)	96.827(4)	100.631(4)
γ [°]	90	91.518(4)	90
Volume [Å^3]	4338.74(7)	1796.04(14)	2321.21(16)
Z	2	2	4
Calc. density [Mg·m^{-3}]	1.209	1.226	1.229
μ (MoK_{α}) [mm^{-1}]	1.518	1.020	0.616
F(000)	1696	708	920
Crystal dimensions [mm]	0.265 x 0.124 x 0.088	0.380 x 0.150 x 0.110	0.250 x 0.190 x 0.130
Theta range θ [°]	2.650 to 67.991	3.685 to 76.705	4.128 to 76.816
Index ranges	-16<=h<=13	-10 <= h <= 10	-19<=h<=20
	-22<=k<=22,	-15 <= k< = 16	-10<=k<=10
	-20<=l<=20	-21<=l<=21	-20<=l<=21
Reflections collected	54186	20399	24285
Independent reflections	15097 [R(int) = 0.0744]	6953 [Rint = 0.0467]	4701 [Rint = 0.0611]
Data/Restraints/Parameter	15097 / 31 / 1017	6953 / 60 / 487	4701 / 0 / 298
Goodness-of-fit on F^2	1.063	1.096	1.086
Final R indices [I>2sigma(I)]	R1 = 0.0797, wR2 = 0.1973	R1 = 0.0480, wR2 = 0.1295	R1 = 0.0656, wR2 = 0.1738
Largest diff. peak and hole [e·Å-3]	1.122 and -0.724	0.515 and -0.551	0.642 and -0.319

Table S4 Data collection and structure refinement details for compounds **10** and **12**.

**Compound**	**10**	**12**
Formula	$C_{27}H_{37}N_2O_3PSi$	$C_{47}H_{63}KN_6O_8$
CCDC	2473684	2473685
Formula weight	496.64	879.13
Temperature [K]	100(2)	100(2)
Wave lenght [Å]	1.54184	1.54184
Crystal system	Monoclinic	Triclinic
Space group	*P* 2_1/n	*P-1*
a [Å]	15.9704(6)	11.8779(6)
b [Å]	8.9257(3)	14.3173(6)
c [Å]	19.8306(9)	14.9732(6)
α [°]	90	67.931(4)
β [°]	108.112(5)	78.678(4)
γ [°]	90	80.959(4)
Volume [$Å^3$]	2686.7(2)	2304.09(19)
Z	4	2
Calc. density [$Mg\cdot m^{-3}$]	1.228	1.267
μ ($Mo_{K\alpha}$) [mm^{-1}]	1.572	1.487
F(000)	1064	940
Crystal dimensions [mm]	0.330 x 0.070 x 0.040	0.180 x 0.110 x 0.080
Theta range θ [°]	4.270 to 68.000	3.221 to 76.702
Index ranges	-14<=h<=19	-14<=h<=14
	-10<=k<=10,	-12<=k<=17
	-23<=l<=23	-18<=l<=18
Reflections collected	32636	27529
Independent reflections	4877 [Rint = 0.0352]	9137 [Rint = 0.0534]
Data/Restraints/Parameter	4877 / 0 / 316	9137 / 0 / 578
Goodness-of-fit on F^2	1.148	1.065
Final R indices [I>2sigma(I)]	R1 = 0.0659, wR2 = 0.1636	R1 = 0.0541, wR2 = 0.1421
Largest diff. peak and hole [e·Å-3]	1.304 and -0.673	0.808 and -0.474

4.2. Molecular Structure of 3^{PO}

All hydrogen atoms were placed on ideal positions.

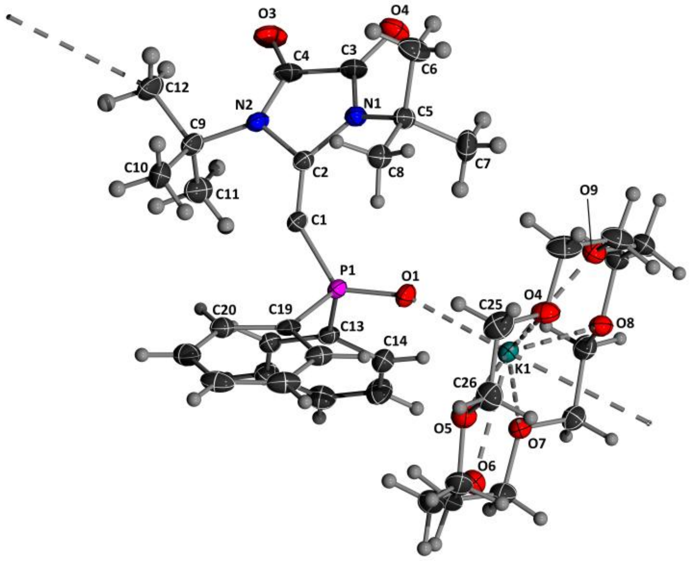

Figure S56 Molecular structure of compound $\mathbf{3^{PO}}$. Thermal ellipsoids at 50% probability level. Selected bond lengths [Å] and angles [°]: P1-C1 1.688(2), C1-C2 1.306(4), P1-O1 1.509(2), C2-N1 1.461(2), C2-N2 1.475(3), C3-O4 1.224(2), C4-O3 1.216(4) P1-C1-C2 144.2(2).

4.3. Molecular Structure of 4^{PO}

All hydrogen atoms were placed on ideal positions. The crystal structure contained a partly disordered 18-crown-6 ligand. The disorder was modelled using DELU restraints and EADP constraints and refined using the PART instructions and free variables, which were optimized to occupancies of 76% and 24%.

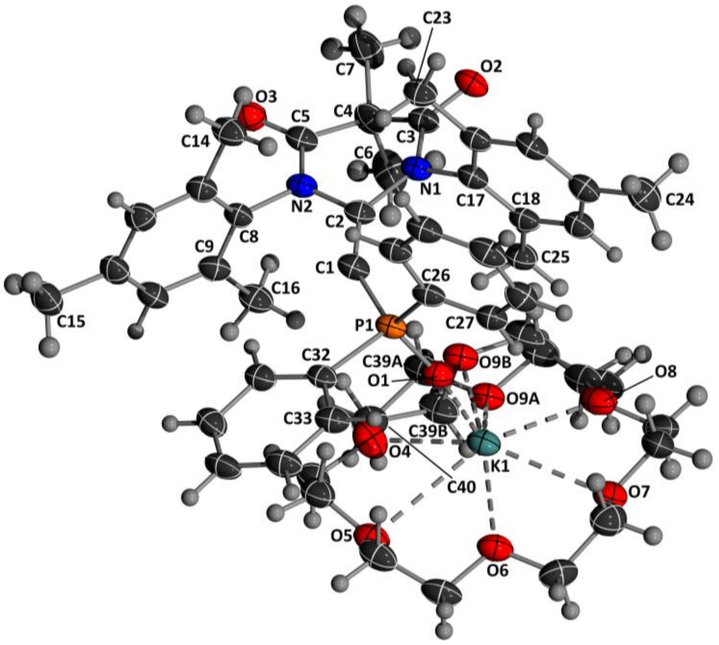

Figure S57 Molecular structure of compound $\mathbf{4^{PO}}$. Thermal ellipsoids at 50% probability level. Selected bond lengths [Å] and angles [°]: P1-C1 1.688(3), C1-C2 1.310(4), P1-O1 1.503(2), C2-N1 1.441(3), C2-N2 1.460(3), C3-O2 1.213(3), C5-O3 1.226(3) P1-C1-C2 148.0(2).

4.4. Molecular Structure of 4^{PO} with THF

All hydrogen atoms were placed on ideal positions.

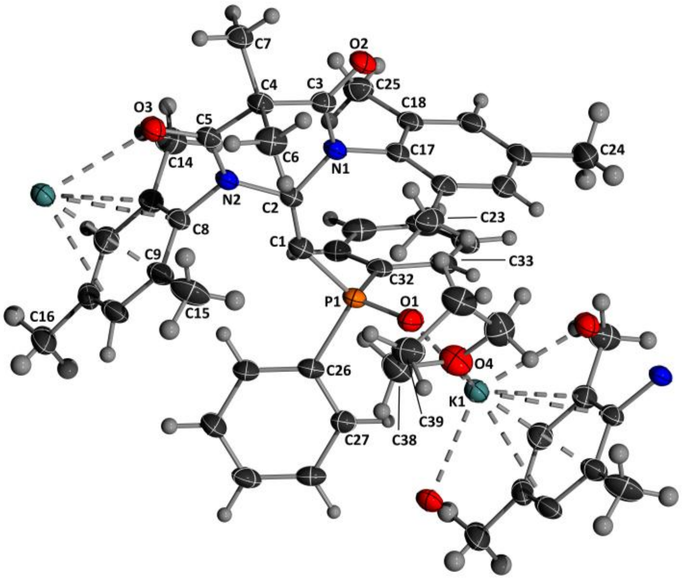

Figure S58 Molecular structure of compound $\mathbf{4^{PO}}$ with THF. Thermal ellipsoids at 50% probability level. Selected bond lengths [Å] and angles [°]: P1-C1 1.689(2), C1-C2 1.301(3), P1-O1 1.503(2), C2-N1 1.448(2), C2-N2 1.458(2), C3-O2 1.227(2), C5-O3 1.230(2) P1-C1-C2 146.7(2).

4.5. Molecular Structure of 4^{CN}

All hydrogen atoms were placed on ideal positions.

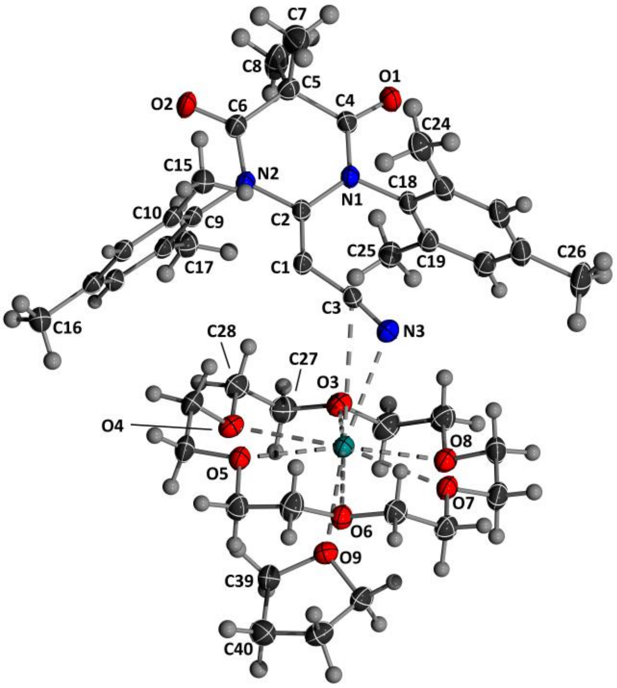

Figure S59 Molecular structure of compound $\mathbf{4^{CN}}$. Thermal ellipsoids at 50% probability level. Selected bond lengths [Å] and angles [°]:C1-C2 1.329(4), C1-C3 1.390(3), C3-N3 1.168(3), C2-N1 1.440(3), C2-N2 1.437(3), C4-O1 1.217(3), C6-O2 1.225(3) C2-C1-C3 127.8(2), C1-C3-N3 169.8(3).

4.6. Molecular Structure of $5^{PO'}$

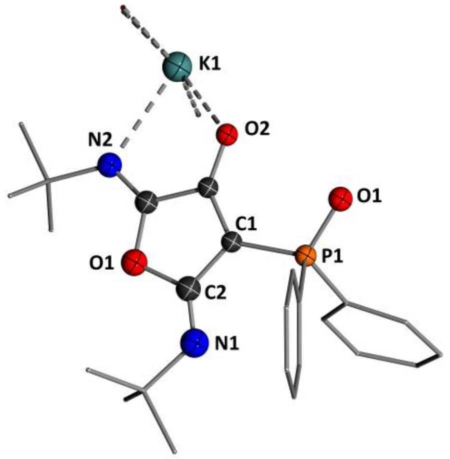

Figure S60 Molecular structure of compound $\mathbf{5^{PO'}}$. Thermal ellipsoids at 50% probability level. H atoms and other fragments are omitted for clarity. The bond lengths and bond angles are not discussed due to the low quality of the crystal.

4.7. Molecular Structure of 5^{CN}

All hydrogen atoms were placed on ideal positions. The crystal structure contained a disordered 18-crown-6 ligand. The disorder was modelled using EADP constraints and refined using the PART instructions and free variables, which were optimized to occupancies of 84% and 16%.

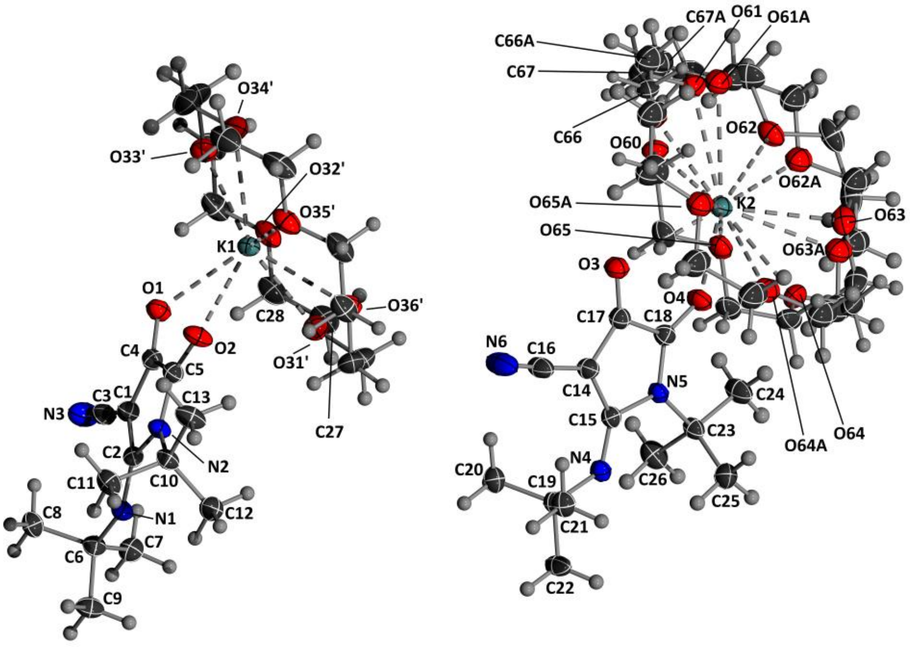

Figure S61 Molecular structure of compound $\mathbf{5^{CN}}$. Thermal ellipsoids at 50% probability level. Selected bond lengths [Å] and angles [°]: C1-C2 1.467(2), C1-C3 1.414(2), C3-N3 1.154(2), C2-N1 1.269(2), C2-N2 1.461(2), C4-O1 1.246(2), C5-O2 1.226(2) C2-C1-C3 129.1(1), C1-C3-N3 177.3(2). C14-C15 1.468(2), C14-C16 1.415(2), C16-N6 1.154(2), C15-N4 1.268(2), C15-N5 1.461(2), C17-O3 1.246(2), C18-O4 1.225(2) C15-C14-C16 129.2(1), C14-C16-N6 176.7(2).

4.8. Molecular Structure of 5^{Tos}

All hydrogen atoms were placed on ideal positions. The crystal structure contained a disordered 18-crown-6 ligand. The disorder was modelled using SIMU, DELU restraints and refined using the PART instructions and free variables, which were optimized to occupancies of 80% and 20%.

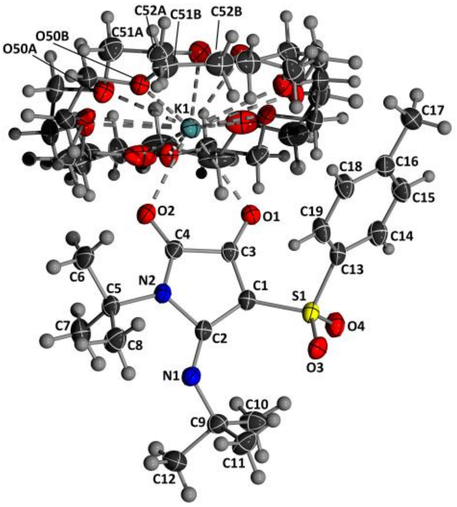

Figure S62 Molecular structure of compound **5^{Tos}**. Thermal ellipsoids at 50% probability level. Selected bond lengths [Å] and angles [°]: C1-C2 1.478(3), C1-S1 1.717(3), C2-N1 1.262(3), C2-N2 1.476(3), C3-O1 1.245(3), C4-O2 1.223(3) C2-C1-S1 128.5(2), C1-C2-N1 139.4(2).

4.9. Molecular Structure of 6^{CN}

All hydrogen atoms were placed on ideal positions. The crystal structure contained a disordered THF. The disorder was modelled using EADP constraints and refined using the PART instructions and free variables, which were optimized to occupancies of 56% and 44%.

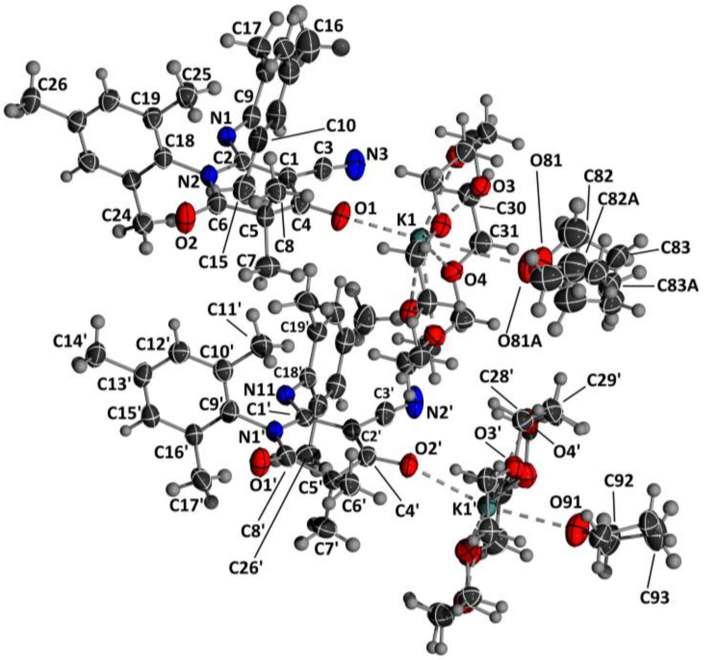

Figure S63 Molecular structure of compound **6^{CN}**. Thermal ellipsoids at 50% probability level. Selected bond lengths [Å] and angles [°]:C1-C2 1.448(7), C1-C3 1.424(8), C3-N3 1.158(8), C2-N1 1.277(8), C2-N2 1.438(6), C4-O1 1.258(7), C6-O2 1.235(7), C2-C1-C3 120.7(5), C1-C3-N3 172.5(6), C1'-C2' 1.455(7), C2'-C3' 1.428(8), C3'-N2' 1.155(8), C1'-N11 1.262(7), C1'-N1' 1.443(7), C4'-O2' 1.270(7), C8'-O1' 1.235(7), C1'-C2'-C3' 119.7(5), C2-C3-N3 175.8(6).

4.10. Molecular Structure of 7

All hydrogen atoms were placed on ideal positions. The crystal structure contained a disordered THF. The disorder was modelled using ISOR restraints and refined using the PART instructions and free variables, which were optimized to occupancies of 73% and 27%.

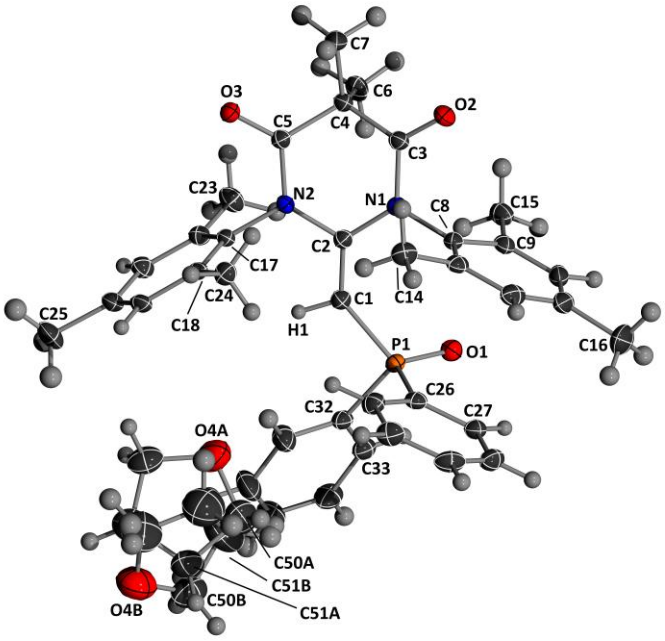

Figure S64 Molecular structure of compound **7**. Thermal ellipsoids at 50% probability level. Selected bond lengths [Å] and angles [°]: P1-C1 1.791(2), C1-C2 1.345(2), P1-O1 1.480(1), C2-N1 1.405(2), C2-N2 1.420(2), C3-O2 1.210(2), C5-O3 1.215(2) P1-C1-C2 138.2(1).

4.11. Molecular Structure of 8

All hydrogen atoms were placed on ideal positions.

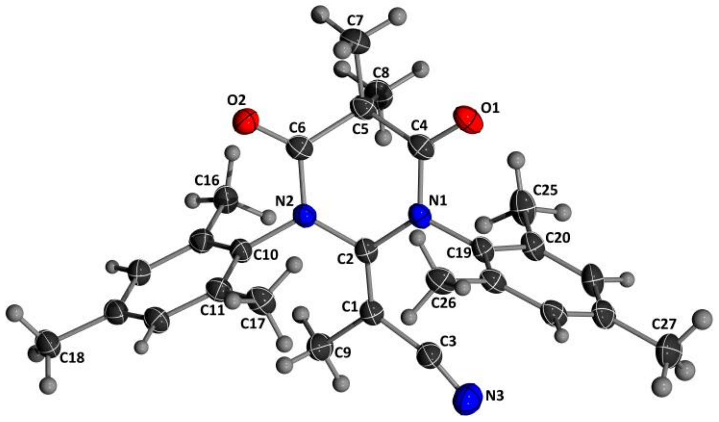

Figure S65 Molecular structure of compound **8**. Thermal ellipsoids at 50% probability level. Selected bond lengths [Å] and angles [°]:C1-C2 1.358(3), C1-C3 1.439(3), C2-C9 1.516(3), C3-N3 1.161(4), C2-N1 1.414(3), C2-N2 1.416(3), C4-O1 1.211(3), C6-O2 1.221(3) C2-C1-C3 124.6(2), C1-C3-N3 167.0(3).

4.12. Molecular Structure of 9

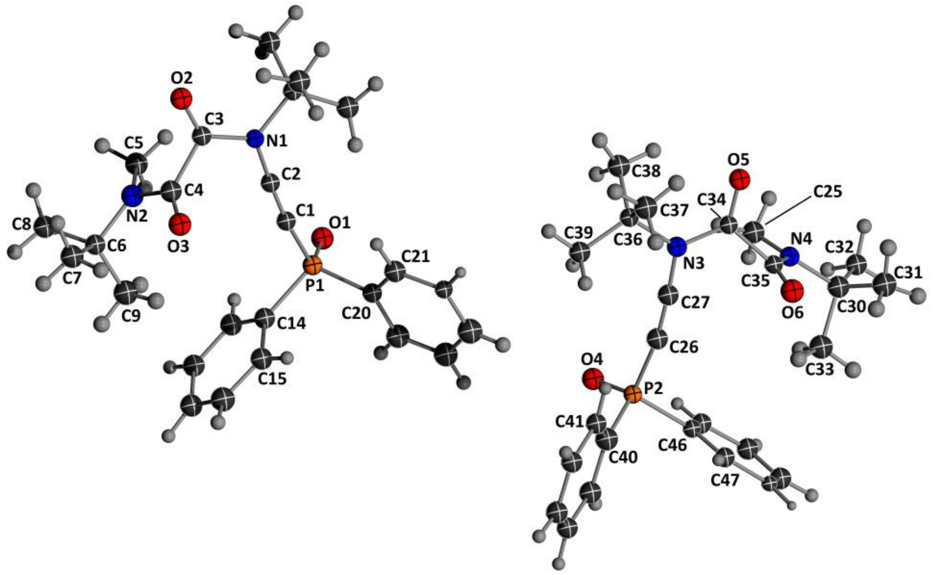

Figure S66 Molecular structure of compound **9**. Thermal ellipsoids at 50% probability level. The bond lengths and bond angles are not discussed due to the low quality of the crystal.

4.13. Molecular Structure of 10

All hydrogen atoms were placed on ideal positions.

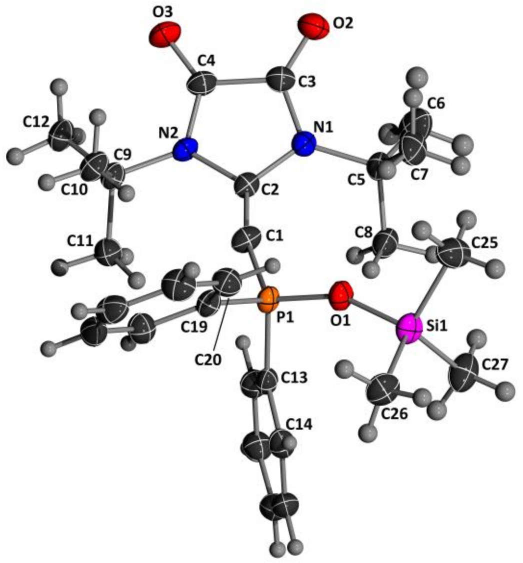

Figure S67 Molecular structure of compound **10**. Thermal ellipsoids at 50% probability level. Selected bond lengths [Å] and angles [°]: P1-C1 1.624(3), C1-C2 1.313(4), P1-O1 1.591(2), Si1-O1 1.666(2), C2-N1 1.440(4), C2-N2 1.448(4), C3-O2 1.217(4), C4-O3 1.217(4) P1-C1-C2 163.9(3).

4.14. Molecular Structure of 12

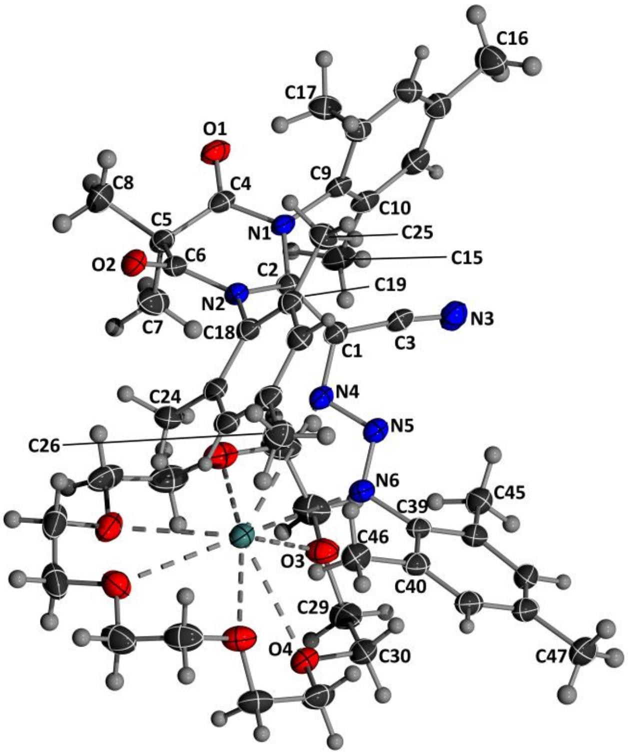

Figure S68 Molecular structure of compound **12**. Thermal ellipsoids at 50% probability level. Selected bond lengths [Å] and angles [°]:C1-C2 1.375(3), C1-C3 1.435(3), C3-N3 1.156(3), C2-N1 1.432(3), C2-N2 1.420(3), C1-N4 1.398(3), N4-N5 1.323(2), N5-N6 1.297(3), C4-O1 1.220(3), C6-O2 1.219(3) C2-C1-C3 122.8(2), C1-C3-N3 173.9(2), C1-N4-N5 111.7(2), N4-N5-N6 111.2(2).

5. Computational studies

5.1. General remarks

Computations were carried out using ORCA 6.0.1[11,12] and the standalone version of NBO 7.0.[13]

Structure optimizations employed the composite method r^{2}SCAN-3c[14] or the hybrid DFT functional PBE0[15,16] in conjunction with Grimme's dispersion correction D4[17,18] and the minimally augmented Karlsruhe basis set ma-def2-TZVPP[19,20] (notation r^{2}SCAN-3c ord PBE0-D4/ma-def2-TZVPP). All structures were fully optimized and confirmed as minima by frequency analyses. Partial charges were determined by Voronoi deformation density method[21] and/ or Natural Population analysis using the NBO program.

Please note that all computations were carried out for single, isolated molecules in the gas phase (ideal gas approximation). There may well be significant differences between gas phase and condensed phase.

5.2. Electronic structure of 3^{PO}

Starting from the single-crystal X-ray structure of **3^{PO}** (CCDC: 2473675), a pre-geometry optimisation of the free anion in the gas phase was first performed with GFN2-xTB[22], followed by a conformer search with CREST[23,24] and then CENSO.[25,26] The structure of the conformer with the lowest energy was optimised at the PBE0[27]-D4[17]/ma-def2-TZVPP[19,20] (convergence criteria: verytightopt, extremescf, integration grid 3) theory level and verified as a minimum on the potential energy surface (PES) using frequency analysis. Based on the DFT-optimised structure of **3^{PO}** with the corresponding wave function, the various parameters were calculated.

5.2.1. Kohn-Sham orbitals of 3^{PO}

5.2.2. NBO analysis

NBO analysis was performed on the electron density obtained from a single point calculation with the optimised geometry of the free anion in the gas phase (PBE0-D4/ma-def2-TZVPP, extremescf, defgrid3). The following section lists the decisive NBOs with their corresponding population numbers, coefficients and hybridisations.

```
    (Occupancy)   Bond orbital / Coefficients / Hybrids
 ------------------ Lewis ------------------------------------------------------

  35. (1.62008) LP ( 1) C  1           s(  9.99%)p 9.00( 89.84%)d 0.01(  0.10%)
                                                   f 0.01(  0.07%)
                                         0.0000  0.3159  0.0068  0.0030  0.0001
                                         0.2280  0.0017  0.0080 -0.0005 -0.5481
                                        -0.0188 -0.0009  0.0012  0.7382  0.0257
                                        -0.0004 -0.0019 -0.0046  0.0033 -0.0018
                                         0.0028  0.0227 -0.0011  0.0069 -0.0002
                                        -0.0204  0.0024 -0.0097 -0.0102  0.0033
                                         0.0144  0.0042 -0.0076  0.0152
```

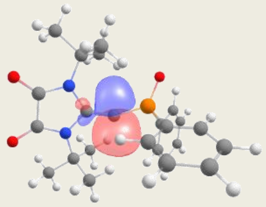

```
  45. (1.96605) BD ( 1) C  1- P  2
                ( 61.51%)   0.7843* C  1 s( 41.56%)p 1.40( 58.20%)d 0.00(  0.15%)
                                                  f 0.00(  0.09%)
                                         0.0000  0.6446  0.0062 -0.0098 -0.0003
                                         0.6586 -0.0046  0.0092 -0.0092  0.2151
```

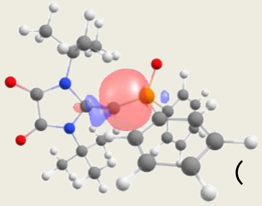

```
                                              -0.0003  0.0012  0.0023 -0.3188 -0.0081
                                              -0.0015 -0.0033  0.0166  0.0000 -0.0270
                                               0.0003 -0.0101  0.0004  0.0195  0.0005
                                              -0.0032 -0.0009  0.0120 -0.0090 -0.0029
                                              -0.0165 -0.0028 -0.0157 -0.0127
                ( 38.49%)   0.6204* P  2 s( 30.44%)p 2.26( 68.71%)d 0.03(  0.84%)
                                                        f 0.00(  0.02%)
                                               0.0000  0.0000  0.5516 -0.0103 -0.0002
                                              -0.0004  0.0000 -0.8094  0.0111 -0.0044
                                              -0.0022  0.0004  0.0000  0.0029 -0.0008
                                              -0.0031  0.0044 -0.0008  0.0000  0.1776
                                              -0.0136  0.0001 -0.0005  0.0004  0.0162
                                              -0.0004 -0.0003 -0.0360  0.0064  0.0000
                                               0.0093  0.0058  0.0008  0.0753 -0.0038
                                               0.0012 -0.0315  0.0021 -0.0006 -0.0033
                                               0.0050  0.0014  0.0060 -0.0011  0.0083
                                               0.0032
  46. (1.97839) BD ( 1) C  1- C 26
                ( 47.01%)   0.6856* C  1 s( 48.02%)p 1.08( 51.65%)d 0.01(  0.25%)
                                                         f 0.00(  0.08%)
```

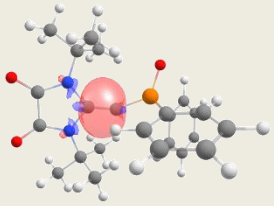

```
                                               0.0000  0.6924 -0.0249  0.0087  0.0001
                                              -0.7116  0.0238  0.0129 -0.0059  0.0944
                                              -0.0159 -0.0010 -0.0018 -0.0076  0.0116
                                               0.0039 -0.0017 -0.0094 -0.0019  0.0158
                                              -0.0002 -0.0013  0.0004  0.0390  0.0087
                                              -0.0232 -0.0053  0.0010  0.0168 -0.0009
                                              -0.0010 -0.0013  0.0217 -0.0036
                ( 52.99%)   0.7279* C 26 s( 45.32%)p 1.20( 54.58%)d 0.00(  0.04%)
                                                        f 0.00(  0.06%)
                                               0.0000  0.6731 -0.0099 -0.0047  0.0001
                                               0.7318  0.0012 -0.0050  0.0029  0.0348
                                               0.0016  0.0023 -0.0011  0.0945 -0.0006
                                               0.0011  0.0009 -0.0010  0.0009  0.0028
                                              -0.0034 -0.0010 -0.0026  0.0171  0.0060
                                              -0.0093 -0.0022 -0.0020 -0.0146 -0.0004
                                               0.0036  0.0002 -0.0190  0.0004
  47. (1.88314) BD ( 2) C  1- C 26
                ( 50.76%)   0.7125* C  1 s(  0.15%)p99.99( 99.66%)d 0.65(  0.10%)
                                                        f 0.56(  0.09%)
```

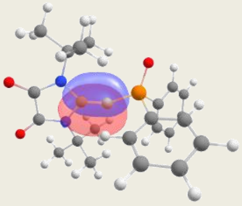

```
                                               0.0000  0.0386  0.0041  0.0057 -0.0001
                                              -0.0624 -0.0003  0.0042  0.0017 -0.8008
                                               0.0276  0.0065  0.0024 -0.5918  0.0190
                                               0.0048  0.0020  0.0052  0.0102  0.0067
                                              -0.0073  0.0103 -0.0008  0.0155 -0.0001
                                               0.0205 -0.0014  0.0086  0.0065  0.0057
                                              -0.0142  0.0023 -0.0013  0.0226
                ( 49.24%)   0.7017* C 26 s(  0.07%)p99.99( 99.87%)d 0.44(  0.03%)
                                                        f 0.54(  0.04%)
                                               0.0000  0.0256 -0.0046  0.0004 -0.0003
                                               0.0900 -0.0031  0.0007 -0.0047 -0.8066
                                               0.0024 -0.0039 -0.0011 -0.5830  0.0022
                                              -0.0022 -0.0006 -0.0139  0.0007 -0.0096
                                               0.0008 -0.0004  0.0007  0.0004  0.0001
                                              -0.0027 -0.0003 -0.0034 -0.0024  0.0065
                                              -0.0134 -0.0015 -0.0021  0.0110
 ---------------- non-Lewis ----------------------------------------------------
 116. (0.17361) BD*( 2) C  1- C 26
                ( 49.24%)   0.7017* C  1 s(  0.15%)p99.99( 99.66%)d 0.65(  0.10%)
                                                        f 0.56(  0.09%)
```

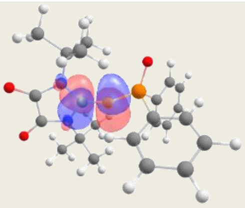

```
                                                0.0000  0.0386  0.0041  0.0057 -0.0001
                                               -0.0624 -0.0003  0.0042  0.0017 -0.8008
                                                0.0276  0.0065  0.0024 -0.5918  0.0190
                                                0.0048  0.0020  0.0052  0.0102  0.0067
                                               -0.0073  0.0103 -0.0008  0.0155 -0.0001
                                                0.0205 -0.0014  0.0086  0.0065  0.0057
                                               -0.0142  0.0023 -0.0013  0.0226
                 ( 50.76%)  -0.7125* C 26 s(  0.07%)p99.99( 99.87%)d 0.44(  0.03%)
                                                         f 0.54(  0.04%)
                                                0.0000  0.0256 -0.0046  0.0004 -0.0003
                                                0.0900 -0.0031  0.0007 -0.0047 -0.8066
                                                0.0024 -0.0039 -0.0011 -0.5830  0.0022
                                               -0.0022 -0.0006 -0.0139  0.0007 -0.0096
                                                0.0008 -0.0004  0.0007  0.0004  0.0001
                                               -0.0027 -0.0003 -0.0034 -0.0024  0.0065
                                               -0.0134 -0.0015 -0.0021  0.0110
117. (0.12922) BD*( 1) P  2- O  3
                 ( 73.96%)   0.8600* P  2 s( 25.35%)p 2.89( 73.15%)d 0.06(  1.49%)
                                                         f 0.00(  0.01%)
```

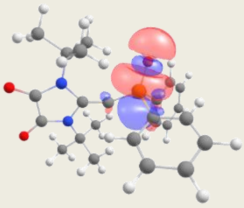

```
                                                0.0000  0.0000  0.5024 -0.0334 -0.0040
                                               -0.0017  0.0000  0.2266 -0.0101  0.0036
                                               -0.0050  0.0021  0.0000  0.6318 -0.0366
                                                0.0170 -0.0067  0.0025  0.0000 -0.5270
                                                0.0363 -0.0081  0.0039 -0.0020  0.0506
                                               -0.0125  0.0016 -0.0453  0.0093 -0.0004
                                               -0.0841  0.0264 -0.0025 -0.0450  0.0141
                                               -0.0023  0.0039 -0.0011  0.0007  0.0043
                                                0.0013  0.0034  0.0050 -0.0079  0.0051
                                                0.0020
                 ( 26.04%)  -0.5103* O  3 s( 37.64%)p 1.64( 61.81%)d 0.01(  0.54%)
                                                         f 0.00(  0.00%)
                                                0.0000  0.6135  0.0101 -0.0003 -0.0021
                                               -0.0001 -0.2426  0.0024 -0.0042 -0.0003
                                               -0.5826  0.0111 -0.0042 -0.0019  0.4686
                                               -0.0071  0.0041  0.0013  0.0294  0.0004
                                               -0.0239 -0.0004 -0.0559 -0.0008 -0.0283
                                               -0.0004  0.0026  0.0001 -0.0025 -0.0011
                                               -0.0023 -0.0035  0.0037 -0.0027 -0.0010
148. (0.08741) BD*( 1) C 26- N 29
                 ( 62.96%)   0.7934* C 26 s( 25.82%)p 2.87( 74.08%)d 0.00(  0.08%)
                                                         f 0.00(  0.02%)
```

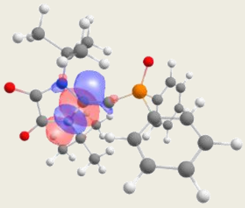

```
                                                0.0000  0.5081  0.0076  0.0025  0.0000
                                               -0.5163 -0.0076 -0.0007  0.0045 -0.4302
                                               -0.0037  0.0058  0.0022  0.5375 -0.0008
                                               -0.0087 -0.0037  0.0137 -0.0031 -0.0135
                                                0.0122 -0.0135 -0.0024  0.0051  0.0065
                                                0.0003 -0.0026 -0.0057 -0.0005 -0.0024
                                                0.0036  0.0097  0.0030  0.0089
                 ( 37.04%)  -0.6086* N 29 s( 31.45%)p 2.18( 68.41%)d 0.00(  0.13%)
                                                         f 0.00(  0.02%)
                                                0.0000  0.5607 -0.0068  0.0044 -0.0006
                                                0.0000  0.5671 -0.0028 -0.0064 -0.0008
                                                0.3724 -0.0017  0.0019 -0.0005 -0.4730
                                               -0.0025 -0.0072  0.0018  0.0173  0.0008
                                               -0.0169 -0.0027 -0.0228  0.0013  0.0087
                                               -0.0013  0.0096 -0.0017  0.0030  0.0003
                                                0.0047 -0.0024 -0.0094  0.0006 -0.0067
149. (0.09818) BD*( 1) C 26- N 30
                 ( 62.55%)   0.7909* C 26 s( 28.69%)p 2.48( 71.20%)d 0.00(  0.08%)
```

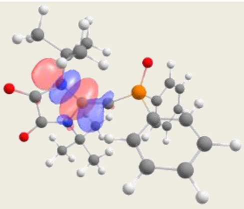

```
                                                 f 0.00(  0.03%)
                                        0.0000  0.5356  0.0080  0.0043 -0.0003
                                       -0.4343 -0.0073 -0.0033  0.0046  0.4028
                                       -0.0018 -0.0050 -0.0031 -0.6008  0.0019
                                        0.0052  0.0038 -0.0085  0.0103  0.0162
                                       -0.0061 -0.0125  0.0011  0.0049  0.0074
                                        0.0037 -0.0050  0.0062 -0.0056  0.0053
                                       -0.0061  0.0120  0.0014 -0.0047
                ( 37.45%)  -0.6120* N 30 s( 31.65%)p 2.15( 68.20%)d 0.00(  0.14%)
                                                 f 0.00(  0.02%)
                                        0.0000  0.5626  0.0026  0.0002 -0.0001
                                        0.5284 -0.0005 -0.0097 -0.0011 -0.3481
                                       -0.0036 -0.0060  0.0017  0.5305  0.0011
                                        0.0024 -0.0017 -0.0090 -0.0028  0.0217
                                        0.0015 -0.0280  0.0023  0.0027 -0.0001
                                        0.0037  0.0012 -0.0055  0.0056 -0.0056
                                        0.0023 -0.0074 -0.0021  0.0030
```

5.2.3. Second order perturbation analysis

Table S5. Results of second order perturbation theory analysis of fock matrix in NBO basis analysis of the free anion (**3^{PO}**) at PBE0-D4/ma-def2-TZVPP level of theory. Only the most significant delocalizations were listed.

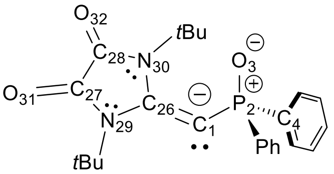

	$E^{(2)}$/ kcal mol^{-1}
LP(C_1) → σ*(P_2-O_3)	25.49
LP(C_1) → σ*(C_{26}-N_{29})	23.18
LP(C_1) → σ*(C_{26}-N_{30})	34.29
LP(C_1) → σ*(P_2-C_4)	12.62
LP(N_{29}) → π*(C_1- C_{26})	27.42
LP(N_{29}) → π*(C_{27}- C_{31})	75.13
LP(N_{30}) → π*(C_1- O_{26})	29.54
LP(N_{30}) → π*(C_{28}- O_{32})	74.08

5.2.4. Natural resonance theory analysis

NRT analysis[28] was performed with NBO7.0 program package[13] implemented in ORCA6.0.1 at PBE0-D4/ma-def2-TZVPP level of theory with restricted NRT averaging to atoms: C1, P2, O3, C4, C15, C26, C27, C28, N29, N30, O31, O32, C33, C46. Only resonance structures accounting for approximately 65% of total resonance weight were displayed (Figure S70).

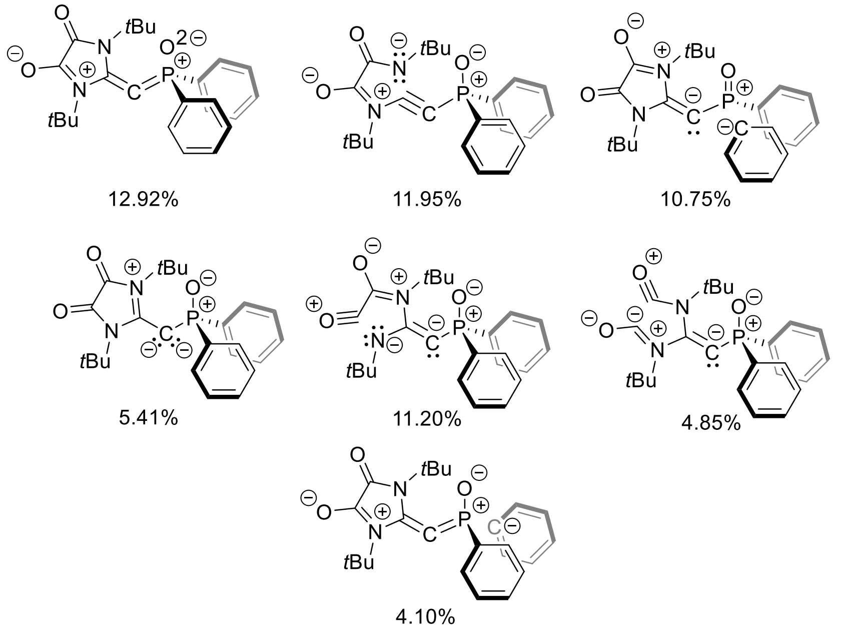

Figure S70. Leading resonance structures accounting for ca. 61% of total resonance weight obtained from NRT analysis of **3^{PO}**. Symmetry equivalent resonance structures were merged and weighted summed up.

In the following, all canonical Lewis structures of the NRT analysis are listed, which are derived from the one with the highest resonance weight (7.09%).

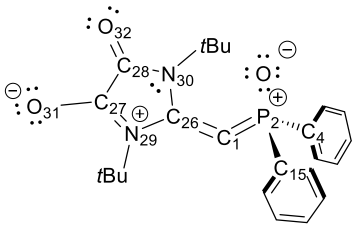

RS	Resonance Weight(%)	Added(Removed)
1	7.09	
2	6.51	(C 1- P 2), C 1- C 26, P 2- O 3, (C 26- N 30), (O 3), N 30
3	6.08	(C 1- P 2), P 2- O 3, P 2- O 3, (P 2- C 15), (C 27- N 29), C 27- O 31, C 28- N 30, (C 28- O 32), C 1, (O 3), (O 3), C 15, N 29, (N 30), (O 31), O 32
4	5.83	(C 27- N 29), C 27- O 31, C 28- N 30, (C 28- O 32), N 29, (N 30), (O 31), O 32
5	5.72	(C 1- P 2), P 2- O 3, (C 28- N 30), C 28- O 32, C 1, (O 3), N 30, (O 32)
6	5.48	(C 1- P 2), P 2- O 3, (C 27- N 29), (C 27- N 29), C 27- O 31, C 27- O 31, C 28- N 30, (C 28- O 32), C 1, (O 3), N 29, N 29, (N 30), (O 31), (O 31), O 32
7	5.44	(C 1- P 2), C 1- C 26, P 2- O 3, (C 26- N 29), (C 27- N 29), C 27- O 31, C 28- N 30, (C 28- O 32), (O 3), N 29, N 29, (N 30), (O 31), O 32
8	5.41	(C 1- P 2), (C 1- C 26), P 2- O 3, C 26- N 30, (C 27- N 29), C 27- O 31, C 1, C 1, (O 3), N 29, (N 30), (O 31)
9	4.85	(C 1- P 2), P 2- O 3, (C 27- C 28), C 28- O 32, C 1, (O 3), C 27, (O 32)
10	4.67	(C 1- P 2), P 2- O 3, P 2- O 3, (P 2- C 15), C 1, (O 3), (O 3), C 15
11	4.10	P 2- O 3, (P 2- C 4), (O 3), C 4
12	4.01	(C 1- P 2), P 2- O 3, (C 27- C 28), (C 27- N 29), C 27- O 31, C 27- O 31, C 28- N 30, (C 28- O 32), C 1, (O 3), C 28, N 29, (N 30), (O 31), (O 31), O 32
13	3.68	(C 1- P 2), (C 1- C 26), P 2- O 3, C 26- N 29, (C 27- N 29), C 27- O 31, C 1, C 1, (O 3), (O 31)
14	3.13	(C 1- P 2), P 2- O 3, P 2- O 3, (P 2- C 4), (C 27- N 29), C 27- O 31, C 28- N 30, (C 28- O 32), C 1, (O 3), (O 3), C 4, N 29, (N 30), (O 31), O 32
15	2.84	P 2- O 3, (P 2- C 4), (C 27- N 29), C 27- O 31, C 28- N 30, (C 28- O 32), (O 3), C 4, N 29, (N 30), (O 31), O 32
16	2.37	(C 1- P 2), (C 1- P 2), P 2- O 3, P 2- O 3, (C 27- N 29), C 27- O 31, C 28- N 30, (C 28- O 32), C 1, C 1, (O 3), (O 3), N 29, (N 30), (O 31), O 32
17	2.19	(C 1- C 26), P 2- O 3, (P 2- C 15), (C 27- N 29), C 27- O 31, C 1, (O 3), C 26, N 29, (O 31)
18	2.00	(C 1- P 2), P 2- O 3, (C 27- C 28), (C 27- N 29), C 27- O 31, C 27- O 31, C 1, (O 3), C 28, N 29, (O 31), (O 31)

```
   19      1.87   ( C 27- N 29),  C 27- O 31,  N 29, ( O 31)
   20      1.85   ( C  1- P  2), ( C  1- P  2),  P  2- O  3,  P  2- O  3,
                  ( C 27- N 29),  C 27- O 31,  C 28- N 30, ( C 28- O 32),
                   C  1,  C  1, ( O  3), ( O  3),  N 29, ( N 30), ( O 31),
                   O 32
   21      1.72   ( C  1- P  2),  P  2- O  3,  P  2- O  3, ( P  2- C  4),
                   C  1, ( O  3), ( O  3),  C  4
   22      1.67   ( C  1- P  2), ( C  1- P  2),  P  2- O  3,  P  2- O  3,
                   C  1,  C  1, ( O  3), ( O  3)
   23      1.64   ( C  1- P  2),  C  1- C 26,  P  2- O  3, ( C 26- N 29),
                  ( O  3),  N 29
   24      1.63   ( C  1- P  2),  C  1- C 26,  P  2- O  3, ( C 26- N 30),
                  ( C 27- N 29),  C 27- O 31,  C 28- N 30, ( C 28- O 32),
                  ( O  3),  N 29, ( O 31),  O 32
   25      1.41   ( C  1- P  2), ( C  1- C 26),  P  2- O  3,  C 26- N 30,
                  ( C 27- N 29),  C 27- O 31, ( C 28- O 32),  C  1,
                  ( O  3),  C 28,  N 29, ( N 30), ( O 31),  O 32
   26      1.40   ( C  1- P  2), ( C  1- C 26),  P  2- O  3,  C 26- N 29,
                  ( C 27- N 29),  C  1, ( O  3),  C 27
   27      1.28   ( C  1- P  2),  P  2- O  3, ( C 27- C 28), ( C 27- N 29),
                   C 27- O 31,  C 28- O 32,  C  1, ( O  3),  C 27,  N 29,
                  ( O 31), ( O 32)
   28      1.15   ( C  1- P  2), ( C  1- P  2),  P  2- O  3,  P  2- O  3,
                   C  1,  C  1, ( O  3), ( O  3)
   29      1.11   ( C  1- P  2), ( C  1- C 26),  P  2- O  3,  C 26- N 30,
                  ( C 27- N 29),  C 27- O 31, ( C 28- O 32),  C  1,
                  ( O  3),  C 28,  N 29, ( N 30), ( O 31),  O 32
   30      1.08   ( C  1- P  2), ( C  1- C 26),  P  2- O  3,  C 26- N 29,
                  ( C 27- N 29),  C  1, ( O  3),  C 27
   31      0.77   ( C  1- P  2),  P  2- O  3,  P  2- O  3, ( P  2- C 15),
                  ( C 27- N 29),  C 27- O 31,  C  1, ( O  3), ( O  3),
                   C 15,  N 29, ( O 31)
 others    0.01
---------------------------------------------------------------------------
        100.00   * Total *
```

5.2.5. Bond indices and charge analysis

To further describe the bonding situation in **3^{PO}**, various bonding indices and the results from the Natural Population Analysis (NPA charges) and Voronoi Deformation Density (VDD)[21] charges obtained for the individual atoms in the molecule are presented here. The latter method was chosen because of its lower dependency of the basis set, resulting in more realistic and chemically meaningful charges.[21] Mayer bond orders, Wiberg bond indices and Voronoi charges were calculated with Multiwfn3.7.[29,30]

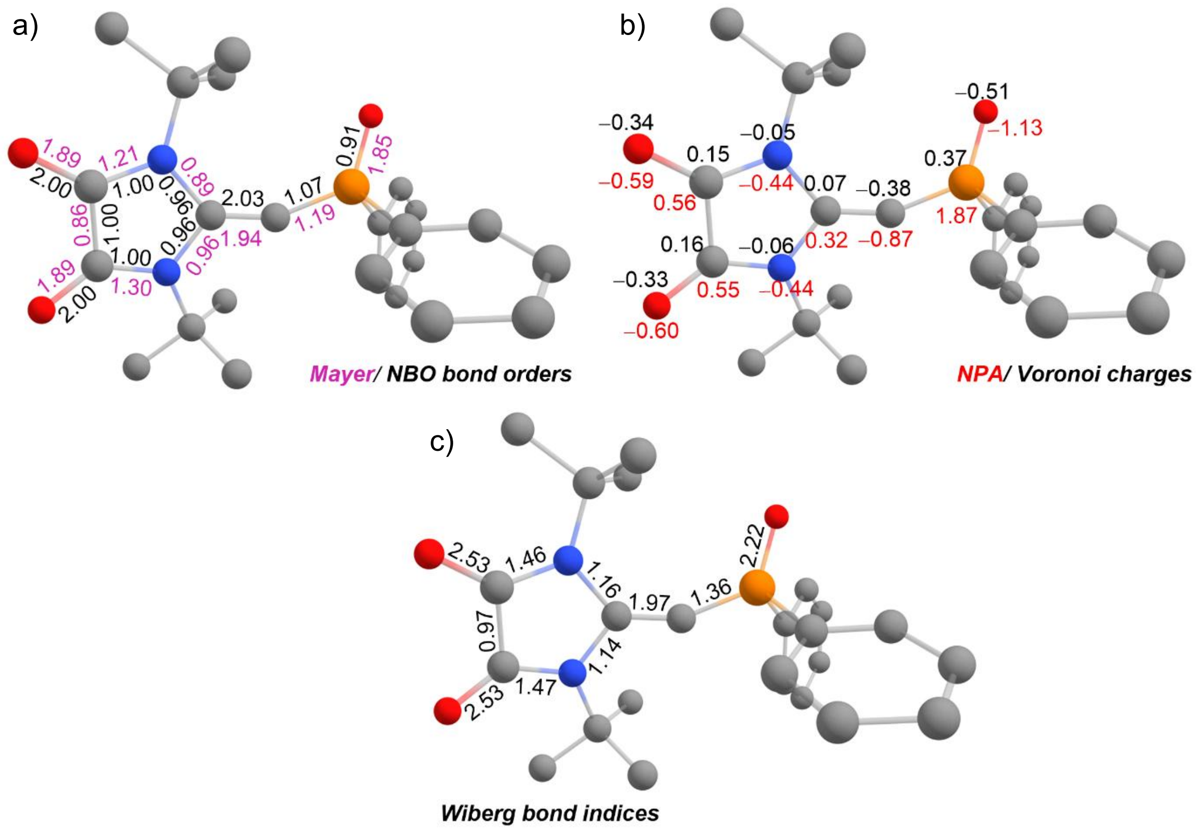

Figure S71. a) Mayer and NBO bond orders (PBE0-D4/ma-def2TZVPP), b) NPA and Voronoi[21] charges analysis and c) Wiberg bond indices of free anion of $\mathbf{3^{PO}}$.

Table S6. Selected atomic charges for anionic NHOs (***3*^{Z}**) with different Z-substituents derived from natural population analysis (NPA) at PBE0-D4/ma-def2-TZVPP level of theory.

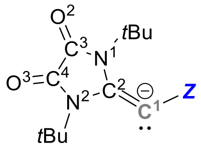

	NPA charge		
	P(O)Ph₂	*CN*	*Tos*
C^1	−0.89	−0.49	−0.79
C^2	0.32	0.30	0.34
N^1/N^2 [a]	0.44	−0.43	0.43
O^2/O^3 [a]	0.60	−0.60	0.59
Z[b]	0.03	−0.30	−0.09

[a] averaged values over both atoms; [b] sum over entire *Z* unit.

5.2.6. Electron localisation function

The Electron localisation function (ELF)[31] was calculated with Multiwfn3.7 based on the electron density obtained from single point calculation of the free anion of **3^PO^**.

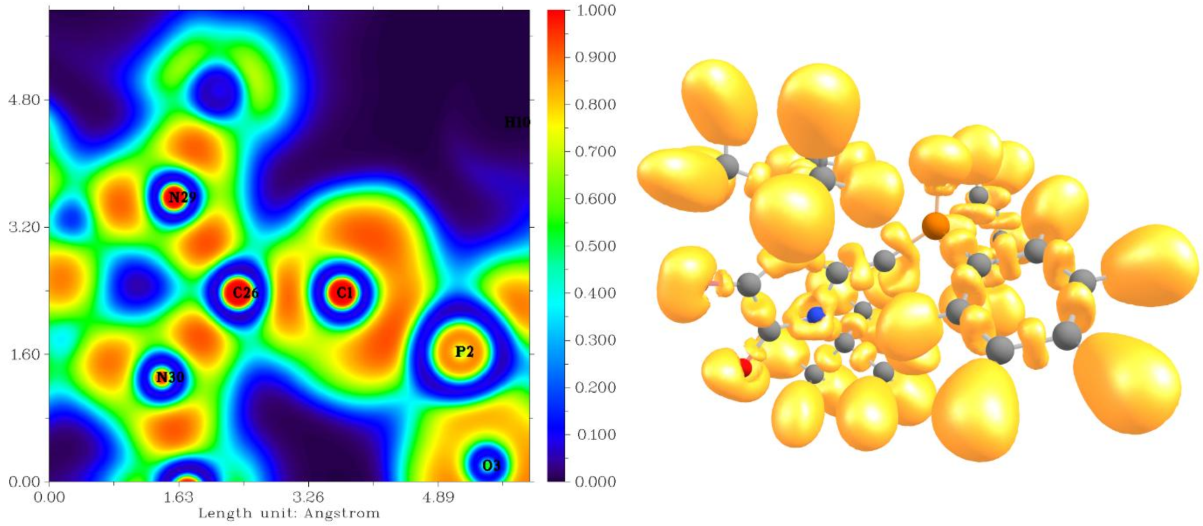

Figure S72. 2D-plot of ELF (left) and 3D-plot (right, isovalue = 0.8). Lone pair at carbon can clearly be seen from ELF maximum between P2-C1-C26 axis in the 2D plane.

5.3. Thermodynamics of carbene exchange

The thermodynamics of the ligand exchange reaction between the 18-crown-6-potassium cyanodiazomethanide[5] (**2^{CN}**) and five different carbenes were calculated. All calculation steps applied implicit solvation[1] models for the solvent THF. First the starting structures were pre-optimised on the GFN2-xTB level of theory using the xTB software package version 6.4.1[22,32] by a conformer search on the same level of theory using CREST[2] version 3.0.2.[23,24] The resulting conformer ensemble was further scanned with CENSO workflow (version 1.2.0)[26] including step 2, yielding geometries and electronic energies on the r^2SCAN-3c(SMD)[14,33] level of theory with thermostatistical contributions on the GFN2-xTB(ALPB) level of theory using the single point hessian approach. The resulting energetically lowest conformer was chosen for a geometry optimisation and frequency[3] analysis at the r^2SCAN-3c(CPCM)[34] with ORCA, to ensure that it is a local minimum on the potential energy surface (PES).
Structures of the corresponding 18-crown-6 potassium anionic *N*-heterocyclic olefin (aNHOs) salts were obtained as follows: First the aNHOs were optimised with GFN2-xTB(ALPB) level of theory. and a conformational search was carried out with CREST-CENSO (step 0 - step 2). The minimum energy conformer was used as guest structure for an ORCA-DOCKER(ALPB) run on GFN2-xTB(ALPB) level of theory with the aim to find the position of the K(18-c-6)-cation. Finally, the resulting conformer ensemble was used for an additional CENSO run (step 0 - step 2) and the minimum energy conformer was optimised (r^2SCAN-3c/CPCM(THF))[3] and verified as a local minimum on the (PES) with frequency analysis. The position of the cation was in most cases close to the cyanide group. For **3^{CN}**, the coordination to the two carbonyl oxygen atoms is favoured by 1.9 kcal mol^{-1} over coordination by the cyano group (Table S7).

[1] analytical linearized Poisson-Boltzmann (alpb)[42] for xTB and CREST, conductor-like polarizable continuum model for structure optimisations and frequency analysis with ORCA 6.0.1, respectively.
[2] CREST energy thershold: 10 kcal mol^{-1}
[3] Numerical integration grid set to defgrid3 and very tight optimization convergence VERYTIGHTOPT was chosen.

Table S7. Summary of calculated data for substitution reaction between potassium cyanodiazomethanide (**2**$^{\mathbf{CN}}$) and different carbenes, including electronic energies and thermal corrections. Optimisations and frequency analysis were all done at r²SCAN-3c(CPCM) level of theory.

**Compound**	**Code**	**PG**	E_{tot}**/ a.u.**[a]	U_{0K}**/ a.u.**	U_{298K}**/ a.u.**	**H/ a.u.**	**G/ a.u.**	$H_{corr.}$**/ a.u.**[b]	$G_{corr.}$**/ a.u.**[c]
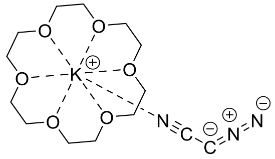	**2**$^{\mathbf{CN}}$	C_1	−1763.16362	0.38849657	−1762.74773	−1762.74679	−1762.82967	0.41683675	0.33395735
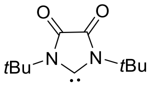	^{tBu}DAC	C_1	−689.796262	0.27766615	−689.502244	−689.5013	−689.559358	0.29496182	0.23690382
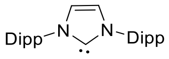	IDipp	C_1	−1159.74743	0.5651718	−1159.15195	−1159.15101	−1159.23777	0.59641977	0.5096593
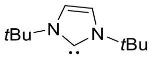	I*t*Bu	C_1	−540.541922	0.29274074	−540.234706	−540.233762	−540.287894	0.30815998	0.25402746
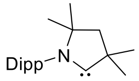	^{Dipp}CAAC	C_1	−835.285087	0.46288481	−834.799365	−834.798421	−834.869059	0.30815998	0.25402746
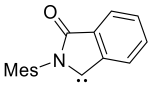	^{Mes}ArAmC	C_1	−786.604361	0.27081656	−786.316557	−786.315613	−786.376254	0.48666572	0.41602731
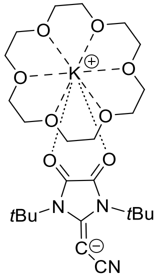	**3**$^{\mathbf{CN}}$	C_1	−2343.56193	0.6618866	−2342.8584	−2342.85745	−2342.96679	0.70448017	0.59514271

Compound	Code	PG	E_{tot}/ a.u.[a]	U_{0K}/ a.u.	U_{298K}/ a.u.	H/ a.u.	G/ a.u.	$H_{corr.}$/ a.u.[b]	$G_{corr.}$/ a.u.[c]
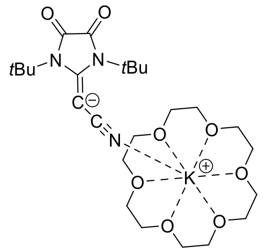	**3**^{CN}	C_1	−2343.55703	0.66123164	−2342.85383	−2342.85288	−2342.96374	0.7041508	0.59329359
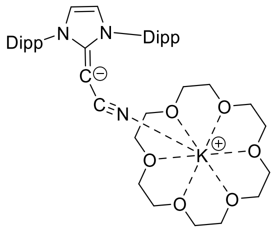		C_1	−2813.48816	0.94686658	−2812.48507	−2812.48413	−2812.62183	1.0040366	0.8663319
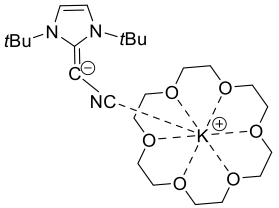		C_1	−2194.2577	0.67413422	−2193.54298	−2193.54203	−2193.64979	0.71567065	0.60791492
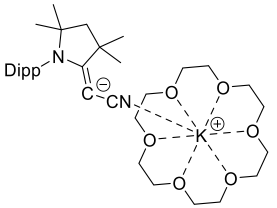		C_1	−2489.05006	0.84529199	−2488.15607	−2488.15512	−2488.27775	0.89494146	0.77231283

Compound	Code	PG	E_{tot}/ a.u.[a]	U_{0K}/ a.u.	U_{298K}/ a.u.	H/ a.u.	G/ a.u.	$H_{corr.}$/ a.u.[b]	$G_{corr.}$/ a.u.[c]
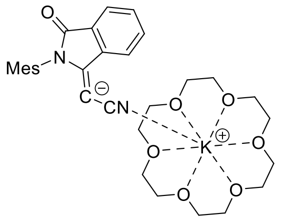		C_1	−2440.41228	0.6547028	−2439.71511	−2439.71416	−2439.82596	0.69812213	0.5863249
N_2		$D_{\infty h}$	−109.507499	0.00557822	−109.49956	−109.498616	−109.521005	0.00888297	−0.01350649

[a] Total SCF energy in a.u.; [b] thermal correction to Gibbs energy in a.u. (298 K unless stated otherwise); [c] thermal correction to Gibbs energy in a.u. (298 K unless stated otherwise)

Table S8. Thermodynamic data of the reaction between [K(18-c-6)][NCCN$_2$] (**2^{CN}**) with different carbenes (r^{2}SCAN-3c(CPCM))[4].

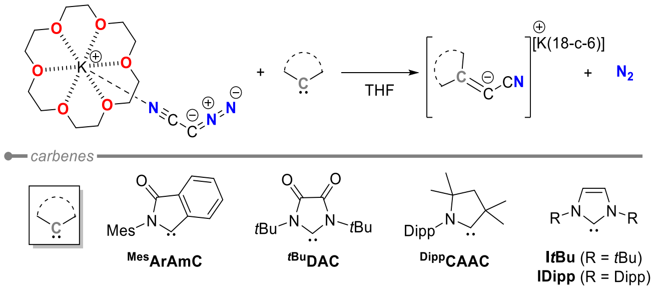

carbene	PG	$\Delta_r H°$/ kcal mol^{-1}	$\Delta_r G°$/ kcal mol^{-1}
^{Mes}ArAmC	C_1	−94.4	−88.5
^{Dipp}CAAC	C_1	−68.1	−62.8
^{tBu}DAC	C_1	−67.8	−62.0
IDipp	C_1	−53.3	−47.3
I*t*Bu	C_1	−37.7	−33.4

5.4. Calculation of HOMO-LUMO gaps and Singlet-Triplet gaps

For calculation of HOMO-LUMO gaps the energies of the corresponding frontier orbitals were taken from the optimisations and frequency analysis at r^{2}SCAN-3c(CPCM)[4] level of theory. For calculation of singlet-triplet gaps the optimised singlet carbene structures were used as starting structures for optimisation in triplet state followed by a frequency analysis confirming local minimum nature (adiabatic excitation).[35] Aiming for more precise electronic energies, coupled cluster calculations were performed on optimised geometries of singlet and triplet carbenes, respectively. For this the Domain-based Local Pair Natural Orbital scheme (DLPNO-CCSD(T)/def2-tzvp)[5] was used in connection with chain of spheres algorithm (RIJCOSX).[36,37]

[4] defgrid3, verytightopt

[5] CPCM(THF), defgrid3

Table S9: Calculated single point energies E_{tot}, Gibbs energies G°, energies of HOMO and LUMO and singlet triplet energies $\Delta E_{S-T}^{CCSD(T)}$ of five different carbenes calculated at r²SCAN-3c/CPCM(THF) and DLPNO-CCSD(T)/def2-TZVP/CPCM(THF) level of theory.

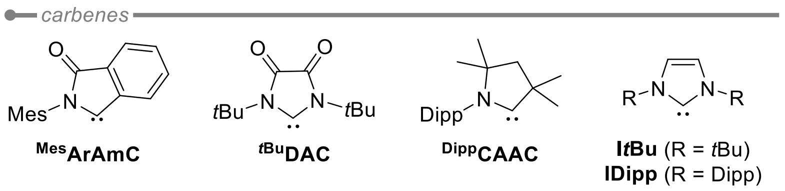

carbene	PG	multiplicity	E_{tot}/ a.u.[a]	G°/ kcal mol⁻¹	$\Delta E_{S-T}^{CCSD(T)}$/ a.u.[b]	T_1[c]	$\Delta E_{S-T}^{CCSD(T)}$/ eV	E_{HOMO}/ eV	E_{LUMO}/ eV	$E_{HOMO-LUMO}$/ eV
MesArAmC	C_1	1	−786.604361	−786.376254	−785.3313	0.012511583	1.22	−5.4853	−3.7593	1.7
		3	−786.579588	−786.352907	−785.2864	0.017824759				
DippCAAC	C_1	1	−835.285087	−834.869059	−833.9052	0.010243351	2.44	−4.8275	−1.1214	3.7
		3	−835.209104	−834.796791	−833.8156	0.011088762				
5-DAC	C_1	1	−689.796262	−689.559358	−688.7535	0.01322952	1.76	−5.6238	−3.7472	1.9
		3	−689.757608	−689.522934	−688.6888	0.016288322				
IDipp	C_1	1	−1159.74743	−1159.23777	−1157.837	0.010302157	4.09	−5.6032	−1.0764	4.5
		3	−1159.62178	−1159.11598	−1157.686	0.014104556				
I*t*Bu	C_1	1	−540.541922	−540.287894	−539.6771	0.011035388	3.80	−5.0973	0.2747	5.4
		3	−540.417611	−540.168873	−539.5376	0.01588747				

[a] single point r²SCAN-3c/CPCM(THF) energy [b] single-point DLPNO-CCSD(T)/def2-TZVP/CPCM(THF) energy [c] T_1 diagnostics[38]

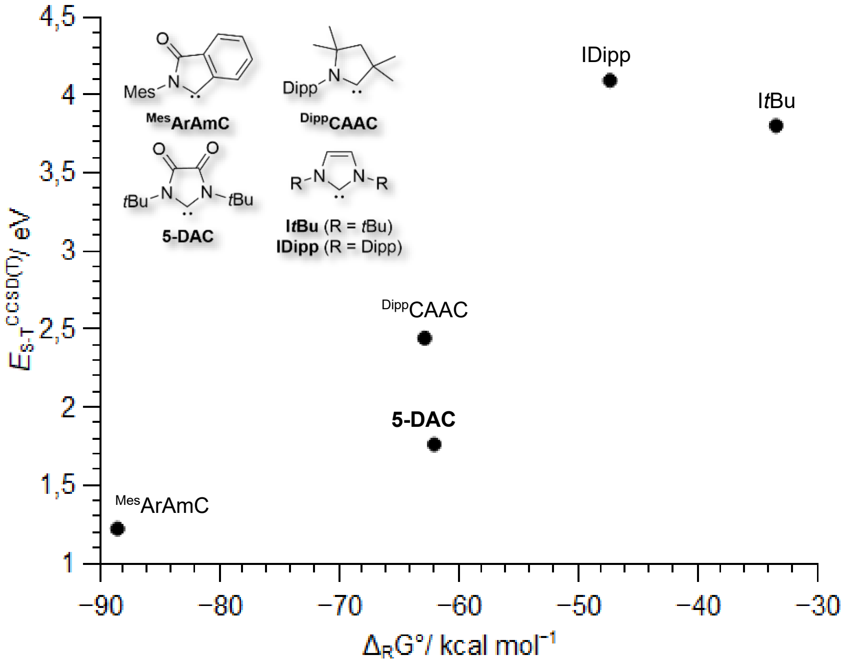

Figure S73: Diagram showing calculated singlet triplet energy difference (DLPNO-CCSD(T)/CPCM(THF)) dependent on free reaction enthalpy $\Delta_\mathrm{r}G°$ for formation of anionic NHO potassium salts from [K(18-c-6)][$NCCN_2$] (**2**$^{\mathbf{CN}}$) and different carbens.

5.5. Thermodynamic calculation of trimethylsilyl addition to free anion 3$^{\mathbf{CN}}$

Reactants and reaction products were pre-optimised on the GFN2-xTB level of theory using the xTB software package version 6.4.1[22,32] by a conformer search on the same level of theory using CREST6 version 3.0.2.[23,24] The resulting conformer ensemble was further scanned with CENSO workflow (version 1.2.0)[26] including step 2. The resulting energetically lowest conformer was chosen for a geometry optimisation and frequency7 analysis (ExtremeSCF) at the PBE0-D4/ma-def2TZVPP[15–17,19,20] level of theory with ORCA, to ensure that it is a local minimum on the potential energy surface (PES).

Table S10: Calculated thermodynamic data for addition reaction of $SiMe_3^+$ to free anion of **3**$^{\mathbf{CN}}$ in the gas phase at PBE0-D4/ma-defz-TZVPP level of theory.

**compound**	**PG**	$\boldsymbol{E_{tot}}$**/ a.u.**[a]	***H*/ a.u.**	***G*/ a.u.**
[^{tBu}DAC-C-P(O)Ph_2]$^-$ (3^{CN})	C_1	−1606.9891	−1606.48	−1606.57
[$SiMe_3$]$^+$	C_1	−408.7689	−408.6513	−408.691
^{tBu}DAC-C-P(O$SiMe_3$)Ph_2	C_1	−2016.0271	−2015.3979	−2015.4992
^{tBu}DAC-C($SiMe_3$)-P(O)Ph_2	C_1	−2016.0115	−2015.3813	−2015.4792

[a] single point PBE0-D4/ma-def2-TZVPP energy

6 CREST energy thershold: 10 kcal mol^{-1}

7 Numerical integration grid set to defgrid3 and very tight optimization convergence VERYTIGHTOPT was chosen.

Table S11: Reaction enthalpies $\Delta_r H°$ and free reaction enthalpies $\Delta_r G°$ for addition to free anion 3^{CN} in the gas phase at PBE0-D4/ma-def2-TZVPP level of theory.

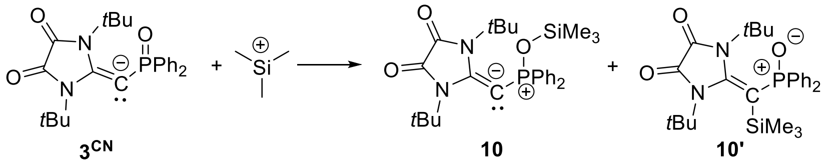

Reaction product	$\Delta_r H°$/ kcal mol^{-1}	$\Delta_r H°$/ kJ mol^{-1}	$\Delta_r G°$/ kcal mol^{-1}.	$\Delta_r G°$/ kJ mol^{-1}
**10**	−165.5	−692.3	−149.9	−627.1
**10'**	−155.1	−648.9	−137.4	−574.7

5.6. Mechanism

To calculate the substitution and rearrangement mechanism, the corresponding reactants and products were first optimised at the PBE-D3/def2-svp/CPCM(THF)[20,34,39,40] level of theory. Then, the Nudged Elastic Band Method (NEB-TS)[41] implemented in ORCA was used for transition state search. Furthermore, an IRC scan was performed to examine whether the saddle points found, connected the corresponding start and end structures. The calculated intermediates and transition states were finally optimised at r^{2}SCAN-3c/CPCM(THF)[4] level of theory.

In case of the substitution reaction, a stepwise mechanism initiated by nitrogen extrusion from the cyano(diazo)methanide anion (**2^{CN}**) followed by transfer of a [K(18-c-6)][CCN] unit was initially suggested (Figure S74). Based on the first NEB-TS run (Figure S74), finer scans were performed in between steps 2 and 10, which led to the localisation of INT1 (Figure S75). (Un)Relaxed surface scans of the corresponding bonds[8] in INT1 then enabled optimisation and verification through frequency analysis of the transition states TS1 and TS2.

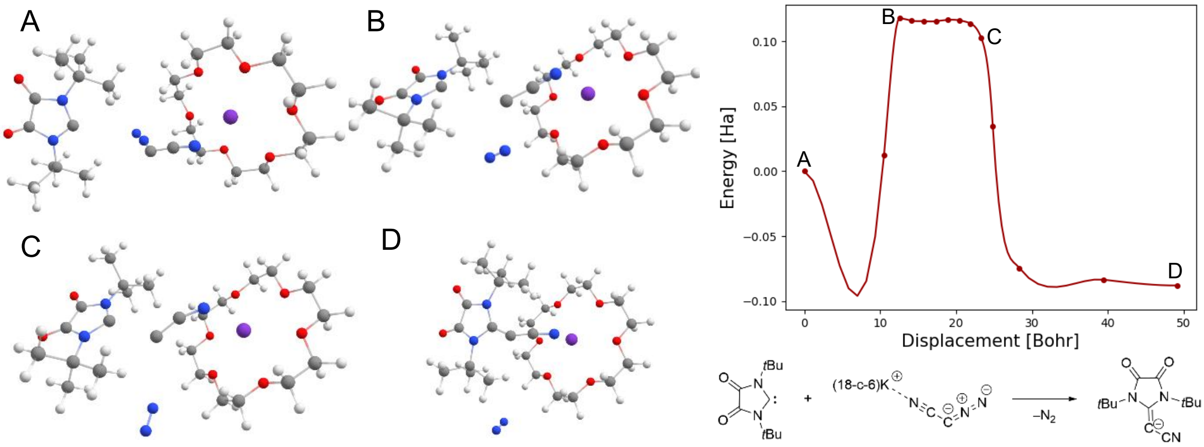

Figure S74: Optimisation profile from NEB-TS run for substitution reaction between [K(18-c-6)][$NCCN_2$] (**2^{CN}**) and **5-DAC** at PBE-D3/def2-svp/CPCM(THF) level of theory. Selected structures along the path were depicted on the left side.

[8] C45-C48 for TS1 and C45-N46 for TS2 in INT1, respectively. In case of surface scan along C45-C48 in INT1, all angels and dihedrals in the molecule were constrained (quasi unrelaxed surface scan), otherwise no saddle point could be located.

Likewise, the rearrangement to the anionic *N*-heterocyclic imine (**5**$^{\mathbf{CN}}$) was calculated The formation of **5**$^{\mathbf{CN}}$ requires the cleavage of one amide C-N bond in **3**$^{\mathbf{CN}}$ with subsequent rotation around the C-N bond and formation of a new C−C bond.

The same procedure as described above was applied for the rearrangement of the anionic *N*-heterocyclic olefin to the anionic *N*-heterocyclic Imine (**5**$^{\mathbf{CN}}$). Here, two almost identical transition states were found with the first NEB-TS runs, which could be optimised as such and confirmed by means of frequency analysis. Both saddle points differ in the position of the cation, whereby the coordination at the terminal cyanide group is favoured by 4.75 kcal/mol compared to the interaction of the cation with both carbonyl functions. The opposite trend was observed for anionic NHO (**3**$^{\mathbf{CN}}$), where the coordination of the chelated potassium cation to both carbonyl groups is slightly favoured by 1.9 kcal/mol compared to the positioning at the cyano group.

Unfortunately, the calculated reaction barrier for a concerted one-step mechanism of 65.3 kcal/mol is too high to explain the observed rearrangement of compound **3**$^{\mathbf{CN}}$ at room temperature. This may indicate an inadequate description of the rearrangement mechanism as unimolecular, the modelling of which with quantum chemical methods would be very computationally intensive on the one hand and would go beyond the scope of this work on the other.

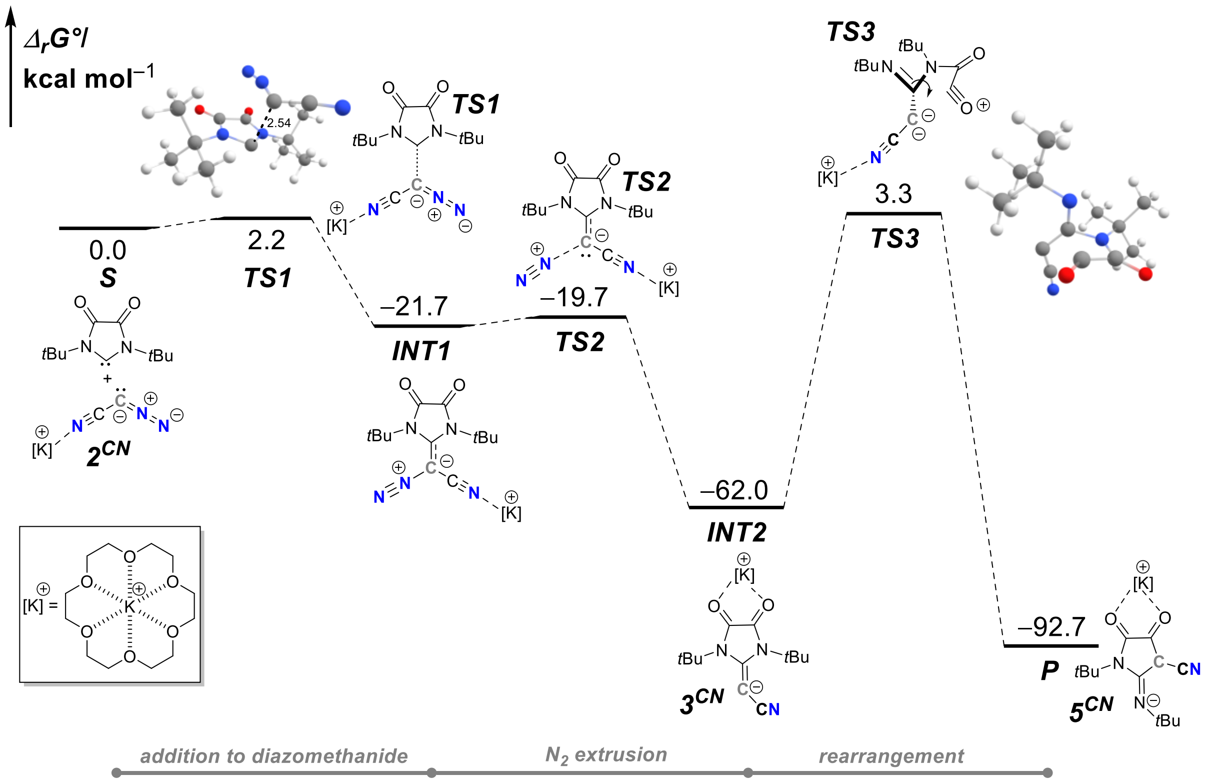

Figure S75. Calculated free reaction enthalpies $\Delta_rG°$ along the experimentally identified compounds INT2 (3^{CN}) and **5**$^{\mathbf{CN}}$. Level of theory r^{2}SCAN-3c/def2-TZVP/CPCM(THF). $[K(18\text{-}c\text{-}6)]^+$ was omitted for clarity in the inset molecular structures (*c.f.*: TS1, TS3). $\Delta_rG°$ were referenced to standard state in solution 1 mol/l.[38]

Table S 12: Calculated thermodynamic values of starting materials, intermediates, transition states and products for the reaction mechanism of ligand substitution reaction and rearrangement. All structures were calculated at r^{2}SCAN-3c/CPCM(THF)[4] level of theory.

**Compound**	**Nr.**	**PG**	***E*$_{tot.}$/ a.u.[a]**	**H/ a.u.**	**G/ a.u.**
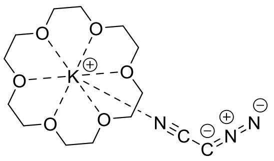	**2^{CN}**	*C*$_1$	−1763.16362	−1762.74679	−1762.82967
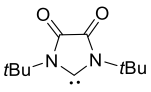	**5-DAC**	*C*$_1$	−689.796262	−689.5013	−689.559358
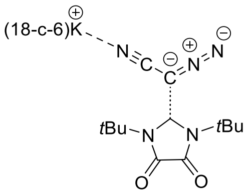	TS1	*C*$_1$	−2452.98053	−2452.26775	−2452.38246
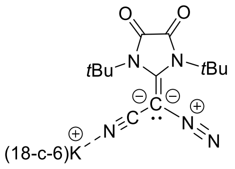	INT1	*C*$_1$	−2453.02059	−2452.30639	−2452.42061
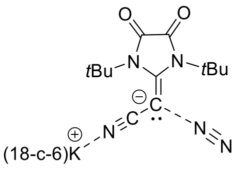	TS2	*C*$_1$	−2453.01488	−2452.30233	−2452.41741
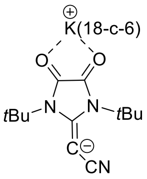	**3^{CN}**	*C*$_1$	−2343.56193	−2342.85745	−2342.96679
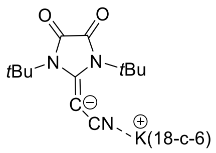	**3^{CN}’**	*C*$_1$	−2343.55703	−2342.85288	−2342.96374
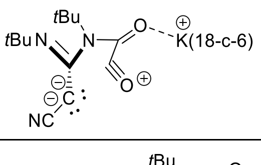	TS3’	*C*$_1$	−2343.44331	−2342.74413	−2342.85526
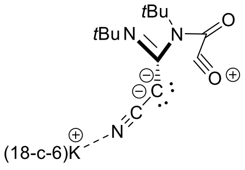	TS3	*C*$_1$	−2343.45006	−2342.75122	−2342.86284

Compound	Nr.	PG	$E_{tot.}$/ a.u.[a]	H/ a.u.	G/ a.u.
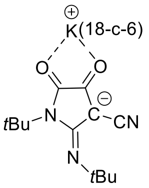	$\mathbf{5^{CN}}$	C_1	−2343.6101	−2342.90607	−2343.01578

5.7. Calculation of activation barriers for different singlet carbenes

The original aim was to calculate the activation barriers of adduct formation (first reaction step in Figure S75) for five different singlet carbenes and correlate them with the corresponding LUMO energies of the compounds. Unfortunately, the corresponding intermediates (INT1) could not be optimized without negative frequencies, except in the case of **5-DAC** and I*t*Bu. For I*t*Bu, the optimization succeeded only when using the highest convergence criteria with respect to optimisation (Verytightopt) SCF procedure (ExtremeSCF) and the integration grid defgrid3. It should also be mentioned that optimisations were only successful if the bond between the central carbon atom and the dinitrogen substituent in die cyanodiazomethanide ($\mathbf{2^{CN}}$) was constrained. Otherwise, nitrogen extrusion under formation of the corresponding anionic NHO was observed. This indicates that the corresponding intermediates are a very shallow minimum on the PES.

Table S13. Calculated thermodynamic data for the transition states (*c.f.* TS1 in Figure S75) for 5-DAC and ItBu at r^{2}SCAN-3c/CPCM(THF) level of theory.

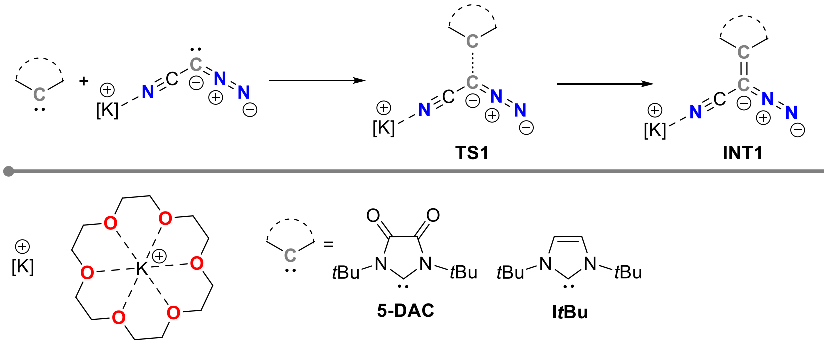

	PG[a]	N_{imag}[b]	$E_{tot.}$/ a.u.[c]	H/ a.u.	G/ a.u.	H_{corr}/ a.u.[d]	G_{corr}/a.u.[d]
5-DAC (TS1)	C_1	1	−2452.98023	−2452.26751	−2452.38245	0.71272839	0.59778479
5-DAC (INT1)	C_1	0	−2453.02059	−2452.30639	−2452.42061	0.71420009	0.59998322
I*t*Bu (TS1)	C_1	1	−2303.65267	−2302.93738	−2303.0439	0.71529205	0.60876914
I*t*Bu (INT1)	C_1	0	−2303.70626	−2302.9805	−2303.09305	0.72576096	0.61320728

[a] point group; [b] number of imaginary frequencies; [c] total electronic energy in atomic units; [d] (free) enthalpy in atomic units.

5.8. Optimized structures (.xyz-files)

5.8.1. ^{tBu}DAC=C-P(O)Ph$_2$ anion (3^{PO})

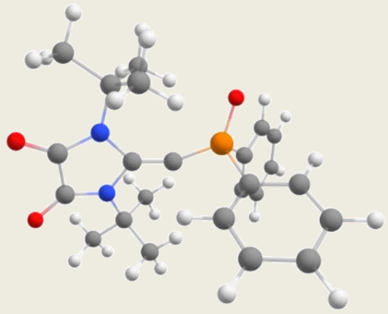

```
58
[tBuDAC-C-P(O)Ph2]- @ PBE0 D4 ma-def2-TZVPP VERYTIGHTOPT ExtremeSCF defgrid3
C     -0.120494    0.339955    0.240712
P      1.442892    0.605349   -0.294646
O      1.922970    1.710839   -1.187981
C      2.484477    0.698334    1.211204
C      3.355394    1.771499    1.359854
C      2.436415   -0.273745    2.205915
C      4.154247    1.881158    2.490930
H      3.388417    2.509898    0.567072
C      3.242141   -0.176528    3.328497
H      1.753229   -1.107101    2.093052
C      4.100853    0.906352    3.475965
H      4.823696    2.727413    2.601358
H      3.197548   -0.942154    4.095220
H      4.726185    0.987949    4.358242
C      1.912392   -1.010837   -1.007938
C      3.253114   -1.237695   -1.310062
C      0.978371   -1.990399   -1.323901
C      3.653598   -2.426412   -1.899356
H      3.983622   -0.469232   -1.082987
C      1.373675   -3.176607   -1.924734
H     -0.064037   -1.809587   -1.088827
C      2.713471   -3.400471   -2.208710
H      4.701640   -2.593021   -2.122818
H      0.633081   -3.930406   -2.167567
H      3.024705   -4.330296   -2.671622
C     -1.420827    0.411595    0.124606
C     -3.566434   -0.391935    0.397702
C     -3.509257    0.493333   -0.838073
N     -2.347011   -0.296189    0.979629
N     -2.266193    1.039914   -0.858753
O     -4.546990   -1.028485    0.724067
O     -4.437756    0.624717   -1.607566
C     -1.930923   -0.953813    2.237018
C     -3.145858   -1.358882    3.071386
C     -1.113362   -2.201794    1.912547
C     -1.121385    0.031355    3.079685
H     -3.804859   -0.509614    3.255880
H     -3.735502   -2.141462    2.603249
H     -2.769946   -1.716422    4.033198
H     -1.717823   -2.908932    1.339984
H     -0.234417   -1.930931    1.330105
H     -0.799972   -2.691136    2.838476
H     -0.837530   -0.454452    4.016234
H     -0.227483    0.357611    2.555817
H     -1.730391    0.906331    3.319219
C     -1.797778    2.065713   -1.820076
C     -0.979382    3.120302   -1.076895
C     -0.987917    1.404384   -2.931122
C     -2.988761    2.795889   -2.445021
H     -1.553855    3.511459   -0.233735
H     -0.022519    2.745246   -0.730854
H     -0.776180    3.945041   -1.763595
```

```
H      -1.592914    0.646227   -3.433282
H      -0.697489    2.157238   -3.667720
H      -0.075938    0.954560   -2.547271
H      -2.583344    3.620256   -3.035576
H      -3.587123    2.155836   -3.085857
H      -3.646021    3.214243   -1.680953
```

5.8.2. ^{tBu}DAC=C-CN anion (3^{CN})

```
[tBuDAC-C-CN]⁻ @ PBE0 D4 ma-def2-TZVPP VERYTIGHTOPT ExtremeSCF defgrid3
C      -0.097800    1.780563    0.277179
C      -1.110205    2.674276    0.423937
C      -3.176772   -1.625680   -0.481535
C       3.752716   -0.382443   -0.186397
H       2.454405    1.077517   -2.061341
H       1.785496    2.309061   -0.966963
H       3.550000    2.086448   -1.102836
H       3.697173    1.763615    1.410181
H       1.928871    1.948601    1.554140
H       2.737474    0.494737    2.189262
H       4.644966    0.247572   -0.228322
H       3.869085   -1.108781    0.613443
H       3.672129   -0.930810   -1.124270
C      -2.418625   -0.334983   -0.164558
C      -2.958056    0.205945    1.158410
H      -2.234619    1.595959   -1.194465
H      -2.303314    0.176316   -2.256209
H      -3.760698    0.755479   -1.435878
H      -3.118651   -2.354612    0.321774
H      -4.221871   -1.344166   -0.632870
C      -2.682323    0.617606   -1.331330
H      -2.812121   -2.102223   -1.390639
H      -4.040198    0.335695    1.079905
H      -2.757666   -0.513226    1.955823
H      -2.526100    1.166933    1.422853
C       0.007192    0.480582    0.079183
C       1.133166   -1.533138    0.023449
C      -0.360369   -1.793919   -0.012253
N       1.279955   -0.185958    0.014660
N      -0.962365   -0.575420   -0.049573
O       1.963361   -2.419878    0.054845
O      -0.841268   -2.908712   -0.003098
C       2.565299    0.553783    0.034792
C       2.581454    1.579690   -1.099068
C       2.738038    1.239057    1.389431
N      -1.893197    3.538227    0.564837
```

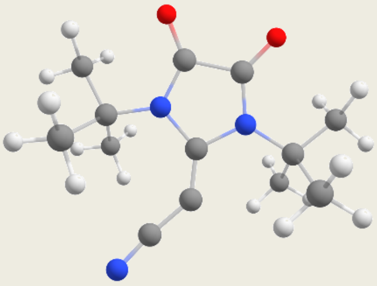

5.8.3. ^{tBu}DAC=C-Tos anion (3^{Tos})

```
51
[tBuDAC-C-Tos]⁻ @ PBE0 D4 ma-def2-TZVPP VERYTIGHTOPT ExtremeSCF defgrid3
C      -0.229985   -0.526654    0.164375
O       1.045623   -2.334582    1.414174
C       2.469542   -0.706565    0.056831
C       2.999538   -0.350287   -1.176146
C       3.099471   -0.276170    1.217131
C       4.128085    0.449942   -1.242736
H       2.521995   -0.717042   -2.076117
```

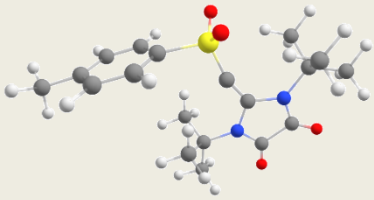

```
C      4.227524    0.523668    1.140093
H      2.699355   -0.585369    2.174632
C      4.756484    0.909473   -0.088583
H      4.534968    0.719159   -2.212746
H      4.712972    0.851172    2.054449
C     -1.388546    0.065443    0.193889
C     -2.901441    1.808430    0.202075
C     -3.651219    0.488889    0.289131
N     -1.584231    1.490829    0.147749
N     -2.708938   -0.489914    0.277192
O     -3.454690    2.887784    0.189617
O     -4.859822    0.418775    0.353529
C     -0.447554    2.436446    0.052463
C      0.450117    2.273363    1.277598
C      0.324526    2.169774   -1.238500
C     -0.922611    3.887527    0.015937
H     -0.110820    2.507744    2.185292
H      0.834658    1.260067    1.349741
H      1.292261    2.965227    1.203525
H     -0.324597    2.332552   -2.102013
H      1.167429    2.861459   -1.304706
H      0.703259    1.152080   -1.266287
H     -0.025482    4.507769   -0.056254
H     -1.560351    4.093763   -0.841034
H     -1.470347    4.167529    0.913238
C     -2.953405   -1.951093    0.344425
C     -2.413057   -2.610340   -0.922897
C     -2.302016   -2.512443    1.606661
C     -4.446921   -2.266979    0.421238
H     -2.933935   -2.209086   -1.795956
H     -1.344102   -2.468550   -1.054578
H     -2.610292   -3.684047   -0.875609
H     -2.743307   -2.040554    2.488200
H     -2.501939   -3.585306    1.661868
H     -1.225526   -2.367590    1.630327
H     -4.530571   -3.355794    0.463704
H     -4.916808   -1.845785    1.307355
H     -4.993255   -1.908863   -0.448815
S      0.939247   -1.625364    0.149392
O      0.938507   -2.409811   -1.074794
C      5.955347    1.806167   -0.166111
H      6.615465    1.663565    0.691837
H      6.533492    1.620265   -1.073450
H      5.661778    2.860747   -0.178225
```

5.8.4. Carbenes

```
34
MesArAmC @ r2SCAN-3c CPCM(THF) VERYTIGHTOPT defgrid3
H     -5.817064   -0.422019   -0.938775
N      0.229137    0.100349    0.197331
O      0.505277   -0.881885   -1.939100
C     -1.207285    0.086578    0.164362
C      2.398535   -0.233195   -0.526810
C     -1.878123    1.193305   -0.362357
C     -1.879980   -1.040180    0.644438
C     -3.274363   -1.033847    0.598146
H     -3.816696   -1.898861    0.974393
```

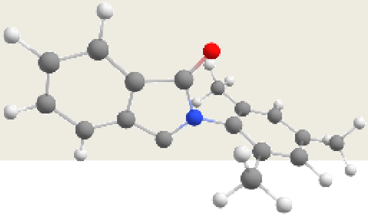

```
C       0.997898   -0.423051   -0.940194
C       2.364762    0.352161    0.744031
C      -3.272670    1.156158   -0.389296
H      -3.813474    2.011458   -0.789145
C      -3.984718    0.051849    0.082414
C       3.543212    0.655267    1.402363
H       3.530636    1.109802    2.389362
C      -1.123377   -2.210681    1.206055
H      -1.811438   -2.999531    1.518163
H      -0.521770   -1.911667    2.072166
H      -0.432238   -2.631595    0.466197
C       0.963723    0.567071    1.210157
C       4.753637    0.357981    0.757151
H       5.693860    0.585338    1.251160
C       3.578141   -0.532881   -1.177373
H       3.586670   -0.987324   -2.164497
C      -1.119686    2.388303   -0.867051
H      -0.522424    2.841600   -0.067489
H      -1.806274    3.143085   -1.256961
H      -0.424047    2.111003   -1.667901
C       4.771987   -0.224830   -0.508373
H       5.723761   -0.443437   -0.983360
C      -5.487079    0.019721    0.009731
H      -5.908757    1.027299    0.066767
H      -5.910318   -0.584548    0.817307
```

```
33
tBuDAC @ r2SCAN-3c CPCM(THF) VERYTIGHTOPT defgrid3
C       0.000036   -0.920170    0.001227
C       0.771604    1.238300   -0.048297
C      -0.771920    1.238308    0.043921
N       1.105324   -0.106072   -0.065770
N      -1.105396   -0.106063    0.065676
O      -1.473196    2.221560    0.084582
O       1.472704    2.221544   -0.092069
C      -2.514634   -0.616261    0.149397
C      -2.501854   -2.141489    0.152471
C      -3.137735   -0.100914    1.451691
C      -3.285220   -0.107436   -1.074160
H      -3.540177   -2.481551    0.215281
H      -2.053672   -2.541234   -0.761056
H      -1.947722   -2.536959    1.007917
H      -2.560902   -0.449143    2.314949
H      -3.185798    0.990531    1.472022
H      -4.156510   -0.491078    1.536554
H      -3.335083    0.983932   -1.094686
H      -2.812795   -0.460658   -1.996794
H      -4.306998   -0.497268   -1.037747
C       2.514657   -0.616271   -0.147885
C       3.285230   -0.103215    1.073913
C       2.502173   -2.141500   -0.145864
C       3.137568   -0.105129   -1.451925
H       3.334926    0.988225    1.090778
H       2.812919   -0.453413    1.997758
H       4.307066   -0.493010    1.038746
H       2.054103   -2.538271    0.769014
H       1.948083   -2.539939   -0.999956
H       3.540558   -2.481576   -0.207574
```

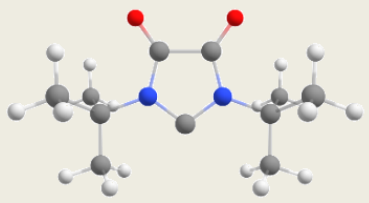

```
H       4.156469   -0.495247   -1.535486
H       2.560823   -0.456411   -2.314000
H       3.185325    0.986257   -1.475893
```

```
52
DippCAAC @ r2SCAN-3c CPCM(THF) VERYTIGHTOPT defgrid3
C       1.372813    0.046741   -1.072992
N       0.646949    0.056110    0.011893
C      -0.797995   -0.006727   -0.040526
C      -1.528295    1.195445   -0.080082
C      -1.437103   -1.259272   -0.090463
C      -2.923038    1.117799   -0.064897
C      -2.834570   -1.283244   -0.071230
C      -3.573467   -0.108661   -0.033715
H      -3.508351    2.032504   -0.097239
H      -3.351083   -2.238303   -0.108743
H      -4.659244   -0.148833   -0.013617
C      -0.867176    2.549761   -0.265634
C      -1.379445    3.609163    0.717273
C      -1.064853    3.018670   -1.717025
H       0.209272    2.434693   -0.113696
H      -2.427553    3.861378    0.523746
H      -1.298666    3.268643    1.754434
H      -0.794607    4.529424    0.610587
H      -2.128992    3.164781   -1.935704
H      -0.547719    3.971022   -1.880875
H      -0.667148    2.281921   -2.422744
C      -0.682797   -2.560201   -0.298374
C      -0.858334   -3.022445   -1.754970
C      -1.108223   -3.669932    0.670234
H       0.382397   -2.370425   -0.151235
H      -1.910055   -3.247828   -1.966483
H      -0.525334   -2.247503   -2.452965
H      -0.270707   -3.929587   -1.937012
H      -2.138330   -3.991990    0.483375
H      -0.461611   -4.544552    0.539194
H      -1.038938   -3.345907    1.713484
C       3.447742   -1.278198   -0.949576
H       2.944620   -2.085550   -0.407265
H       3.376908   -1.493349   -2.020958
H       4.505886   -1.279269   -0.662587
C       3.572548    1.182324   -1.408837
H       3.569553    0.962894   -2.481641
H       3.103326    2.160982   -1.256743
H       4.613175    1.243821   -1.067677
C       2.825861    0.092963   -0.631833
C       1.371676    0.097637    1.368943
C       1.240304   -1.247715    2.084206
H       0.190846   -1.482151    2.289413
H       1.681518   -2.066157    1.510018
H       1.763626   -1.185810    3.043914
C       0.814287    1.189720    2.277415
H       0.935325    2.183724    1.841330
H      -0.244560    1.023220    2.502779
H       1.366388    1.168939    3.223044
C       2.801712    0.390481    0.892232
H       3.534473   -0.206894    1.444438
H       3.035126    1.447419    1.064339
```

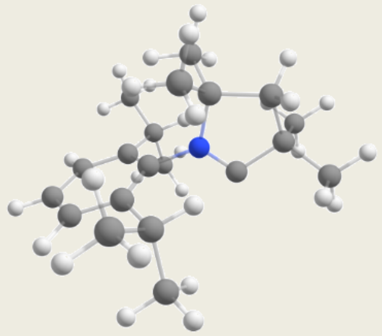

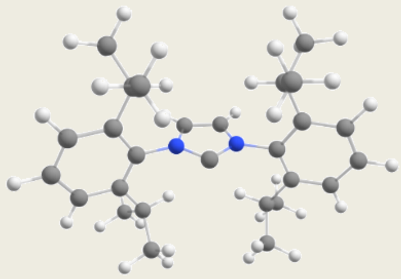

```
65
IDipp @ r2SCAN-3c CPCM(THF) VERYTIGHTOPT defgrid3
C       0.001036   -0.001052   -0.304953
N       1.056964    0.071769    0.560969
N      -1.058486   -0.067893    0.557059
C      -2.420249   -0.160893    0.118411
C      -2.977798   -1.433041   -0.075306
C      -3.135996    1.023450   -0.107657
C      -4.299589   -1.499026   -0.522864
C      -4.454870    0.907236   -0.554184
C      -5.030573   -0.340836   -0.760557
H      -4.760338   -2.468279   -0.693319
H      -5.036149    1.804737   -0.748133
H      -6.056844   -0.411569   -1.110959
C       2.420570    0.161190    0.127330
C       2.979350    1.431706   -0.073358
C       3.136866   -1.024994   -0.087029
C       4.303049    1.494005   -0.515781
C       4.457697   -0.912467   -0.528688
C       5.034682    0.333888   -0.741781
H       4.764825    2.461846   -0.691421
H       5.039524   -1.811562   -0.713411
H       6.062474    0.401741   -1.088277
C      -2.180501   -2.704120    0.149054
C      -1.860920   -3.380701   -1.192208
C      -2.899870   -3.671522    1.097503
H      -1.227111   -2.432829    0.615144
H      -1.317153   -2.698304   -1.854009
H      -1.242700   -4.270927   -1.030059
H      -2.781040   -3.691818   -1.700445
H      -2.261573   -4.538234    1.301131
H      -3.136442   -3.186631    2.050699
H      -3.834334   -4.041177    0.661472
C      -2.512877    2.392781    0.087918
C      -3.332953    3.257207    1.054419
C      -2.326518    3.098621   -1.263056
H      -1.518783    2.257528    0.527512
H      -4.324096    3.483893    0.646494
H      -3.467768    2.752421    2.017074
H      -2.819638    4.208388    1.233145
H      -3.293792    3.280127   -1.745730
H      -1.830145    4.065119   -1.120277
H      -1.714129    2.492402   -1.938960
C       2.512604   -2.392682    0.116169
C       3.326905   -3.248634    1.095044
C       2.334602   -3.110099   -1.229807
H       1.515801   -2.253853    0.548449
H       3.455463   -2.735719    2.054252
H       2.812908   -4.198544    1.278523
H       4.320698   -3.478154    0.695224
H       1.837217   -4.075282   -1.081764
H       1.726495   -2.509725   -1.914732
H       3.304802   -3.295899   -1.704900
C       2.181616    2.704653    0.138477
C       1.869025    3.372423   -1.208824
C       2.896864    3.677932    1.084035
H       1.225781    2.436964    0.601623
H       2.791778    3.679579   -1.714686
H       1.328024    2.685937   -1.868664
```

```
H       1.250589    4.264086   -1.055648
H       3.128206    3.199296    2.041665
H       3.833829    4.043925    0.650288
H       2.258208    4.546467    1.278580
C      -0.679755   -0.037872    1.894534
C       0.672693    0.050931    1.897032
H      -1.393134   -0.082156    2.704593
H       1.382709    0.100815    2.709717
```

```
33
ItBu @ r2SCAN-3c CPCM(THF) VERYTIGHTOPT defgrid3
C       0.005419    0.586129    0.337163
N       1.065613   -0.142501   -0.123521
N      -1.068150   -0.163966   -0.052898
C      -0.688347   -1.316593   -0.731396
C       0.665071   -1.303394   -0.775503
H      -1.384228   -2.043379   -1.123349
H       1.348003   -2.017743   -1.210736
C      -2.493279    0.182352    0.195314
C      -3.200041    0.340145   -1.157247
C      -3.136886   -0.948901    1.007485
C      -2.589032    1.489511    0.977431
H      -3.177028   -0.589543   -1.734618
H      -4.247943    0.611505   -0.994588
H      -2.722799    1.130075   -1.746843
H      -2.613049   -1.080214    1.960212
H      -4.182493   -0.701218    1.215975
H      -3.116652   -1.898344    0.463372
H      -3.648703    1.711127    1.141114
H      -2.089773    1.410155    1.946529
H      -2.134492    2.316727    0.426532
C       2.497085    0.226455    0.041092
C       3.195025   -0.876198    0.847724
C       3.131853    0.356539   -1.349567
C       2.615999    1.555350    0.782227
H       2.724742   -0.985052    1.830752
H       3.152690   -1.840336    0.331236
H       4.248220   -0.615212    0.992091
H       3.096026   -0.588856   -1.899914
H       2.613017    1.122570   -1.935543
H       4.181900    0.648086   -1.247098
H       3.679645    1.794380    0.883842
H       2.122090    2.361589    0.233820
H       2.168755    1.496766    1.777710
```

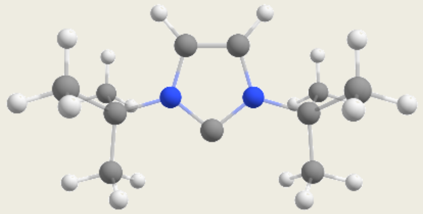

5.8.5. *[K(18-c-6)][(carbene)-C-CN]*

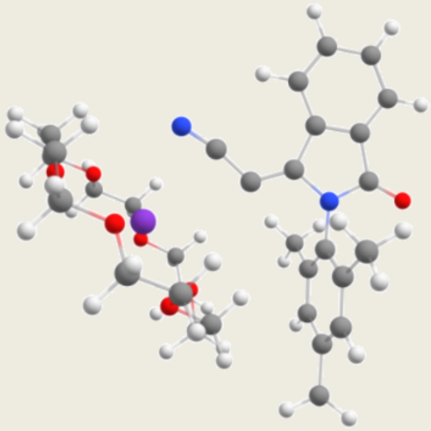

```
80
[K(18-c-6)][MesArAmC=CCN] @ r2SCAN-3c CPCM(THF) VERYTIGHTOPT defgrid3
H     -5.677340    0.065763   -1.791491
N      0.288705    0.301410   -0.187631
O      0.452574   -1.246882    1.543853
C     -1.112679    0.144710   -0.402232
C      2.367918    0.001715    0.689692
C     -1.558643   -0.899073   -1.220029
C     -2.003712    0.985322    0.271596
C     -3.371581    0.762691    0.107945
H     -4.076142    1.408892    0.628575
C      0.961016   -0.424984    0.781170
C      2.472356    0.960675   -0.326072
C     -2.934082   -1.089120   -1.356218
H     -3.295006   -1.897136   -1.990044
C     -3.853418   -0.268454   -0.699936
C      3.708363    1.531654   -0.621118
H      3.810687    2.275447   -1.404676
C     -1.492944    2.089711    1.151558
H     -2.320318    2.678876    1.555711
H     -0.829184    2.755953    0.588587
H     -0.911637    1.688999    1.990735
C      1.142982    1.181203   -0.914484
C      4.815404    1.120423    0.119216
H      5.788774    1.553171   -0.095027
C      3.467047   -0.411690    1.430774
H      3.362570   -1.157172    2.214531
C     -0.574120   -1.790396   -1.922210
H      0.008123   -2.377507   -1.201886
H      0.140025   -1.200355   -2.507968
H     -1.088019   -2.483701   -2.592627
C      4.701258    0.161150    1.133981
H      5.584677   -0.136348    1.691519
C     -5.332189   -0.465292   -0.894603
H     -5.900602   -0.077421   -0.044363
H     -5.578471   -1.522953   -1.028220
C      0.701466    1.992989   -1.897802
C      1.558320    2.840983   -2.557080
N      2.115947    3.649902   -3.216032
K     -0.766606    4.479313   -3.662327
O     -0.354181    3.030694   -6.064799
O     -2.647262    2.436546   -4.489605
O      0.595883    5.715538   -5.915547
O     -3.003406    3.851191   -2.011557
O      0.320080    7.115417   -3.431198
O     -1.892047    6.435191   -1.792033
H     -1.288783    8.093498   -0.681329
C     -1.336470    2.071325   -6.449725
C      0.189460    3.728505   -7.183450
C     -1.913004    1.444632   -5.208486
C     -3.267091    1.883787   -3.325650
C      1.231076    4.698285   -6.693447
C      1.543099    6.671082   -5.434905
C     -3.979396    2.978638   -2.579787
C     -3.588192    4.868649   -1.204294
C      0.824943    7.715824   -4.623802
C     -0.345991    8.060786   -2.596629
```

```
C      -2.490678    5.759138   -0.685591
C      -0.875159    7.348906   -1.380347
H      -0.060919    6.809091   -0.868498
H      -0.880998    1.285154   -7.073771
H      -2.135397    2.557807   -7.033401
H       0.663777    3.021191   -7.883392
H      -0.610004    4.264225   -7.721743
H      -2.576684    0.614697   -5.501350
H      -1.107506    1.038802   -4.573053
H      -2.507268    1.422758   -2.675980
H      -3.995637    1.108773   -3.615505
H       1.738419    5.146280   -7.563451
H       1.980071    4.171591   -6.081359
H       2.303620    6.168544   -4.816928
H       2.048557    7.164249   -6.281342
H      -4.643572    3.540919   -3.257165
H      -4.593215    2.525556   -1.785423
H      -4.123476    4.420961   -0.350443
H      -4.308515    5.459345   -1.794234
H      -0.002208    8.152137   -5.207831
H       1.534598    8.521333   -4.374121
H       0.353986    8.849357   -2.275562
H      -1.175084    8.536391   -3.146136
H      -2.920500    6.486384    0.021859
H      -1.735017    5.159737   -0.150875
```

```
79
[K(18-c-6)][tBuDAC=CCN] @ r2SCAN-3c CPCM(THF) VERYTIGHTOPT defgrid3
C       0.421678    0.426880    1.806179
C       0.027753    1.311140    2.742152
C      -3.503030   -2.065769    2.320043
C       2.666431   -1.968597   -1.234671
H       1.435948    0.438353   -1.740671
H       1.751912    1.180278   -0.149569
H       3.108345    0.669630   -1.189698
H       4.021630   -0.784746    0.711636
H       2.674202   -0.295173    1.771817
H       2.987394   -2.021618    1.454423
H       3.713001   -1.688137   -1.392809
H       2.632159   -2.993450   -0.855110
H       2.149641   -1.934429   -2.194576
C      -2.353711   -1.046252    2.348606
C      -1.739080   -1.115686    3.754034
H      -2.203619    1.127102    2.059924
H      -3.400866    0.312285    1.027566
H      -3.729562    0.561553    2.754329
H      -3.154288   -3.085805    2.499246
H      -4.181856   -1.785413    3.132208
C      -2.946771    0.331196    2.024151
H      -4.057661   -2.044281    1.381579
H      -2.515983   -0.870795    4.485203
H      -1.388155   -2.133405    3.956322
H      -0.911853   -0.423685    3.896734
C      -0.047256   -0.657276    1.206939
C      -0.085802   -2.290994   -0.415596
C      -1.385256   -2.331500    0.362861
N       0.686974   -1.339780    0.166456
N      -1.299359   -1.377542    1.328926
```

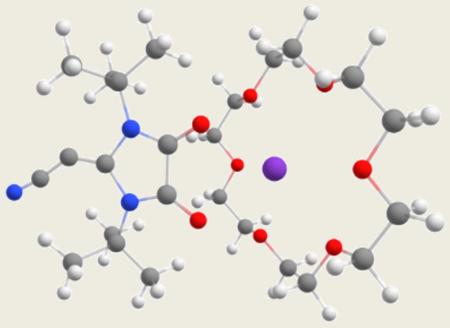

```
O      0.116088   -3.033011   -1.377339
O     -2.280660   -3.129198    0.086013
C      2.094609   -0.966369   -0.222645
C      2.091426    0.427081   -0.862843
C      2.996517   -1.015344    1.019513
N     -0.178035    2.163583    3.540151
K     -0.891026   -5.603845   -0.548109
O      1.944406   -5.447781   -0.164494
O      0.343080   -5.689448    2.151372
O      0.963661   -6.544237   -2.600774
O     -2.469660   -5.963208    1.824253
O     -1.869322   -6.692478   -2.985021
O     -3.433258   -7.039695   -0.624679
H     -4.971719   -7.400055   -1.985563
C      2.500804   -5.523744    1.144391
C      2.798293   -6.009025   -1.158064
C      1.532177   -4.899688    2.114140
C     -0.607994   -5.174710    3.083543
C      2.177205   -5.792960   -2.513389
C      0.329505   -6.376793   -3.869739
C     -1.822196   -6.065413    3.089717
C     -3.642595   -6.768791    1.741672
C     -0.918816   -7.218065   -3.912216
C     -3.067445   -7.468087   -2.946331
C     -4.293154   -6.540909    0.402316
C     -4.003240   -6.877993   -1.924474
H     -4.173573   -5.808997   -2.135350
H      3.455399   -4.973392    1.189267
H      2.692516   -6.573919    1.420131
H      3.783734   -5.514528   -1.140724
H      2.948102   -7.084987   -0.969094
H      1.997599   -4.856990    3.112051
H      1.293375   -3.867743    1.802363
H     -0.894889   -4.147803    2.801007
H     -0.176194   -5.145965    4.097004
H      2.887013   -6.125954   -3.287945
H      1.968021   -4.722147   -2.663632
H      0.079519   -5.315066   -4.032419
H      1.003175   -6.700332   -4.679704
H     -1.528192   -7.109545    3.287209
H     -2.499971   -5.737993    3.895264
H     -4.356591   -6.490257    2.534047
H     -3.385062   -7.833631    1.866173
H     -0.679348   -8.263243   -3.657131
H     -1.330210   -7.196351   -4.934352
H     -3.561356   -7.458765   -3.931463
H     -2.833576   -8.513179   -2.685647
H     -5.260264   -7.068459    0.379267
H     -4.479105   -5.464741    0.251793
```

```
79
[K(18-c-6)][tBuDAC=CCN] @ r2SCAN-3c CPCM(THF) VERYTIGHTOPT defgrid3
C       1.189185     2.494757    -1.067827
C       2.454423     2.927257    -1.179133
C      -3.230140     1.921625    -1.443121
C       2.437078    -0.803823     1.984287
H       2.823162    -0.959057    -0.668304
H       3.187924     0.746720    -1.006153
H       4.214456    -0.171828     0.102517
H       3.828402     1.468473     2.031717
H       2.772579     2.505365     1.063802
H       2.173468     1.799980     2.580606
H       3.497063    -0.866718     2.252434
H       1.861216    -0.624623     2.893541
H       2.132098    -1.760167     1.556706
C      -1.743329     2.300693    -1.478451
C      -1.628846     3.757170    -1.005335
H      -0.208718     2.408212    -3.024464
H      -1.377711     1.079219    -3.233964
H      -1.867314     2.750618    -3.581919
H      -3.663519     2.030936    -0.447514
H      -3.739216     2.615529    -2.120529
C      -1.256511     2.123111    -2.923870
H      -3.408195     0.903908    -1.794617
H      -2.218254     4.392939    -1.675107
H      -2.034456     3.855421     0.007827
H      -0.591003     4.092370    -1.009977
C       0.498174     1.567863    -0.415678
C      -0.186604    -0.179197     0.925804
C      -1.390777     0.396025     0.200483
N       0.897940     0.549515     0.526619
N      -0.926615     1.419195    -0.569758
O      -0.276773    -1.121591     1.703412
O      -2.523243    -0.049181     0.350031
C       2.317661     0.351525     0.978581
C       3.183535    -0.020371    -0.233744
C       2.796821     1.620815     1.697895
N       3.518861     3.416585    -1.379413
K       4.107026     5.833321    -2.703212
O       4.316661     4.448025    -5.156128
O       1.766177     5.467122    -4.336945
O       6.622651     5.727078    -4.109851
O       1.593426     6.687583    -1.740981
O       6.468311     6.880493    -1.510501
O       3.933834     7.888777    -0.682423
H       5.028465     8.653707     0.918407
C       3.147259     4.406842    -5.973449
C       5.506513     4.635658    -5.918843
C       1.940673     4.263058    -5.085064
C       0.624210     5.380712    -3.477745
C       6.688152     4.600840    -4.986860
C       7.725471     5.757684    -3.202742
C       0.512838     6.646176    -2.672393
C       1.557729     7.846529    -0.912748
C       7.621393     6.989902    -2.344884
C       6.317512     8.015195    -0.657150
C       2.712187     7.781717     0.051198
C       5.075054     7.842085     0.174489
H       5.107092     6.880683     0.713632
```

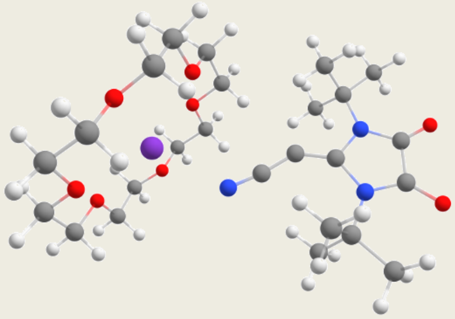

```
H       3.193393    3.547379   -6.661622
H       3.069075    5.328282   -6.573632
H       5.617644    3.830761   -6.663502
H       5.468811    5.599183   -6.453515
H       1.054264    4.071022   -5.711620
H       2.072442    3.408385   -4.400615
H       0.727617    4.514849   -2.803752
H      -0.292198    5.252257   -4.077368
H       7.615408    4.633673   -5.581168
H       6.682724    3.665516   -4.403400
H       7.724144    4.852979   -2.572498
H       8.676580    5.790366   -3.758186
H       0.542769    7.529903   -3.331317
H      -0.450863    6.645208   -2.137597
H       0.616104    7.881730   -0.340846
H       1.623932    8.759380   -1.527747
H       7.546875    7.889010   -2.978293
H       8.531962    7.074061   -1.729868
H       7.187422    8.107211    0.013046
H       6.244856    8.935534   -1.259464
H       2.626859    8.609724    0.773082
H       2.686437    6.830453    0.607998
```

```
98
[K(18-c-6)][DippCAAC=CCN] @ r2SCAN-3c CPCM(THF) VERYTIGHTOPT defgrid3
N      -0.403563   -0.274436    0.046918
C      -0.446168    0.014112    1.405004
C      -1.251011   -0.637842    2.279401
C      -1.275898   -0.435264    3.622253
N      -1.408305   -0.396124    4.808364
H       1.991311    1.924247    0.178379
C      -1.181831   -1.332148   -0.516027
C      -2.447651   -1.039662   -1.069886
C      -0.713178   -2.664319   -0.479843
C      -3.181238   -2.071713   -1.661304
C      -1.481175   -3.663820   -1.084207
C      -2.697796   -3.372850   -1.686646
H      -4.154871   -1.855163   -2.093774
H      -1.126563   -4.691477   -1.067477
H      -3.278874   -4.163234   -2.155166
C      -3.086219    0.333540   -0.964143
C      -4.267747    0.288550    0.017516
C      -3.544875    0.882212   -2.321688
H      -2.342692    1.015503   -0.541408
H      -4.694676    1.290263    0.146531
H      -5.059793   -0.374809   -0.350638
H      -3.933238   -0.076995    0.992748
H      -2.735233    0.872934   -3.057788
H      -4.375317    0.292427   -2.725849
H      -3.897012    1.914157   -2.210316
C       0.543372   -3.075202    0.265700
C       1.538411   -3.840733   -0.617615
C       0.173845   -3.925200    1.491332
H       1.025302   -2.166938    0.635120
H       2.466672   -4.023689   -0.064153
H       1.132895   -4.815143   -0.912821
H       1.787239   -3.290903   -1.529969
H       1.071288   -4.152901    2.079710
```

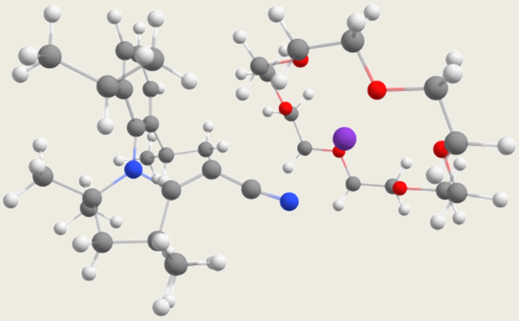

```
H      -0.537149   -3.389210    2.127147
H      -0.277259   -4.876298    1.183103
C       0.587563    0.507922   -0.734766
C       0.569925    1.133550    1.670552
C      -0.003317    1.076307   -2.028209
H       0.770231    1.648004   -2.552638
H      -0.341039    0.275030   -2.695039
H      -0.841964    1.746748   -1.828310
C       1.820401   -0.322955   -1.122042
H       1.532185   -1.151134   -1.776811
H       2.524845    0.311026   -1.671646
H       2.338245   -0.729976   -0.250543
C       1.781155    0.556287    2.421417
H       2.245910   -0.266687    1.869589
H       2.537395    1.335568    2.578793
H       1.468250    0.173111    3.398681
C      -0.039425    2.267713    2.500206
H      -0.943005    2.657727    2.018147
H      -0.307532    1.920539    3.502128
H       0.681743    3.089093    2.598662
C       0.937978    1.633186    0.259455
H       0.330703    2.514855    0.021252
K      -3.084267   -2.444262    5.800191
O      -1.492203   -4.832386    6.303335
O      -2.526447   -4.274220    3.729392
O      -1.955441   -2.939651    8.379830
O      -4.861044   -2.666201    3.510579
O      -4.359022   -1.420804    8.210161
O      -5.396006   -0.819182    5.639729
H      -6.722934    0.385576    6.703051
C      -0.849300   -5.255150    5.099312
C      -0.550604   -4.568024    7.343099
C      -1.897358   -5.509893    4.049193
C      -3.450985   -4.390979    2.648880
C      -1.297687   -4.187786    8.592967
C      -2.701122   -2.527787    9.524255
C      -4.050172   -3.036785    2.386423
C      -5.451177   -1.380472    3.327201
C      -3.375134   -1.216085    9.224585
C      -5.080111   -0.221879    7.921528
C      -6.276691   -1.040298    4.539034
C      -6.091347   -0.506926    6.843364
H      -6.740743   -1.347456    7.139389
H      -0.280113   -6.182190    5.275884
H      -0.149551   -4.477797    4.750285
H       0.054721   -5.465576    7.549383
H       0.130020   -3.754073    7.042415
H      -1.408639   -5.936053    3.158091
H      -2.641893   -6.236035    4.416465
H      -4.235073   -5.125630    2.897846
H      -2.930630   -4.730854    1.737636
H      -0.582185   -4.105700    9.427302
H      -2.035512   -4.967189    8.844789
H      -3.454123   -3.290864    9.781175
H      -2.032008   -2.396412   10.390207
H      -3.246035   -2.292662    2.236098
H      -4.667870   -3.086970    1.473742
H      -6.099066   -1.379560    2.435007
H      -4.666740   -0.619090    3.184221
```

```
H      -2.632550   -0.473039    8.889485
H      -3.848192   -0.840398   10.146279
H      -5.604500    0.134619    8.822782
H      -4.385961    0.567165    7.588315
H      -6.863566   -0.130611    4.332308
H      -6.976342   -1.861000    4.768048
```

```
111
[K(18-c-6)][IDipp=CCN] @ r2SCAN-3c CPCM(THF) VERYTIGHTOPT defgrid3
C      -0.395491   -0.122382   -0.375516
C       0.794806    0.264355   -0.879116
H      -1.381581    0.299445   -0.501605
H       1.048468    1.088390   -1.528604
N       1.761195   -0.629085   -0.421511
N      -0.177937   -1.261794    0.399190
C       1.190106   -1.604350    0.413947
C       1.863666   -2.610815    1.035948
C       1.263592   -3.592397    1.754455
N       0.905294   -4.536682    2.392492
C       3.162432   -0.449242   -0.596635
C       3.831650   -1.220776   -1.561791
C       3.825857    0.512868    0.182161
C       5.195064   -0.991891   -1.753019
C       5.195171    0.698877   -0.034079
C       5.871698   -0.041229   -0.994972
H       5.737484   -1.563043   -2.501518
H       5.734993    1.438454    0.552215
H       6.934632    0.121140   -1.154447
C      -1.161492   -1.865607    1.232712
C      -1.179231   -1.554064    2.600833
C      -2.088229   -2.750359    0.651418
C      -2.143091   -2.175223    3.399583
C      -3.035552   -3.342807    1.486859
C      -3.057818   -3.063616    2.850595
H      -2.180563   -1.955072    4.463418
H      -3.763195   -4.035043    1.074999
H      -3.800040   -3.539165    3.486442
C       3.079596   -2.229163   -2.408072
C       3.858081   -3.533006   -2.612800
C       2.693320   -1.601155   -3.756131
H       2.157696   -2.480785   -1.872542
H       4.164963   -3.963283   -1.652917
H       3.224535   -4.263519   -3.128374
H       4.752938   -3.382666   -3.227528
H       2.095268   -0.694744   -3.611554
H       3.590602   -1.330391   -4.325750
H       2.107620   -2.308588   -4.354353
C       3.096004    1.362085    1.207517
C       3.167765    2.850025    0.834303
C       3.619145    1.127836    2.630385
H       2.040555    1.072326    1.199103
H       2.784194    3.020557   -0.177487
H       2.570185    3.444337    1.534478
H       4.199179    3.218638    0.873169
H       3.472350    0.085913    2.931869
H       4.686464    1.364298    2.710937
H       3.076205    1.765166    3.337805
C      -2.008973   -3.087432   -0.827236
```

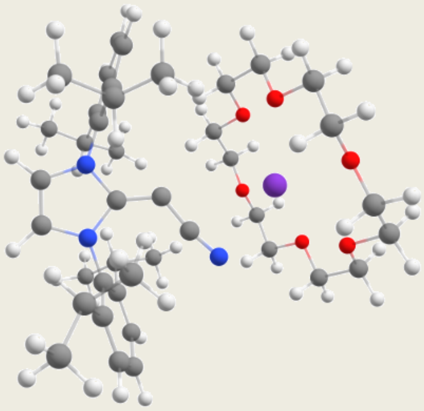

C	-3.349131	-3.515063	-1.430223
C	-0.941635	-4.168796	-1.066994
H	-1.684055	-2.183744	-1.357673
H	-4.138113	-2.784577	-1.220386
H	-3.248142	-3.607946	-2.516690
H	-3.673603	-4.489472	-1.048478
H	0.035415	-3.854774	-0.685466
H	-1.222996	-5.096605	-0.554177
H	-0.851062	-4.380741	-2.138885
C	-0.215501	-0.556967	3.215041
C	0.591565	-1.173184	4.365561
C	-0.963002	0.702626	3.677438
H	0.498363	-0.254780	2.442529
H	1.126487	-2.065386	4.025167
H	1.324180	-0.447816	4.738623
H	-0.057298	-1.454517	5.203322
H	-0.253233	1.443136	4.064128
H	-1.518605	1.157141	2.849744
H	-1.675494	0.465592	4.476144
K	3.665745	-5.318205	2.540687
O	5.838605	-3.361896	2.340331
O	5.378175	-5.287179	0.273156
O	3.995717	-3.364230	4.537132
O	4.252558	-7.763820	1.005777
O	2.945164	-5.878825	5.333284
O	2.602659	-7.874821	3.306395
H	1.645724	-8.909286	4.839662
C	6.070894	-3.111870	0.952515
C	5.341428	-2.181523	2.976396
C	6.483596	-4.385743	0.269895
C	5.661514	-6.491278	-0.432799
C	5.090634	-2.458627	4.433040
C	3.659115	-3.668058	5.887035
C	4.456355	-7.389589	-0.356291
C	3.183310	-8.698481	1.151586
C	2.507249	-4.636921	5.887079
C	1.898430	-6.850008	5.339246
C	3.035678	-9.039534	2.610373
C	2.397848	-8.116169	4.696687
H	3.338628	-8.442105	5.170934
H	6.869394	-2.361682	0.829033
H	5.155206	-2.716795	0.485291
H	6.072403	-1.360892	2.883142
H	4.401618	-1.877481	2.487288
H	6.780253	-4.149279	-0.765758
H	7.346963	-4.842100	0.781826
H	6.538386	-6.994298	0.007112
H	5.881021	-6.273094	-1.491324
H	4.851203	-1.508119	4.938914
H	5.989034	-2.886635	4.908416
H	4.527101	-4.104319	6.409013
H	3.356964	-2.752611	6.422759
H	3.565963	-6.867880	-0.746550
H	4.634230	-8.281437	-0.978651
H	3.399567	-9.618581	0.584946
H	2.244285	-8.266526	0.767355
H	1.674685	-4.233453	5.288076
H	2.160112	-4.781351	6.923400
H	1.589519	-7.070191	6.374443

```
H      1.026483   -6.467079    4.785673
H      2.294580   -9.848940    2.715609
H      3.996741   -9.395365    3.017759
```

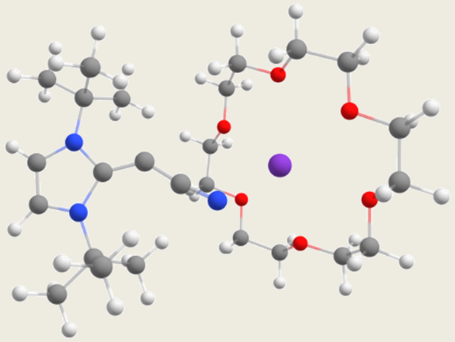

```
79
[K(18-c-6)][IDipp=CCN] @ r2SCAN-3c CPCM(THF) VERYTIGHTOPT defgrid3
C      0.057807    1.019428    0.311213
C     -1.248642    0.956278   -0.016407
H      0.644706    1.890586    0.546406
H     -1.952649    1.765810   -0.106213
N     -1.580052   -0.374830   -0.244222
N      0.575980   -0.267599    0.293260
C     -0.435887   -1.195880   -0.052776
C     -0.234500   -2.538193   -0.149014
C     -0.984010   -3.603213   -0.438169
N     -1.541018   -4.641394   -0.683464
H     -2.530738   -0.793495   -2.762569
H     -3.869577   -1.864313   -2.293039
C     -2.931440   -0.842614   -0.632464
C      1.977920   -0.613927    0.639606
C      2.650011   -1.311940   -0.551391
H      2.672061   -0.642326   -1.418909
H      3.680659   -1.574031   -0.287931
H      2.093362   -2.216499   -0.810454
C      2.764627    0.661927    0.956006
H      2.357321    1.192854    1.822959
H      3.791774    0.370775    1.194556
H      2.799760    1.344268    0.100035
C      1.991844   -1.514448    1.883219
H      1.418156   -2.424355    1.687261
H      3.025262   -1.778707    2.134450
H      1.550420   -0.987209    2.737260
C     -3.480348   -1.790395    0.444960
H     -3.543268   -1.266246    1.405707
H     -4.486341   -2.120731    0.163755
H     -2.848680   -2.671705    0.560569
C     -3.885380    0.353414   -0.735460
H     -3.994461    0.875051    0.221146
H     -3.566446    1.068017   -1.501356
H     -4.869415   -0.027968   -1.023092
C     -2.869854   -1.516111   -2.011701
H     -2.194197   -2.371939   -2.010954
K     -0.779621   -6.640621    1.115570
O      0.720331   -4.908896    2.750695
O     -2.017871   -5.287943    3.377054
O      2.134845   -6.861266    1.302160
O     -3.496485   -7.137450    1.771840
O      0.636769   -8.600295   -0.387921
O     -2.034598   -9.169497    0.428945
H     -1.912374  -10.649563   -1.034077
C      0.088011   -4.275940    3.858040
C      2.110810   -5.135196    2.949382
C     -1.352434   -4.028197    3.496022
C     -3.399400   -5.132266    3.053780
C      2.700482   -5.593825    1.641971
C      2.621200   -7.344472    0.050460
C     -4.025354   -6.496260    2.930877
C     -3.992326   -8.462695    1.605153
```

```
C       2.046803   -8.713472   -0.198397
C       0.019169   -9.873319   -0.570471
C      -3.450992   -9.020703    0.315782
C      -1.459302   -9.678959   -0.774903
H      -1.640683   -8.979666   -1.607943
H       0.573828   -3.310452    4.075028
H       0.157095   -4.910830    4.757176
H       2.613667   -4.204122    3.257084
H       2.270468   -5.892660    3.734923
H      -1.825839   -3.418784    4.282939
H      -1.404653   -3.478520    2.540654
H      -3.507508   -4.581094    2.104864
H      -3.916702   -4.567378    3.846466
H       3.794556   -5.678653    1.743892
H       2.477577   -4.854392    0.854223
H       2.334741   -6.654889   -0.761248
H       3.720687   -7.418328    0.068199
H      -3.810187   -7.095209    3.831229
H      -5.118311   -6.385715    2.840946
H      -5.093527   -8.459134    1.554507
H      -3.687202   -9.095182    2.455342
H       2.262234   -9.373423    0.657892
H       2.519417   -9.144648   -1.095852
H       0.437575  -10.378652   -1.456197
H       0.199943  -10.511751    0.309871
H      -3.922437   -9.997432    0.120273
H      -3.695231   -8.343890   -0.519400
```

5.8.6. [K(18-c-6)][NC-C-N2] (2^{CN})

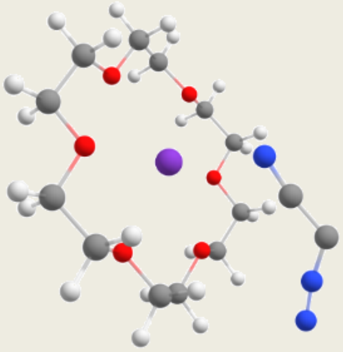

```
48
[K(18-c-6)][NC-C-N2] @ r2SCAN-3c CPCM(THF) VERYTIGHTOPT defgrid3
K      -0.497163    0.315470   -0.107273
O      -1.110817   -2.390225    0.656276
O       1.203808   -1.774401   -0.852306
O      -2.727848   -0.208452    1.546525
O       1.333231    0.655080   -2.295459
O      -2.683292    2.174006   -0.007106
O      -0.283247    2.826988   -1.390984
N       1.404094    0.637969    1.958549
C       0.091176   -3.162011    0.725076
C      -1.809675   -2.383407    1.901222
C       0.780047   -3.113372   -0.612601
C       1.952831   -1.639569   -2.056391
C      -3.073138   -1.579222    1.747847
C      -3.877111    0.615305    1.356784
C       2.452437   -0.222537   -2.147846
C       1.735384    2.022275   -2.380303
C      -3.431742    2.045063    1.203380
C      -2.244518    3.517333   -0.218846
C       0.512909    2.879038   -2.575271
C      -1.483883    3.587872   -1.515969
C       2.245471   -0.190177    1.928978
H      -0.142196   -4.209649    0.974199
H       0.757369   -2.756078    1.504157
H      -2.074506   -3.411388    2.197258
H      -1.174888   -1.948375    2.691065
H       1.649000   -3.790296   -0.589253
```

```
H       0.096321    -3.447664    -1.410806
H       1.324084    -1.883747    -2.928967
H       2.818417    -2.320628    -2.046155
H      -3.682134    -1.688687     2.659652
H      -3.658851    -1.953163     0.892162
H      -4.434429     0.290658     0.462705
H      -4.548423     0.544195     2.227907
H       3.018044     0.034776    -1.236956
H       3.126145    -0.130525    -3.014809
H       2.414293     2.169449    -3.235752
H       2.267188     2.320094    -1.461362
H      -2.812968     2.344117     2.065334
H      -4.320827     2.695198     1.173245
H      -3.109915     4.197130    -0.274939
H      -1.602425     3.842527     0.616154
H       0.828283     3.915357    -2.777305
H      -0.068372     2.518220    -3.439540
H      -1.248636     4.642441    -1.732848
H      -2.099371     3.193619    -2.341220
C       3.244827    -1.104460     2.064443
N       3.674448    -1.875311     1.156766
N       4.175449    -2.636006     0.448505
```

5.8.7. N_2

```
2
N2 @ r2SCAN-3c CPCM(THF) VERYTIGHTOPT defgrid3
N       0.000000     0.000000    -0.546922
N       0.000000     0.000000     0.546922
```

5.8.8. *N*-heterocyclic imine (5^{CN})

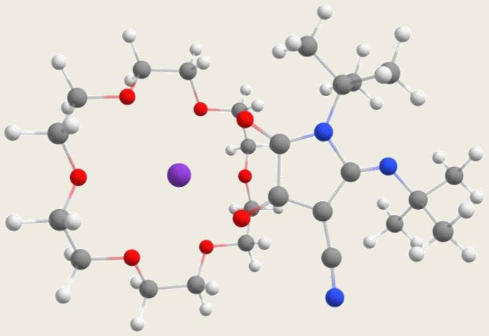

```
79
NHI (rearranged product) @ r2SCAN-3c CPCM(THF) VERYTIGHTOPT defgrid3
C       1.819390     0.873880    -2.303302
C       1.445732    -0.284953    -2.975876
C       3.592577     4.691332     0.012403
C       2.026019    -1.765511    -0.149595
H       4.221415    -2.720498    -1.590369
H       3.514934    -1.409882    -2.545682
H       5.079635    -1.173650    -1.744144
H       4.411786    -2.602138     0.887155
H       5.290937    -1.063896     0.725317
H       3.854421    -1.158828     1.765343
H       1.587921    -1.390725     0.782329
H       1.332652    -1.556298    -0.965595
H       2.130767    -2.853004    -0.066062
C       3.655357     3.158836     0.090891
C       3.255166     2.742319     1.513802
H       5.262620     1.682313    -0.112647
H       5.364400     3.049503    -1.247545
H       5.773004     3.284840     0.462724
H       2.612783     5.084390     0.286732
H       4.329575     5.070247     0.727823
C       5.105622     2.754427    -0.224534
H       3.853829     5.064445    -0.980401
H       3.892575     3.273626     2.228980
H       2.214525     3.021302     1.712894
```

```
H       3.369800    1.668835    1.662995
C       2.687896    1.082095   -1.142814
C       1.307664    2.116123   -2.725800
C       1.883436    3.165186   -1.775030
N       3.335005    0.338384   -0.339887
N       2.733058    2.522769   -0.915402
O       0.531814    2.421155   -3.645983
O       1.567501    4.350881   -1.837249
C       3.407800   -1.127412   -0.365735
C       4.090195   -1.634074   -1.646155
C       4.297656   -1.514138    0.828664
N       1.058597   -1.180010   -3.618159
K      -1.246510    4.346540   -2.697545
O      -2.609274    3.559267   -5.099123
O      -2.316484    6.329935   -4.574070
O      -2.719182    1.801207   -2.837406
O      -1.019024    7.160701   -2.194712
O      -1.330920    2.555181   -0.457094
O      -1.172639    5.368759    0.037731
H      -0.518153    4.749737    1.917838
C      -3.205996    4.567080   -5.910872
C      -3.395592    2.368500   -5.058848
C      -2.326241    5.788518   -5.895484
C      -1.550048    7.531259   -4.490265
C      -2.704203    1.350036   -4.193278
C      -2.081172    0.862173   -1.970652
C      -1.653035    8.072371   -3.089398
C      -1.108062    7.593271   -0.837650
C      -2.134782    1.379231   -0.559198
C      -1.400441    3.128902    0.847307
C      -0.423081    6.585110    0.044768
C      -0.571511    4.383553    0.879333
H       0.450688    4.169471    0.531939
H      -3.309460    4.213382   -6.949867
H      -4.209551    4.819471   -5.530079
H      -3.511096    1.954148   -6.073745
H      -4.399206    2.590914   -4.659876
H      -2.725990    6.527243   -6.608710
H      -1.301801    5.525393   -6.207575
H      -0.496277    7.331226   -4.746846
H      -1.939237    8.284601   -5.194017
H      -3.238617    0.389698   -4.280118
H      -1.665104    1.206595   -4.528085
H      -1.036645    0.710493   -2.282133
H      -2.600015   -0.109724   -2.011816
H      -2.712086    8.202451   -2.812254
H      -1.160435    9.057528   -3.047117
H      -0.611965    8.569925   -0.715091
H      -2.164850    7.702625   -0.543187
H      -3.175642    1.609786   -0.278134
H      -1.756998    0.598891    0.121796
H      -1.006814    2.423195    1.597455
H      -2.447888    3.357665    1.104582
H      -0.364488    6.989878    1.068456
H       0.600067    6.397604   -0.317383
```

5.8.9. ^{tBu}DAC-C-P(OSiMe$_3$)Ph$_2$ (10)

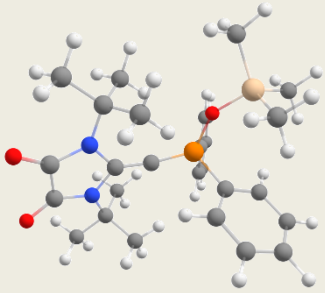

```
71
10 @ PBE0-D4/ma-def2-TZVPP VERYTIGHTOPT defgrid3 ExtremeSCF
C        0.613208000       0.307938000      -0.180953000
O        5.124396000      -1.010574000      -0.193077000
N        2.652843000       1.220104000      -1.116882000
P       -0.932072000      -0.025511000       0.118794000
Si      -2.264672000      -2.462013000       1.566826000
O       -1.350501000      -1.106651000       1.228446000
N        2.870349000      -0.589904000       0.246886000
C        1.924074000       0.308233000      -0.311012000
O        4.863305000       1.288385000      -1.867113000
C        4.100892000      -0.390888000      -0.320564000
C        3.958527000       0.828381000      -1.220348000
C        2.526943000      -1.646839000       1.232015000
C        3.781543000      -2.328536000       1.776308000
H        4.322765000      -2.884683000       1.016754000
H        3.450568000      -3.021718000       2.552377000
H        4.470435000      -1.615662000       2.226932000
C        1.822633000      -1.009134000       2.427960000
H        1.592130000      -1.785839000       3.158934000
H        0.899348000      -0.511105000       2.156249000
H        2.486623000      -0.284729000       2.903167000
C        1.671492000      -2.709985000       0.548741000
H        2.233034000      -3.172673000      -0.264976000
H        0.752830000      -2.292033000       0.147527000
H        1.415020000      -3.490739000       1.267452000
C        2.035245000       2.412985000      -1.755242000
C        1.376113000       3.264590000      -0.672224000
H        2.129774000       3.620126000       0.033209000
H        0.616746000       2.713954000      -0.125396000
H        0.911290000       4.135947000      -1.137064000
C        1.033706000       1.966417000      -2.817440000
H        0.604118000       2.846982000      -3.299528000
H        0.233193000       1.371603000      -2.385792000
H        1.537782000       1.375621000      -3.584863000
C        3.088922000       3.283695000      -2.436105000
H        3.575406000       2.781031000      -3.267661000
H        3.861162000       3.612961000      -1.743224000
H        2.568916000       4.165806000      -2.815293000
C       -1.731033000      -0.606204000      -1.386841000
C       -3.109203000      -0.496105000      -1.549094000
H       -3.704477000       0.005344000      -0.795131000
C       -3.720546000      -1.014812000      -2.679760000
H       -4.792449000      -0.920451000      -2.804269000
C       -2.960196000      -1.651303000      -3.649930000
H       -3.438802000      -2.059254000      -4.531858000
C       -1.584507000      -1.755803000      -3.495066000
H       -0.987727000      -2.244281000      -4.255539000
C       -0.970789000      -1.229771000      -2.371028000
H        0.104050000      -1.292438000      -2.247782000
C       -1.837803000       1.420164000       0.702072000
C       -2.258805000       1.534323000       2.022222000
H       -2.074485000       0.723520000       2.714630000
C       -2.906300000       2.683779000       2.450850000
H       -3.223636000       2.770149000       3.482933000
C       -3.150737000       3.716685000       1.559478000
H       -3.661980000       4.611399000       1.893533000
```

```
C        -2.738641000      3.605130000      0.238018000
H        -2.928277000      4.410745000     -0.460649000
C        -2.077240000      2.466180000     -0.186411000
H        -1.742465000      2.383598000     -1.213623000
C        -1.543984000     -3.145988000      3.140521000
H        -2.106246000     -4.019477000      3.479100000
H        -1.566341000     -2.401304000      3.939079000
H        -0.505738000     -3.449432000      2.994272000
C        -4.034554000     -1.936227000      1.826243000
H        -4.110575000     -1.150178000      2.580491000
H        -4.636677000     -2.783560000      2.164089000
H        -4.479720000     -1.562580000      0.902405000
C        -2.140653000     -3.694921000      0.177558000
H        -1.103048000     -3.971671000     -0.018258000
H        -2.569861000     -3.308602000     -0.748450000
H        -2.683660000     -4.605276000      0.445482000
```

5.8.10. ^{tBu}DAC-C(SiMe$_3$)-P(O)Ph$_2$ (10')

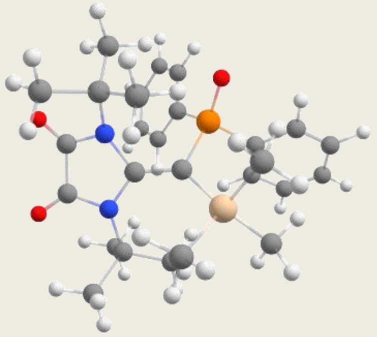

```
71
10‘ @ PBE0-D4/ma-def2-TZVPP VERYTIGHTOPT defgrid3 ExtremeSCF
C      -0.758234   -0.114465   -0.502306
        1.905676    3.708630   -0.280607
N      -1.004240    2.090483    0.687662
P       0.626399   -1.250945   -0.653583
O       1.173109   -1.645783   -1.980366
N       0.621245    1.864039   -0.880783
C      -0.444960    1.202688   -0.263221
O      -0.359766    4.188939    1.468522
C       0.949094    2.997048   -0.163979
C      -0.206755    3.218823    0.776465
C       0.996734    1.854987   -2.357020
C       0.055961    0.992687   -3.178047
H       0.157830   -0.065524   -2.962300
H       0.327793    1.141240   -4.224525
H      -0.982078    1.306001   -3.058430
C       0.845873    3.287498   -2.886325
H       0.989001    3.258432   -3.966212
H       1.573637    3.976121   -2.467428
H      -0.158942    3.672587   -2.694639
C       2.435601    1.389817   -2.512866
H       2.524232    0.327892   -2.296493
H       3.099606    1.960096   -1.864379
H       2.745053    1.549280   -3.547698
C      -1.972637    1.879535    1.822384
C      -1.254963    2.070722    3.163741
H      -0.816281    3.056816    3.275889
H      -0.478734    1.316173    3.301862
H      -1.988241    1.933580    3.959280
C      -2.585346    0.497387    1.855308
H      -1.843420   -0.294663    1.902147
H      -1.926610   -0.961698   -3.519385
H      -3.202204    0.441499    2.753894
C      -3.087427    2.909728    1.657258
H      -3.819001    2.775762    2.456170
H      -3.596062    2.771322    0.702511
H      -2.700887    3.924879    1.708678
C       0.014146   -2.732417    0.198235
```

```
C      -0.340021   -2.747189    1.543731
H      -0.249650   -1.847986    2.140005
C      -0.795703   -3.912593    2.134492
H      -1.068688   -3.916569    3.182796
C      -0.901381   -5.075655    1.383056
H      -1.262715   -5.986886    1.844295
C      -0.531979   -5.072631    0.046783
H      -0.599034   -5.982212   -0.537754
C      -0.067597   -3.905918   -0.542772
H       0.245506   -3.889181   -1.579409
C       1.942543   -0.638803    0.434430
C       3.248053   -0.925357    0.050589
H       3.414655   -1.460415   -0.876398
C       4.313834   -0.516672    0.837633
H       5.327724   -0.737227    0.526571
C       4.084082    0.180316    2.013742
H       4.917503    0.509242    2.622390
C       2.783412    0.460362    2.408526
H       2.598192    1.007998    3.324754
C       1.720414    0.046952    1.624116
H       0.709527    0.273235    1.935509
Si     -2.421227   -0.813625   -1.075996
C      -2.105001   -1.675136   -2.714748
C      -3.593525    0.608835   -1.476120
C      -3.292466   -2.107818   -0.028744
H      -3.402290   -1.871743    1.027205
H      -2.774778   -3.064948   -0.106213
H      -4.294266   -2.243483   -0.448581
H      -4.218879    0.939719   -0.647243
H      -4.265305    0.268946   -2.269049
H      -3.055458    1.479110   -1.857904
H      -3.241009    0.321101    1.011345
H      -2.998972   -2.253309   -2.966634
H      -1.254731   -2.355710   -2.685827
```

5.8.11. $SiMe_3^+$

```
13
SiMe₃⁺ @ PBE0-D4/ma-def2-TZVPP VERYTIGHTOPT defgrid3 ExtremeSCF
Si     -3.677587    0.307776    0.183769
C      -4.256498    0.109890   -1.531988
C      -2.907137    1.872147    0.710301
C      -3.869065   -1.058702    1.372960
H      -3.607201    2.697706    0.536148
H      -2.035447    2.077644    0.077897
H      -2.603190    1.869384    1.756326
H      -4.089341    0.994417   -2.145365
H      -5.325166   -0.134800   -1.528269
H      -3.751953   -0.750278   -1.987603
H      -4.340492   -1.940317    0.940445
H      -4.459370   -0.713065    2.229773
H      -2.885824   -1.330683    1.774436
```

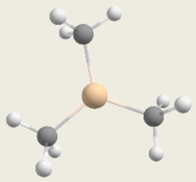

5.8.12. Transition state 1

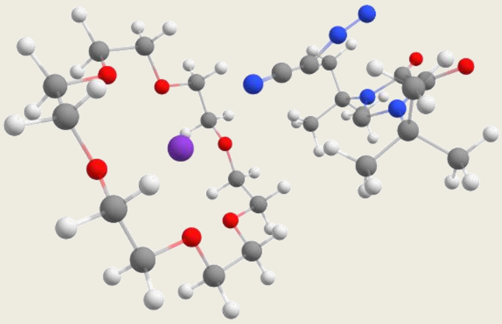

```
81
TS1 @ r2SCAN-3c CPCM(THF) VERYTIGHTOPT OptTS Freq defgrid3
K      -2.613743   -0.452192   -1.089175
O      -4.707022   -2.774028   -1.205113
O      -2.198869   -3.074035   -2.594593
O      -4.985060   -0.246034    0.479914
O      -0.740211   -0.553078   -3.301404
O      -3.422198    2.258370   -0.112147
O      -0.942772    1.925279   -1.589176
N      -0.958382   -1.121279    1.019684
C      -4.044481   -4.147987   -1.420082
C      -5.492486   -2.776332   -0.019813
C      -3.316021   -4.099513   -2.746327
C      -1.383218   -2.984208   -3.756578
C      -6.111364   -1.259392    0.164597
C      -5.467802    1.193873    0.733932
C      -0.186387   -1.937420   -3.474294
C       0.330495    0.483653   -3.148491
C      -4.207780    2.162644    1.068987
C      -2.230281    3.153854    0.102314
C      -0.303651    1.938092   -2.863009
C      -1.513198    3.272722   -1.224165
C       0.247534   -0.939585    0.777907
H      -4.843994   -5.043553   -1.502406
H      -3.252027   -4.404104   -0.529296
H      -6.373676   -3.598804   -0.149054
H      -4.792071   -3.084992    0.936487
H      -2.901861   -5.210487   -2.967992
H      -4.093622   -3.795407   -3.627280
H      -2.047361   -2.623330   -4.681198
H      -0.944437   -4.057439   -3.986516
H      -6.825689   -1.295983    1.021352
H      -6.770287   -0.924166   -0.802924
H      -6.116186    1.612664   -0.184537
H      -6.151235    1.223488    1.618383
H       0.467209   -2.270720   -2.541195
H       0.498336   -1.947991   -4.356330
H       0.923437    0.533829   -4.094917
H       1.097334    0.192979   -2.296484
H      -3.529055    1.716518    1.953854
H      -4.596501    3.256672    1.389640
H      -2.551183    4.248065    0.447708
H      -1.479706    2.706316    0.914367
H       0.549546    2.746619   -2.834690
H      -1.095325    2.231950   -3.703499
H      -0.671498    4.084349   -1.091686
H      -2.281419    3.647719   -2.045314
C       1.617945   -0.612932    0.536640
N       2.585632   -1.344586    0.081730
N       3.681857   -1.720018   -0.205623
C       2.841366    0.817813    1.697402
C       5.017591    0.665699    0.828475
C       4.916662   -0.238357    2.053669
N       3.723341    1.222204    0.691492
N       3.588220   -0.067522    2.496651
O       5.855735   -0.867352    2.574078
O       6.062022    0.927303    0.210039
```

```
C       3.055557   -0.703889    3.741557
C       1.611050   -0.211474    4.019260
C       3.074967   -2.246827    3.536087
C       3.969999   -0.285754    4.933334
H       1.269488   -0.675945    4.965627
H       1.571870    0.900630    4.137736
H       0.895212   -0.503230    3.212219
H       2.396034   -2.547687    2.695410
H       4.112203   -2.606929    3.312393
H       2.722388   -2.748777    4.458895
H       5.023619   -0.629107    4.779358
H       3.965370    0.828042    5.060582
H       3.581589   -0.738057    5.867393
C       3.349652    2.198659   -0.387905
C       3.626024    1.499332   -1.751918
C       1.853434    2.611357   -0.277970
C       4.233586    3.472308   -0.211343
H       4.732821    1.299446   -1.870429
H       3.084288    0.503463   -1.832588
H       3.267626    2.162999   -2.580297
H       1.164428    1.741577   -0.398736
H       1.643097    3.095189    0.705680
H       1.622937    3.345953   -1.082218
H       3.995146    4.199788   -1.021288
H       4.025310    3.974340    0.769548
H       5.324144    3.209753   -0.255442
```

5.8.13. Intermediate 1

```
81
INT1 @ r2SCAN-3c CPCM(THF) defgrid3 VERYTIGHTOPT OptTS Freq
K      -2.621714    0.289298   -1.114032
O      -2.719868   -2.308559    0.093968
O      -0.655115   -1.600038   -1.691158
O      -4.573794   -0.265519    0.835419
O      -0.924456    0.629049   -3.396692
O      -4.995968    1.854144   -1.015402
O      -2.796202    2.648647   -2.635170
N      -0.529448    1.096442    0.624890
C      -1.421879   -2.905072    0.140785
C      -3.308602   -2.213617    1.391673
C      -0.849410   -2.941869   -1.251400
C       0.026384   -1.514641   -2.940088
C      -4.687938   -1.624679    1.259760
C      -5.848653    0.344067    0.632245
C       0.313453   -0.064048   -3.224254
C      -0.725145    2.021035   -3.646275
C      -5.646055    1.787581    0.255833
C      -4.780686    3.204880   -1.430564
C      -2.064394    2.674478   -3.861645
C      -4.102859    3.206503   -2.774843
C       0.454780    0.464017    0.610268
H      -1.486489   -3.932244    0.534194
H      -0.760996   -2.320310    0.802575
H      -3.389411   -3.212563    1.849417
H      -2.683974   -1.582494    2.046084
H       0.111685   -3.479846   -1.225189
H      -1.528970   -3.474853   -1.936608
```

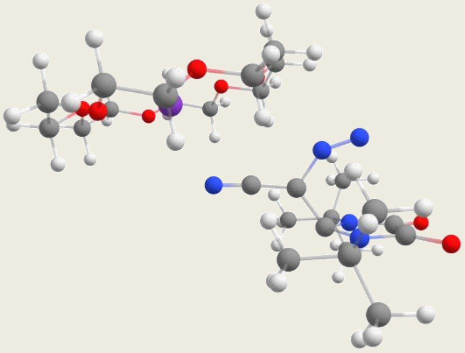

H	-0.589087	-1.952633	-3.743040
H	0.980975	-2.062849	-2.894311
H	-5.197004	-1.677516	2.235433
H	-5.277159	-2.202366	0.529022
H	-6.400014	-0.185329	-0.162103
H	-6.446378	0.299968	1.556861
H	0.880246	0.377385	-2.387198
H	0.925754	0.012738	-4.137057
H	-0.109162	2.163712	-4.548626
H	-0.203992	2.488331	-2.793814
H	-5.035782	2.296566	1.019749
H	-6.627713	2.285509	0.208638
H	-5.742423	3.735687	-1.514890
H	-4.156989	3.732945	-0.690808
H	-1.908971	3.714508	-4.190372
H	-2.622838	2.141417	-4.648346
H	-4.039630	4.244800	-3.138274
H	-4.690103	2.620602	-3.500904
C	1.651642	-0.226526	0.658882
N	1.846981	-1.320567	-0.143926
N	2.627555	-2.136290	-0.413036
C	2.841663	0.025246	1.485759
C	4.976120	-0.679016	0.965807
C	4.416368	-1.567089	2.039776
N	4.012524	0.269448	0.728365
N	3.176697	-1.056886	2.335034
O	5.023301	-2.489370	2.594896
O	6.117502	-0.753542	0.497922
C	2.313116	-1.586224	3.435197
C	1.040750	-0.750393	3.596766
C	1.936563	-3.037890	3.117654
C	3.111334	-1.495835	4.745744
H	0.557194	-1.061842	4.527960
H	1.267694	0.317952	3.665815
H	0.333245	-0.908637	2.780649
H	1.370335	-3.087173	2.181204
H	2.827232	-3.664446	3.026978
H	1.308133	-3.433360	3.922650
H	4.035123	-2.073783	4.691901
H	3.356133	-0.450870	4.965969
H	2.497246	-1.886749	5.563322
C	4.208737	1.421152	-0.205267
C	4.414874	0.881540	-1.625254
C	3.004677	2.366361	-0.186340
C	5.439984	2.208402	0.271179
H	5.288118	0.226502	-1.674233
H	3.531614	0.321424	-1.951069
H	4.565053	1.720520	-2.312929
H	2.131343	1.936238	-0.680295
H	2.730433	2.647625	0.835083
H	3.288025	3.272977	-0.730135
H	5.606561	3.052094	-0.406184
H	5.267838	2.602392	1.278905
H	6.334272	1.583392	0.281515

5.8.14. Transition state 2

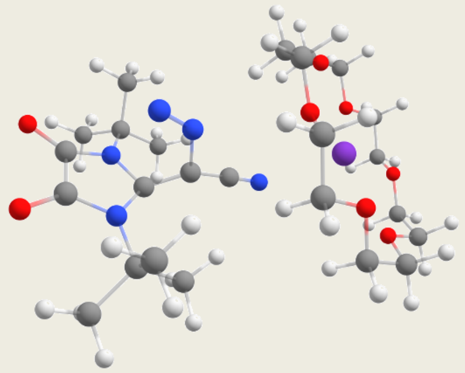

```
81
TS2 @ r2SCAN-3c CPCM(THF) defgrid3 VERYTIGHTOPT OptTS Freq
K      -2.625333    0.279578   -1.079492
O      -2.840903   -2.465563   -0.223805
O      -0.699499   -1.583590   -1.818815
O      -4.735008   -0.503458    0.630322
O      -0.870504    0.802353   -3.312484
O      -5.055882    1.792762   -1.018390
O      -2.788704    2.743769   -2.447464
N      -0.576405    0.928712    0.743228
C      -1.541784   -3.058845   -0.161158
C      -3.506899   -2.510908    1.038200
C      -0.893021   -2.961031   -1.516614
C       0.082445   -1.367041   -2.988188
C      -4.875174   -1.901017    0.889672
C      -5.995581    0.133202    0.423607
C       0.360737    0.108385   -3.100013
C      -0.670431    2.212643   -3.411948
C      -5.770578    1.605405    0.204842
C      -4.832732    3.177034   -1.294753
C      -2.001600    2.880493   -3.631281
C      -4.091466    3.306290   -2.598558
C       0.475789    0.398642    0.775347
H      -1.619165   -4.118946    0.129241
H      -0.923025   -2.534992    0.586472
H      -3.615580   -3.553833    1.376938
H      -2.923605   -1.960582    1.795528
H       0.074235   -3.487508   -1.482733
H      -1.523274   -3.439172   -2.284837
H      -0.449990   -1.732077   -3.882202
H       1.043284   -1.901838   -2.912458
H      -5.443955   -2.060499    1.819792
H      -5.417952   -2.387687    0.062985
H      -6.505367   -0.309018   -0.448050
H      -6.641492    0.000309    1.306496
H       0.837899    0.465905   -2.172640
H       1.051325    0.286484   -3.940244
H      -0.007911    2.446180   -4.261014
H      -0.199608    2.594992   -2.490501
H      -5.199964    2.031918    1.046184
H      -6.747670    2.112338    0.157319
H      -5.793890    3.709469   -1.375849
H      -4.249864    3.636805   -0.479795
H      -1.837552    3.946148   -3.858321
H      -2.519750    2.418920   -4.487687
H      -4.020342    4.374116   -2.861467
H      -4.638352    2.787364   -3.402893
C       1.705828   -0.171123    0.813495
N       1.797239   -1.585507   -0.003404
N       2.666270   -2.317432   -0.128370
C       2.909118    0.010309    1.487778
C       5.053632   -0.684806    1.063597
C       4.519468   -1.426250    2.265619
N       4.079080    0.220612    0.723386
N       3.282819   -0.887133    2.518444
O       5.131332   -2.289335    2.894299
O       6.167230   -0.848167    0.566935
```

```
C       2.427277   -1.287862    3.681161
C       1.232672   -0.342673    3.840579
C       1.937939   -2.723814    3.467400
C       3.278652   -1.180296    4.957098
H       0.779084   -0.546948    4.815441
H       1.548618    0.705389    3.822621
H       0.472863   -0.494134    3.074005
H       1.313708   -2.783947    2.569972
H       2.780605   -3.412900    3.361791
H       1.337645   -3.035008    4.328878
H       4.136192   -1.852762    4.932724
H       3.635069   -0.152713    5.088917
H       2.650939   -1.439528    5.815261
C       4.227114    1.233691   -0.370642
C       4.219143    0.511846   -1.721607
C       3.102206    2.272458   -0.314837
C       5.555096    1.978735   -0.156419
H       5.031474   -0.218491   -1.778300
H       3.266415   -0.005063   -1.872248
H       4.349460    1.243032   -2.526521
H       2.135872    1.858326   -0.603015
H       3.013572    2.706487    0.686342
H       3.358861    3.073570   -1.015223
H       5.644822    2.752806   -0.924841
H       5.563399    2.465302    0.825129
H       6.413375    1.310758   -0.227947
```

5.8.15. Transition state 3

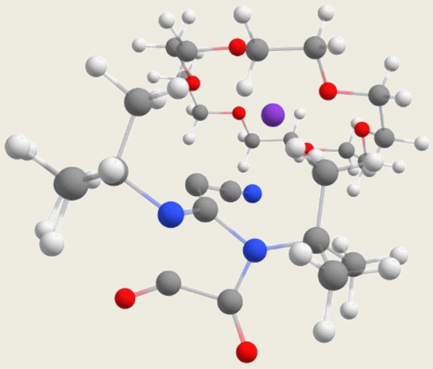

```
79
TS3 @ r2SCAN-3c CPCM(THF) defgrid3 VERYTIGHTOPT OptTS Freq
C      -1.646819   -0.709904    1.086258
C      -0.410917   -0.203925    1.394579
C      -5.115299   -3.495265    0.131860
C      -0.238886   -5.214875    0.761911
H      -0.726558   -3.200658   -1.074738
H       0.598248   -2.117195   -0.575105
H       0.960475   -3.739216   -1.203411
H       2.331825   -4.166695    0.849508
H       1.898274   -2.581651    1.533101
H       1.601755   -4.068644    2.465884
H       0.489685   -5.803532    0.194433
H      -0.311482   -5.634668    1.770083
H      -1.215225   -5.295176    0.275008
C      -4.161749   -2.306168    0.308736
C      -4.853449   -1.244275    1.180328
H      -3.207075   -0.823157   -0.964384
H      -3.309149   -2.446661   -1.691377
H      -4.764083   -1.429827   -1.580675
H      -5.326506   -3.960601    1.101131
H      -6.062849   -3.165925   -0.309176
C      -3.835367   -1.711374   -1.070984
H      -4.669189   -4.252394   -0.522628
H      -5.736114   -0.867158    0.651061
H      -5.186419   -1.674366    2.129510
H      -4.177377   -0.409621    1.384475
C      -1.847659   -2.084246    1.100299
C      -1.086377   -3.159290    2.889401
```

C	-2.334864	-2.290009	3.002096
N	-0.751119	-2.961826	1.609033
N	-2.934435	-2.868845	0.907387
O	-0.632864	-3.817847	3.810665
O	-3.142909	-1.877628	3.737498
C	0.216099	-3.750398	0.810252
C	0.258622	-3.157537	-0.597383
C	1.598945	-3.632684	1.463148
N	0.594010	0.371055	1.640924
K	1.446084	2.158887	-0.380128
O	1.602013	-0.183217	-1.902862
O	-0.631147	1.520562	-2.274313
O	3.983187	0.849636	-0.774577
O	-0.857178	3.778479	-0.529223
O	3.765176	3.161893	0.890936
O	1.560416	4.904130	0.454087
H	2.617400	6.178963	1.720490
C	0.668976	-0.365413	-2.965400
C	2.885764	-0.728306	-2.192530
C	-0.680381	0.109384	-2.497972
C	-1.876394	2.009552	-1.767488
C	3.763479	-0.546714	-0.982722
C	4.824622	1.092647	0.353715
C	-1.779373	3.499715	-1.583693
C	-0.683301	5.179182	-0.322474
C	5.011637	2.577919	0.512683
C	3.854788	4.578414	1.038271
C	0.268068	5.388620	0.825042
C	2.520043	5.105196	1.493256
H	2.200026	4.584474	2.410741
H	0.594607	-1.432882	-3.230941
H	0.993046	0.192979	-3.859129
H	2.803588	-1.805141	-2.414093
H	3.327900	-0.226068	-3.068796
H	-1.435041	-0.129219	-3.265493
H	-0.957867	-0.406227	-1.564141
H	-2.110442	1.515034	-0.809568
H	-2.689073	1.790478	-2.479371
H	4.721825	-1.064000	-1.150629
H	3.277854	-0.989116	-0.096885
H	4.370802	0.667564	1.264241
H	5.809288	0.621012	0.204617
H	-1.441849	3.977759	-2.517977
H	-2.776336	3.895767	-1.331850
H	-1.647095	5.653433	-0.076021
H	-0.288359	5.652304	-1.236618
H	5.367691	3.019282	-0.432655
H	5.770905	2.763193	1.289478
H	4.615202	4.838059	1.792448
H	4.143383	5.043005	0.081044
H	0.316202	6.464555	1.057495
H	-0.093335	4.856407	1.720295

```
79
TS3’ @ r2SCAN-3c CPCM(THF) defgrid3 VERYTIGHTOPT OptTS Freq
C      4.226165   -0.477630   -0.631307
C      4.898124   -1.320389   -1.469477
C      2.872117    2.381949    2.196095
C      1.091291   -3.847642    0.408947
H      2.473126   -4.422926   -1.856500
H      1.385011   -3.053016   -2.143562
H      3.145040   -2.816919   -2.180501
H      3.650506   -4.701633    0.354300
H      4.478275   -3.157016    0.094307
H      3.543953   -3.423778    1.583727
H      1.040389   -3.722798    1.496422
H      0.164727   -3.457046   -0.026680
H      1.162899   -4.917128    0.181360
C      2.559493    0.882439    2.116694
C      1.219340    0.577872    2.797959
H      3.475716   -0.998355    2.713275
H      4.641253    0.273373    2.274814
H      3.761020    0.362400    3.817847
H      2.081842    2.971983    1.722089
H      2.945566    2.680732    3.246767
C      3.683274    0.075610    2.764845
H      3.823079    2.600838    1.700456
H      1.270660    0.867429    3.853103
H      0.407913    1.141784    2.326147
H      0.993266   -0.491113    2.734394
C      3.041277   -0.718593    0.038131
C      1.840893   -0.134412   -1.300978
C      1.570081    0.886083   -0.208215
N      2.074325   -1.683233    0.199737
N      2.482092    0.503341    0.688265
O      1.580859   -0.414461   -2.413683
O      0.700683    1.751664   -0.240982
C      2.300124   -3.092238   -0.167768
C      2.333132   -3.349753   -1.683891
C      3.577768   -3.618632    0.505652
N      5.603374   -1.892008   -2.227797
K     -1.943115    0.943415   -0.462779
O     -2.023675   -1.874932   -0.565052
O     -2.788159   -0.545773    1.826443
O     -2.237705   -0.389168   -2.973528
O     -2.292884    2.226460    2.033611
O     -1.736619    2.401567   -2.851974
O     -2.283731    3.754176   -0.392302
H     -1.758146    5.443362   -1.494133
C     -2.623153   -2.551777    0.540104
C     -2.387969   -2.472952   -1.810485
C     -2.227544   -1.861178    1.816802
C     -2.595036    0.105600    3.083192
C     -1.724205   -1.723053   -2.933025
C     -1.692839    0.353097   -4.067916
C     -3.093534    1.522525    2.983300
C     -2.629301    3.613041    1.965303
C     -2.231314    1.758417   -4.025832
C     -2.122739    3.773281   -2.775347
C     -1.827660    4.256473    0.865104
C     -1.592941    4.354014   -1.490842
H     -0.511020    4.163578   -1.406760
```

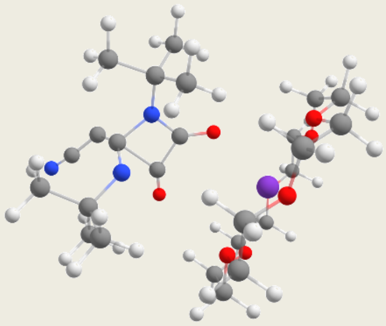

```
H      -2.280041   -3.598432    0.579084
H      -3.720071   -2.550574    0.432653
H      -2.055176   -3.523267   -1.840280
H      -3.483358   -2.451859   -1.931186
H      -2.610673   -2.447084    2.667393
H      -1.129270   -1.805538    1.899658
H      -1.527779    0.098815    3.353826
H      -3.155377   -0.418316    3.874221
H      -1.937766   -2.244172   -3.880094
H      -0.632883   -1.702285   -2.787394
H      -0.593327    0.368195   -4.003407
H      -1.979643   -0.113652   -5.023884
H      -4.151182    1.538463    2.672249
H      -3.018110    1.994464    3.976139
H      -2.395320    4.108654    2.921051
H      -3.706810    3.733440    1.766525
H      -3.333907    1.749222   -4.019436
H      -1.894711    2.293773   -4.928193
H      -1.702636    4.336011   -3.624439
H      -3.221072    3.863837   -2.808812
H      -1.967501    5.348398    0.912898
H      -0.755507    4.035755    0.995594
```

5.8.16. [K(18-c-6)][ItBu-C(N$_2$)(CN)] (I*t*Bu-INT1)

```
81
[K(18-c-6)][ItBu-C(N2)(CN)]  @  r2SCAN-3c  VERYTIGHTOPT  Freq  CPCM(THF)  defgrid3
ExtremeSCF
H      -0.600493   -4.173954    2.111773
H       0.049060   -3.949740    3.750836
H       1.736011   -3.900941    1.205875
C      -2.538555   -0.249216   -0.936059
C      -2.199156    0.762476   -2.037441
C      -3.101411   -1.541164   -1.548193
C      -3.591364    0.373275   -0.021149
N      -3.371568   -1.295443    3.965931
H       2.691852   -2.450404    1.618291
H       2.299797   -3.642055    2.863678
H       1.443335   -1.875269    4.290103
H       1.674578   -0.624446    3.047618
H       0.076886   -0.849774    3.790591
H      -3.126418    1.076794   -2.525825
H      -1.714778    1.644896   -1.606037
H      -1.549442    0.342312   -2.810094
H      -2.388638   -1.989692   -2.248193
H      -3.318626   -2.266435   -0.756226
H      -4.030252   -1.326177   -2.087106
H      -3.926226   -0.323649    0.748208
C      -2.629404   -0.911600    3.128414
C      -1.794667   -0.385555    2.183191
N      -1.414371    1.035213    2.386910
N      -0.862411    1.733018    1.592742
C      -1.197701   -1.090585    1.102968
C       0.632824   -1.731942   -0.091590
C      -0.181850   -1.060359   -0.933653
H      -3.209688    1.275986    0.462685
N       0.065475   -1.724861    1.164387
N      -1.274922   -0.592663   -0.206238
```

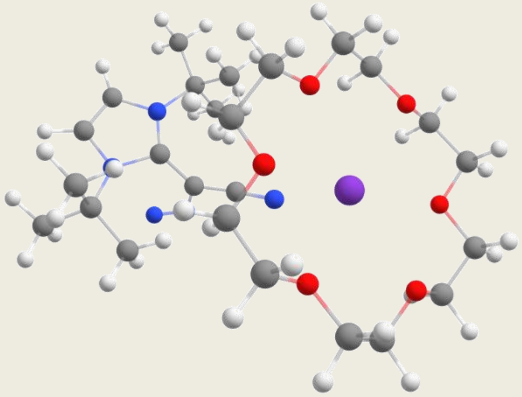

```
H     -1.314146   -2.961962    3.194386
H     -4.450460    0.643816   -0.644580
C      0.631206   -2.403593    2.359962
C     -0.378069   -3.430830    2.885733
C      1.917184   -3.138943    1.971279
C      0.972446   -1.366714    3.441894
H      1.582612   -2.192155   -0.309083
H     -0.037781   -0.847052   -1.979923
K     -5.191447   -3.294820    4.650185
O     -3.257938   -5.052822    3.638220
O     -5.304198   -4.143309    1.889405
O     -3.709776   -5.041040    6.426611
O     -7.194407   -2.265213    2.930958
O     -5.580859   -3.148663    7.460466
O     -7.682715   -2.316331    5.728589
H     -8.453210   -1.456542    7.464601
C     -3.417139   -5.544957    2.311198
C     -2.648648   -5.997420    4.511130
C     -3.974286   -4.437691    1.456895
C     -5.888903   -3.094359    1.117247
C     -2.437376   -5.341687    5.850184
C     -3.579391   -4.426607    7.709085
C     -7.281513   -2.829265    1.623054
C     -8.477124   -2.005474    3.496910
C     -4.952064   -4.149546    8.260937
C     -6.896142   -2.841626    7.921855
C     -8.289929   -1.368763    4.848131
C     -7.489655   -1.778016    7.037503
H     -6.821579   -0.901963    6.993589
H     -2.444433   -5.858333    1.898557
H     -4.093578   -6.415943    2.302848
H     -1.670187   -6.313576    4.113224
H     -3.285540   -6.891599    4.616310
H     -3.978717   -4.760551    0.402906
H     -3.339967   -3.538432    1.543960
H     -5.277734   -2.180800    1.198653
H     -5.939282   -3.383580    0.054718
H     -1.874105   -6.027890    6.502993
H     -1.850307   -4.416581    5.727241
H     -3.007082   -3.487822    7.624761
H     -3.044231   -5.096284    8.401553
H     -7.862293   -3.765978    1.649573
H     -7.784800   -2.128141    0.937736
H     -9.048145   -1.315283    2.854835
H     -9.049042   -2.943276    3.592181
H     -5.555426   -5.072076    8.258448
H     -4.855777   -3.801177    9.301973
H     -6.860490   -2.465854    8.957219
H     -7.525797   -3.746267    7.902858
H     -9.273025   -1.060578    5.238814
H     -7.655189   -0.471751    4.759687
```

5.8.17. [K(18-c-6)][ItBu---C(N_2)(CN)] (I*t*Bu-TS1)

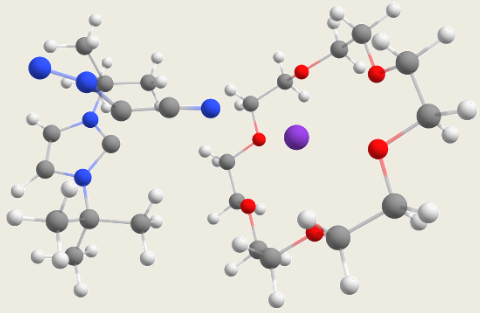

```
81
[K(18-c-6)][ItBu-C(N2)(CN)]  @  r2SCAN-3c  VERYTIGHTOPT  OptTS  Freq  CPCM(THF)
defgrid3 ExtremeSCF
H      -0.785032   -4.107784    2.321810
H       0.176168   -3.872095    3.799596
H       1.386405   -4.361485    0.988805
C      -2.362830   -0.404511   -1.012721
C      -2.370160    1.111756   -0.769215
C      -2.329675   -0.694341   -2.519636
C      -3.617137   -1.040997   -0.416251
N      -3.667896   -1.240223    3.610983
H       2.694283   -3.173240    1.199351
H       2.220028   -4.205202    2.550867
H       1.928070   -2.058799    3.934822
H       2.258439   -1.044297    2.514952
H       0.728236   -0.849596    3.405661
H      -3.245733    1.563428   -1.247820
H      -2.404177    1.315745    0.305103
H      -1.470602    1.574942   -1.189277
H      -1.504810   -0.180818   -3.022260
H      -2.240538   -1.770174   -2.705674
H      -3.261854   -0.339936   -2.970593
H      -3.625059   -2.121871   -0.591581
C      -2.750684   -0.724573    3.071743
C      -1.673932   -0.295622    2.320032
N      -1.193029    1.040623    2.753488
N      -0.430171    1.899224    2.583449
C      -1.128127   -1.613944    0.855025
C       0.975568   -1.436137   -0.046305
C       0.121155   -0.834378   -0.906082
H      -3.676230   -0.864210    0.659333
N       0.207639   -1.948798    0.993672
N      -1.156697   -0.988023   -0.379380
H      -1.045020   -2.667336    3.320961
H      -4.496039   -0.602102   -0.900523
C       0.800934   -2.627506    2.169786
C      -0.288602   -3.360724    2.949703
C       1.839368   -3.649976    1.687836
C       1.472831   -1.574931    3.063963
H       2.046907   -1.547888   -0.112999
H       0.329333   -0.337433   -1.841158
K      -5.372713   -3.202241    4.647014
O      -3.373584   -4.933727    3.673259
O      -5.578584   -4.305628    1.987310
O      -3.681488   -4.699208    6.473977
O      -7.543626   -2.475540    2.985698
O      -5.618031   -2.844125    7.456501
O      -7.873299   -2.327721    5.799515
H      -8.615266   -1.384542    7.504545
C      -3.584224   -5.552661    2.407354
C      -2.676186   -5.770185    4.589594
C      -4.258345   -4.558753    1.500043
C      -6.267404   -3.354820    1.174063
C      -2.427617   -4.988505    5.852156
C      -3.514597   -3.953780    7.680225
C      -7.650504   -3.145737    1.730047
C      -8.812624   -2.274039    3.603618
```

C	-4.865429	-3.716408	8.299841
C	-6.924612	-2.597136	7.974955
C	-8.610011	-1.512391	4.886233
C	-7.653546	-1.664552	7.045356
H	-7.065176	-0.745428	6.888103
H	-2.620852	-5.846885	1.959443
H	-4.204890	-6.457184	2.520467
H	-1.706090	-6.079587	4.166913
H	-3.264659	-6.677094	4.807616
H	-4.301860	-4.970671	0.478641
H	-3.676455	-3.622661	1.475266
H	-5.717544	-2.399792	1.159654
H	-6.347690	-3.724394	0.138709
H	-1.794515	-5.585243	6.528518
H	-1.898123	-4.051199	5.614959
H	-3.018332	-2.992121	7.467758
H	-2.888999	-4.514518	8.393445
H	-8.160134	-4.114917	1.857469
H	-8.235138	-2.536681	1.021703
H	-9.473730	-1.689174	2.943590
H	-9.296210	-3.243798	3.806874
H	-5.397514	-4.673423	8.426402
H	-4.728910	-3.261521	9.294222
H	-6.859195	-2.128983	8.970472
H	-7.478698	-3.544964	8.073574
H	-9.593866	-1.256525	5.311263
H	-8.063638	-0.575584	4.688028

6. References

[1] J. P. Moerdyk, C. W. Bielawski, "Reductive generation of stable, five-membered N,N′-diamidocarbenes" *Chem. Commun.* **2014**, *50*, 4551–4553.

[2] T. W. Hudnall, J. P. Moerdyk, C. W. Bielawski, "Ammonia N–H activation by a N,N′-diamidocarbene" *Chem. Commun.* **2010**, *46*, 4288–4290.

[3] M. Jörges, S. Mondal, M. Kumar, P. Duari, F. Krischer, J. Löffler, V. H. Gessner, "Phosphinoyl-Substituted Ketenyl Anions: Synthesis and Substituent Effects on the Structural Properties" *Organometallics* **2024**, *43*, 585–593.

[4] R. J. Ward, M. Jörges, H. Remm, E. Kiliani, F. Krischer, Q. Le Dé, V. H. Gessner, "An Azide-Free Synthesis of Metallodiazomethanes Using Nitrous Oxide" *J. Am. Chem. Soc.* **2024**, *146*, 24602–24608.

[5] Q. Le Dé, Y. Zhang, L. Zhao, F. Krischer, K. Feichtner, G. Frenking, V. H. Gessner, "Isolation and Structure Elucidation of the Heterocumulene Anions [NCC-L]− (L=CO, CS, N2)" *Angew. Chem. Int. Ed.* **2025**, *64*, e202422496.

[6] G. M. Sheldrick, "Crystal structure refinement with SHELXL" *Acta Cryst. C* **2015**, *71*, 3–8.

[7] G. M. Sheldrick, "SHELXT - integrated space-group and crystal-structure determination" *Acta Cryst. A* **2015**, *71*, 3–8.

[8] G. M. Sheldrick, "A short history of SHELX" *Acta. Cryst. A* **2007**, *64*, 112–122.

[9] G. M. Sheldrick, "Crystal structure refinement with SHELXL" *Acta. Cryst. C* **2015**, *71*, 3–8.

[10] A. Thorn, B. Dittrich, G. M. Sheldrick, "Enhanced rigid-bond restraints" *Acta. Cryst. A* **2012**, *68*, 448–451.

[11] F. Neese, "Software Update: The ORCA Program System—Version 6.0" *WIREs Comput. Mol. Sci.* **2025**, *15*, e70019.

[12] F. Neese, J. Wiley, "The ORCA program system" *WIREs Comput. Mol. Sci* **2012**, *2*, 73–78.

[13] E. D. Glendening, K. B. J, A. E. Reed, J. E. Carpenter, J. A. Bohmann, C. M. Morales, P. Karafiloglou, C. R. Landis, F. Weinhold, **2018**, Theoretical Chemistry Institute preprint.

[14] S. Grimme, A. Hansen, S. Ehlert, J.-M. Mewes, J.-M. Mewes, "r2SCAN-3c: A 'Swiss army knife' composite electronic-structure method" *J. Chem. Phys* **2021**, *154*, 64103.

[15] M. Ernzerhof, G. E. Scuseria, "Assessment of the Perdew–Burke–Ernzerhof exchange-correlation functional" *J. Chem. Phys.* **1999**, *110*, 5029–5036.

[16] C. Adamo, V. Barone, "Toward reliable density functional methods without adjustable parameters: The PBE0 model" *J. Chem. Phys.* **1999**, *110*, 6158–6170.

[17] E. Caldeweyher, J. M. Mewes, S. Ehlert, S. Grimme, "Extension and evaluation of the D4 London-dispersion model for periodic systems" *Phys. Chem. Chem. Phys.* **2020**, *22*, 8499–8512.

[18] E. Caldeweyher, S. Ehlert, A. Hansen, H. Neugebauer, S. Spicher, C. Bannwarth, S. Grimme, "A generally applicable atomic-charge dependent London dispersion correction" *J. Chem. Phys.* **2019**, *150*, 154122.

[19] J. Zheng, X. Xu, D. G. Truhlar, "Minimally augmented Karlsruhe basis sets" *Theor Chem Acc* **2011**, *128*, 295–305.

[20] F. Weigend, R. Ahlrichs, "Balanced basis sets of split valence, triple zeta valence and quadruple zeta valence quality for H to Rn: Design and assessment of accuracy" *Phys. Chem. Chem. Phys.* **2005**, *7*, 3297–3305.

[21] C. Fonseca Guerra, J. W. Handgraaf, E. J. Baerends, F. M. Bickelhaupt, "Voronoi Deformation Density (VDD) Charges: Assessment of the Mulliken, Bader, Hirshfeld, Weinhold, and VDD Methods for Charge Analysis" *J. Comput. Chem.* **2004**, *25*, 189–210.

[22] C. Bannwarth, S. Ehlert, S. Grimme, "GFN2-xTB - An Accurate and Broadly Parametrized Self-Consistent Tight-Binding Quantum Chemical Method with Multipole Electrostatics and Density-Dependent Dispersion Contributions" *J. Chem. Theory Comput.* **2019**, *15*, 1652–1671.

[23] P. Pracht, S. Grimme, C. Bannwarth, F. Bohle, S. Ehlert, G. Feldmann, J. Gorges, M. Müller, T. Neudecker, C. Plett, S. Spicher, P. Steinbach, P. A. Wesołowski, F. Zeller, "CREST—A program for the exploration of low-energy molecular chemical space" *J. Chem. Phys.* **2024**, *160*, 114110.

[24] P. Pracht, F. Bohle, S. Grimme, "Automated exploration of the low-energy chemical space with fast quantum chemical methods" *Phys. Chem. Chem. Phys.* **2020**, *22*, 7169–7192.

[25] S. Grimme, "Semiempirical GGA-type density functional constructed with a long-range dispersion correction" *J. Comput. Chem.* **2006**, *27*, 1787–1799.

[26] S. Grimme, F. Bohle, A. Hansen, P. Pracht, S. Spicher, M. Stahn, "Efficient Quantum Chemical Calculation of Structure Ensembles and Free Energies for Nonrigid Molecules" *J. Phys. Chem. A* **2021**, *125*, 4039–4054.

[27] C. Adamo, V. Barone, "Toward reliable density functional methods without adjustable parameters: The PBE0 model" *J Chem Phys* **1999**, *110*, 6158–6170.

[28] E. D. Glendening, C. R. Landis, F. Weinhold, "Resonance Theory Reboot" *J. Am. Chem. Soc.* **2019**, *141*, 4156–4166.

[29] T. Lu, F. Chen, "Multiwfn: A multifunctional wavefunction analyzer" *J. Comput. Chem.* **2012**, *33*, 580–592.

[30] T. Lu, "A comprehensive electron wavefunction analysis toolbox for chemists, Multiwfn" *J. Chem. Phys.* **2024**, *161*, DOI 10.1063/5.0216272/3309709.

[31] A. Savin, R. Nesper, S. Wengert, T. F. Fässler, "ELF: The electron localization function" *Angew. Chem Int. Ed. Engl.* **1997**, *36*, 1808–1832.

[32] C. Bannwarth, E. Caldeweyher, S. Ehlert, A. Hansen, P. Pracht, J. Seibert, S. Spicher, S. Grimme, “Extended tight-binding quantum chemistry methods” *WIREs Comput. Mol. Sci.* **2021**, *11*, e1493.

[33] A. V. Marenich, C. J. Cramer, D. G. Truhlar, “Universal solvation model based on solute electron density and on a continuum model of the solvent defined by the bulk dielectric constant and atomic surface tensions” *J. Phys. Chem. B* **2009**, *113*, 6378–6396.

[34] V. Barone, M. Cossi, “Quantum Calculation of Molecular Energies and Energy Gradients in Solution by a Conductor Solvent Model” *J. Phys. Chem. A* **1998**, *102*, 1995–2001.

[35] K. P. Zois, A. A. Danopoulos, D. Tzeli, “N-Heterocyclic Carbenes: A Benchmark Study on their Singlet–Triplet Energy Gap as a Critical Molecular Descriptor” *ChemPhysChem* **2025**, *26*, e202500012.

[36] B. Helmich-Paris, B. de Souza, F. Neese, R. Izsák, “An improved chain of spheres for exchange algorithm” *J. Chem. Phys.* **2021**, *155*, DOI 10.1063/5.0058766/1013244.

[37] A. Hellweg, C. Hättig, S. Höfener, W. Klopper, “Optimized accurate auxiliary basis sets for RI-MP2 and RI-CC2 calculations for the atoms Rb to Rn” *Theor. Chem. Acc.* **2007**, *117*, 587–597.

[38] Christopher J. Cramer, *Essentials of Computational Chemistry Theories and Models Second Edition*, John Wiley & Sons, Ltd, Department of Chemistry and Supercomputing Institute, University of Minnesota, USA, **2004**.

[39] J. P. Perdew, K. Burke, M. Ernzerhof, “Generalized Gradient Approximation Made Simple [Phys. Rev. Lett. 77, 3865 (1996)]” *Phys. Rev. Lett.* **1997**, *78*, 1396.

[40] J. P. Perdew, K. Burke, M. Ernzerhof, “Generalized Gradient Approximation Made Simple” *Phys. Rev. Lett.* **1996**, *77*, 3865.

[41] V. Ásgeirsson, B. O. Birgisson, R. Bjornsson, U. Becker, F. Neese, C. Riplinger, H. Jónsson, “Nudged Elastic Band Method for Molecular Reactions Using Energy-Weighted Springs Combined with Eigenvector following” *J. Chem. Theory Comput.* **2021**, *17*, 4929–4945.

[42] S. Ehlert, M. Stahn, S. Spicher, S. Grimme, “Robust and efficient implicit solvation model for fast semiempirical methods” *J. Chem. Theory Comput.* **2021**, *17*, 4250–4261.
